# Supplementary material for: Electrochemical cobalt-catalyzed semi-deuteration of alkynes to access deuterated Z-alkenes
Source: Nat Commun. 2025 Mar 10;16:2390. doi: 10.1038/s41467-025-57782-x (PMC11893763; doi:10.1038/s41467-025-57782-x)
Supplement: Supplementary file 1 — Supplementary Information [file 41467_2025_57782_MOESM1_ESM.pdf]

Supplementary Information

Electrochemical Cobalt-Catalysed Semi-Deuteration of Alkynes to Access Deuterated *Z*-Alkenes

Wen-Jie Feng[1,†], Zhe Chang[1,†], Xi Lu[1,*,‡], Yao Fu[1,*,‡]

[1] State Key Laboratory of Precision and Intelligent Chemistry, University of Science and Technology of China, Hefei 230026 (China)

[†] These authors contributed equally: Wen-Jie Feng, Zhe Chang

[‡] Joint supervision: Xi Lu, Yao Fu

E-Mails: luxi@mail.ustc.edu.cn (Xi Lu); fuyao@ustc.edu.cn (Yao Fu)

Table of contents

Supplementary Methods 3
General Information 3
Optimization of Reaction Conditions. 4
Standard Condition 6
Semi-hydrogenation Condition 7
Modified Condition 8
Preparation of Substrates 9
Characterization Data of Alkynes 11
Characterization Data of Products 19
Supplementary Discussions 35
Control experiments 35
^{18}O labelling experiments 35
D sources experiments 39
Non-rinse experiments 42
CV experiments 45
Kinetic experiments 49
Supplementary Figures of NMR Spectra 53
Supplementary References 132

Supplementary Methods

General Information

Materials

The following chemicals were purchased and used as received without further purification unless otherwise noted: cobalt(II) bromide (CAS: 7789-43-7, Leyan.com, 1229292); 4,4'-di-*tert*-butyl-2,2'-bipyridine (CAS: 72914-19-3, Bidepharm, BD217706); *N*,*N*-dimethylacetamide (CAS: 127-19-5, Adamas, 011342855); *N,N*-dimethylformamide (CAS: 68-12-2, Adamas, 011342852); triphenylphosphine (CAS: 603-35-0, Macklin, T742559-100g); acetic acid-*d* (CAS: 758-12-3, Energy chemical, E090026); deuterium oxide (CAS: 7789-20-0, J&K chemical, 261750).

Electrodes (graphite, Zn and Mg) were purchased from www.1688.com.

IKA Electra-Syn 2.0 was used for electrolysis experiments.

CV experiments were recorded using a Signal 1000E electrochemical workstation which was purchased from Wuxi Signal Technology Co., Ltd.

Analytical methods

^{1}H NMR, ^{13}C NMR and ^{19}F NMR spectra were recorded on Bruker 400 MHz, 500 MHz or 600 M spectrometer at 298 K in $CDCl_3$ unless otherwise noted. ^{2}H NMR spectra were recorded on Bruker 400 MHz, 500 MHz or 600 M spectrometer at 298 K in $CHCl_3$ unless otherwise noted. Data for ^{1}H NMR were reported as follows: chemical shift (δ ppm), multiplicity, coupling constant (Hz), and integration. Data for ^{13}C NMR were reported as follows: chemical shift (δ ppm), multiplicity, and coupling constant (Hz). Data for ^{19}F NMR were reported as follows: chemical shift (δ ppm), multiplicity, and coupling constant (Hz). Data for ^{2}H NMR were reported as follows: chemical shift (δ ppm), multiplicity, and coupling constant (Hz). Chemical shifts were reported using the residual solvent $CHCl_3$ as the internal reference for ^{1}H NMR (δ = 7.260 ppm) and $CDCl_3$ peak as the internal reference for ^{13}C NMR (δ = 77.160 ppm). High-resolution mass spectral analysis (HRMS) data were acquired on Water XEVO G2 Q-TOF (Waters Corporation) or Gas Chromatography Q Exactive Orbitrap Mass Sepctrometer (Thermo-Fisher). Gas chromatographic (GC) analysis was acquired on a Shimadzu GC-2010 plus Series GC system equipped with a flame-ionization detector. Organic solutions were concentrated under reduced pressure on Buchi rotary evaporator. Column chromatographic purification of products was accomplished using forced-flow chromatography on Silica Gel (300-400 mesh).

Optimization of Reaction Conditions.

Supplementary Table 1. Optimization of reaction conditions for cobalt-electro-catalysis.

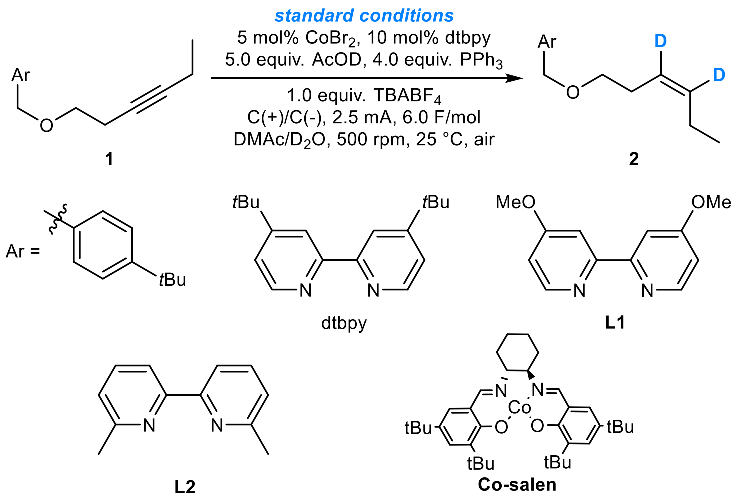

Entry	Variation	GC Yield[a] (%)	D content (%)
1	none	92 (88[b])	92 / 91
2	$Co(OAc)_2$ instead of $CoBr_2$	71	89 / 91
3	$Co(acac)_2$ instead of $CoBr_2$	79	90 / 91
4	$CoCl_2$ instead of $CoBr_2$	81	90 / 91
5	Co-salen instead of $CoBr_2$ and dtbpy	11	nd / nd
6	**L1** instead of dtbpy	63	90 / 91
7	**L2** instead of dtbpy	14	nd / nd
8	Et_3N instead of PPh_3	16	nd / nd
9	TEOA instead of PPh_3	41	72 / 70
10	DIPEA instead of PPh_3	33	88 / 87
11	DABCO instead of PPh_3	44	93 / 93
12	DMF instead of DMAc	81	92 / 91
13	DMSO instead of DMAc	80	91 / 92
14	MeCN instead of DMAc	45	90 / 91
15	EtOH instead of D_2O	61	5 / 5
16	*t*BuOH instead of D_2O	34	7 / 7
17	TBAI instead of $TBABF_4$	60	91 / 92
18	TBAB instead of $TBABF_4$	57	91 / 93
19	Zn(+)/C(-) instead of C(+)/C(-), w/o PPh_3	18	nd / nd
20	Mg(+)/C(-) instead of C(+)/C(-), w/o PPh_3	51	90 / 91
21	C(+)/Ni Foam(-) instead of C(+)/C(-)	32	nd /nd-
22	5.0 mA instead of 2.5 mA	56	92 / 94
23	w/o AcOD	30	nd / nd
24	w/o D_2O	17	nd / nd
25	0.2 ml D_2O used	87(82[b])	92 / 91

Optimization of reaction conditions. Standard conditions: **1** (1.0 equiv.), cobalt catalyst (5 mol%), ligand (10 mol%), AcOD (5.0 equiv.), sacrificial reductant (4.0 equiv.),

electrolyte (1.0 equiv.), C(+)/C(-), 2.5 mA, 6.0 F/mol, non-deuterated solvent (3.6 mL), D_2O (0.4 mL), 500 rpm, 25 °C, under air. 0.2 mmol scales, [a]GC (gas chromatography) yield using triphenylmethane as an internal standard. The D content was determined by ^{1}H-NMR. [b] Isolated yields are given in parentheses. $Co(OAc)_2$, cobalt acetate; $Co(acac)_2$, bis(acetylacetonato)cobalt; AcOH, acetic acid; DMAc, *N*,*N*-dimethylacetamide; DMF, *N*,*N*-dimethylformamide; DMSO, dimethyl sulfoxide; $TBABF_4$, tetrabutylammonium tetrafluoroborate; TBAI, tetrabutylammonium iodide; TBAB, tetrabutylammonium bromide; TEOA, triethanolamine; DIPEA, *N*,*N*-diisopropylethylamine; DABCO, triethylenediamine; rpm, revolutions per minute; GC, gas chromatography; nd, not determined, D content was not detected when the GC yield was <30%.

To a 5 mL vial equipped with a magnetic stirring bar, cobalt source (0.01 mmol, 5 mol%), ligand (0.02 mmol, 10 mol%), electrolyte (0.20 mmol, 1.0 equiv.), and sacrificial reductant (0.80 mmol, 4.0 equiv.) were added. Subsequently, non-deuterated solvent (3.6 mL) was added, and the solution was stirred for 5 minutes to ensure complete dissolution. To this solution, Na_2SO_4 was added for the purposes of drying and dehydration. After allowing the solution to stand for 30 minutes, Na_2SO_4 was removed by filtration, and the resulting solution was then transferred into a 5 mL ElectraSyn vial that was equipped with a magnetic stirring bar. Alkyne (0.20 mmol, 1.0 equiv.), AcOD (1.0 mmol, 5.0 equiv.), D_2O (0.4 mL), were added to the solution in ElectraSyn vial via syringe. The Electra-Syn vial cap, equipped with an anode (graphite) and a cathode (graphite), was inserted into the reaction mixture. After pre-stirring for 5 minutes, the reaction mixture was electrolyzed under a constant current of 2.5 mA for 6 F/mol. After the reaction, the electrodes were washed with ethyl acetate, and the organic phases were collected. Added triphenylmethane as an internal standard into the organic phases and mixed evenly. Took a solution of organic phase, added ethyl acetate (EtOAc) and water for extraction, then took the organic layer and test the yield by GC. The D content was determined by ^{1}H-NMR after flash chromatography, when the GC yield was ≥30%.

Note: the graphite electrode can be reused after polished with a sand paper (800 grit) and washed with ethyl acetate (three times), acetone (three times). The graphite electrode was 53 mm * 8 mm * 2 mm.

The instructions for using ElectraSyn 2.0 (IKA) can be found in the literature[1].

Standard Condition

To a 5 mL vial equipped with a magnetic stirring bar, $CoBr_2$ (0.01 mmol, 5 mol%), dtbpy (0.02 mmol, 10 mol%), $TBABF_4$ (0.20 mmol, 1.0 equiv.), and PPh_3 (0.80 mmol, 4.0 equiv.) were added. Subsequently, DMAc (3.6 mL) was added, and the solution was stirred for 5 minutes to ensure complete dissolution. To this solution, Na_2SO_4 was added for the purposes of drying and dehydration. After allowing the solution to stand for 30 minutes, Na_2SO_4 was removed by filtration, and the resulting solution was then transferred into a 5 mL Electra-Syn vial that was equipped with a magnetic stirring bar. Another method involves preparing 30 mL of a standard solution in a 50 mL vial, which contains $CoBr_2$ (2.78 mmol/L), dtbpy (5.56 mmol/L), PPh_3 (222 mmol/L), $TBABF_4$ (55.6 mmol/L) in DMAc. This solution was dried with Na_2SO_4, and subsequently, the Na_2SO_4 was removed by filtration. Afterward, 3.6 mL of this standard solution was transferred into a 5 mL ElectraSyn vial equipped with a magnetic stirring bar. The standard solution is sufficient for use in a batch of 6 parallel reactions. Following the solution preparation method, alkyne (0.20 mmol, 1.0 equiv.), AcOD (1.0 mmol, 5.0 equiv.), and D_2O (0.4 mL), were added to the solution in ElectraSyn vial via syringe. The Electra-Syn vial cap, equipped with an anode (graphite) and a cathode (graphite), was inserted into the reaction mixture. After pre-stirring for 5 minutes, the reaction mixture was electrolyzed under a constant current of 2.5 mA for 6 F/mol. The voltage of the electrolytic cell was approximately 2.0 V and increased over time. After the reaction, the electrodes were washed with ethyl acetate, and the organic phases were collected. The mixture was diluted with H_2O, followed by extraction with ethyl acetate, dried with anhydrous Na_2SO_4, and concentrated in vacuo. The residue was purified by flash column chromatography to yield the target product.

Semi-hydrogenation Condition

To a 5 mL Electrasyn-vial equipped with a magnetic stirring bar, $CoBr_2$ (0.01 mmol, 5 mol%), dtbpy (0.02 mmol, 10 mol%), $TBABF_4$ (0.20 mmol, 1.0 equiv.), and PPh_3 (0.80 mmol, 4.0 equiv.) were added. Subsequently, DMAc (3.6 mL) was added, and the solution was stirred for 5 minutes to ensure complete dissolution. Another method involves preparing 30 mL of a standard solution in a 50 mL vial, which contains $CoBr_2$ (2.78 mmol/L), dtbpy (5.56 mmol/L), PPh_3 (222 mmol/L), $TBABF_4$ (55.6 mmol/L) in DMAc. Afterward, 3.6 mL of this standard solution was transferred into a 5 mL ElectraSyn vial equipped with a magnetic stirring bar. The standard solution is sufficient for use in a batch of 6 parallel reactions. Following the solution preparation method, alkyne (0.20 mmol, 1.0 equiv.), AcOH (1.0 mmol, 5.0 equiv.), and H_2O (0.4 mL), were added to the solution in ElectraSyn vial via syringe. The ElectraSyn vial cap, equipped with an anode (graphite) and a cathode (graphite), was inserted into the reaction mixture. After pre-stirring for 5 minutes, the reaction mixture was electrolyzed under a constant current of 2.5 mA for 6 F/mol. The voltage of the electrolytic cell was approximately 2.0 V and increased over time. After the reaction, the electrodes were washed with ethyl acetate, and the organic phases were collected. The mixture was diluted with H_2O, followed by extraction with ethyl acetate, dried with anhydrous Na_2SO_4, and concentrated in vacuo. The residue was purified by flash column chromatography to yield the target product.

Modified Condition

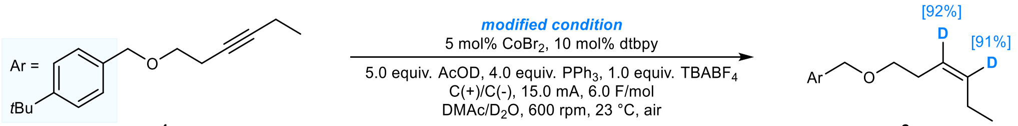

1 4.5 mmol, 1.098 g

2 0.941 g, 85% yield, >20:1 *Z*:*E*

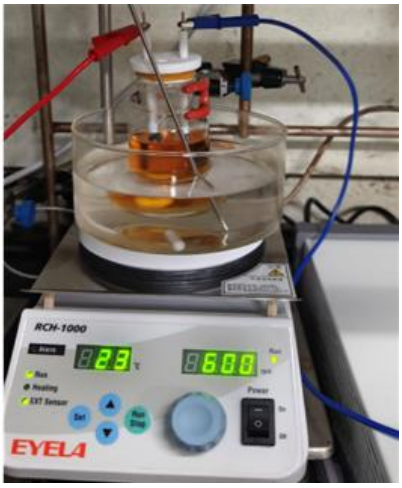

Supplementary Figure 1. Gram scale experiment

To a 250 mL flask equipped with a magnetic stirring bar, $CoBr_2$ (0.225 mmol, 5 mol%), dtbpy (0.45 mmol, 10 mol%), $TBABF_4$ (4.5 mmol, 1.0 equiv.), and PPh_3 (18 mmol, 4.0 equiv.) were added. Subsequently, DMAc (90.0 mL) was added, and the solution was stirred for 10 minutes to ensure complete dissolution. To this solution, Na_2SO_4 was added for the purposes of drying and dehydration. After allowing the solution to stand for 30 minutes, Na_2SO_4 was removed by filtration, and the resulting solution was then transferred into another 250 mL flask that was equipped with a magnetic stirring bar. Following the solution preparation method, alkyne (4.5 mmol, 1.0 equiv.), AcOD (22.5 mmol, 5.0 equiv.), and D_2O (12.0 mL), were added to the solution in the flask. The flask cap, equipped with an anode (graphite) and a cathode (graphite), was inserted into the reaction mixture. After pre-stirring for 10 minutes, the reaction mixture was electrolyzed under a constant current of 15 mA for 6 F/mol. After the reaction, the electrodes were washed with ethyl acetate, and the organic phases were collected. The mixture was diluted with H_2O, followed by extraction with ethyl acetate, dried with anhydrous Na_2SO_4, and concentrated in vacuo. The residue was purified by flash column chromatography to yield the target product.

Preparation of Substrates

The following alkyne substrates can be purchased and used directly: **S14**, **S15**, **S16**, **S17**, **S18**, **S22**, **S26** and **S30**. The following alkyne substrates were synthesized according to literature procedures: **1**, **S3**, **S4**, **S5**, **S6**, **S7**, **S8**, **S9**, **S10**, **S11**, **S12**, **S13**, **S19**, **S20**, **S21**, **S23**, **S24**, **S25**, **S27**, **S29**, **31**, **33**, **35**.

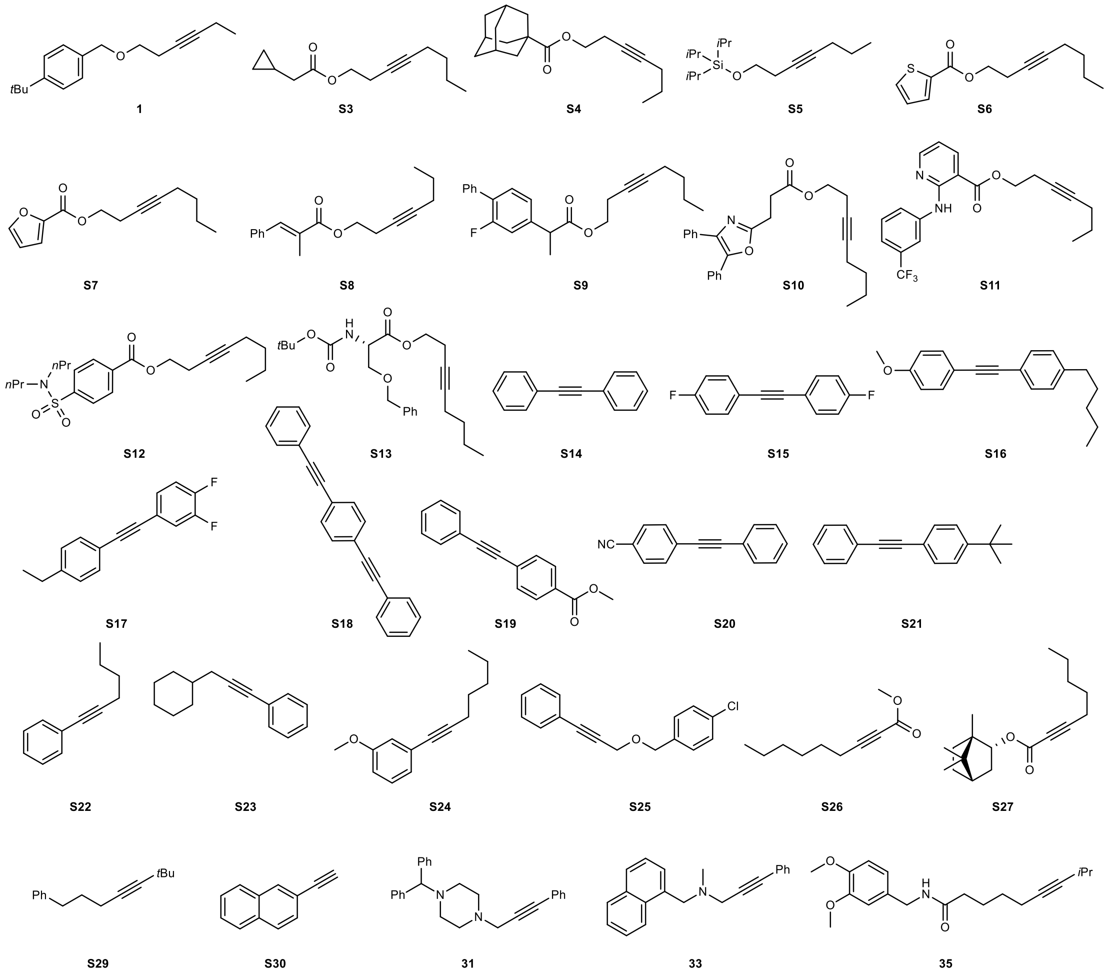

Supplementary Figure 2. Alkyne substrates

Synthesis of internal Alkynes

General Process 1.

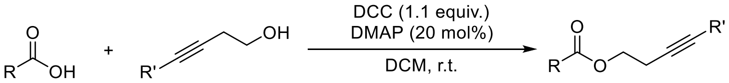

1, **S3**, **S4**, **S5**, **S6**, **S7**, **S8**, **S9**, **S10**, **S11**, **S12**, **S13**, **S27** were synthesized by **general process 1**. By analogy to a modified literature procedure[1-5]. The acid (1.0 equiv.), DCC (1.1 equiv.), and DMAP (0.2 equiv.) were added to a 10 mL Schlenk flask equipped

with a stir bar under N_2. Then, DCM (6 mL) and the alcohol (5 mmol, 1.0 equiv.) were added sequentially. The reaction mixture was stirred at room temperature overnight. Subsequently, 15 mL of water were added, and the reaction mixture was extracted with EtOAc (3×5 mL), washed with brine, and dried over Na_2SO_4. After filtration, the solvent was removed under reduced pressure. The residue was purified by silica gel column chromatography using petroleum ether and ethyl acetate as the eluent.

General Process 2.

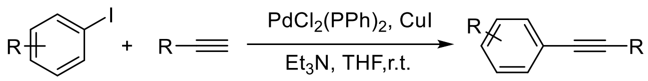

S19, **S20**, **S21**, **S23**, **S24**, **S25** were synthesized by **general process 2**. By analogy to a modified literature procedure[6-14]. The corresponding aryl iodide (1.0 equiv.), $Pd(PPh_3)_2Cl_2$ (2 mol%), CuI (1 mol%), and alkynes (1.1 equiv.) were added to a 10 mL Schlenk flask equipped with a stir bar under a nitrogen atmosphere (N_2). Subsequently, THF (4 mL) and Et_3N (2 mL) were added in sequence. The reaction mixture was stirred at room temperature overnight. Afterward, 15 mL of water was added, and the reaction mixture was extracted with EtOAc (3×5 mL), washed with brine, and dried over Na_2SO_4. Following filtration, the solvent was removed under reduced pressure. The residue was then purified by silica gel column chromatography using a mixture of petroleum ether and ethyl acetate as the eluent.

Characterization Data of Alkynes

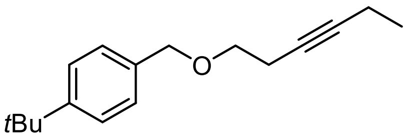

1-(*tert*-butyl)-4-((hex-3-yn-1-yloxy)methyl)benzene (**1**)[15]

Compound **1** was isolated as yellow oil. (1.96 g,10 mmol, 80%).

<u>^{1}H NMR</u> (500 MHz, Chloroform-*d*) δ 7.40 – 7.37 (m, 2H), 7.32 – 7.28 (m, 2H), 4.54 (s, 2H), 3.57 (t, *J* = 7.2 Hz, 2H), 2.48 (tt, *J* = 7.2, 2.4 Hz, 2H), 2.18 (qt, *J* = 7.5, 2.4 Hz, 2H), 1.34 (s, 9H), 1.14 (t, *J* = 7.5 Hz, 3H).

<u>^{13}C NMR</u> (101 MHz, Chloroform-*d*) δ 150.67, 135.25, 127.69, 125.40, 82.86, 76.10, 72.80, 68.89, 34.61, 31.45, 20.24, 14.30, 12.53.

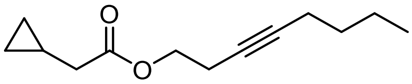

oct-3-yn-1-yl 2-cyclopropylacetate (**S3**)

Compound **S3** was isolated as colorless oil. (0.86 g, 83%).

<u>^{1}H NMR</u> (500 MHz, Chloroform-*d*) δ 4.12 (t, *J* = 6.9 Hz, 2H), 2.46 (tt, *J* = 7.0, 2.4 Hz, 2H), 2.19 (d, *J* = 7.2 Hz, 2H), 2.11 (tt, *J* = 7.2, 2.4 Hz, 2H), 1.48 – 1.29 (m, 4H), 1.08 – 0.97 (m, 1H), 0.87 (t, *J* = 7.2 Hz, 3H), 0.54 – 0.46 (m, 2H), 0.17 – 0.09 (m, 2H).

<u>^{13}C NMR</u> (126 MHz, Chloroform-*d*) δ 173.02, 81.93, 75.53, 62.79, 39.30, 31.00, 21.91, 19.32, 18.39, 13.64, 6.91, 4.38.

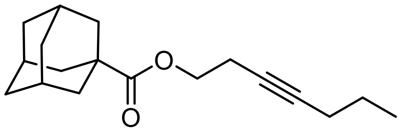

hept-3-yn-1-yl (*3r,5r,7r*)-adamantane-1-carboxylate (**S4**)

Compound **S4** was isolated as colorless oil. (1.03 g, 75%).

<u>^{1}H NMR</u> (500 MHz, Chloroform-*d*) δ 4.10 (t, *J* = 6.9 Hz, 2H), 2.56 – 2.40 (m, 2H), 2.14 – 2.08 (m, 2H), 1.88 (d, *J* = 3.0 Hz, 6H), 1.76 – 1.61 (m, 7H), 1.49 (q, *J* = 7.3 Hz, 2H), 1.35 – 1.24 (m, 2H), 0.95 (d, *J* = 7.4 Hz, 3H).

<u>^{13}C NMR</u> (126 MHz, Chloroform-*d*) δ 177.57, 81.80, 75.91, 62.56, 40.80, 38.92, 36.64, 28.08, 22.43, 20.81, 19.39, 13.56.

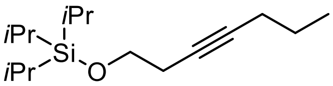

(hept-3-yn-1-yloxy)triisopropylsilane[1] (**S5**)

Compound **S5** was isolated as colorless oil.

^{1}H NMR (400 MHz, Chloroform-*d*) δ 3.76 (t, *J* = 7.4 Hz, 2H), 2.40 (tt, *J* = 7.4, 2.4 Hz, 2H), 2.11 (tt, *J* = 7.1, 2.4 Hz, 2H), 1.49 (h, *J* = 7.3 Hz, 2H), 1.07 (q, *J* = 4.5 Hz, 21H), 0.95 (t, *J* = 7.3 Hz, 3H).

^{13}C NMR (101 MHz, Chloroform-*d*) δ 81.33, 77.13, 62.81, 23.40, 22.54, 20.91, 18.08, 13.61, 12.13.

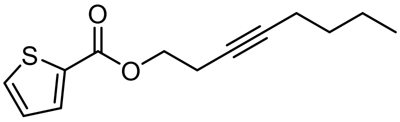

oct-3-yn-1-yl thiophene-2-carboxylate (**S6**)

Compound **S6** was isolated as colorless oil. (0.99 g, 84%).

^{1}H NMR (400 MHz, Chloroform-*d*) δ 7.81 (dd, *J* = 3.8, 1.3 Hz, 1H), 7.55 (dd, *J* = 5.0, 1.3 Hz, 1H), 7.09 (dd, *J* = 5.0, 3.7 Hz, 1H), 4.34 (t, *J* = 7.1 Hz, 2H), 2.71 – 2.53 (m, 2H), 2.24 – 2.10 (m, 2H), 1.59 – 1.30 (m, 4H), 0.87 (t, *J* = 7.2 Hz, 3H).

^{13}C NMR (126 MHz, Chloroform-*d*) δ 161.96, 133.67, 133.56, 132.51, 127.74, 82.25, 75.29, 63.44, 30.95, 21.89, 19.43, 18.39, 13.63.

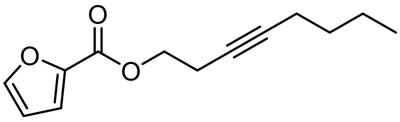

oct-3-yn-1-yl furan-2-carboxylate (**S7**)

Compound **S7** was isolated as colorless oil. (0.95 g, 86%).

^{1}H NMR (400 MHz, Chloroform-*d*) δ 7.57 (dd, *J* = 1.8, 0.9 Hz, 1H), 7.18 (dd, *J* = 3.5, 0.9 Hz, 1H), 6.50 (dd, *J* = 3.5, 1.7 Hz, 1H), 4.35 (t, *J* = 7.1 Hz, 2H), 2.65 – 2.53 (m, 2H), 2.19 – 2.01 (m, 2H), 1.52 – 1.31 (m, 4H), 0.86 (t, *J* = 7.1 Hz, 3H).

^{13}C NMR (126 MHz, Chloroform-*d*) δ 158.43, 146.42, 144.53, 118.10, 111.85, 82.29, 75.13, 63.21, 30.91, 21.85, 19.38, 18.35, 13.59.

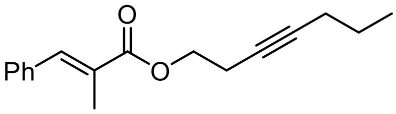

hept-3-yn-1-yl (*E*)-2-methyl-3-phenylacrylate (**S8**)

Compound **S8** was isolated as colorless oil. (0.99 g, 77%).

^{1}H NMR (500 MHz, Chloroform-*d*) δ 7.72 (d, *J* = 1.6 Hz, 1H), 7.40 (d, *J* = 4.4 Hz, 4H), 7.33 (q, *J* = 4.3 Hz, 1H), 4.28 (t, *J* = 7.0 Hz, 2H), 2.68 – 2.55 (m, 2H), 2.21 – 2.09 (m, 5H), 1.51 (q, *J* = 7.2 Hz, 2H), 0.97 (t, *J* = 7.4 Hz, 3H).

^{13}C NMR (101 MHz, Chloroform-*d*) δ 168.44, 139.15, 135.91, 129.71, 128.42, 128.39, 128.34, 81.95, 75.82, 63.36, 22.37, 20.75, 19.43, 14.10, 13.50.

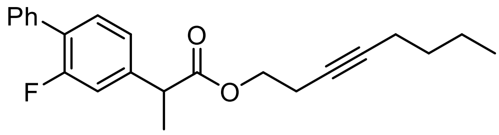

oct-3-yn-1-yl 2-(2-fluoro-[1,1'-biphenyl]-4-yl)propanoate (**S9**)

Compound **S9** was isolated as white solid. (1.43 g, 81%).

^{1}H NMR (500 MHz, Chloroform-*d*) δ 7.54 (dt, *J* = 8.1, 1.5 Hz, 2H), 7.44 (t, *J* = 7.6 Hz, 2H), 7.40 – 7.35 (m, 2H), 7.19 – 7.12 (m, 2H), 4.17 (qt, *J* = 10.6, 6.9 Hz, 2H), 3.78 (q, *J* = 7.2 Hz, 1H), 2.48 (tt, *J* = 6.9, 2.3 Hz, 2H), 2.12 (tt, *J* = 7.0, 2.3 Hz, 2H), 1.55 (d, *J* = 7.2 Hz, 3H), 1.47 – 1.33 (m, 4H), 0.89 (t, *J* = 7.2 Hz, 3H).

^{13}C NMR (101 MHz, Chloroform-*d*) δ 173.79, 160.96, 158.49, 141.81 (d, *J* = 7.7 Hz), 135.55 (d, *J* = 1.4 Hz), 130.81 (d, *J* = 3.9 Hz), 128.99 (d, *J* = 2.9 Hz), 128.51, 127.72, 123.64 (d, *J* = 3.3 Hz), 115.35 (d, *J* = 23.6 Hz), 82.13, 75.36, 63.40, 45.03, 31.00, 21.95, 19.26, 18.48, 18.38, 13.67.

^{19}F NMR (376 MHz, Chloroform-*d*) δ -117.56.

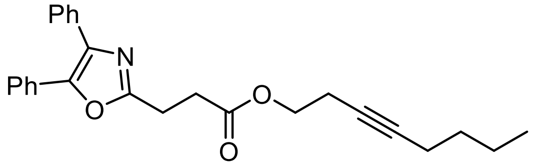

oct-3-yn-1-yl 3-(4,5-diphenyloxazol-2-yl)propanoate (**S10**)

Compound **S10** was isolated as white solid. (1.53 g, 76%).

^{1}H NMR (400 MHz, Chloroform-*d*) δ 7.67 – 7.59 (m, 2H), 7.58 – 7.50 (m, 2H), 7.34 – 7.22 (m, 6H), 4.16 (t, *J* = 7.0 Hz, 2H), 3.15 (dd, *J* = 8.4, 6.7 Hz, 2H), 2.90 (dd, *J* = 8.3, 6.8 Hz, 2H), 2.46 (tt, *J* = 7.0, 2.4 Hz, 2H), 2.10 (tt, *J* = 6.9, 2.4 Hz, 2H), 1.46 – 1.30 (m, 4H), 0.86 (t, *J* = 7.1 Hz, 3H).

^{13}C NMR (126 MHz, Chloroform-*d*) δ 171.89, 161.81, 145.52, 135.18, 132.50, 129.05, 128.74, 128.66, 128.56, 128.17, 127.99, 126.55, 82.21, 75.41, 63.31, 31.16, 31.04,

23.60, 21.99, 19.36, 18.46, 13.72.

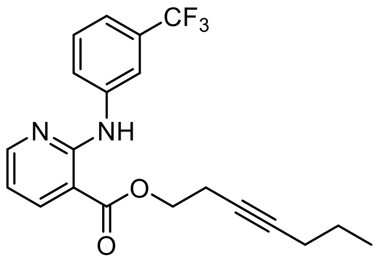

hept-3-yn-1-yl 2-((3-(trifluoromethyl)phenyl)amino)nicotinate (**S11**)

Compound **S11** was isolated as yellow solid. (1.36 g, 74%).

^{1}H NMR (500 MHz, Chloroform-*d*) δ 10.33 (s, 1H), 8.42 (dd, *J* = 4.8, 2.0 Hz, 1H), 8.32 (dd, *J* = 7.8, 2.0 Hz, 1H), 8.08 (q, *J* = 2.5, 2.1 Hz, 1H), 7.87 (dd, *J* = 8.0, 2.3 Hz, 1H), 7.44 (t, *J* = 7.9 Hz, 1H), 7.30 (d, *J* = 7.8 Hz, 1H), 6.81 (dd, *J* = 7.8, 4.7 Hz, 1H), 4.41 (t, *J* = 6.8 Hz, 2H), 2.66 (tt, *J* = 6.9, 2.4 Hz, 2H), 2.13 (tt, *J* = 7.0, 2.4 Hz, 2H), 1.49 (h, *J* = 7.2 Hz, 2H), 0.95 (t, *J* = 7.4 Hz, 3H).

^{13}C NMR (126 MHz, Chloroform-*d*) δ 167.06, 155.57, 152.91, 140.39, 140.09, 131.03 (q, *J* = 31.9 Hz), 129.12, 124.05 (q, *J* = 272.6 Hz), 123.46, 119.01 (q, *J* = 3.7 Hz), 117.08 (q, *J* = 4.0 Hz), 113.96, 107.36, 82.26, 75.17, 63.64, 22.13, 20.55, 19.24, 13.31.

^{19}F NMR (376 MHz, Chloroform-*d*) δ -62.60.

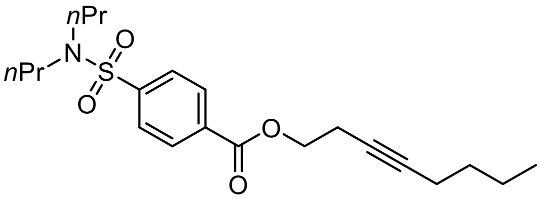

oct-3-yn-1-yl 4-(*N,N*-dipropylsulfamoyl)benzoate (**S12**)

Compound **S12** was isolated as white solid. (1.6 g, 81%).

^{1}H NMR (400 MHz, Chloroform-*d*) δ 8.19 – 8.10 (m, 2H), 7.91 – 7.79 (m, 2H), 4.38 (t, *J* = 6.9 Hz, 2H), 3.11 – 3.02 (m, 4H), 2.61 (tt, *J* = 6.9, 2.4 Hz, 2H), 2.12 (tt, *J* = 7.0, 2.4 Hz, 2H), 1.57 – 1.46 (m, 4H), 1.46 – 1.29 (m, 4H), 0.84 (td, *J* = 7.3, 2.2 Hz, 9H).

^{13}C NMR (101 MHz, Chloroform-*d*) δ 165.08, 144.30, 133.47, 130.35, 127.03, 82.39, 75.27, 63.96, 49.95, 30.95, 21.97, 21.90, 19.41, 18.39, 13.66, 11.21.

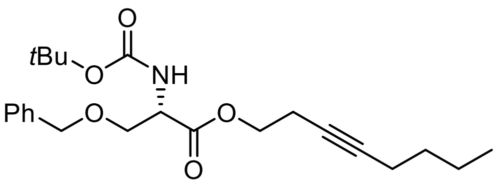

oct-3-yn-1-yl O-benzyl-*N*-(*tert*-butoxycarbonyl)-*L*-serinate (**S13**)

Compound **S13** was isolated as white solid. (1.68 g, 83%).

^{1}H NMR (500 MHz, Chloroform-*d*) δ 7.34 (dd, *J* = 8.0, 6.4 Hz, 2H), 7.32 – 7.24 (m, 3H), 5.41 (d, *J* = 8.8 Hz, 1H), 4.57 – 4.45 (m, 2H), 4.45 (dt, *J* = 8.9, 3.1 Hz, 1H), 4.19 (q, *J* = 6.7 Hz, 2H), 3.88 (dd, *J* = 9.4, 3.3 Hz, 1H), 3.70 (dd, *J* = 9.3, 3.3 Hz, 1H), 2.45 (tt, *J* = 7.2, 3.6 Hz, 4H), 2.11 (tt, *J* = 6.9, 2.2 Hz, 2H), 1.45 (s, 11H), 1.41 – 1.32 (m, 1H), 0.89 (t, *J* = 7.2 Hz, 3H).

^{13}C NMR (126 MHz, Chloroform-*d*) δ 170.50, 155.53, 137.62, 128.47, 127.87, 127.64, 82.21, 79.96, 75.15, 73.29, 70.10, 63.85, 54.05, 30.98, 28.36, 21.95, 19.22, 18.40, 13.66.

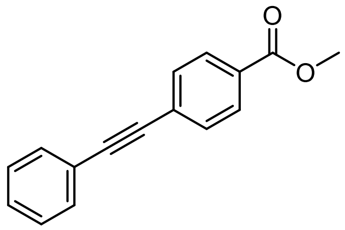

methyl 4-(phenylethynyl)benzoate[12] (**S19**)

Compound **S19** was isolated as yellow solid. (1.04 g, 88%).

^{1}H NMR (400 MHz, Chloroform-*d*) δ 8.02 (d, *J* = 8.4 Hz, 2H), 7.59 (d, *J* = 8.4 Hz, 2H), 7.57 – 7.52 (m, 2H), 7.41 – 7.33 (m, 3H), 3.93 (s, 3H).

^{13}C NMR (101 MHz, Chloroform-*d*) δ 166.70, 131.86, 131.63, 129.65, 129.57, 128.89, 128.57, 128.12, 122.81, 92.48, 88.75, 52.37.

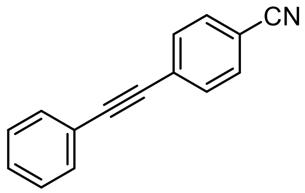

4-(phenylethynyl)benzonitrile[12] (**S20**)

Compound **S20** was isolated as yellow solid. (0.81 g, 80%).

^{1}H NMR (400 MHz, Chloroform-*d*) δ 7.70 – 7.59 (m, 4H), 7.57 – 7.50 (m, 2H), 7.42 – 7.35 (m, 3H).

^{13}C NMR (101 MHz, Chloroform-*d*) δ 132.18, 132.16, 131.90, 129.25, 128.63, 128.34, 122.32, 118.66, 111.56, 93.89, 87.84.

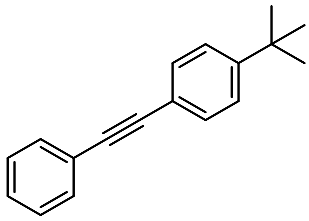

1-(*tert*-butyl)-4-(phenylethynyl)benzene[10] (**S21**)

Compound **S21** was isolated as yellow solid. (1.03 g, 88%).

^{1}H NMR (400 MHz, Chloroform-*d*) δ 7.51 – 7.44 (m, 2H), 7.43 – 7.39 (m, 2H), 7.34 – 7.22 (m, 5H), 1.26 (s, 9H).

^{13}C NMR (101 MHz, Chloroform-*d*) δ 151.65, 131.71, 131.47, 128.44, 128.20, 125.49, 123.63, 120.36, 89.66, 88.86, 34.92, 31.32.

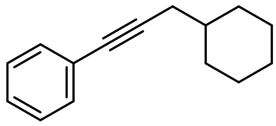

(3-cyclohexylprop-1-yn-1-yl)benzene[12] (**S23**)

Compound **S23** was isolated as colorless oil. (0.85 g, 86%).

^{1}H NMR (400 MHz, Chloroform-*d*) δ 7.41 – 7.37 (m, 2H), 7.28 – 7.23 (m, 3H), 2.29 (d, *J* = 6.7 Hz, 2H), 1.91 – 1.82 (m, 2H), 1.74 (dt, *J* = 12.1, 3.1 Hz, 2H), 1.69 – 1.64 (m, 1H), 1.61 – 1.50 (m, 1H), 1.36 – 1.15 (m, 3H), 1.06 (qd, *J* = 12.1, 2.9 Hz, 2H).

^{13}C NMR (101 MHz, Chloroform-*d*) δ 131.67, 128.29, 127.54, 124.28, 89.47, 81.58, 37.65, 32.91, 27.36, 26.43, 26.31.

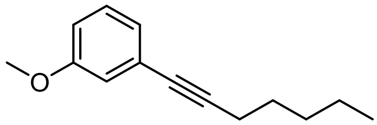

1-(hept-1-yn-1-yl)-3-methoxybenzene[16] (**S24**)

Compound **S24** was isolated as colorless oil. (0.91 g, 90%).

^{1}H NMR (400 MHz, Chloroform-*d*) δ 7.20 (t, *J* = 7.9 Hz, 1H), 7.01 (dt, *J* = 7.5, 1.2 Hz, 1H), 6.95 (dd, *J* = 2.7, 1.5 Hz, 1H), 6.84 (ddd, *J* = 8.3, 2.6, 1.0 Hz, 1H), 3.79 (s, 3H), 2.41 (t, *J* = 7.2 Hz, 2H), 1.68 – 1.58 (m, 2H), 1.51 – 1.33 (m, 4H), 0.94 (t, *J* = 7.1 Hz, 3H).

^{13}C NMR (101 MHz, Chloroform-*d*) δ 159.35, 129.29, 125.21, 124.17, 116.48, 114.09, 90.42, 80.57, 55.21, 31.24, 28.56, 22.35, 19.45, 14.10.

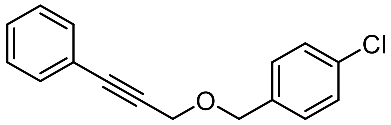

1-chloro-4-(((3-phenylprop-2-yn-1-yl)oxy)methyl)benzene (**S25**)

Compound **S25** was isolated as colorless oil. (1.12 g, 87%).

^{1}H NMR (400 MHz, Chloroform-*d*) δ 7.44 – 7.38 (m, 2H), 7.29 – 7.24 (m, 7H), 4.57 (s, 2H), 4.34 (s, 2H).

^{13}C NMR (126 MHz, Chloroform-*d*) δ 136.09, 133.68, 131.84, 129.48, 128.66, 128.62, 128.40, 122.57, 86.77, 84.85, 70.86, 58.09.

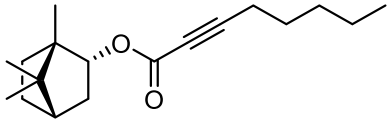

(*1S,2R,4S*)-1,7,7-trimethylbicyclo[2.2.1]heptan-2-yl oct-2-ynoate (**S27**)

Compound **S27** was isolated as colorless oil. (1.12 g, 81%).

^{1}H NMR (500 MHz, Chloroform-*d*) δ 4.94 (ddd, *J* = 10.0, 3.5, 2.0 Hz, 1H), 2.39 – 2.28 (m, 3H), 2.03 – 1.92 (m, 1H), 1.74 (dtd, *J* = 16.2, 7.9, 7.5, 3.0 Hz, 1H), 1.67 (t, *J* = 4.6 Hz, 1H), 1.58 (p, *J* = 7.2 Hz, 2H), 1.44 – 1.22 (m, 6H), 1.03 (dd, *J* = 13.9, 3.5 Hz, 1H), 0.94 – 0.82 (m, 12H).

^{13}C NMR (126 MHz, Chloroform-*d*) δ 154.49, 89.18, 81.69, 73.61, 48.94, 47.95, 44.88, 36.58, 31.11, 28.01, 27.35, 27.13, 22.19, 19.77, 18.89, 18.79, 13.97, 13.55.

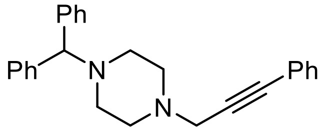

1-benzhydryl-4-(3-phenylprop-2-yn-1-yl)piperazine (**31**)[17]

Compound **31** was isolated as yellow solid. (1.32 g, 72%).

^{1}H NMR (400 MHz, Chloroform-*d*) δ 7.46 – 7.37 (m, 6H), 7.32 – 7.27 (m, 4H), 7.27 – 7.22 (m, 3H), 7.21 – 7.05 (m, 2H), 4.23 (s, 1H), 3.53 (s, 2H), 2.69 (brs, 4H), 2.49 (brs, 4H).

^{13}C NMR (101 MHz, Chloroform-*d*) δ 142.84, 131.88, 128.61, 128.38, 128.24, 128.04, 127.06, 123.25, 85.44, 84.57, 76.34, 52.46, 51.91, 47.70.

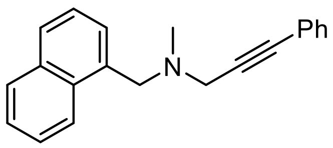

N-methyl-*N*-(naphthalen-1-ylmethyl)-3-phenylprop-2-yn-1-amine (**33**)[17]

Compound **33** was isolated as yellow solid. (1.27 g, 89%).

^{1}H NMR (500 MHz, Chloroform-*d*) δ 8.38 (dd, *J* = 8.4, 1.2 Hz, 1H), 7.89 (dd, *J* = 8.1, 1.5 Hz, 1H), 7.83 (d, *J* = 8.2 Hz, 1H), 7.61 – 7.49 (m, 5H), 7.45 (dd, *J* = 8.3, 6.9 Hz, 1H), 7.40 – 7.33 (m, 3H), 4.10 (s, 2H), 3.60 (s, 2H), 2.50 (s, 3H).

^{13}C NMR (126 MHz, Chloroform-*d*) δ 134.34, 133.99, 132.72, 131.86, 128.55, 128.44, 128.33, 128.18, 127.87, 126.14, 125.76, 125.26, 124.77, 123.45, 86.07, 84.63, 58.32, 45.97, 42.31.

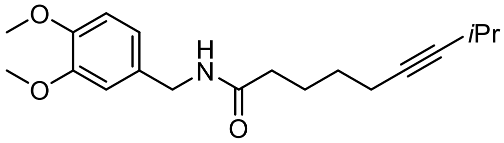

N-(3,4-dimethoxybenzyl)-8-methylnon-6-ynamide (**35**)[18]

Compound **35** was isolated as white solid. (1.24 g, 78%).

^{1}H NMR (400 MHz, Chloroform-*d*) δ 6.80 (s, 3H), 5.81 (s, 1H), 4.36 (d, *J* = 5.6 Hz, 2H), 3.85 (d, *J* = 1.2 Hz, 6H), 2.48 (dtd, *J* = 8.9, 6.8, 4.7 Hz, 1H), 2.22 (t, *J* = 7.6 Hz, 2H), 2.15 (td, *J* = 7.1, 2.2 Hz, 2H), 1.84 – 1.67 (m, 2H), 1.55 – 1.41 (m, 2H), 1.10 (dd, *J* = 6.8, 0.5 Hz, 6H).

^{13}C NMR (126 MHz, Chloroform-*d*) δ 172.71, 149.21, 148.52, 131.05, 120.17, 111.21, 111.19, 86.60, 78.73, 56.03, 55.96, 43.53, 36.34, 28.72, 25.04, 23.51, 20.58, 18.58.

Characterization Data of Products

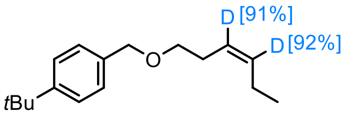

(Z)-1-(*tert*-butyl)-4-(((hex-3-en-1-yl-3,4-d_2)oxy)methyl)benzene (**2**)

Following **modified condition**, **2** was obtained as yellow oil (0.941 g, 85% yield, Z:E > 20:1, 92% D, 91% D).

Following **standard condition**, **2** was obtained as yellow oil (43.7 mg, 88% yield, Z:E > 20:1, 92% D, 91% D).

^{1}H NMR (500 MHz, Chloroform-d) δ 7.43 – 7.37 (m, 2H), 7.37 – 7.29 (m, 2H), 5.50 (tt, J = 7.2, 1.6 Hz, 0.08H), 5.40 (tt, J = 7.3, 1.6 Hz, 0.09H), 4.52 (s, 2H), 3.51 (t, J = 7.1 Hz, 2H), 2.40 (t, J = 6.9 Hz, 2H), 2.09 (q, J = 7.5 Hz, 2H), 1.35 (s, 9H), 1.00 (t, J = 7.6 Hz, 3H).

^{13}C NMR (126 MHz, Chloroform-d) δ 150.57, 135.63, 133.31 (dd, J = 45.4, 22.3 Hz), 127.63, 125.38, 124.66 (dd, J = 44.6, 20.9 Hz), 72.82, 70.14, 34.63, 31.49, 27.88, 20.62, 14.39.

^{2}H NMR (61 MHz, Chloroform) δ 5.50, 5.40.

HRMS (EI) calcd for $C_{17}H_{24}D_2O$ $[M]^+$ 248.2104, found 248.2108.

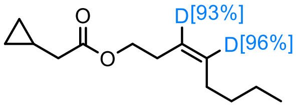

(Z)-oct-3-en-1-yl-3,4-d_2 2-cyclopropylacetate (**3**)

Following **standard condition**, **3** was obtained as colorless oil (30.4 mg, 72% yield, Z:E > 20:1, 96% D, 93% D).

^{1}H NMR (500 MHz, Chloroform-d) δ 5.48 (t, J = 7.3 Hz, 0.04H), 5.33 (t, J = 7.3 Hz, 0.07H), 4.07 (t, J = 6.9 Hz, 2H), 2.36 (t, J = 6.9 Hz, 2H), 2.19 (d, J = 7.2 Hz, 2H), 2.10 – 1.98 (m, 2H), 1.38 – 1.26 (m, 4H), 1.10 – 0.97 (m, 1H), 0.96 – 0.82 (m, 3H), 0.62 – 0.48 (m, 2H), 0.23 – 0.10 (m, 2H).

^{13}C NMR (126 MHz, Chloroform-d) δ 173.44, 132.57 (dd, J = 45.1, 22.1 Hz), 124.07 (dd, J = 44.8, 20.9 Hz), 63.99, 39.53, 31.87, 26.99, 26.84, 22.44, 14.10, 7.00, 4.47.

^{2}H NMR (92 MHz, Chloroform) δ 5.48, 5.33.

HRMS (ESI) calcd for $C_{13}H_{21}O_2D_2$ $[M+H]^+$ 213.1818, found 213.1817.

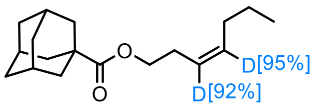

(Z)-hept-3-en-1-yl-3,4-d_2 (3r,5r,7r)-adamantane-1-carboxylate (**4**)

Following **standard condition**, **4** was obtained as colorless oil (49.0 mg, 88% yield, Z:E > 20:1, 95% D, 92% D).

<u>^{1}H NMR</u> (500 MHz, Chloroform-d) δ 5.47 (t, J = 7.1 Hz, 0.05H), 5.34 (t, J = 7.2 Hz, 0.08H), 4.03 (t, J = 6.9 Hz, 2H), 2.35 (t, J = 6.8 Hz, 2H), 2.01 (q, J = 6.1, 4.9 Hz, 5H), 1.91 – 1.82 (m, 6H), 1.75 – 1.65 (m, 6H), 1.37 (h, J = 7.3 Hz, 2H), 0.90 (t, J = 7.4 Hz, 3H).

<u>^{13}C NMR</u> (126 MHz, Chloroform-d) δ 177.89, 132.13 (dd, J = 45.5, 22.1 Hz), 124.42 (dd, J = 44.8, 20.9 Hz), 63.71, 40.78, 38.93, 36.62, 29.32, 28.06, 26.87, 22.84, 13.89.

<u>^{2}H NMR</u> (92 MHz, Chloroform) δ 5.47, 5.34.

<u>HRMS (ESI)</u> calcd for $C_{18}H_{27}O_2D_2$ $[M+H]^+$ 279.2288, found 279.2286.

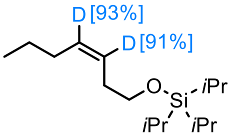

(Z)-((hept-3-en-1-yl-3,4-d_2)oxy)triisopropylsilane (**5**)

Following **standard condition**, **5** was obtained as colorless oil (52.1 mg, 96% yield, Z:E > 20:1, 93% D, 91% D).

<u>^{1}H NMR</u> (500 MHz, Chloroform-d) δ 5.45 (tt, J = 7.3, 1.6 Hz, 0.07H), 5.40 (tt, J = 7.3, 1.6 Hz, 0.09H), 3.67 (t, J = 7.2 Hz, 2H), 2.30 (t, J = 7.2 Hz, 2H), 2.02 (t, J = 7.4 Hz, 2H), 1.42 – 1.32 (m, 2H), 1.13 – 1.03 (m, 21H), 0.90 (t, J = 7.4 Hz, 3H).

<u>^{13}C NMR</u> (126 MHz, Chloroform-d) δ 131.30 (dd, J = 45.3, 21.8 Hz), 125.53 (dd, J = 44.4, 20.9 Hz), 63.41, 31.30, 29.42, 22.99, 18.16, 13.93, 12.14.

<u>^{2}H NMR</u> (92 MHz, Chloroform) δ 5.45, 5.40.

<u>HRMS (ESI)</u> calcd for $C_{14}H_{32}D_2OSi$ $[M-C_3H_7]^+$ 229.1951, found 229.1951.

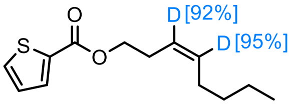

(Z)-oct-3-en-1-yl-3,4-d_2 thiophene-2-carboxylate (**6**)

Following **standard condition**, **6** was obtained as colorless oil (44.7 mg, 93% yield, Z:E > 20:1, 95% D, 92% D).

<u>^{1}H NMR</u> (500 MHz, Chloroform-d) δ 7.79 (dd, J = 3.7, 1.4 Hz, 1H), 7.54 (dd, J = 5.0,

1.4 Hz, 1H), 7.08 (dd, J = 5.1, 3.6 Hz, 1H), 5.52 (t, J = 7.3 Hz, 0.05H), 5.40 (t, J = 7.4 Hz, 0.08H), 4.28 (t, J = 6.9 Hz, 2H), 2.49 (t, J = 6.9 Hz, 2H), 2.06 (t, J = 7.1 Hz, 2H), 1.37 – 1.27 (m, 4H), 0.89 – 0.86 (m, 3H).

^{13}C NMR (126 MHz, Chloroform-*d*) δ 162.37, 134.03, 133.43, 132.84 (dd, J = 45.5, 22.1 Hz), 123.79 (dd, J = 44.6, 20.6 Hz), 64.70, 31.85, 27.02, 26.90, 22.44, 14.09.

^{2}H NMR (92 MHz, Chloroform) δ 5.52, 5.40.

HRMS (ESI) calcd for $C_{13}H_{17}O_2D_2S$ $[M+H]^+$ 241.1226, found 241.1223.

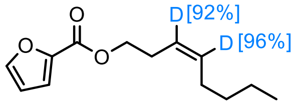

(*Z*)-oct-3-en-1-yl-3,4-*d*$_2$ furan-2-carboxylate (**7**)

Following **standard condition**, **7** was obtained as colorless oil (42.4 mg, 95% yield, *Z:E* > 20:1, 96% D, 92% D).

^{1}H NMR (500 MHz, Chloroform-*d*) δ 7.66 – 7.44 (m, 1H), 7.19 – 7.04 (m, 1H), 6.48 (dd, J = 3.5, 1.8 Hz, 1H), 5.50 (t, J = 7.3 Hz, 0.04H), 5.37 (t, J = 7.5 Hz, 0.08H), 4.27 (t, J = 7.0 Hz, 2H), 2.47 (t, J = 7.0 Hz, 2H), 2.04 (t, J = 7.1 Hz, 2H), 1.37 – 1.24 (m, 4H), 0.95 – 0.80 (m, 3H).

^{13}C NMR (126 MHz, Chloroform-*d*) δ 158.83, 146.31, 144.84, 132.94 (dd, J = 45.3, 21.8 Hz), 123.59 (dd, J = 44.4, 20.9 Hz), 117.89, 111.88, 64.46, 31.80, 26.97, 26.87, 22.39, 14.05.

^{2}H NMR (92 MHz, Chloroform) δ 5.50, 5.36.

HRMS (ESI) calcd for $C_{13}H_{17}O_3D_2$ $[M+H]^+$ 225.1454, found 225.1450.

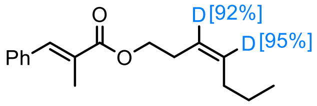

(*Z*)-hept-3-en-1-yl-3,4-*d*$_2$ (*E*)-2-methyl-3-phenylacrylate (**8**)

Following **standard condition**, **8** was obtained as yellow oil (50.1 mg, 96% yield, *Z:E* > 20:1, 95% D, 92% D).

^{1}H NMR (500 MHz, Chloroform-*d*) δ 7.73 – 7.67 (m, 1H), 7.43 – 7.37 (m, 4H), 7.35 – 7.30 (m, 1H), 5.53 (t, J = 7.4 Hz, 0.05H), 5.43 (t, J = 7.4 Hz, 0.08H), 4.22 (t, J = 6.9 Hz, 2H), 2.48 (t, J = 6.9 Hz, 2H), 2.12 (d, J = 1.5 Hz, 3H), 2.06 (t, J = 7.5 Hz, 2H), 1.40 (h, J = 7.3 Hz, 2H), 0.92 (t, J = 7.4 Hz, 3H).

^{13}C NMR (126 MHz, Chloroform-*d*) δ 168.80, 138.89, 136.03, 132.40 (dd, J = 45.3, 21.8 Hz), 129.74, 128.62, 128.46, 128.37, 124.35 (dd, J = 44.4, 20.9 Hz), 64.55, 29.33, 26.93, 22.84, 14.17, 13.90.

^{2}H NMR (92 MHz, Chloroform) δ 5.53, 5.43.

HRMS (ESI) calcd for $C_{17}H_{21}O_2D_2$ $[M+H]^+$ 261.1818, found 261.1817.

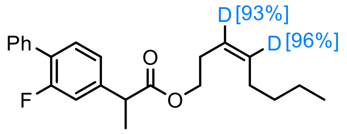

(*Z*)-oct-3-en-1-yl-3,4-*d*$_2$ 2-(2-fluoro-[1,1'-biphenyl]-4-yl)propanoate (**9**)

Following **standard condition**, **9** was obtained as white solid (51.2 mg, 72% yield, *Z:E* > 20:1, 96% D, 93% D).

^{1}H NMR (500 MHz, Chloroform-*d*) δ 7.56 (d, *J* = 7.8 Hz, 2H), 7.45 (t, *J* = 7.6 Hz, 3H), 7.39 (p, *J* = 7.6 Hz, 2H), 7.20 – 7.12 (m, 2H), 5.50 (t, *J* = 7.6 Hz, 0.04H), 5.31 (t, *J* = 7.9 Hz, 0.07H), 4.18 – 4.07 (m, 2H), 3.76 (q, *J* = 7.2 Hz, 1H), 2.38 (t, *J* = 6.9 Hz, 2H), 2.02 (t, *J* = 6.9 Hz, 2H), 1.55 (d, *J* = 7.2 Hz, 3H), 1.39 – 1.30 (m, 4H), 0.95 – 0.88 (m, 3H).

^{13}C NMR (126 MHz, Chloroform-*d*) δ 174.11, 159.76 (d, *J* = 248.6 Hz), 141.99 (d, *J* = 7.8 Hz), 135.62, 132.71 (dd, *J* = 45.2, 22.2 Hz), 130.85 (d, *J* = 3.7 Hz), 129.05 (d, *J* = 2.8 Hz), 128.55, 127.85 (d, *J* = 13.3 Hz), 127.75, 123.82 (dd, *J* = 44.6, 21.0 Hz), 123.67 (d, *J* = 3.2 Hz), 115.38 (d, *J* = 23.4 Hz), 64.61, 45.17, 31.84, 26.97, 26.74, 22.43, 18.53, 14.09.

^{19}F NMR (471 MHz, Chloroform-*d*) δ -117.56.

^{2}H NMR (92 MHz, Chloroform) δ 5.50, 5.31.

HRMS (ESI) calcd for $C_{23}H_{26}O_2FD_2$ $[M+H]^+$ 357.2193, found 357.2194.

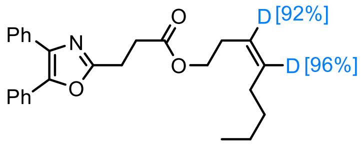

(*Z*)-oct-3-en-1-yl-3,4-*d*$_2$ 3-(4,5-diphenyloxazol-2-yl)propanoate (**10**)

Following **standard condition**, **10** was obtained as white solid (76.8 mg, 95% yield, *Z:E* > 20:1, 96% D, 92% D).

^{1}H NMR (500 MHz, Chloroform-*d*) δ 7.67 – 7.61 (m, 2H), 7.61 – 7.55 (m, 2H), 7.40 – 7.28 (m, 6H), 5.49 (t, *J* = 7.3 Hz, 0.04H), 5.34 (t, *J* = 7.4 Hz, 0.08H), 4.13 (t, *J* = 6.9 Hz, 2H), 3.19 (dd, *J* = 8.3, 6.8 Hz, 2H), 2.92 (dd, *J* = 8.3, 6.8 Hz, 2H), 2.39 (t, *J* = 6.9 Hz, 2H), 2.03 (t, *J* = 7.1 Hz, 2H), 1.37 – 1.28 (m, 4H), 0.95 – 0.86 (m, 3H).

^{13}C NMR (126 MHz, Chloroform-*d*) δ 172.07, 161.84, 145.44, 135.13, 132.64 (dd, *J* = 45.3, 22.3 Hz), 132.46, 129.00, 128.68, 128.60, 128.49, 128.11, 127.94, 126.49, 123.79

(dd, J = 44.6, 20.6 Hz), 64.40, 31.78, 31.18, 26.92, 26.70, 23.59, 22.36, 14.06.

^{2}H NMR (92 MHz, Chloroform) δ 5.49, 5.34.

HRMS (ESI) calcd for $C_{26}H_{27}O_3ND_2Na$ $[M+Na]^+$ 428.2165, found 428.2156.

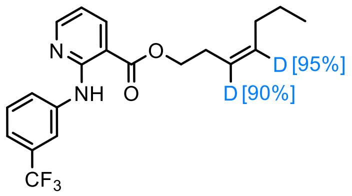

(Z)-hept-3-en-1-yl-3,4-d_2 2-((3-(trifluoromethyl)phenyl)amino)nicotinate (**11**)

Following **standard condition**, **11** was obtained as yellow oil (50.3 mg, 66% yield, Z:E > 20:1, 95% D, 90% D).

^{1}H NMR (500 MHz, Chloroform-d) δ 10.39 (s, 1H), 8.40 (dd, J = 4.7, 2.0 Hz, 1H), 8.26 (dd, J = 7.8, 2.0 Hz, 1H), 8.10 (t, J = 2.1 Hz, 1H), 7.95 – 7.80 (m, 1H), 7.43 (t, J = 7.9 Hz, 1H), 7.36 – 7.20 (m, 1H), 6.78 (dd, J = 7.8, 4.7 Hz, 1H), 5.56 (t, J = 7.3 Hz, 0.05H), 5.45 (t, J = 7.2 Hz, 0.10H), 4.34 (t, J = 6.9 Hz, 2H), 2.54 (t, J = 6.8 Hz, 2H), 2.07 (t, J = 7.3 Hz, 2H), 1.40 (h, J = 7.3 Hz, 2H), 0.92 (t, J = 7.4 Hz, 3H).

^{13}C NMR (126 MHz, Chloroform-d) δ 167.56, 155.83, 153.05, 140.46, 140.35, 132.90 (dd, J = 45.5, 22.1 Hz), 131.21 (q, J = 31.9 Hz), 129.30, 124.32 (q, J = 272.5 Hz), 123.85 (dd, J = 46.0, 22.6 Hz), 123.56, 119.06 (q, J = 3.7 Hz), 117.17 (q, J = 4.1 Hz), 114.14, 107.74, 65.01, 29.37, 26.81, 22.80, 13.87.

^{19}F NMR (471 MHz, Chloroform-d) δ -62.58.

^{2}H NMR (92 MHz, Chloroform) δ 5.57, 5.45.

HRMS (ESI) calcd for $C_{20}H_{20}O_2N_2F_3D_2$ $[M+H]^+$ 381.1753, found 381.1746.

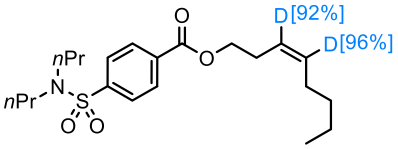

(Z)-oct-3-en-1-yl-3,4-d_2 4-(N,N-dipropylsulfamoyl)benzoate (**12**)

Following **standard condition**, **12** was obtained as white solid (61.8 mg, 78% yield, Z:E > 20:1, 96% D, 92% D).

^{1}H NMR (500 MHz, Chloroform-d) δ 8.12 (d, J = 8.4 Hz, 2H), 7.84 (d, J = 8.4 Hz, 2H), 5.50 (t, J = 7.6 Hz, 0.04H), 5.39 (t, J = 7.3 Hz, 0.08H), 4.32 (t, J = 6.9 Hz, 2H), 3.06 (dd, J = 8.9, 6.6 Hz, 4H), 2.50 (t, J = 6.9 Hz, 2H), 2.43 (t, J = 6.7 Hz, 0.16H), 2.03 (t, J = 7.0 Hz, 2H), 1.97 (t, J = 6.9 Hz, 0.16H), 1.57 – 1.45 (m, 4H), 1.34 – 1.23 (m, J = 3.4 Hz, 4H), 0.90 – 0.78 (m, 9H).

^{13}C NMR (126 MHz, Chloroform-*d*) δ 165.26, 144.14, 133.70, 132.88 (dd, *J* = 45.0, 22.1 Hz), 130.22, 126.98, 123.67 (dd, *J* = 44.8, 20.9 Hz), 65.12, 49.94, 31.75, 26.94, 26.75, 22.34, 21.95, 14.01, 11.18.

^{2}H NMR (92 MHz, Chloroform) δ 5.50, 5.38.

HRMS (ESI) calcd for $C_{21}H_{32}O_4NSD_2$ $[M+H]^+$ 420.2148, found 420.2141.

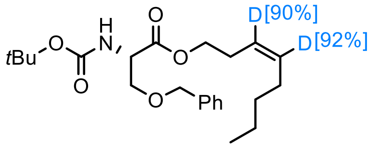

(*Z*)-oct-3-en-1-yl-3,4-d_2 *O*-benzyl-*N*-(tert-butoxycarbonyl)-*D*-serinate (**13**)

Following **standard condition**, **13** was obtained as white solid (76.6 mg, 94% yield, *Z:E* > 20:1, 92% D, 90% D).

^{1}H NMR (500 MHz, Chloroform-*d*) δ 7.37 – 7.24 (m, 5H), 5.47 (t, *J* = 7.2 Hz, 0.08H), 5.42 (d, *J* = 8.9 Hz, 1H), 5.29 (t, *J* = 7.9 Hz, 0.10H), 4.58 – 4.45 (m, 2H), 4.43 (dt, *J* = 8.9, 3.2 Hz, 1H), 4.12 (td, *J* = 7.1, 1.6 Hz, 2H), 3.86 (dd, *J* = 9.5, 3.2 Hz, 1H), 3.68 (dd, *J* = 9.4, 3.3 Hz, 1H), 2.35 (t, *J* = 6.9 Hz, 2H), 2.07 – 1.96 (m, 2H), 1.45 (s, 9H), 1.36 – 1.28 (m, 4H), 0.93 – 0.85 (m, 3H).

^{13}C NMR (126 MHz, Chloroform-*d*) δ 170.75, 155.56, 137.65, 132.72 (dd, *J* = 44.8, 21.8 Hz), 128.46, 127.85, 127.65, 123.56 (dd, *J* = 44.4, 20.9 Hz), 79.95, 73.29, 70.13, 65.07, 54.11, 31.77, 28.38, 26.94, 26.60, 22.37, 14.06.

^{2}H NMR (92 MHz, Chloroform) δ 5.46, 5.29.

HRMS (ESI) calcd for $C_{23}H_{34}O_5ND_2$ $[M+H]^+$ 430.2533, found 430.2527.

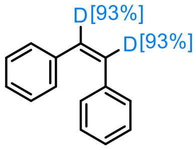

(*Z*)-1,2-diphenylethene-1,2-d_2 (**14**)

Following **standard condition**, **14** was obtained as white solid (29.2 mg, 80% yield, *Z:E* > 20:1, 93% D, 93% D).

^{1}H NMR (500 MHz, Chloroform-*d*) δ 7.32 – 7.15 (m, 10H), 6.59 (s, 0.14H).

^{13}C NMR (126 MHz, Chloroform-*d*) δ 137.25, 129.97 (dd, *J* = 46.0, 22.0 Hz), 128.98, 128.33, 127.22.

^{2}H NMR (92 MHz, Chloroform) δ 6.59.

HRMS (EI) calcd for $C_{14}H_{10}D_2$ $[M]^+$ 182.1060, found 182.1059.

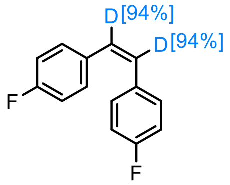

(*Z*)-1,2-bis(4-fluorophenyl)ethene-1,2-d_2 (**15**)

Following **standard condition**, **15** was obtained as white solid (26.2 mg, 60% yield, *Z:E* > 20:1, 94% D, 94% D).

^{1}H NMR (500 MHz, Chloroform-*d*) δ 7.23 – 7.16 (m, 4H), 6.98 – 6.90 (m, 4H), 6.55 (s, 0.12H).

^{13}C NMR (126 MHz, Chloroform-*d*) δ 162.00 (d, J = 246.8 Hz), 133.02 (d, J = 2.4 Hz), 130.61 (d, J = 7.8 Hz), 128.84 (dd, J = 45.6, 21.6 Hz), 115.40 (d, J = 21.2 Hz).

^{2}H NMR (61 MHz, Chloroform) δ 6.55.

^{19}F NMR (471 MHz, Chloroform-*d*) δ -114.32.

HRMS (EI) calcd for $C_{14}H_8F_2D_2$ $[M]^+$ 218.0871, found 218.0871.

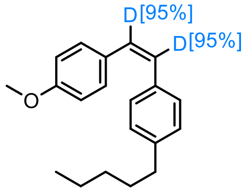

(*Z*)-1-methoxy-4-(2-(4-pentylphenyl)vinyl-1,2-d_2)benzene (**16**)

Following **standard condition**, **16** was obtained as white solid (23.6 mg, 42% yield, *Z:E* > 20:1, 95% D, 95% D).

^{1}H NMR (400 MHz, Chloroform-*d*) δ 7.27 – 7.18 (m, 4H), 7.07 (d, J = 8.1 Hz, 2H), 6.83 – 6.74 (m, 2H), 6.50 (s, 0.10H), 3.81 (s, 3H), 2.63 – 2.55 (m, 2H), 1.63 (p, J = 7.6 Hz, 1H), 1.42 – 1.29 (m, 5H), 0.92 (t, J = 6.9 Hz, 3H).

^{13}C NMR (101 MHz, Chloroform-*d*) δ 158.73, 141.90, 134.87, 130.22, 130.01, 129.02 (d, J = 11.5 Hz), 128.82, 128.60 (dd, J = 23.4, 2.7 Hz), 128.37, 113.70, 55.30, 35.82, 31.69, 31.17, 22.68, 14.18.

^{2}H NMR (61 MHz, Chloroform) δ 6.50.

HRMS (EI) calcd for $C_{20}H_{22}D_2O$ $[M]^+$ 282.1948, found 282.1947.

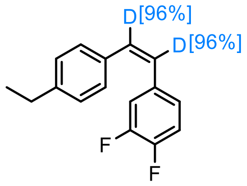

(*Z*)-4-(2-(4-ethylphenyl)vinyl-1,2-*d*2)-1,2-difluorobenzene (**17**)

Following **standard condition**, **17** was obtained as white solid (47.6 mg, 97% yield, *Z:E* > 20:1, 96% D, 96% D).

^{1}H NMR (500 MHz, Chloroform-*d*) δ 7.19 – 6.93 (m, 7H), 6.61 (s, 0.04H), 6.44 (s, 0.04H), 2.64 (q, *J* = 7.6 Hz, 2H), 1.24 (t, *J* = 7.6 Hz, 3H).

^{13}C NMR (126 MHz, Chloroform-*d*) δ 150.81 (dd, *J* = 93.5, 12.6 Hz), 148.84 (dd, *J* = 94.7, 12.9 Hz), 143.89, 134.51 (dd, *J* = 5.2, 5.2 Hz), 133.80, 131.11 (dd, *J* = 46.0, 22.1 Hz), 128.88, 128.04, 127.09 (dd, *J* = 44.8, 21.4 Hz), 125.25 (dd, *J* = 6.2, 3.4 Hz), 117.64 (d, *J* = 17.0 Hz), 117.11 (d, *J* = 17.0 Hz), 28.73, 15.52.

^{2}H NMR (92 MHz, Chloroform) δ 6.60, 6.44.

^{19}F NMR (471 MHz, Chloroform-*d*) δ -138.23 (d, *J* = 20.8 Hz), -139.62 (d, *J* = 21.7 Hz).

HRMS (EI) calcd for $C_{16}H_{12}F_2D_2$ $[M]^+$ 246.1184, found 246.1186.

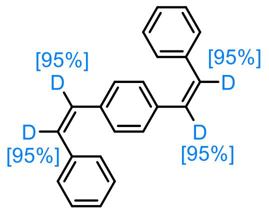

1,4-bis((*Z*)-2-phenylvinyl-1,2-*d*2)benzene (**18**)

Following **standard condition**, **18** was obtained as white solid (20.4 mg, 71% yield, *Z:E* > 20:1, 95% D, 95% D, 95%D, 95%D).

^{1}H NMR (500 MHz, Chloroform-*d*) δ 7.29 – 7.16 (m, 10H), 7.13 – 7.09 (m, 4H), 6.55 (d, *J* = 17.5 Hz, 0.20H).

^{13}C NMR (126 MHz, Chloroform-*d*) δ 137.36, 136.11, 130.04 (dd, *J* = 42.1, 20.0 Hz), 129.68 (dd, *J* = 23.9, 18.7 Hz), 128.98, 128.87, 128.33, 127.27.

^{2}H NMR (61 MHz, Chloroform) δ 6.60.

HRMS (EI) calcd for $C_{22}H_{14}D_4$ $[M]^+$ 286.1655, found 286.1655.

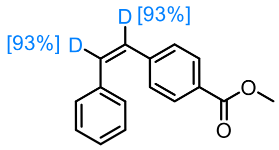

methyl (*Z*)-4-(2-phenylvinyl-1,2-*d*2)benzoate (**19**)

Following **standard condition**, **19** was obtained as yellow solid (39.0 mg, 81% yield, *Z:E* > 20:1, 93% D, 93% D).

^{1}H NMR (400 MHz, Chloroform-*d*) δ 7.90 (d, *J* = 8.4 Hz, 1H), 7.31 (d, *J* = 8.4 Hz, 1H), 7.23 (s, 3H), 6.71 (s, 0.07H), 6.61 (s, 0.07H), 3.90 (s, 2H).
^{13}C NMR (126 MHz, Chloroform-*d*) δ 167.04, 142.15, 136.71, 131.97 (dd, *J* = 48.0, 21.2 Hz), 131.63, 129.65, 128.97, 128.95 (dd, *J* = 44.7, 21.8 Hz), 128.71, 128.47, 127.65, 52.16.
^{2}H NMR (92 MHz, Chloroform) δ 6.71, 6.60.
HRMS (EI) calcd for $C_{16}H_{12}D_2O_2$ $[M]^+$ 240.1114, found 240.1114.

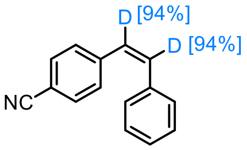

(*Z*)-4-(2-phenylvinyl-1,2-*d*$_2$)benzonitrile[19] (**20**)
Following **standard condition**, **20** was obtained as yellow solid (30.7 mg, 74% yield, *Z:E* > 20:1, 94% D, 94% D).
^{1}H NMR (500 MHz, Chloroform-*d*) δ 7.50 – 7.44 (m, 2H), 7.33 – 7.28 (m, 2H), 7.27 – 7.21 (m, 3H), 7.21 – 7.15 (m, 2H), 6.75 (s, 0.06H), 6.55 (s, 0.06H).
^{13}C NMR (126 MHz, Chloroform-*d*) δ 142.11, 136.26, 133.05 (dd, *J* = 46.6, 22.8 Hz), 132.12, 129.64, 128.88, 128.62, 128.10 (dd, *J* = 45.1, 21.1 Hz), 127.95, 119.06, 110.58.
^{2}H NMR (92 MHz, Chloroform) δ 6.74, 6.55.

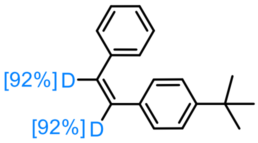

(*Z*)-1-(tert-butyl)-4-(2-phenylvinyl-1,2-*d*$_2$)benzene (**21**)
Following **standard condition**, **21** was obtained as yellow oil (40.0 mg, 84% yield, *Z:E* > 20:1, 92% D, 92% D).
^{1}H NMR (500 MHz, Chloroform-*d*) δ 7.32 – 7.27 (m, 2H), 7.24 – 7.16 (m, 7H), 6.56 – 6.51 (m, 0.16H), 1.29 (s, 9H).
^{13}C NMR (126 MHz, Chloroform-*d*) δ 150.29, 137.65, 134.24, 129.84 (dd, *J* = 45.8, 22.4 Hz), 129.32 (dd, *J* = 45.5, 22.3 Hz), 128.95, 128.72, 128.35, 127.11, 125.21, 34.68, 31.42.
^{2}H NMR (92 MHz, Chloroform) δ 6.54.
HRMS (EI) calcd for $C_{18}H_{18}D_2$ $[M]^+$ 238.1685, found 238.1687.

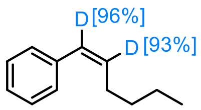

(*Z*)-(hex-1-en-1-yl-1,2-d_2)benzene (**22**)

Following **standard condition**, **22** was obtained as colorless oil (31.0 mg, 96% yield, *Z*:*E* > 20:1, 96% D, 93% D).

^{1}H NMR (500 MHz, Chloroform-*d*) δ 7.38 – 7.28 (m, 4H), 7.25 – 7.20 (m, 1H), 6.42 (s, 0.04H), 5.69 (t, *J* = 7.2 Hz, 0.07H), 2.35 (t, *J* = 7.4 Hz, 1.88H), 2.23 (t, *J* = 7.3 Hz, 0.12H), 1.52 – 1.42 (m, 2H), 1.38 (h, *J* = 6.9 Hz, 2H), 0.92 (t, *J* = 7.2 Hz, 3H).

^{13}C NMR (151 MHz, Chloroform-*d*) δ 137.94, 132.95 (dd, *J* = 51.3, 28.2 Hz), 128.90, 128.48 (dd, *J* = 31.1, 6.5 Hz), 128.23, 126.54, 32.29, 28.36, 22.57, 14.10.

^{2}H NMR (61 MHz, Chloroform) δ 6.42, 5.68.

HRMS (EI) calcd for $C_{12}H_{14}D_2$ $[M]^+$ 162.1373, found 162.1371.

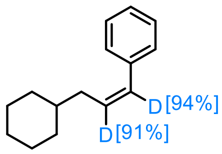

(*Z*)-(3-cyclohexylprop-1-en-1-yl-1,2-d_2)benzene (**23**)

Following **standard condition**, **23** was obtained as colorless oil (31.6 mg, 78% yield, *Z*:*E* > 20:1, 94% D, 91% D).

^{1}H NMR (500 MHz, Chloroform-*d*) δ 7.35 – 7.24 (m, 4H), 7.22 – 7.17 (m, 1H), 6.42 (s, 0.06H), 5.68 (t, *J* = 7.3 Hz, 0.09H), 2.21 (d, *J* = 6.8 Hz, 2H), 1.75 (dd, *J* = 13.1, 3.5 Hz, 2H), 1.70 – 1.65 (m, 2H), 1.45 – 1.32 (m, 1H), 1.26 – 1.03 (m, 4H), 0.99 – 0.82 (m, 2H).

^{13}C NMR (126 MHz, Chloroform-*d*) δ 137.96, 131.62 (dd, *J* = 46.7, 23.3 Hz), 128.93, 128.91 (dd, *J* = 64.2, 17.4 Hz), 128.19, 126.48, 38.82, 36.27, 33.37, 26.68, 26.52.

^{2}H NMR (92 MHz, Chloroform) δ 6.41, 5.68.

HRMS (EI) calcd for $C_{15}H_{18}D_2$ $[M]^+$ 202.1685, found 202.1685.

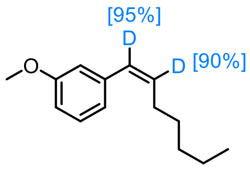

(*Z*)-1-(hept-1-en-1-yl-1,2-d_2)-3-methoxybenzene (**24**)

Following **standard condition**, **24** was obtained as colorless oil (37.2 mg, 90% yield, *Z*:*E* > 20:1, 95% D, 90% D).

^{1}H NMR (500 MHz, Chloroform-*d*) δ 7.24 (t, *J* = 7.9 Hz, 1H), 6.88 (dt, *J* = 7.6, 1.3 Hz, 1H), 6.84 – 6.81 (m, 1H), 6.77 (ddd, *J* = 8.2, 2.6, 0.9 Hz, 1H), 6.37 (s, 0.05H), 5.66 (t, *J* = 7.2 Hz, 0.10H), 3.80 (s, 3H), 2.32 (t, *J* = 7.6 Hz, 2H), 1.55 – 1.41 (m, 2H), 1.35 – 1.27 (m, 4H), 0.92 – 0.84 (m, 3H).

^{13}C NMR (126 MHz, Chloroform-*d*) δ 159.50, 139.28, 133.30 (dd, *J* = 46.6, 23.4 Hz), 129.17, 128.32 (dd, *J* = 44.4, 20.8 Hz), 121.46, 114.43, 112.06, 55.27, 31.73, 29.78, 28.71, 22.70, 14.19.

^{2}H NMR (92 MHz, Chloroform) δ6.37, 5.66.

HRMS (EI) calcd for $C_{14}H_{18}D_2O$ [M]$^+$ 206.1634.1373, found 206.1634.

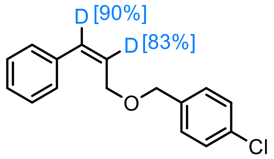

(*Z*)-1-chloro-4-(((3-phenylallyl-2,3-*d*$_2$)oxy)methyl)benzene (**25**)

Following **standard condition**, **25** was obtained as colorless oil (49.5 mg, 95% yield, *Z*:*E* > 20:1, 90% D, 83% D).

^{1}H NMR (500 MHz, Chloroform-*d*) δ 7.39 – 7.26 (m, 7H), 7.25 – 7.20 (m, 2H), 6.66 (s, 0.10H), 5.92 (t, *J* = 6.6 Hz, 0.17H), 4.50 (s, 2H), 4.32 (s, 2H).

^{13}C NMR (126 MHz, Chloroform-*d*) δ 136.81, 136.60, 133.48, 131.82 (dd, *J* = 45.0, 21.6 Hz), 129.24, 128.87, 128.63, 128.38 (dd, *J* = 49.2, 25.0 Hz), 128.36, 127.36, 71.71, 66.97.

^{2}H NMR (61 MHz, Chloroform) δ 6.66, 5.92.

HRMS (APCI) calcd for $C_{16}H_{13}D_2ClO$ [M+H]$^+$ 261.1010, found 261.1008.

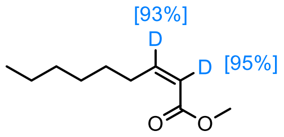

methyl (*Z*)-non-2-enoate-2,3-*d*$_2$ (**26**)

Following **standard condition**, **26** was obtained as colorless oil (18.3 mg, 53% yield, *Z*:*E* > 20:1, 95% D, 93% D).

^{1}H NMR (500 MHz, Chloroform-*d*) δ 6.23 (t, *J* = 7.4 Hz, 0.07H), 5.76 (s, 0.05H), 3.70 (s, 3H), 2.64 (t, *J* = 7.5 Hz, 2H), 1.43 (p, *J* = 7.4, 6.9 Hz, 2H), 1.35 – 1.26 (m, 6H), 0.88 (t, *J* = 6.9 Hz, 3H).

^{13}C NMR (126 MHz, Chloroform-*d*) δ 167.09, 150.90 (dd, *J* = 47.3, 24.3 Hz), 119.07 (dd, *J* = 40.0, 28.0 Hz), 64.27 (d, *J* = 3306.3 Hz), 31.77, 29.12, 29.10, 29.00, 22.73,

14.22.

^{2}H NMR (92 MHz, Chloroform) δ 6.23, 5.76.

HRMS (EI) calcd for $C_{10}H_{16}D_2O_2$ $[M]^+$ 172.1427, found 172.1425.

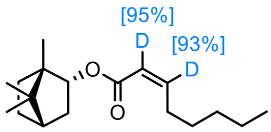

(*1S,2R,4S*)-1,7,7-trimethylbicyclo[2.2.1]heptan-2-yl (*Z*)-oct-2-enoate-2,3-d_2 (**27**)

Following **standard condition**, **27** was obtained as colorless oil (49.9 mg, 89% yield, *Z:E* > 20:1, 95% D, 93% D).

^{1}H NMR (500 MHz, Chloroform-*d*) δ 6.19 (t, J = 7.5 Hz, 0.07H), 5.78 (s, 0.05H), 4.93 (ddd, J = 9.9, 3.6, 2.4 Hz, 1H), 2.68 – 2.59 (m, 2H), 2.44 – 2.33 (m, 1H), 1.96 (ddd, J = 13.3, 9.4, 4.5 Hz, 1H), 1.75 (tq, J = 12.1, 4.3 Hz, 1H), 1.68 (t, J = 4.6 Hz, 1H), 1.49 – 1.39 (m, 2H), 1.37 – 1.25 (m, 6H), 1.00 (dd, J = 13.7, 3.5 Hz, 1H), 0.92 (s, 3H), 0.88 (s, 6H), 0.84 (s, 3H).

^{13}C NMR (151 MHz, Chloroform-*d*) δ 167.19, 149.67 (dd, J = 52.0, 28.9 Hz), 119.95 (dd, J = 47.0, 22.4 Hz), 79.68, 48.88, 47.93, 45.09, 37.08, 31.68, 29.08, 28.90, 28.20, 27.42, 22.63, 19.88, 18.99, 14.13, 13.66.

^{2}H NMR (61 MHz, Chloroform) δ 6.20, 5.78.

HRMS (EI) calcd for $C_{18}H_{28}D_2O_2$ $[M]^+$ 280.2366, found 280.2367.

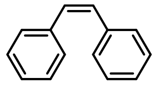

(*Z*)-1,2-diphenylethene (**28**)[20]

Following **semi-hydrogenation condition**, **28** was obtained as colorless oil (29.3 mg, 81% yield,).

^{1}H NMR (500 MHz, Chloroform-*d*) δ 7.30 – 7.23 (m, 7H), 7.23 – 7.18 (m, 3H), 6.62 (s, 2H).

^{13}C NMR (126 MHz, Chloroform-*d*) δ 137.38, 130.38, 129.01, 128.34, 127.22.

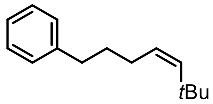

(*Z*)-(6,6-dimethylhept-4-en-1-yl)benzene (**29**)[1]

Following **semi-hydrogenation condition**, **29** was obtained as colorless oil (31.2 mg,

77% yield, *Z:E* > 20:1).

^{1}H NMR (500 MHz, Chloroform-*d*) δ 7.33 – 7.24 (m, 2H), 7.22 – 7.12 (m, 3H), 5.34 (dt, *J* = 12.0, 1.7 Hz, 1H), 5.19 (dt, *J* = 11.9, 7.4 Hz, 1H), 2.68 – 2.59 (m, 2H), 2.22 (qd, *J* = 7.4, 1.7 Hz, 2H), 1.78 – 1.58 (m, 2H), 1.09 (s, 9H).

^{13}C NMR (126 MHz, Chloroform-*d*) δ 142.67, 140.30, 128.56, 128.51, 128.40, 125.80, 35.75, 33.28, 32.17, 31.27, 28.09.

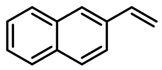

2-vinylnaphthalene (**30**)[21]

Following **semi-hydrogenation condition**, **30** was obtained as colorless solid (23.8 mg, 77% yield).

^{1}H NMR (500 MHz, Chloroform-*d*) δ 7.82 (dt, *J* = 5.5, 2.9 Hz, 2H), 7.77 (d, *J* = 1.7 Hz, 1H), 7.66 (dd, *J* = 8.6, 1.8 Hz, 1H), 7.47 (ddd, *J* = 7.2, 5.0, 1.7 Hz, 2H), 6.90 (dd, *J* = 17.6, 10.9 Hz, 1H), 5.89 (dd, *J* = 17.6, 0.8 Hz, 1H), 5.36 (dd, *J* = 10.8, 0.8 Hz, 1H).

^{13}C NMR (126 MHz, Chloroform-*d*) δ 137.07, 135.15, 133.69, 133.29, 128.29, 128.18, 127.80, 126.51, 126.37, 126.05, 123.31, 114.32.

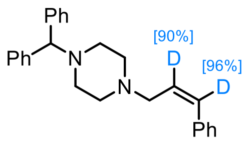

(*Z*)-1-benzhydryl-4-(3-phenylallyl-2,3-*d*$_2$)piperazine (**32**)

Following **standard condition**, **32** was obtained as white solid (41.0 mg, 55% yield, *Z:E* > 20:1, 96% D, 90% D).

^{1}H NMR (500 MHz, Chloroform-*d*) δ 7.45 – 7.41 (m, 4H), 7.39 – 7.34 (m, 2H), 7.31 – 7.25 (m, 7H), 7.21 – 7.16 (m, 2H), 6.60 (s, 0.04H), 5.82 (t, *J* = 6.6 Hz, 0.10H), 4.26 (s, 1H), 3.33 (s, 2H), 2.53 (brs, 8H).

^{13}C NMR (151 MHz, Chloroform-*d*) δ 142.87, 137.17, 131.13 (dd, *J* = 48.1, 25.0 Hz), 129.01 (dd, *J* = 79.5, 30.3 Hz), 129.01, 128.56, 128.24, 128.05, 127.01, 126.97, 76.30, 56.16, 53.61, 51.97.

^{2}H NMR (61 MHz, Chloroform) δ 6.60, 5.82.

HRMS (EI) calcd for $C_{26}H_{26}D_{2}N_{2}$ [M]$^{+}$ 370.2373, found 370.2363.

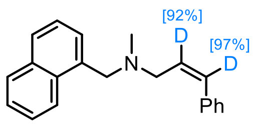

(*Z*)-*N*-methyl-*N*-(naphthalen-1-ylmethyl)-3-phenylprop-2-en-1-amine-2,3-*d*$_2$ (**34**)

Following **standard condition**, **34** was obtained as white solid (35.9 mg, 62% yield, *Z*:*E* > 20:1, 97% D, 92% D).

^{1}H NMR (500 MHz, Chloroform-*d*) δ 8.36 – 8.27 (m, 1H), 7.85 (dd, *J* = 7.9, 1.9 Hz, 1H), 7.77 (dd, *J* = 7.6, 2.1 Hz, 1H), 7.50 (m, 2H), 7.42 – 7.35 (m, 2H), 7.29 – 7.21 (m, 5H), 6.61 (s, 0.03H), 5.94 (t, *J* = 6.6 Hz, 0.08H), 3.91 (s, 2H), 3.38 (s, 2H), 2.25 (s, 3H).

^{13}C NMR (126 MHz, Chloroform-*d*) δ 137.12, 134.74, 133.97, 132.60, 131.26 (dd, *J* = 43.8, 21.0 Hz), 129.81 (dd, *J* = 48.0, 25.5 Hz), 129.09, 128.54, 128.21, 128.10, 127.72, 126.96, 126.01, 125.72, 125.25, 124.80, 60.42, 55.62, 42.76.

^{2}H NMR (92 MHz, Chloroform) δ 6.61, 5.94.

HRMS (EI) calcd for $C_{11}H_{19}D_2N$ [M]$^+$ 289.1795, found 289.1797.

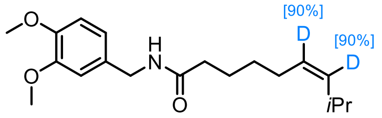

(*Z*)-*N*-(3,4-dimethoxybenzyl)-8-methylnon-6-enamide-6,7-*d*$_2$ (**36**)

Following **standard condition**, **36** was obtained as white solid (51.5 mg, 80% yield, *Z*:*E* > 20:1, 90% D, 90% D).

^{1}H NMR (500 MHz, Chloroform-*d*) δ 6.77 (s, 3H), 5.96 (s, 1H), 5.19 – 5.12 (m, 0.20H), 4.31 (d, *J* = 5.8 Hz, 2H), 3.82 (s, 3H), 3.81 (s, 3H), 2.52 (hept, *J* = 6.6 Hz, 1H), 2.17 (t, *J* = 7.6 Hz, 2H), 2.01 (t, *J* = 7.5 Hz, 2H), 1.63 (p, *J* = 7.6 Hz, 2H), 1.39 – 1.30 (m, 2H), 0.90 (s, 3H), 0.89 (s, 3H).

^{13}C NMR (126 MHz, Chloroform-*d*) δ 172.86, 149.14, 148.42, 137.86, 137.56 (dd, *J* = 42.6, 19.6 Hz), 131.16, 126.35 (dd, *J* = 45.2, 22.2 Hz), 120.07, 111.20, 55.95, 55.87, 43.38, 36.69, 29.58, 26.92, 26.36, 25.48, 23.21.

^{2}H NMR (61 MHz, Chloroform) δ 5.15.

HRMS (EI) calcd for $C_{19}H_{27}D_2NO_3$ [M]$^+$ 321.2268, found 321.2265.

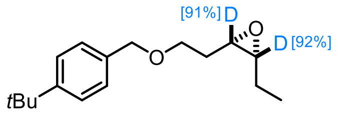

2-(2-((4-(*tert*-butyl)benzyl)oxy)ethyl)-3-ethyloxirane-2,3-*d*$_2$ (**37**)

Following reference[22], **37** was obtained as colorless oil (35.5 mg, 67% yield, 92% D, 91% D).

^{1}H NMR (500 MHz, Chloroform-*d*) δ 7.41 – 7.36 (m, 2H), 7.32 – 7.27 (m, 2H), 4.56 – 4.48 (m, 2H), 3.66 (dd, *J* = 7.2, 5.8 Hz, 2H), 3.10 (dd, *J* = 7.3, 5.0 Hz, 0.09H), 2.91 (t, *J* = 6.4 Hz, 0.08H), 1.91 (dt, *J* = 14.2, 7.2 Hz, 1H), 1.77 (dt, *J* = 14.3, 5.8 Hz, 1H), 1.63

– 1.47 (m, 2H), 1.33 (s, 9H), 1.05 (t, *J* = 7.6 Hz, 3H).

^{13}C NMR (126 MHz, Chloroform-*d*) δ 150.64, 135.32, 127.61, 125.40, 73.03, 67.63, 57.86 (dd, *J* = 50.6, 24.8 Hz), 54.56 (dd, *J* = 49.6, 23.4 Hz), 34.60, 31.44, 28.45, 21.15, 10.60.

^{2}H NMR (92 MHz, Chloroform) δ 3.10, 2.91.

HRMS (APCI) calcd for $C_{17}H_{24}D_2O_2$ $[M+H]^+$ 265.4127, found 265.4126.

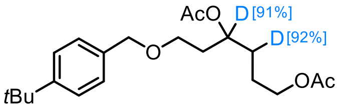

6-((4-(*tert*-butyl)benzyl)oxy)hexane-1,4-diyl-3,4-d_2 diacetate (**38**)

Following reference[23], **38** was obtained as colorless oil (56.4 mg, 77% yield, 92% D, 91% D).

^{1}H NMR (500 MHz, Chloroform-*d*) δ 7.36 (d, *J* = 8.4 Hz, 2H), 7.26 (d, *J* = 8.5 Hz, 2H), 5.07 (q, *J* = 6.3 Hz, 0.09H), 4.44 (d, *J* = 3.0 Hz, 2H), 4.07 – 4.01 (m, 2H), 3.53 – 3.43 (m, 2H), 2.03 (s, 3H), 2.00 (s, 3H), 1.85 (t, *J* = 6.4 Hz, 2H), 1.71 – 1.54 (m, 3.08H), 1.31 (s, 9H).

^{13}C NMR (126 MHz, Chloroform-*d*) δ 171.16, 170.76, 150.63, 135.28, 127.70, 125.37, 72.97, 71.03 (dd, *J* = 45.0, 22.5 Hz), 66.52, 64.21, 34.59, 34.24, 31.43, 30.57 (dd, *J* = 42.0, 23.2 Hz), 24.45, 21.24, 21.04.

^{2}H NMR (92 MHz, Chloroform) δ 5.07, 1.65.

HRMS (APCI) calcd for $C_{21}H_{30}D_2O_5$ $[M+H]^+$ 367.2448, found 367.2449.

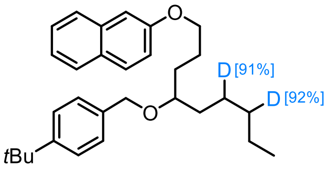

2-((4-((4-(*tert*-butyl)benzyl)oxy)nonyl-6,7-d2)oxy)naphthalene (**39**)

Following reference[24], **39** was obtained as colorless oil (60.8 mg, 70% yield, 92% D, 91% D).

^{1}H NMR (500 MHz, Chloroform-*d*) δ 7.78 – 7.66 (m, 3H), 7.41 (ddd, *J* = 8.2, 6.8, 1.3 Hz, 1H), 7.35 – 7.25 (m, 5H), 7.16 – 7.08 (m, 2H), 4.55 – 4.44 (m, 2H), 4.12 – 3.99 (m, 2H), 3.52 – 3.42 (m, 1H), 2.03 – 1.86 (m, 2H), 1.81 – 1.68 (m, 2H), 1.66 – 1.57 (m, 1H), 1.55 – 1.46 (m, 1H), 1.30 (s, 13.17H), 0.89 (t, *J* = 7.2 Hz, 3H).

^{13}C NMR (126 MHz, Chloroform-*d*) δ 157.13, 150.53, 136.02, 134.72, 129.43, 128.99, 127.79, 127.74, 126.82, 126.39, 125.39, 123.58, 119.14, 106.64, 78.64, 70.76, 68.12,

34.62, 33.79, 31.88 (dd, J = 38.0, 15.8 Hz), 31.48, 30.39, 25.29, 24.71 (dd, J = 37.1, 18.2 Hz), 22.68, 14.22.

^{2}H NMR (61 MHz, Chloroform) δ 1.30.

HRMS (APCI) calcd for $C_{30}H_{38}D_2O_2$ $[M+H]^+$ 435.3227, found 435.3224.

Supplementary Discussions

Control experiments

^{18}O labelling experiments

Under standard condition, we used $H_2{}^{18}O$ and AcOH instead of D_2O and AcOD, and successfully obtained $^{18}OPPh_3$ and **2a**. HRMS results indicate that ^{18}O atoms originate from $H_2{}^{18}O$.

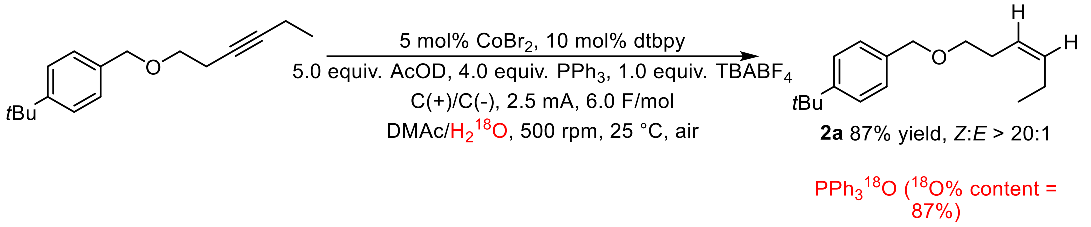

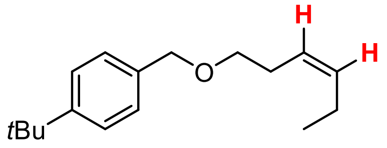

(*Z*)-1-(*tert*-butyl)-4-((hex-3-en-1-yloxy)methyl)benzene (**2a**, 42.8 mg, 87% yield, *Z:E* > 20:1, colorless oil)

^{1}H NMR (500 MHz, Chloroform-*d*) δ 7.43 – 7.36 (m, 2H), 7.32 – 7.27 (m, 2H), 5.54 – 5.43 (m, 1H), 5.42 – 5.33 (m, 1H), 4.50 (s, 2H), 3.49 (t, *J* = 7.1 Hz, 2H), 2.39 (qd, *J* = 7.0, 1.5 Hz, 2H), 2.08 (pd, *J* = 7.5, 1.5 Hz, 2H), 1.33 (s, 9H), 0.98 (t, *J* = 7.6 Hz, 3H).

^{13}C NMR (126 MHz, Chloroform-*d*) δ 150.60, 135.62, 133.75, 127.65, 125.41, 125.04, 72.83, 70.15, 34.65, 31.50, 28.01, 20.78, 14.42.

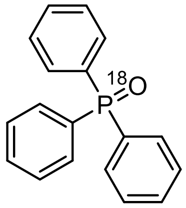

triphenylphosphine oxide-^{18}O

^{1}H NMR (500 MHz, Chloroform-*d*) δ 7.70 – 7.61 (m, 6H), 7.56 – 7.51 (m, 3H), 7.48 – 7.42 (m, 6H).

^{13}C NMR (126 MHz, Chloroform-*d*) δ 133.00, 132.19 (d, *J* = 9.7 Hz), 132.05 (d, *J* = 2.8 Hz), 128.61 (d, *J* = 11.9 Hz).

^{31}P NMR (202 MHz, Chloroform-*d*) δ 29.26.

HRMS (ESI) calcd for $C_{18}H_{15}{}^{18}OP$ $[M+Na]^+$ 303.0795, found 303.0804.

HRMS (ESI) calcd for $C_{18}H_{15}OP$ $[M+Na]^+$ 301.0753, found 301.0760.

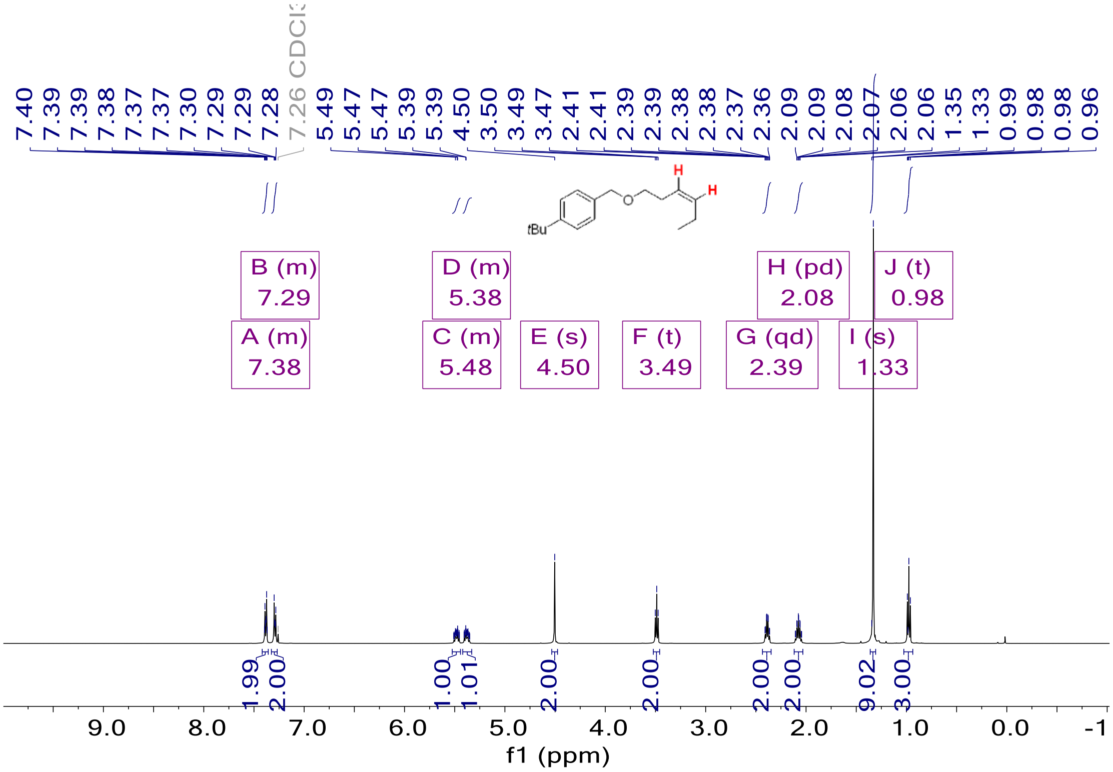

^{1}H-NMR (500 MHz, 298 K, Chloroform-*d*) spectra for **2a**

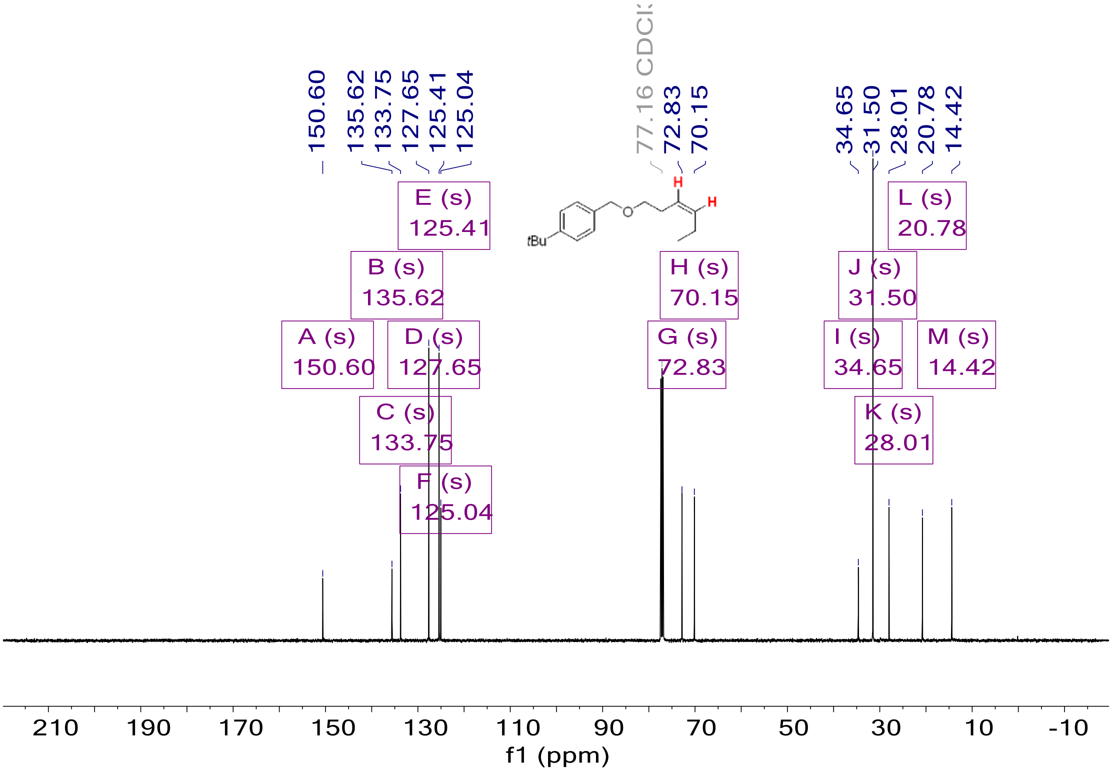

^{13}C-NMR (126 MHz, Chloroform-*d*) spectra for **2a**

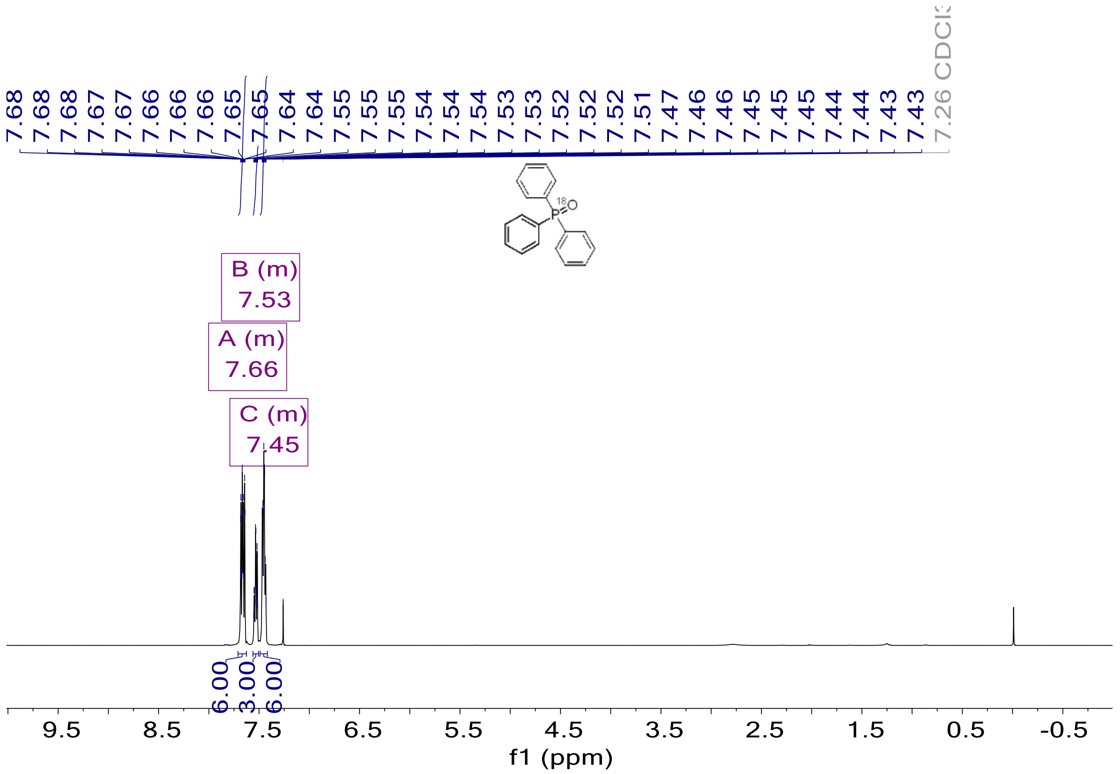

^{1}H-NMR (500 MHz, 298 K, Chloroform-*d*) spectra for **^{18}OPPh$_3$**

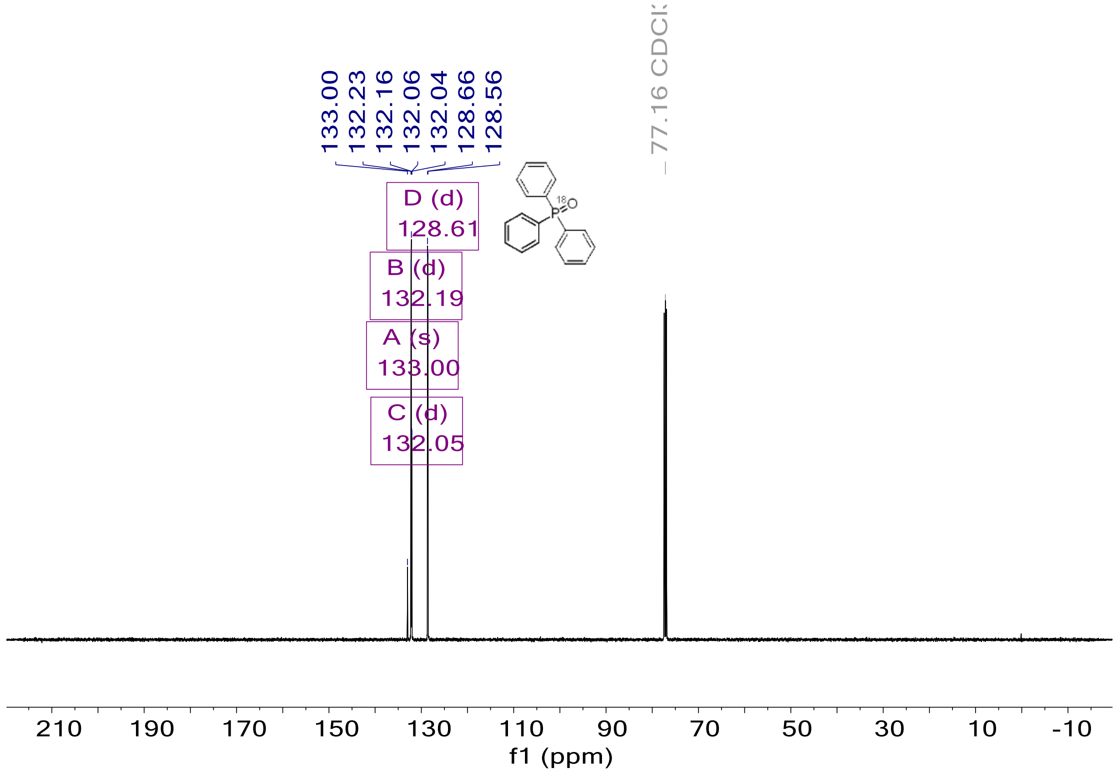

^{13}C-NMR (126 MHz, 298 K, Chloroform-*d*) spectra for **^{18}OPPh$_3$**

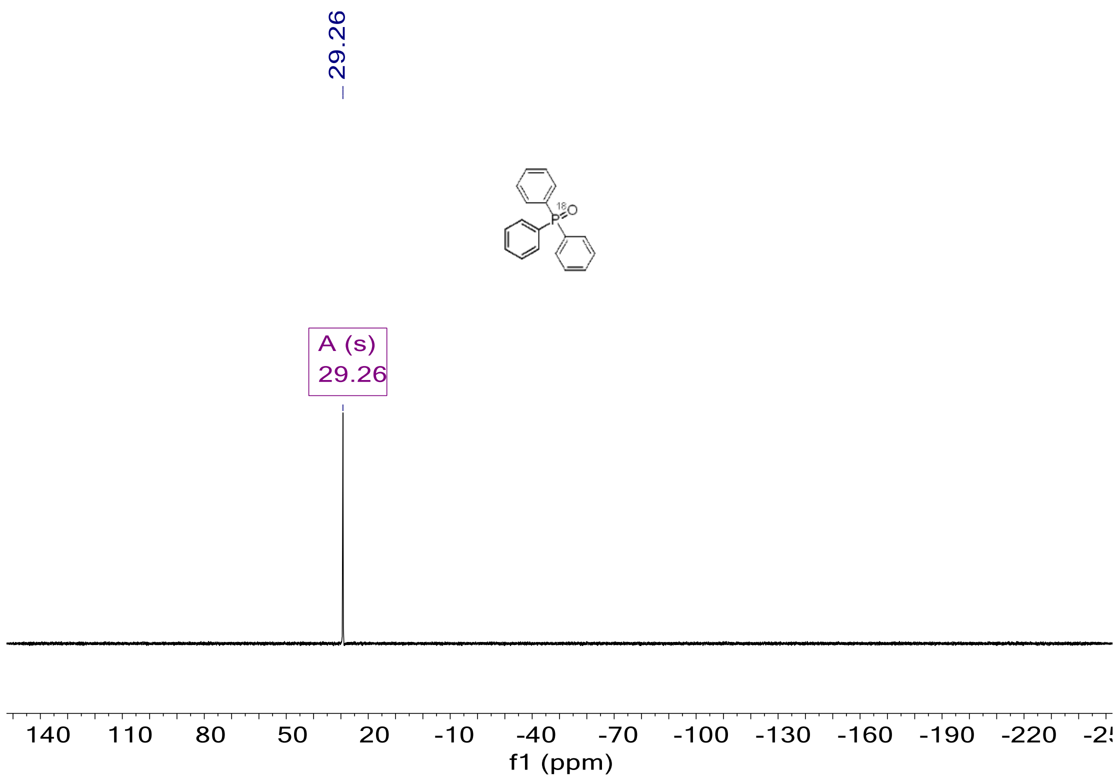

^{31}P-NMR (202 MHz, 298 K, Chloroform-*d*) spectra for **^{18}OPPh$_3$**

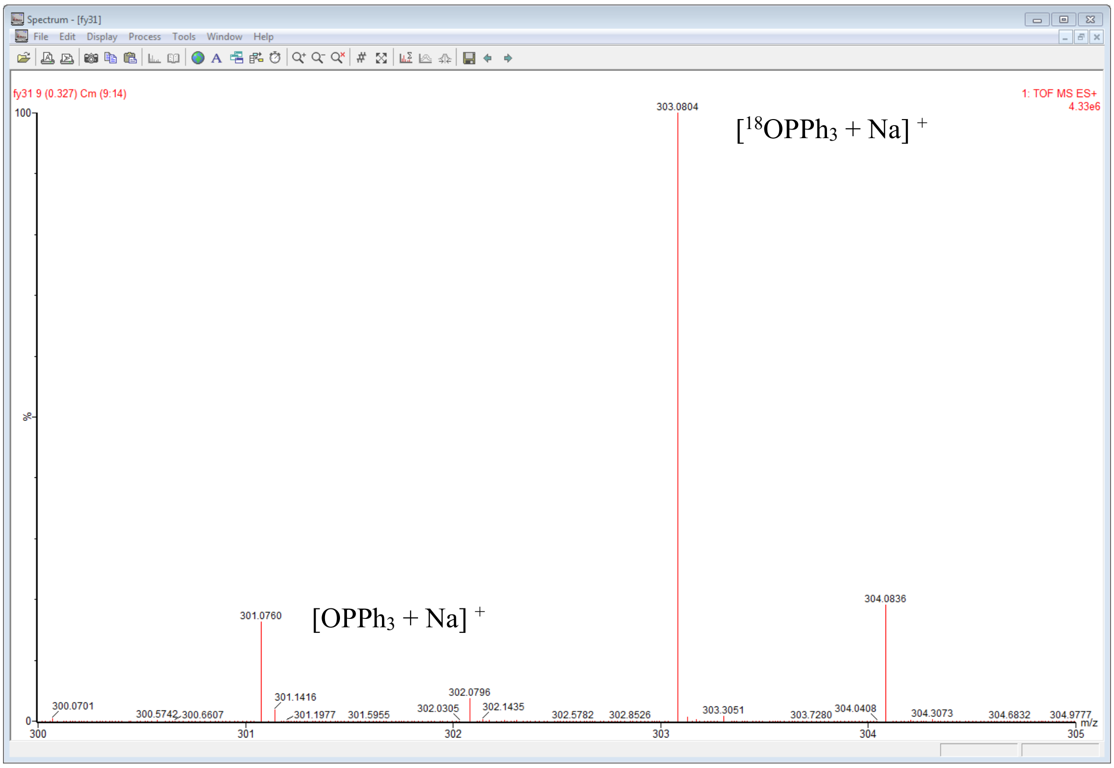

HRMS for **^{18}OPPh$_3$**

D sources experiments

Supplementary Table 2. Reaction conditions for D sources experiment.

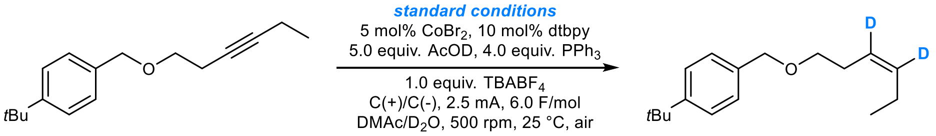

Entry	Variation	GC Yield (%)	D content (%)
1	none	92	92 / 91
2	AcOH and DMAc/D_2O used	88	74 / 73
3	AcOD and DMAc/H_2O used	85	<5 / <5
4	AcOH and DMAc/H_2O used	87	<5 / <5
5	AcOD and DMAc/EtOH used	61	5 / 5
6	AcOD and DMAc/*t*BuOH used	34	7 / 7
7	DMAc/D_2O (3.6 mL/0.2 mL) used	87	92 / 91

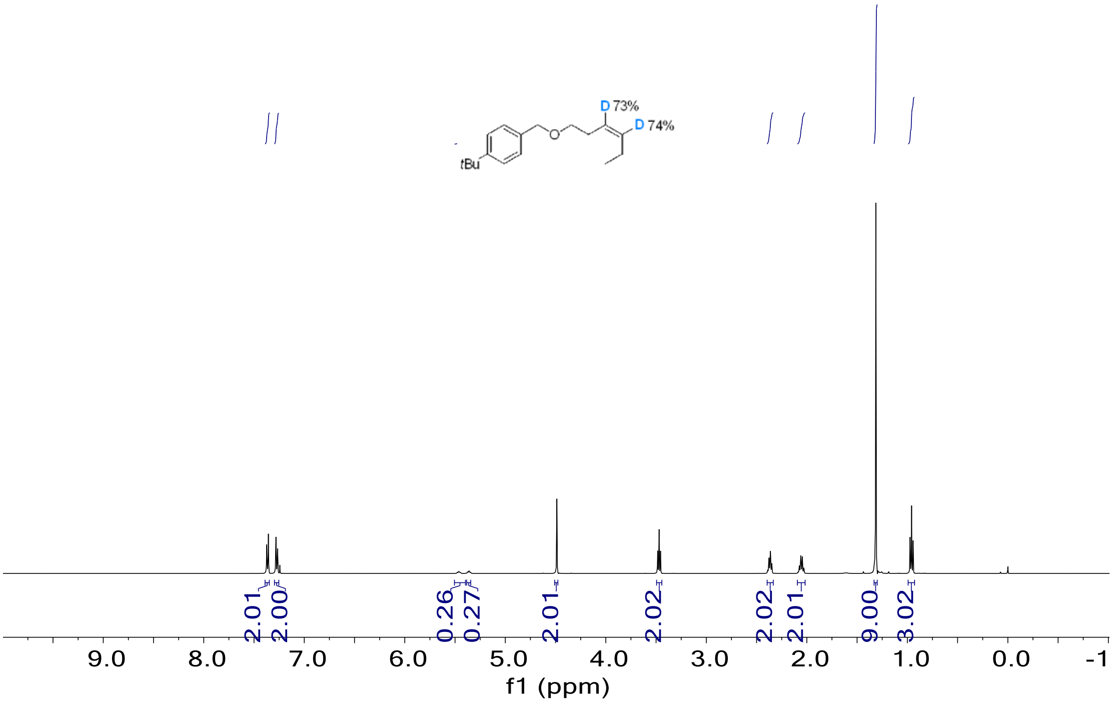

^{1}H-NMR (500 MHz, 298 K, Chloroform-*d*) spectra for **Entry 2**

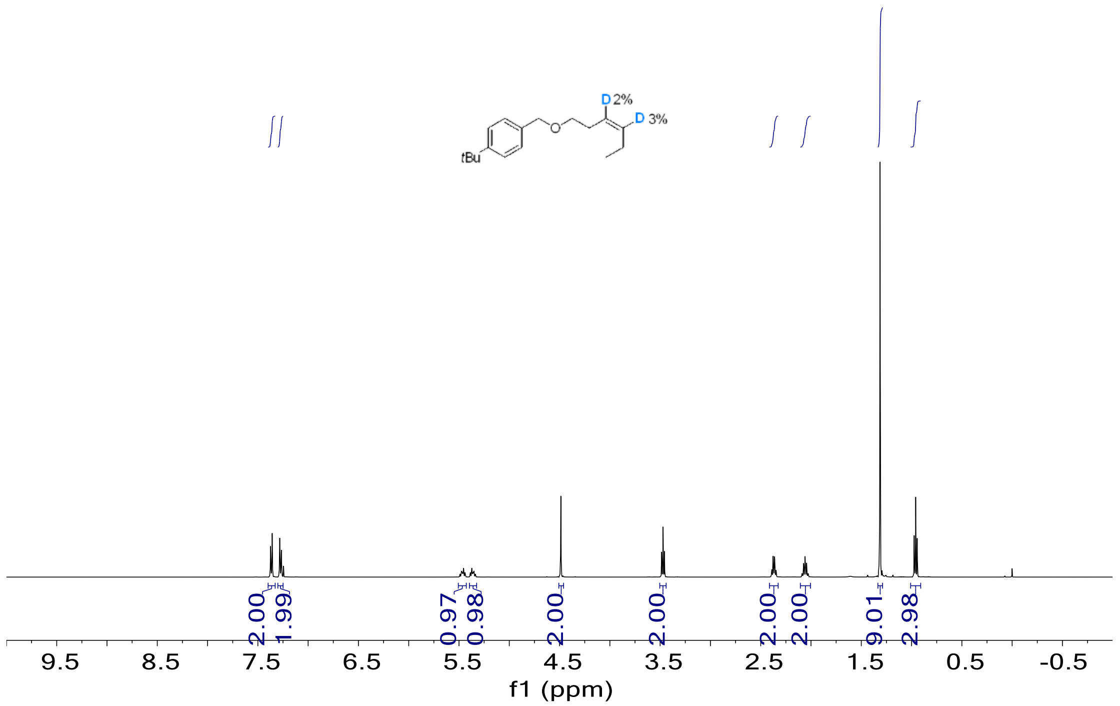

^{1}H-NMR (500 MHz, 298 K, Chloroform-*d*) spectra for **Entry 3**

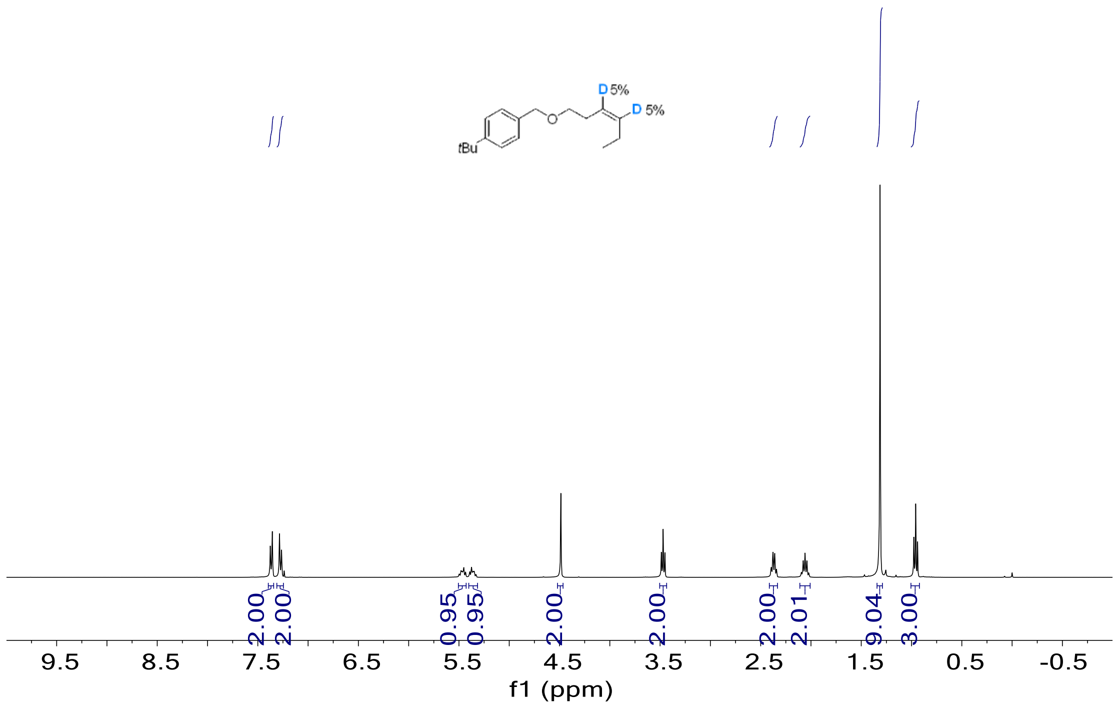

^{1}H-NMR (400 MHz, 298 K, Chloroform-*d*) spectra for **Entry 5**

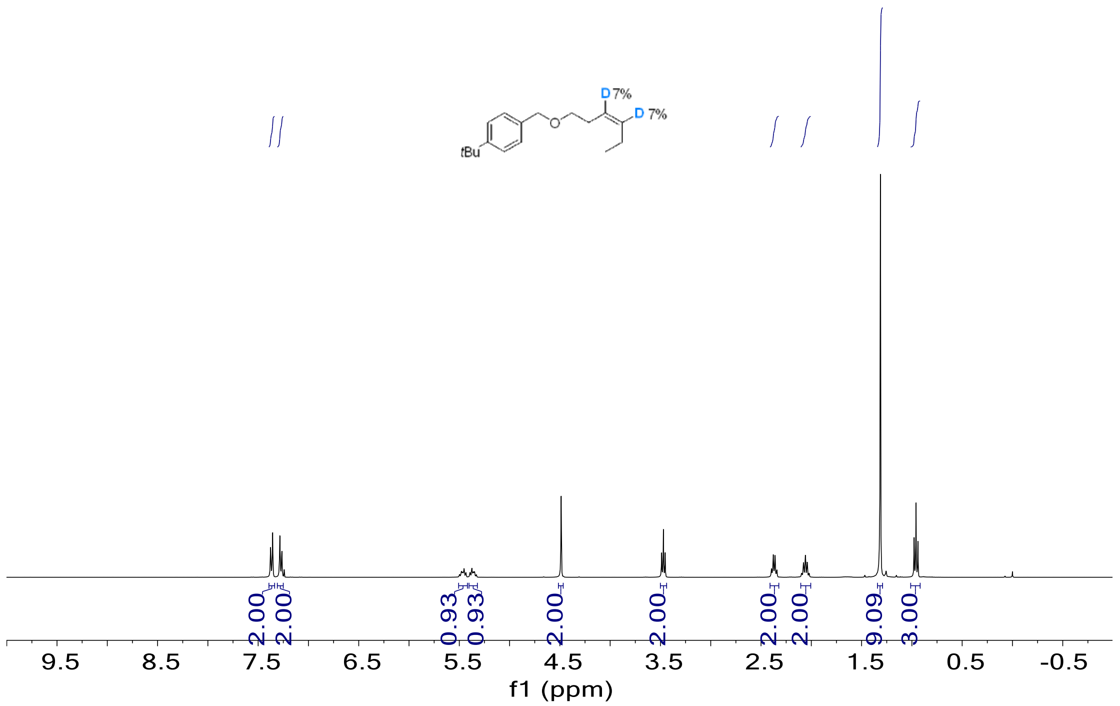

^{1}H-NMR (400 MHz, 298 K, Chloroform-*d*) spectra for **Entry 6**

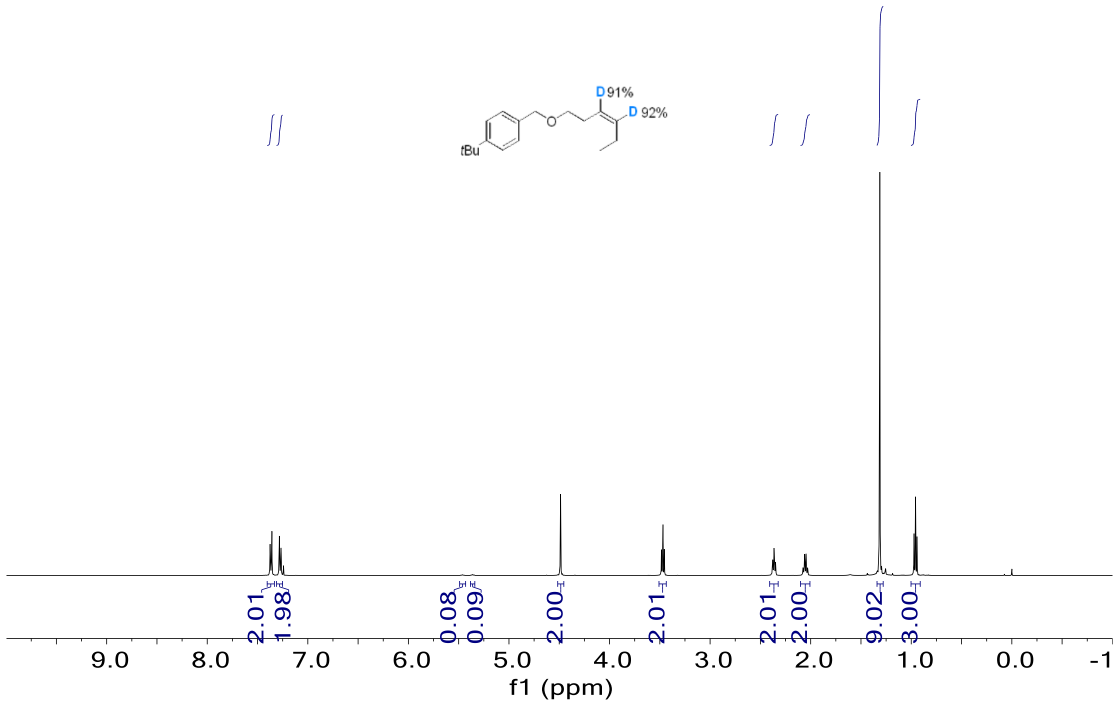

^{1}H-NMR (500 MHz, 298 K, Chloroform-*d*) spectra for **Entry 7**

Non-rinse experiments

Supplementary Table 3. Reaction conditions for non-rinse experiments.

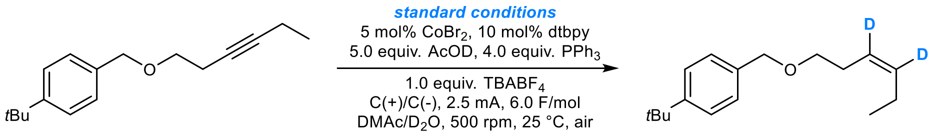

	brand new electrodes	non-rinsed electrodes
with $CoBr_2$ and dtbpy	92% yield, D content: 92%/91%	89% yield, D content: 92%/91%
without $CoBr_2$ and dtbpy	<2% yield	4% yield

Brand new electrodes were purchased from different store on www.1688.com. Without further processing, only ethyl acetate was used to rinse off the graphite powder on the surface of the brand new electrodes. To avoid errors caused by the previous experiment, non-rinse electrodes refer to electrodes that are only rinsed with EA drops to remove solvents on the surface, without deeper cleaning, including sanding with sandpaper and multiple washes with ethyl acetate and acetone. After the first experiment using the brand new electrodes, we dropped ethyl acetate on the graphite electrodes to wash off the previous reaction solution. There were indeed some deposits with luster on the surface of the graphite electrodes. However, these deposits did not affect the reaction. In the absence of cobalt and ligand, almost no target product can be obtained.

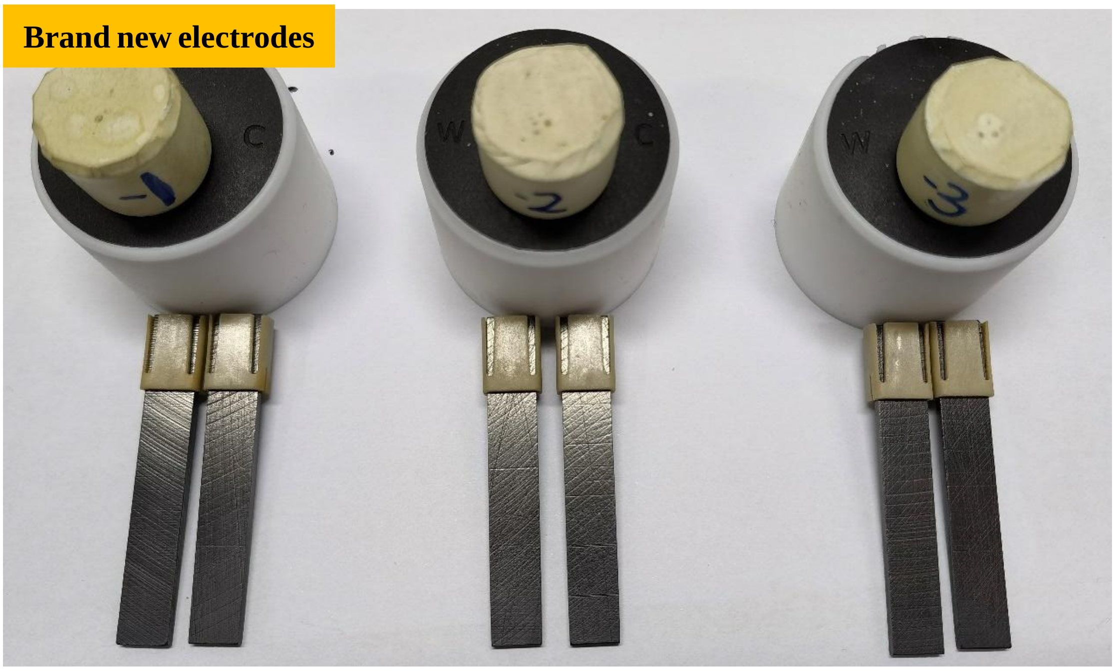

Supplementary Figure 3. Brand new electrodes.

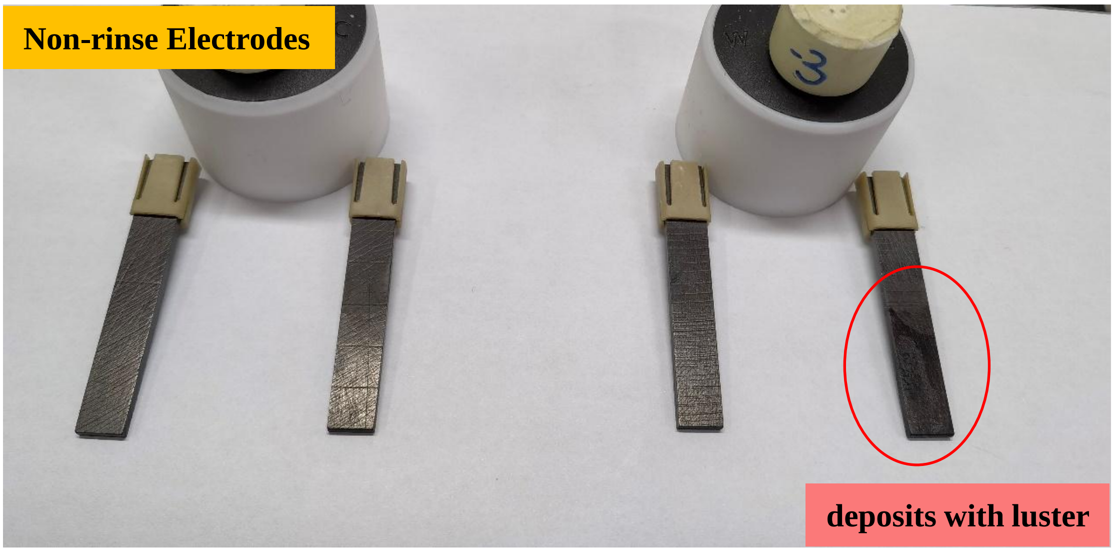

Supplementary Figure 4. Non-rinse electrodes after using 1 time.
The left electrodes were non-rinse electrodes without added $CoBr_2$-2dtbpy catalyst; the right electrodes were non-rinse electrodes with added $CoBr_2$-2dtbpy catalyst.

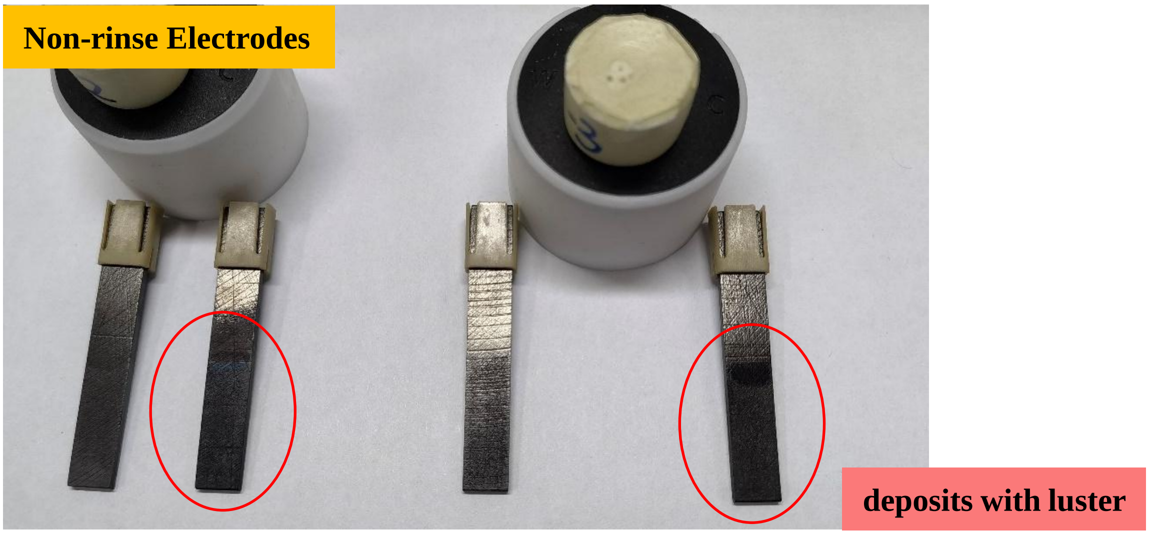

Supplementary Figure 5. Non-rinse electrodes after using 2 times.
The left electrodes were same electrodes as Supplementary Figure 4's left electrodes; these electrodes were used with added $CoBr_2$-2dtbpy catalyst, and they were non-rinse electrodes after electrolysis.
The right electrodes were same electrodes as Supplementary Figure 4's right electrodes; these electrodes were used without added $CoBr_2$-2dtbpy catalyst, and they were non-rinse electrodes after electrolysis.

Non-rinse tests were conducted to examine the catalytic properties of an electrode that had already been used in at least one catalytic cycle.

Brand new electrodes were purchased from different store on www.1688.com. In the non-rinse tests, only the newly purchased electrodes with sufficient rinse by ethyl acetate and acetone can be considered as ‘brand new electrodes’. However, electrodes that have undergone deep cleaning, including sanding with sandpaper and multiple washes with ethyl acetate and acetone, are also considered as ‘clean electrodes’ in other experiments.

Non-rinse electrodes refer to electrodes that had already been used in at least one catalytic cycle and only rinsed with ethyl acetate drops to remove solvents on the surface, without deeper cleaning, including sanding with sandpaper and multiple washes with ethyl acetate and acetone. Due to the ability of the reaction solution to wet the electrode surface, the reaction solution not only contains homogeneous cobalt catalyst, but also directly contains some reaction products. If these attached reaction solutions are carried into the next cycle, it can lead to unpredictable and irreproducible errors. We dropped ethyl acetate on the electrodes to wash off the previous reaction solutions. This very gentle rinse is necessary.

In addition, there were indeed some deposits with luster on the surface of the electrodes. However, these deposits did not affect the reaction. In the absence of cobalt and ligand, almost no target product (only with 4% yield) can be obtained.

CV experiments

All the cyclic voltametric experiments were recorded with a Signal 1000E potentiostat room temperature in DMAc within a glovebox. $TBABF_4$ (0.1 M) was used as the supporting electrolyte, and a 3 mm glassy carbon electrode served as the working electrode. The counter electrode was a coiled Pt wire, and the reference electrode was a commercial Ag/AgCl electrode. All potentials were referenced against ferrocene. The scan rate was set to 50 mV/s, with a sampling interval of 2 mV.

Before each experiment, it was necessary to polish the glassy carbon electrode to ensure that the electrode surface was clean.

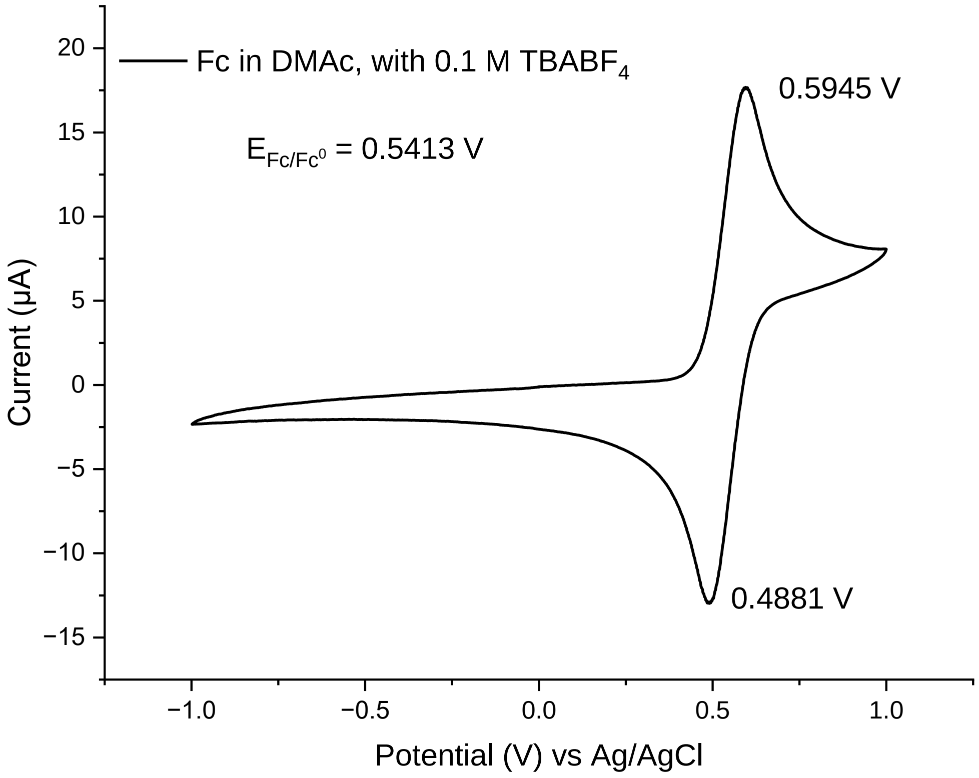

Supplementary Figure 6. Cyclic voltametric profiles of 3 mM Ferrocene in DMAc solvent with 0.1 M $TBABF_4$ at 50 mV/s.

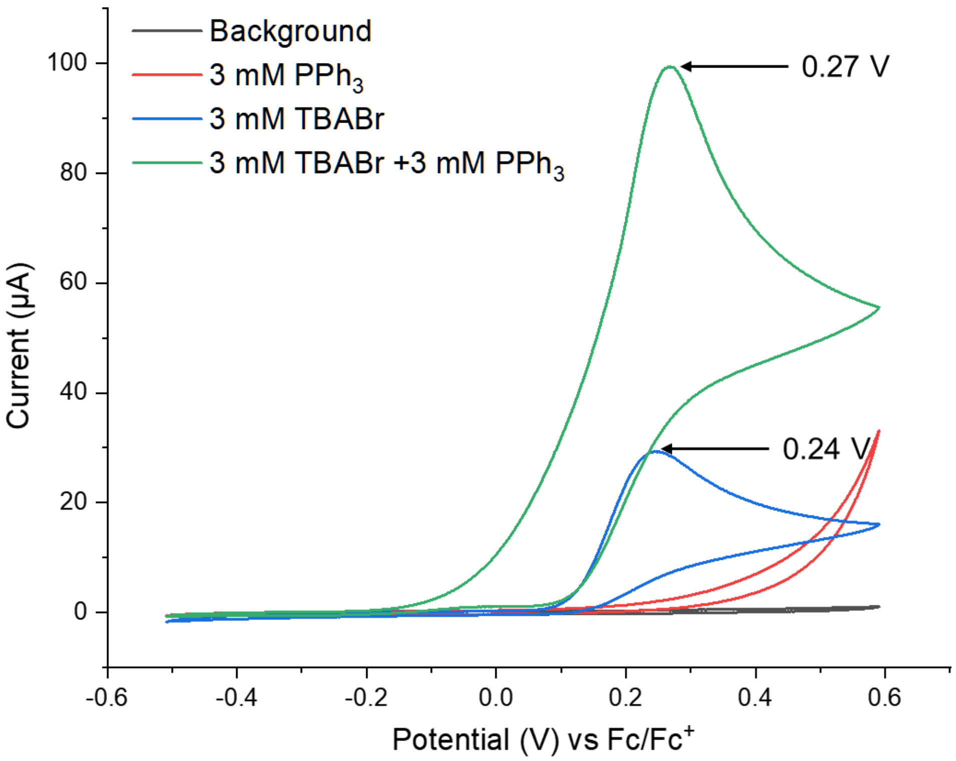

Supplementary Figure 7. Cyclic voltametric profiles of background, 3 mM PPh_3, 3 mM TBAB, 3 mM TBAB + 3 mM PPh_3 in DMAc solvent with 0.1 M $TBABF_4$ at 50 mV/s.

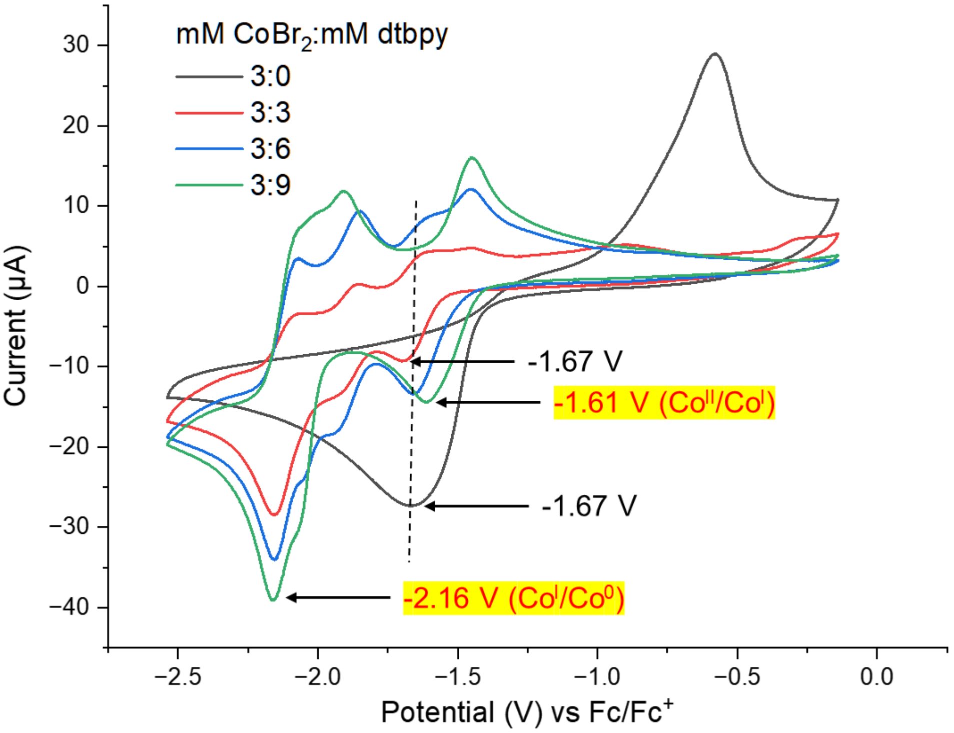

Supplementary Figure 8. Cyclic voltametric profiles of 3 mM $CoBr_2$, 3 mM $CoBr_2$ &3 mM dtbpy, 3 mM $CoBr_2$ &6 mM dtbpy, 3 mM $CoBr_2$ & 9 mM dtbpy in DMAc solvent with 0.1 M $TBABF_4$ at 50 mV/s.

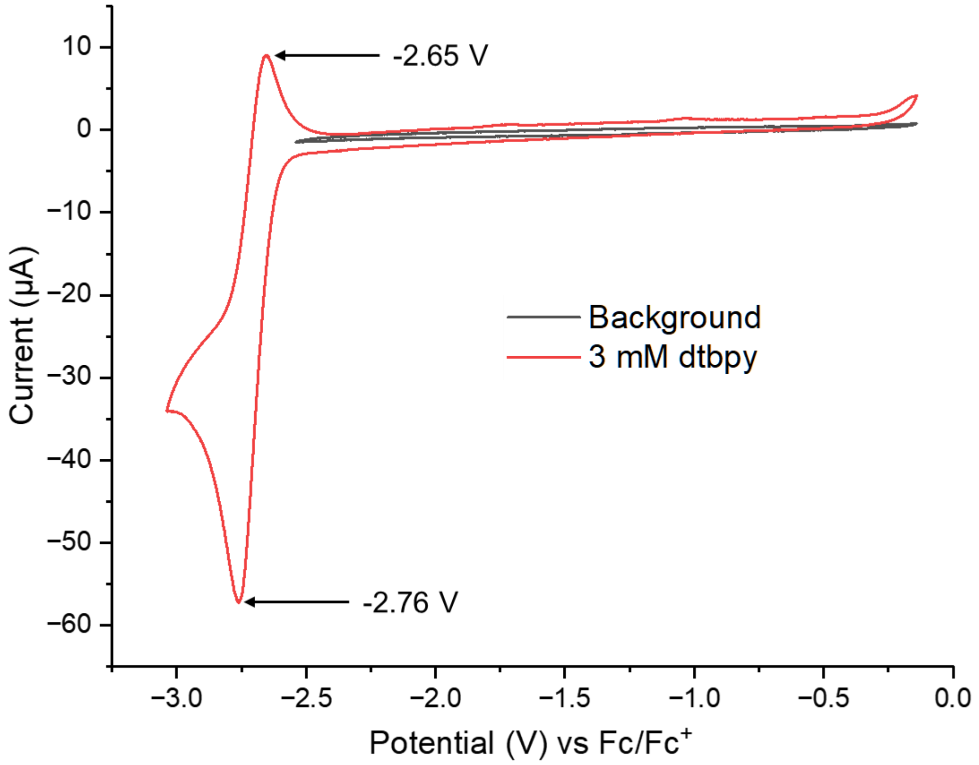

Supplementary Figure 9. Cyclic voltametric profiles of background, 3 mM dtbpy in DMAc solvent with 0.1 M $TBABF_4$ at 50 mV/s.

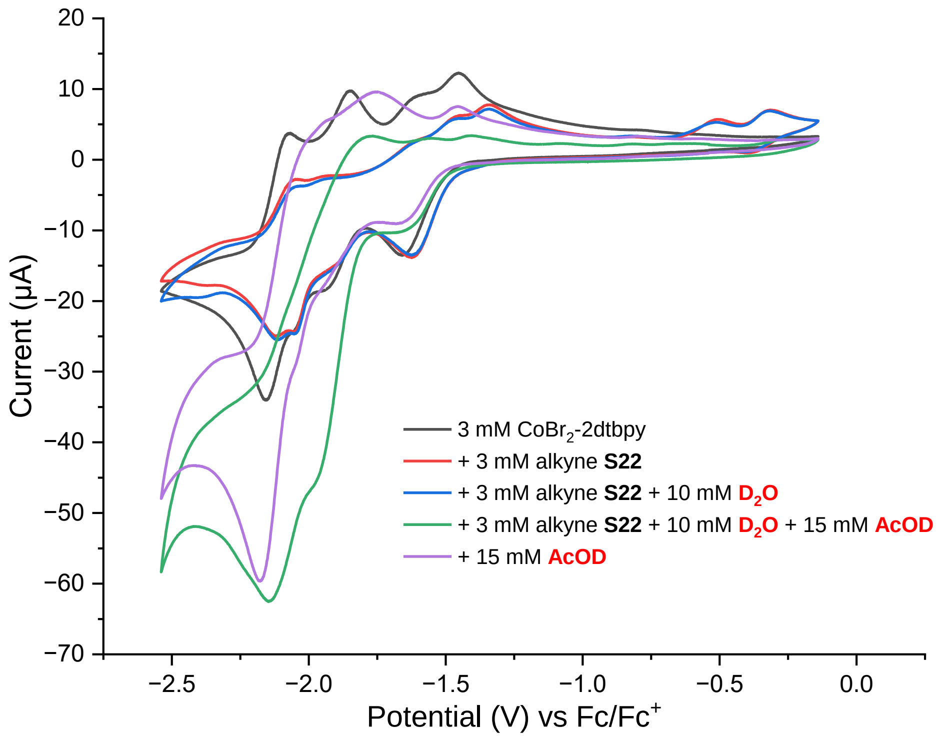

Supplementary Figure 10. Cyclic voltametric profiles of 3 mM $CoBr_2$ & 6 mM

dtbpy (black line), 3 mM $CoBr_2$ & 6 mM dtbpy + 3 mM alkyne **S22** (red line), 3 mM $CoBr_2$ &6 mM dtbpy + 3 mM alkyne **S22** + 10 mM D_2O (blue line), 3 mM $CoBr_2$ & 6 mM dtbpy + 3 mM alkyne **S22** + 10 mM D_2O + 15 mM AcOD (green line), 3 mM $CoBr_2$ & 6 mM dtbpy 15 mM AcOD (purple line) in DMAc solvent with 0.1 M $TBABF_4$ at 50 mV/s.

Kinetic experiments

The general method for electrochemistry kinetic studies:
To a 5 mL vial equipped with a magnetic stirring bar, $CoBr_2$ (0.01 mmol, 5 mol%), dtbpy (0.02 mmol, 10 mol%), $TBABF_4$ (0.20 mmol, 1.0 equiv.), and PPh_3 (0.80 mmol, 4.0 equiv.) were added. Subsequently, DMAc (3.6 mL) was added, and the solution was stirred for 5 minutes to ensure complete dissolution. To this solution, Na_2SO_4 was added for the purposes of drying and dehydration. After allowing the solution to stand for 30 minutes, Na_2SO_4 was removed by filtration, and the resulting solution was then transferred into a 5 mL ElectraSyn vial that was equipped with a magnetic stirring bar. Following the solution preparation method, alkyne (0.20 mmol, 1.0 equiv.), D sources (including AcOD and D_2O), were added to the solution in Electra-Syn vial via syringe. The Electra-Syn vial cap, equipped with an anode (graphite) and a cathode (graphite), was inserted into the reaction mixture. After pre-stirring for 5 minutes, the reaction mixture was electrolyzed under various constant currents (e.g., 2.5 mA, 5 mA, 7.5 mA) for 6 F/mol, and the stopwatch was started. At fixed time points, the aliquots of the reaction mixture (50 μL) were taken out by syringe. To quench each sample, an aliquot was immediately placed to H_2O & EA (0.1 mL & 0.1 mL). After mixing evenly, the mixture was centrifuged, and the upper organic phase was separated to test GC. The product composition was analyzed by GC.

The different-excess experiments under 2.5 mA

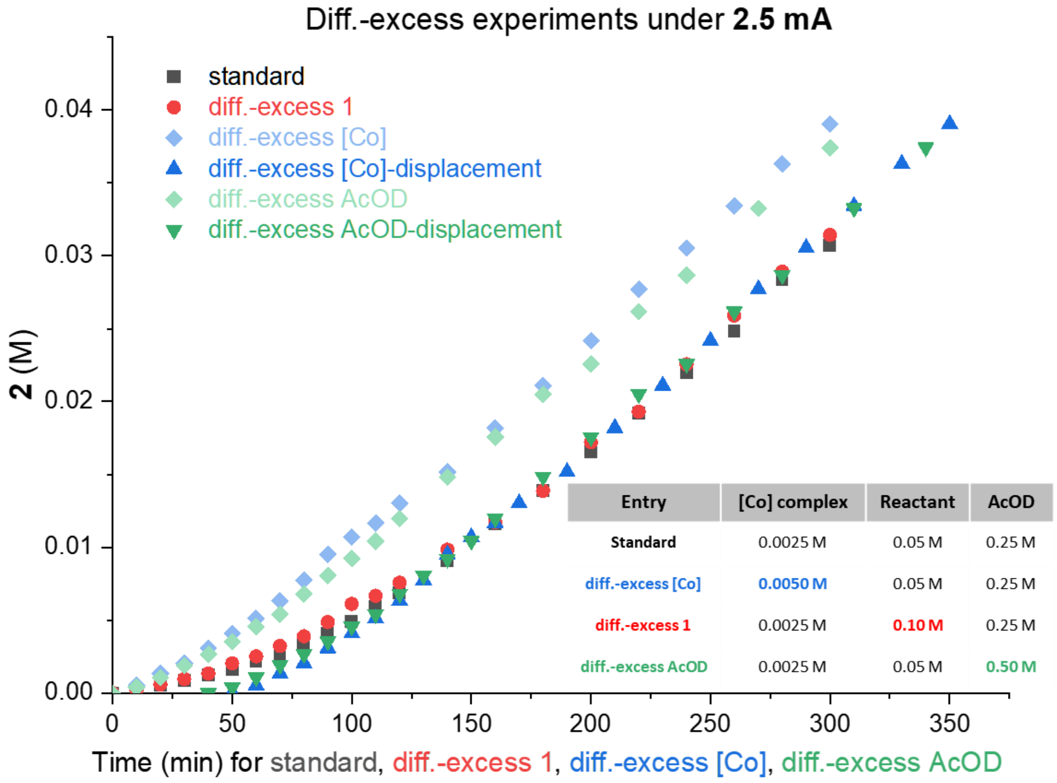

Supplementary Figure 11. Plot of the rise of product from the reaction of **standard**, **diff.-excess [Co]**, **diff.-excess 1**, **diff.-excess AcOD** under 2.5 mA.

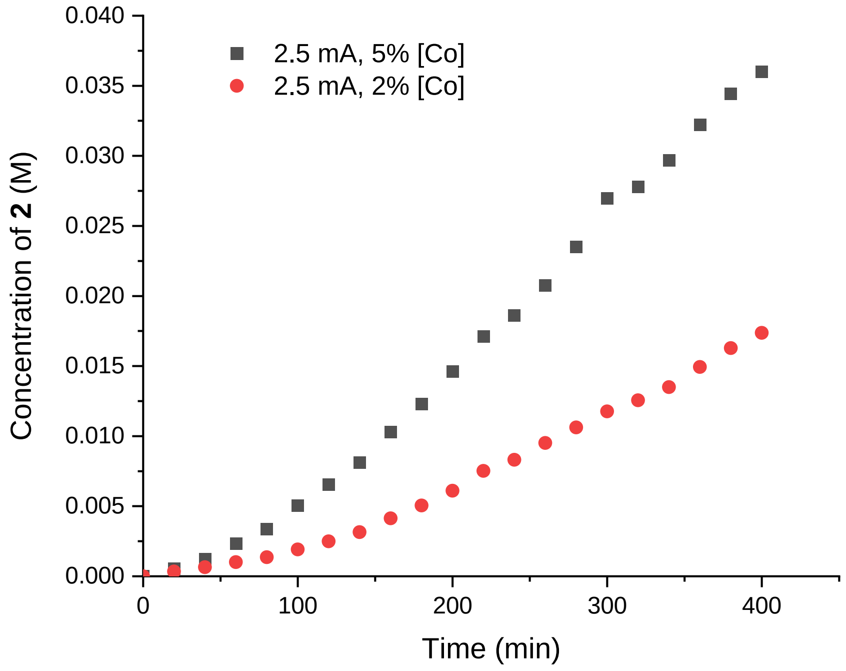

Supplementary Figure 12. Plot of the rise of product from the reaction of **5% or 2% [Co]** under 2.5 mA.

The different-excess experiments under 7.5 mA

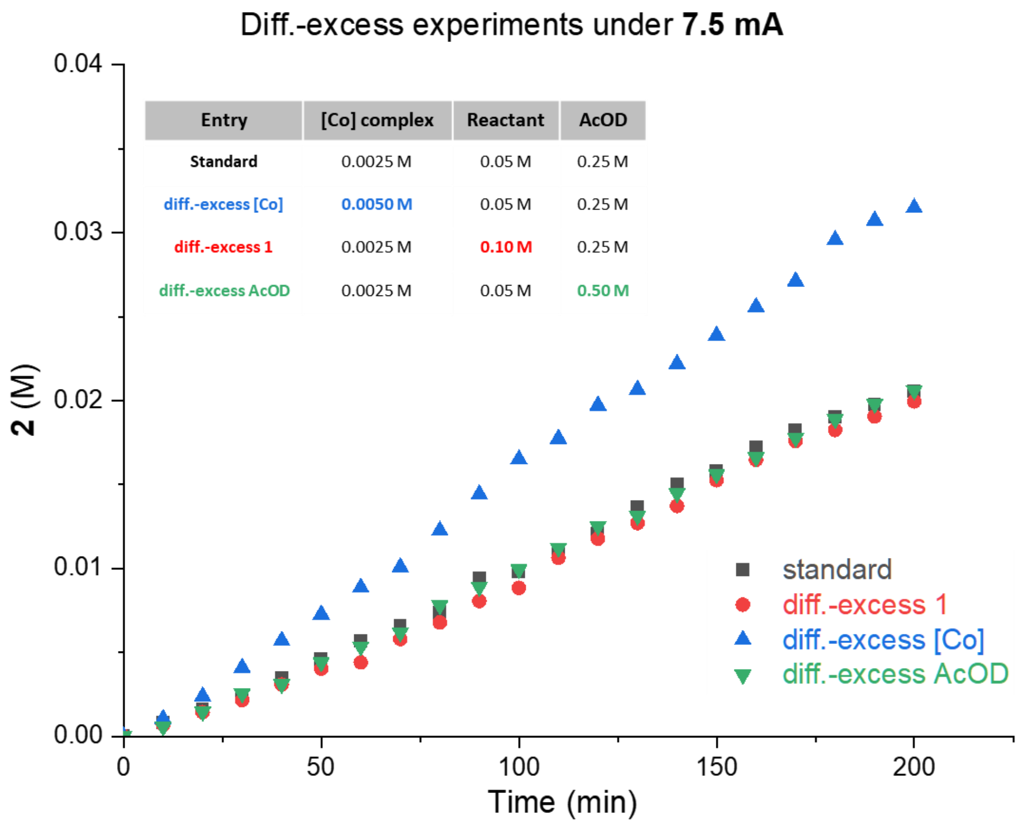

Supplementary Figure 13. Plot of the rise of product from the reaction of **standard**, **diff.-excess [Co]**, **diff.-excess 1**, **diff.-excess AcOD** under 7.5 mA.

Burés graphical rate analysis

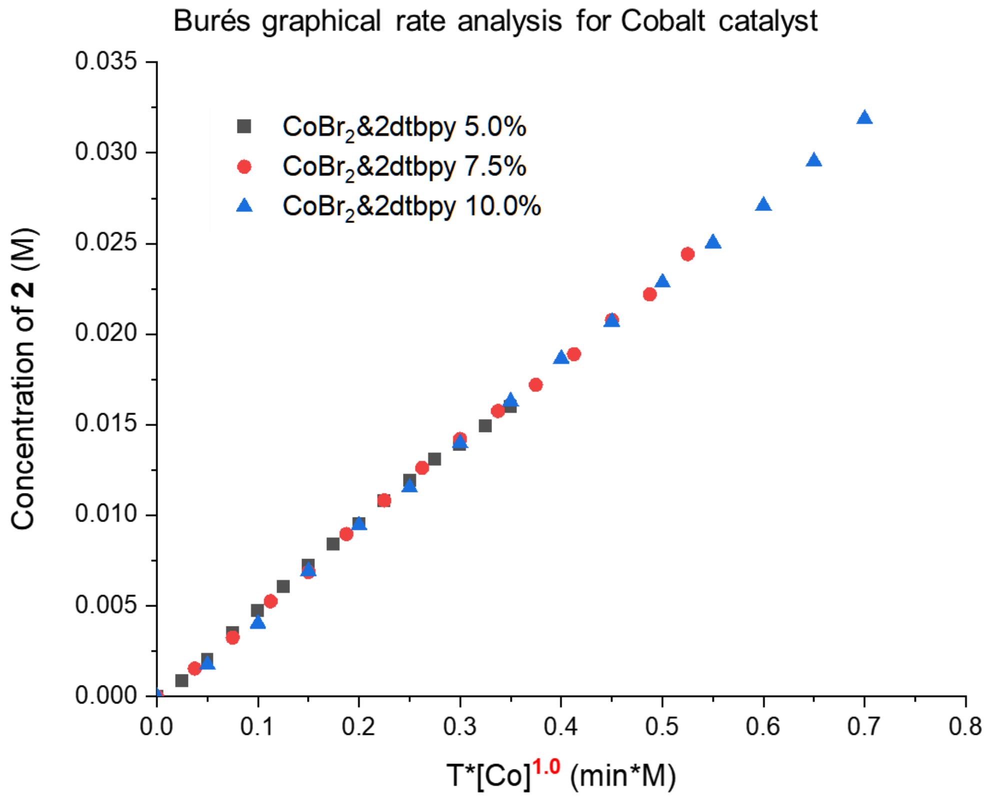

Supplementary Figure 14. Burés graphical rate analysis for the concentration of **2** and the time multiply the concentration of cobalt catalyst (0.0025 M, 0.00375 M, 0.0050 M) under 7.5 mA.

Supplementary Figures of NMR Spectra

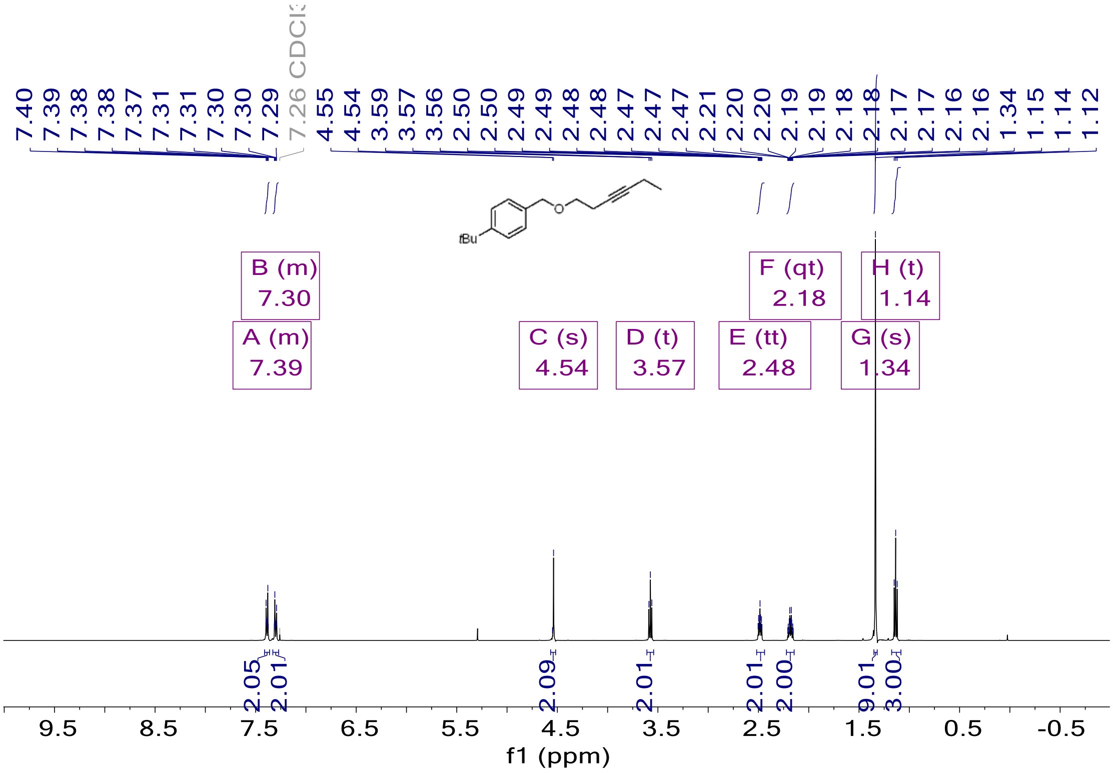

^{1}H NMR (500 MHz, 298 K, Chloroform-*d*) spectra for **1**

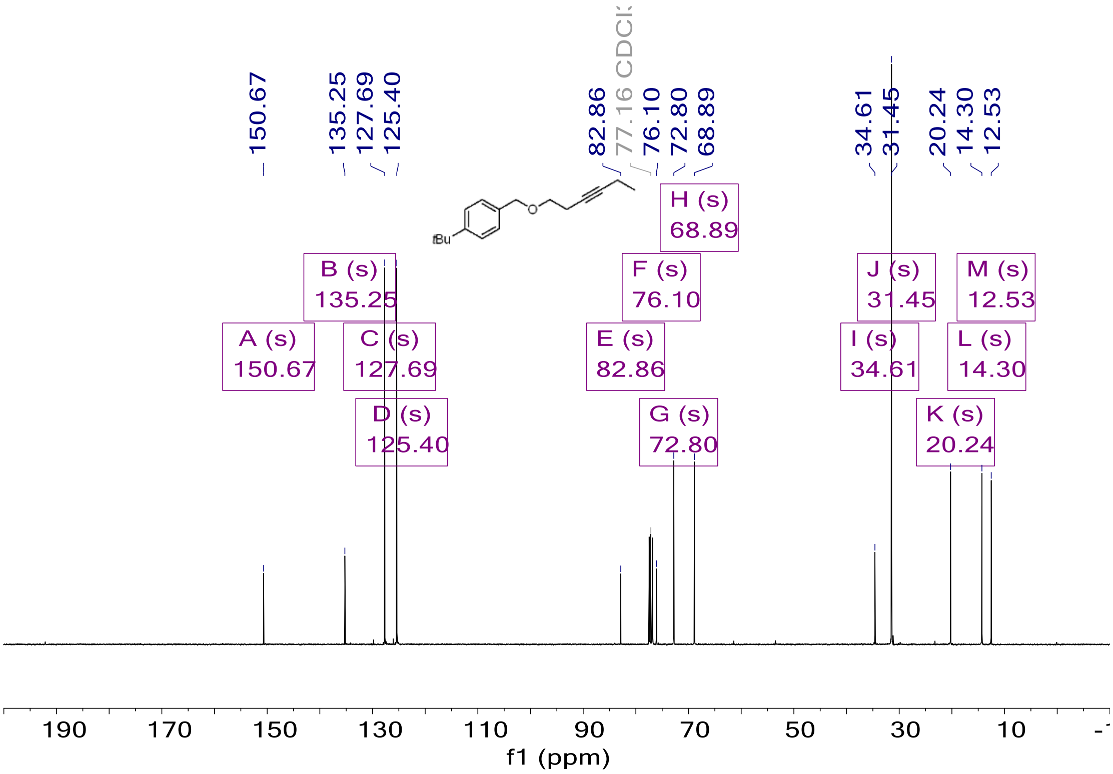

^{13}C NMR (101 MHz, 298 K, Chloroform-*d*) spectra for **1**

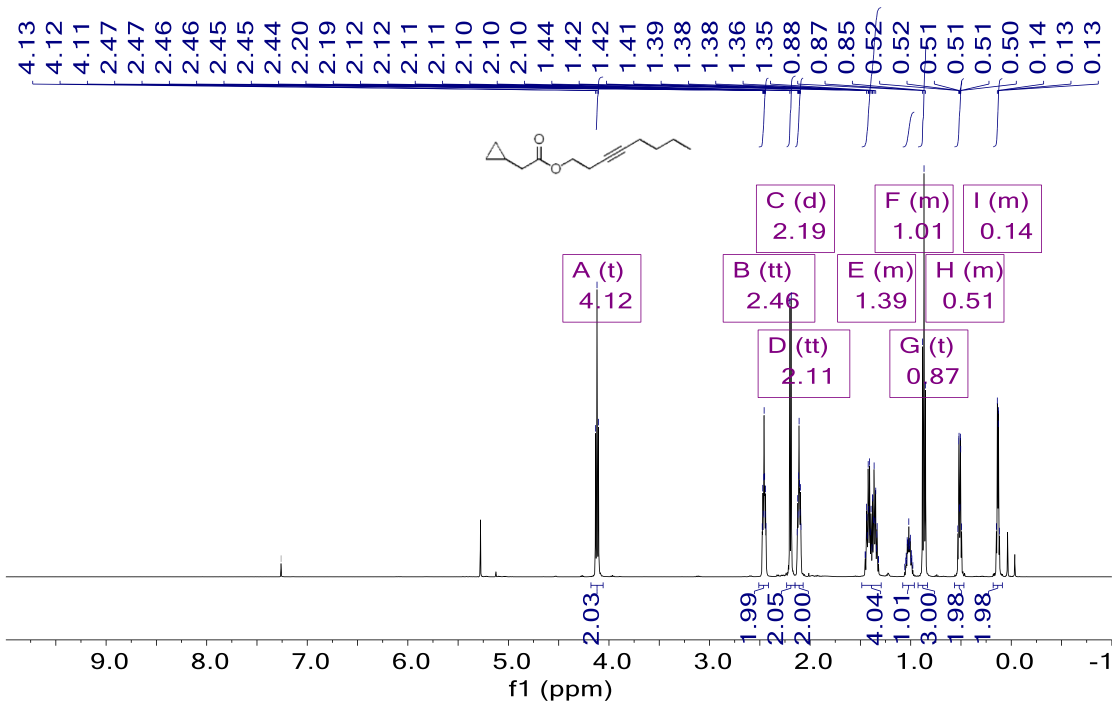

^{1}H NMR (500 MHz, 298 K, Chloroform-*d*) spectra for **S3**

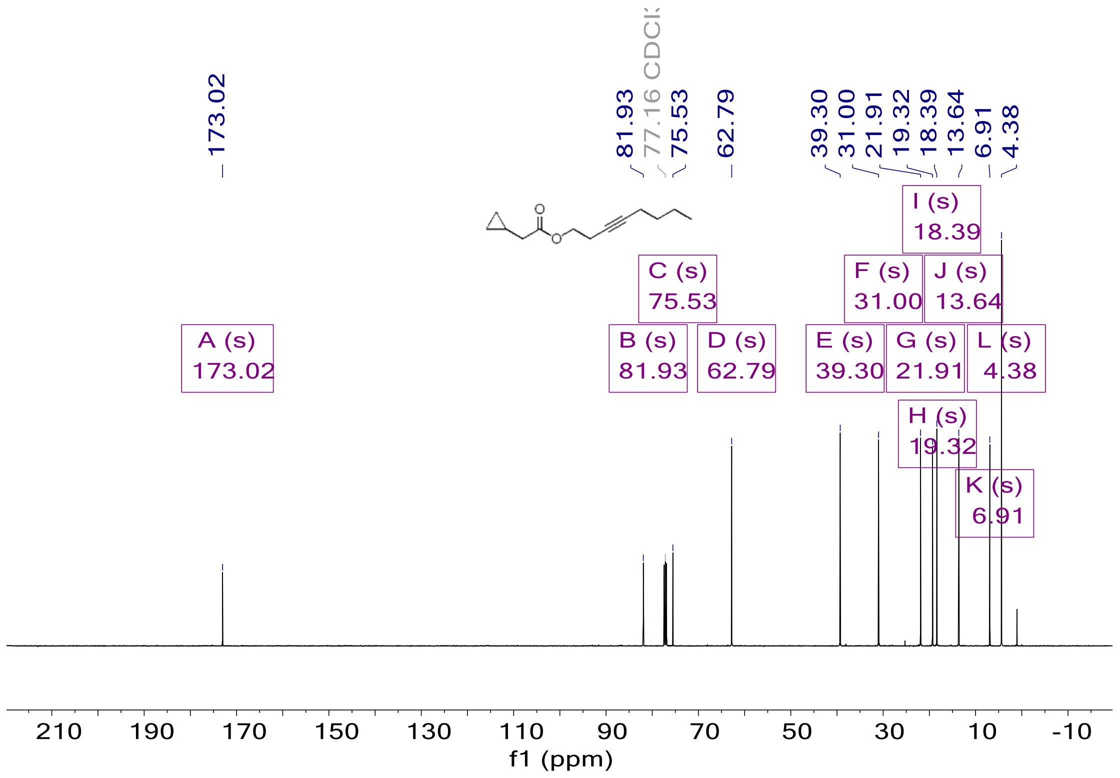

^{13}C NMR (126 MHz, 298 K, Chloroform-*d*) spectra for **S3**

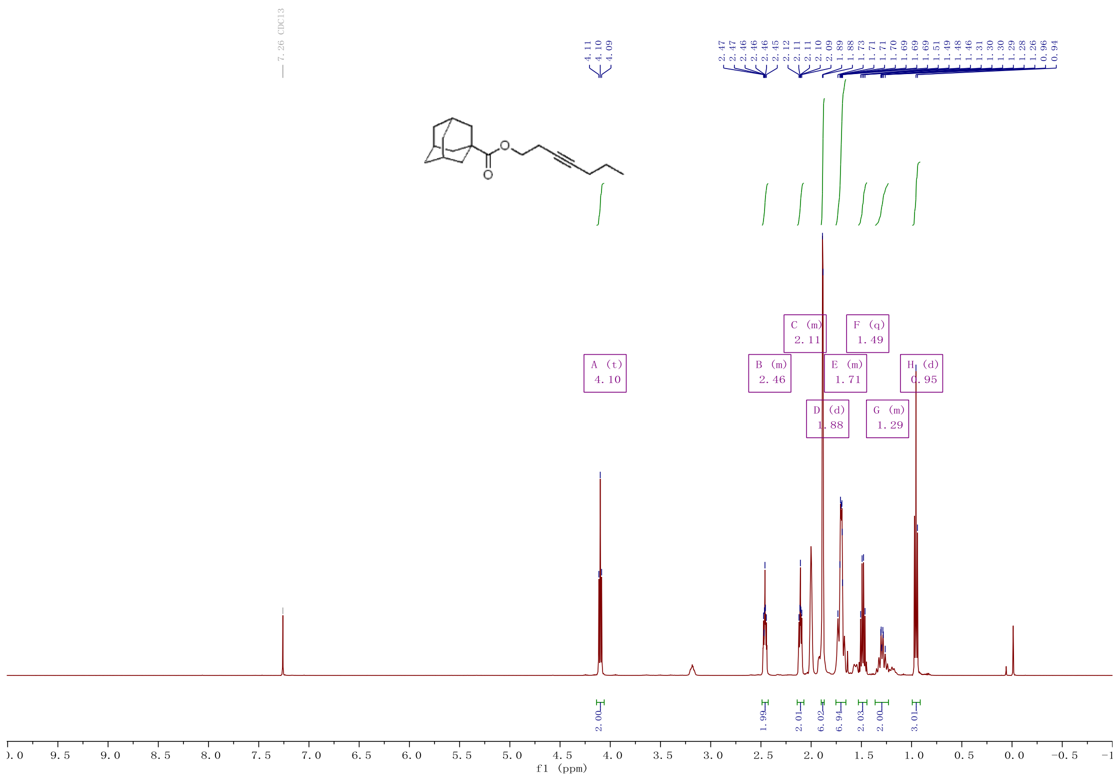

^{1}H NMR (500 MHz, 298 K, Chloroform-*d*) spectra for **S4**

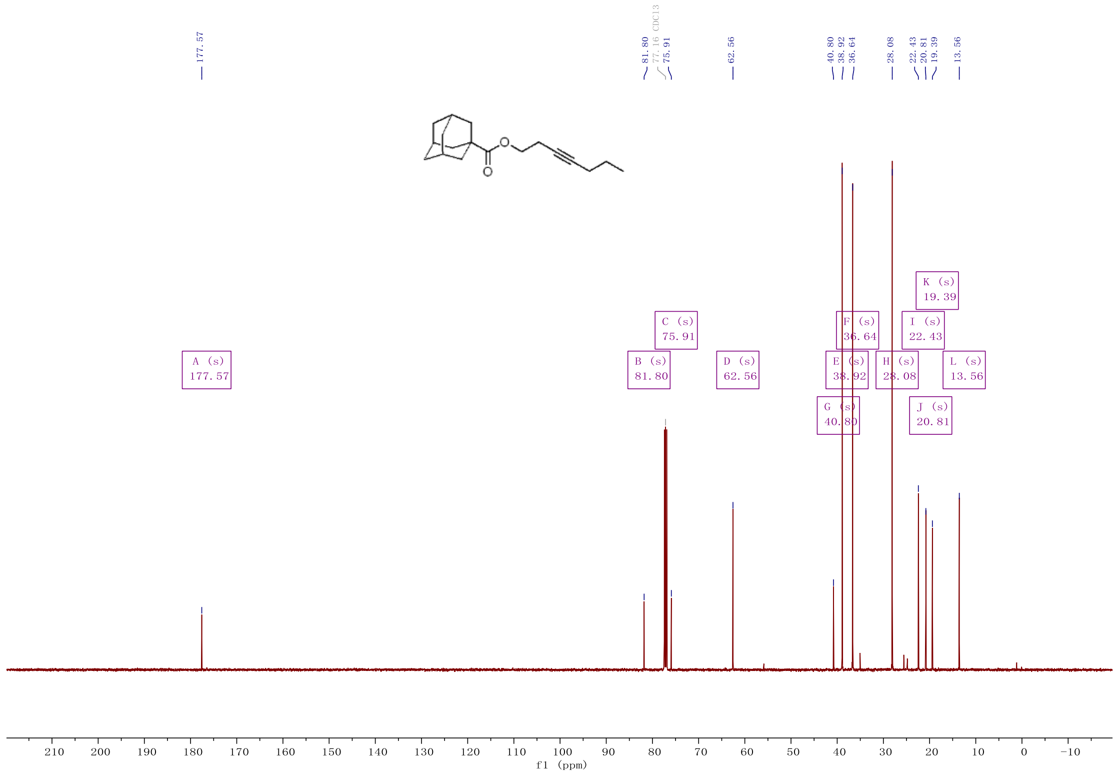

^{13}C NMR (126 MHz, 298 K, Chloroform-*d*) spectra for **S4**

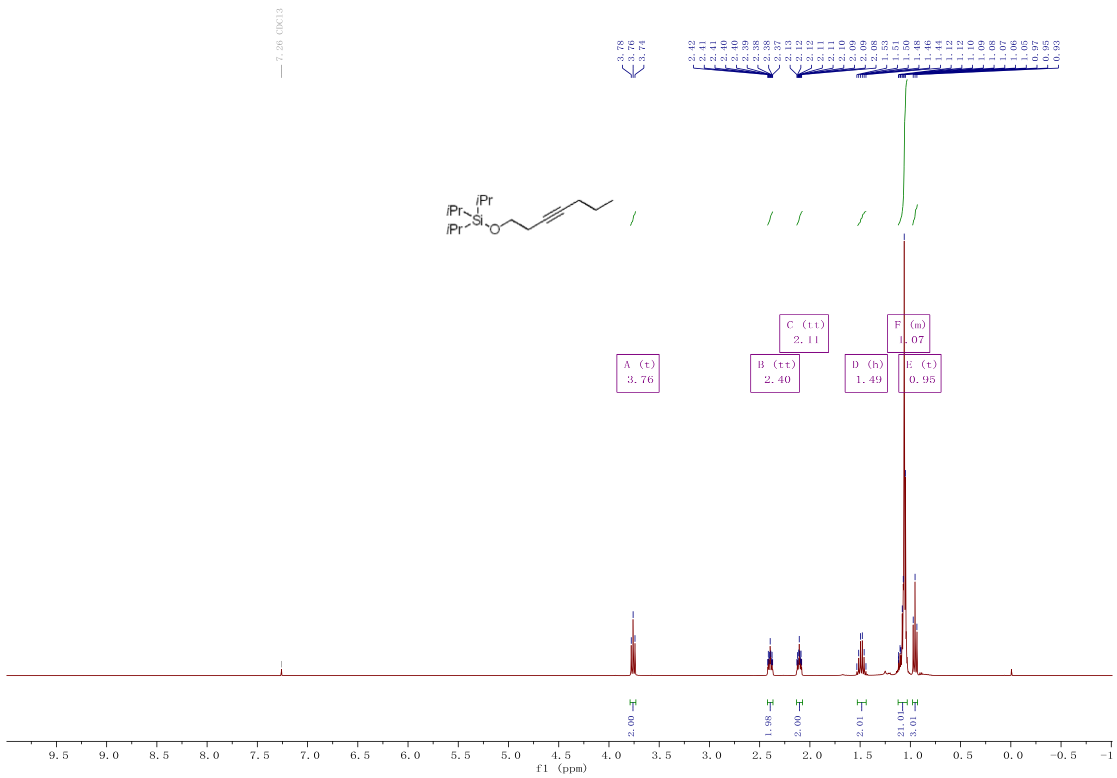

^{1}H NMR (400 MHz, 298 K, Chloroform-*d*) spectra for **S5**

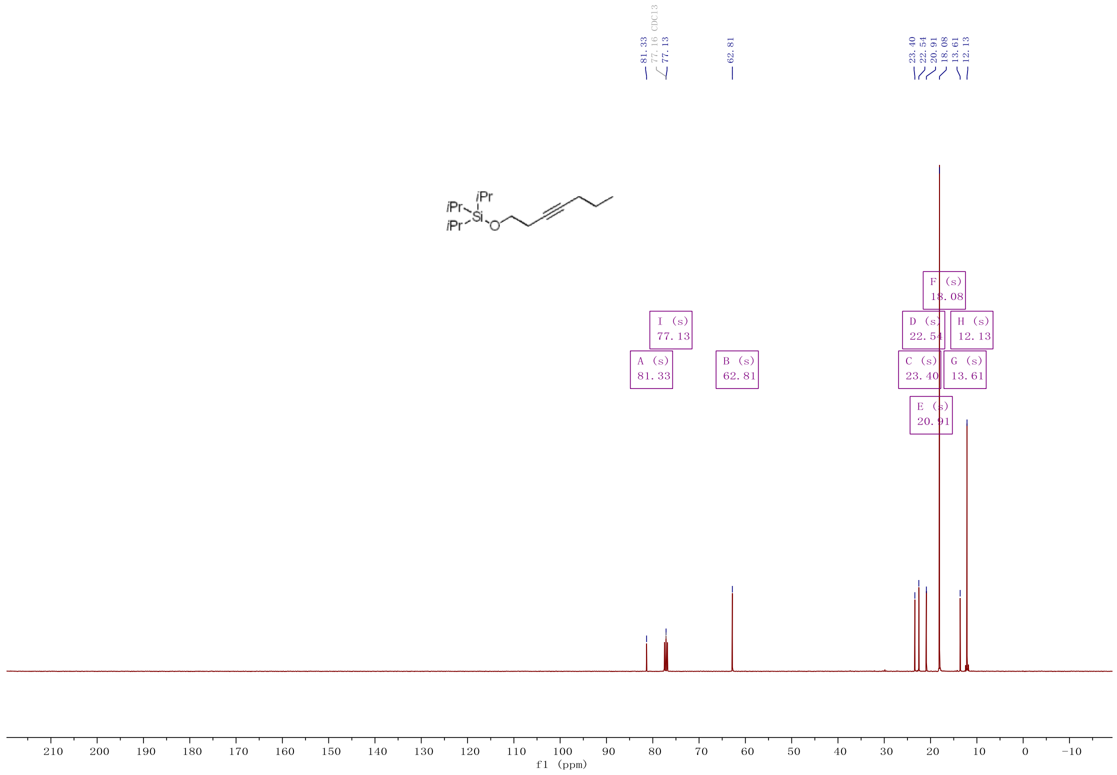

^{13}C NMR (101 MHz, 298 K, Chloroform-*d*) spectra for **S5**

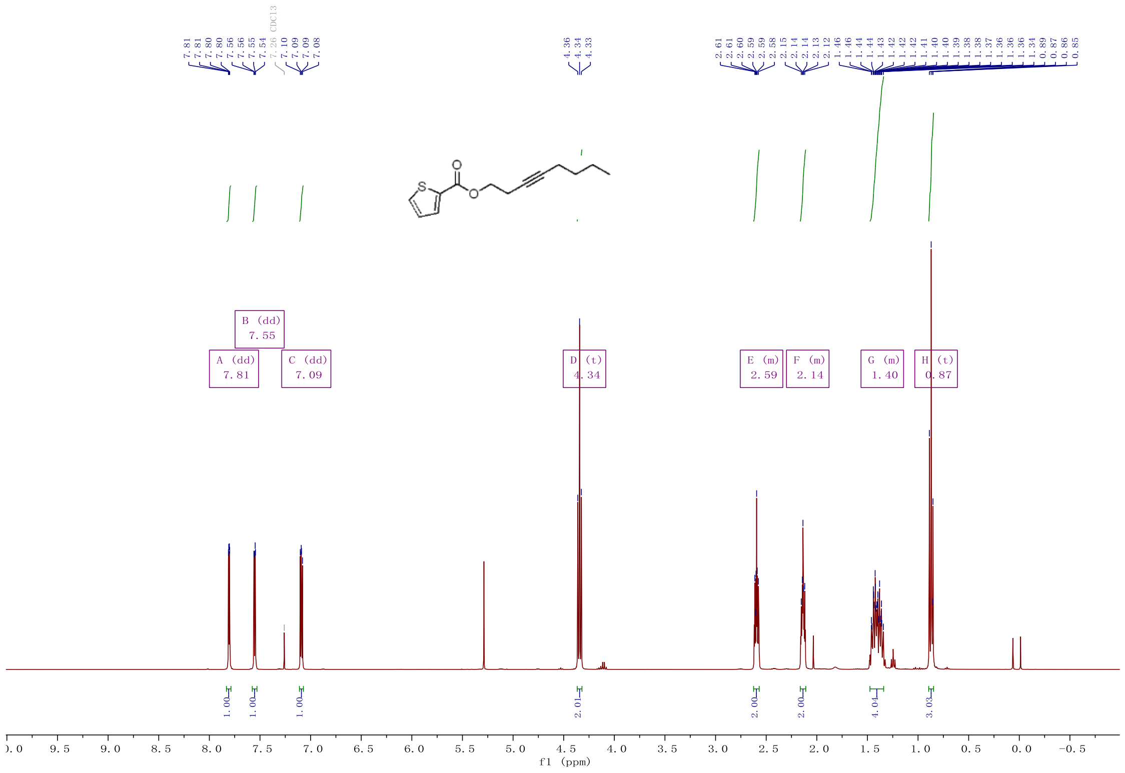

^{1}H NMR (400 MHz, 298 K, Chloroform-*d*) spectra for **S6**

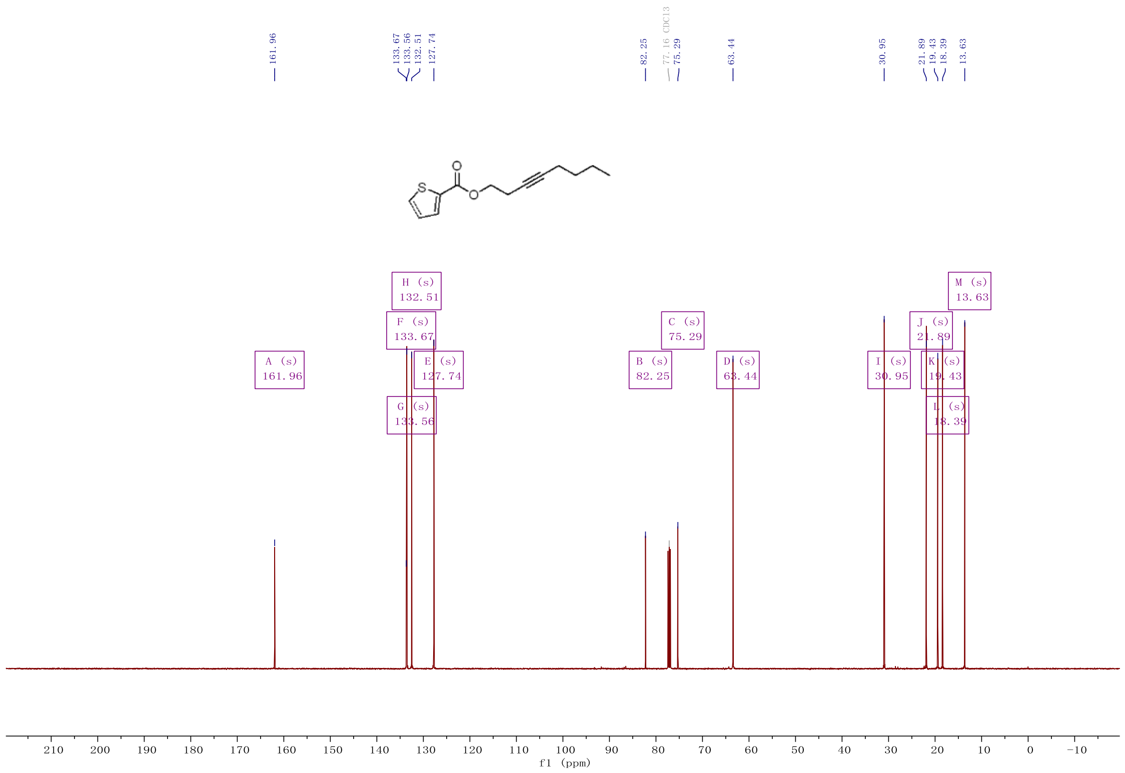

^{13}C NMR (126 MHz, 298 K, Chloroform-*d*) spectra for **S6**

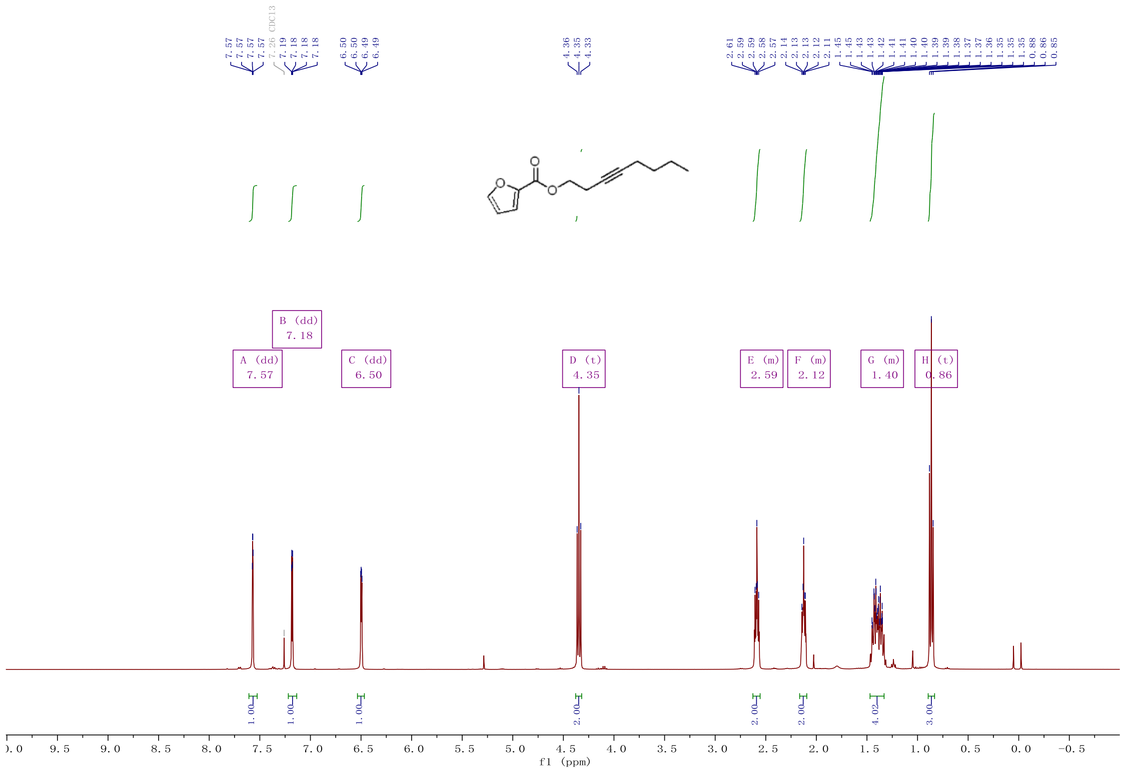

^{1}H NMR (400 MHz, 298 K, Chloroform-*d*) spectra for **S7**

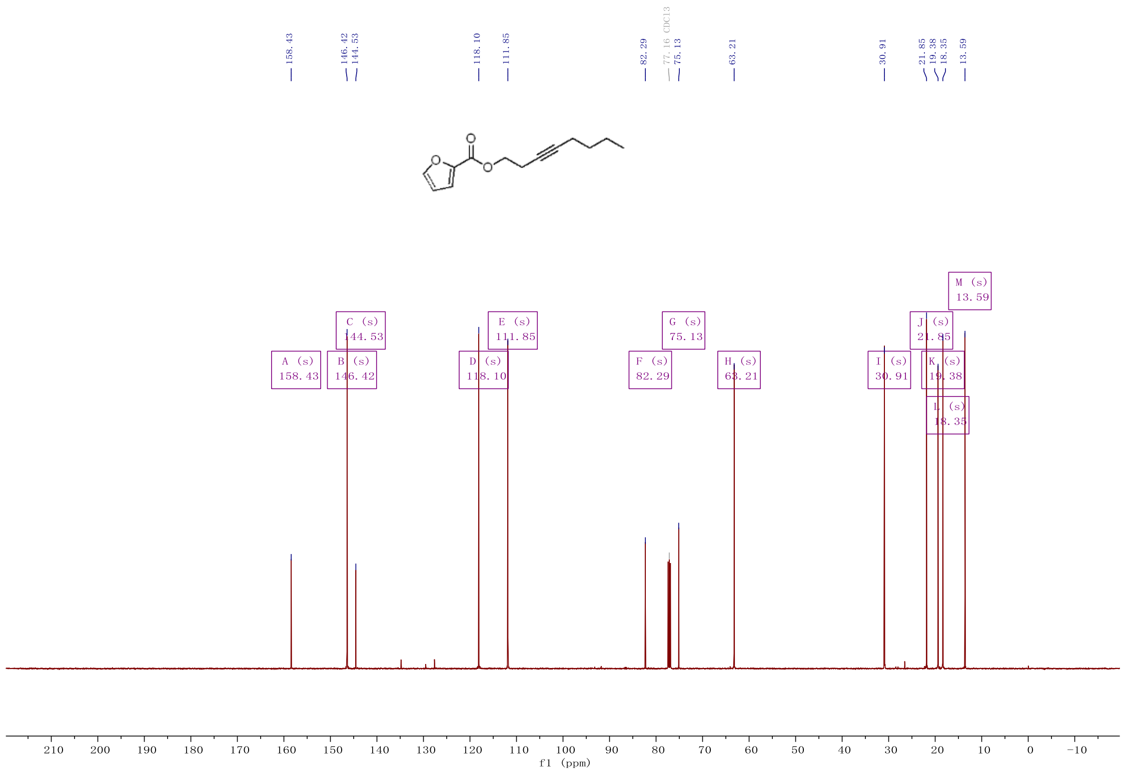

^{13}C NMR (126 MHz, 298 K, Chloroform-*d*) spectra for **S7**

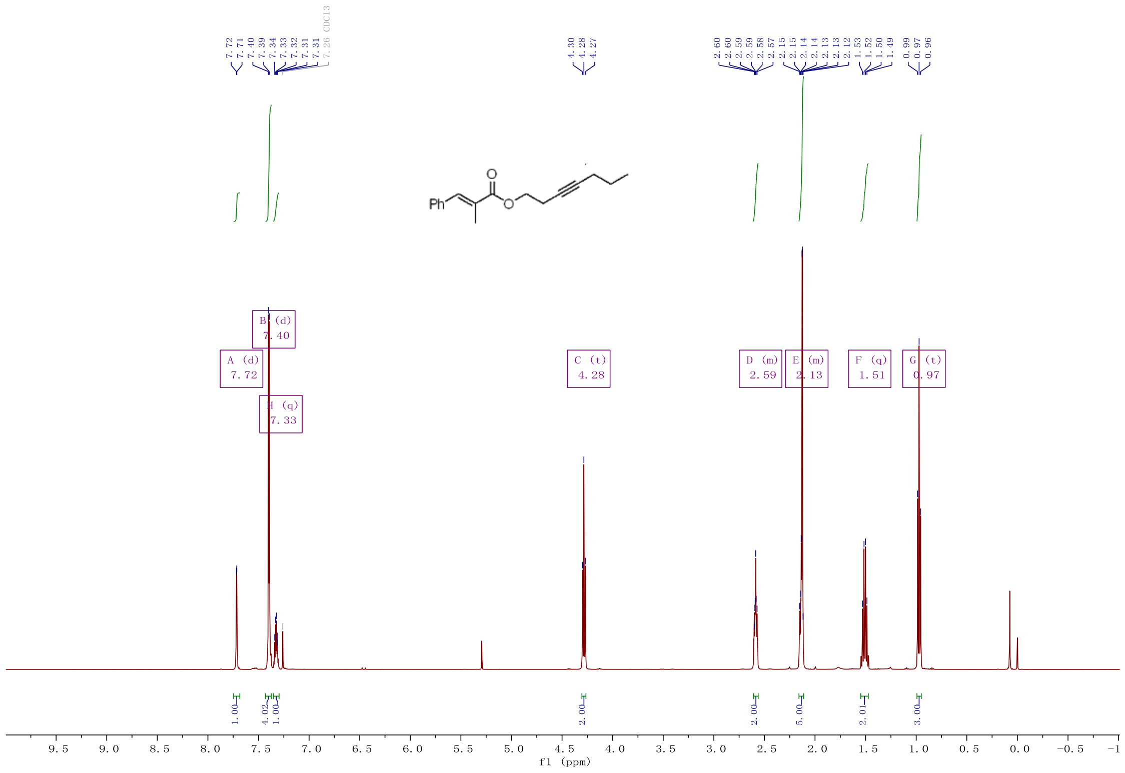

^{1}H NMR (500 MHz, 298 K, Chloroform-*d*) spectra for **S8**

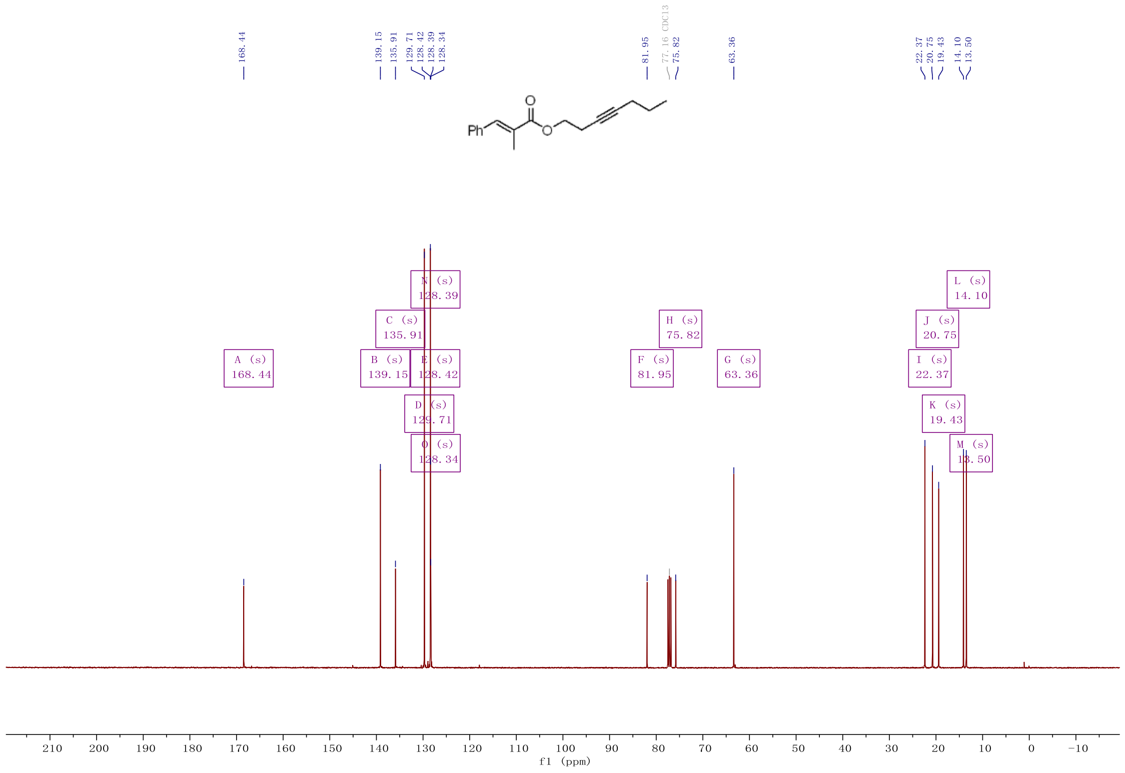

^{13}C NMR (101 MHz, 298 K, Chloroform-*d*) spectra for **S8**

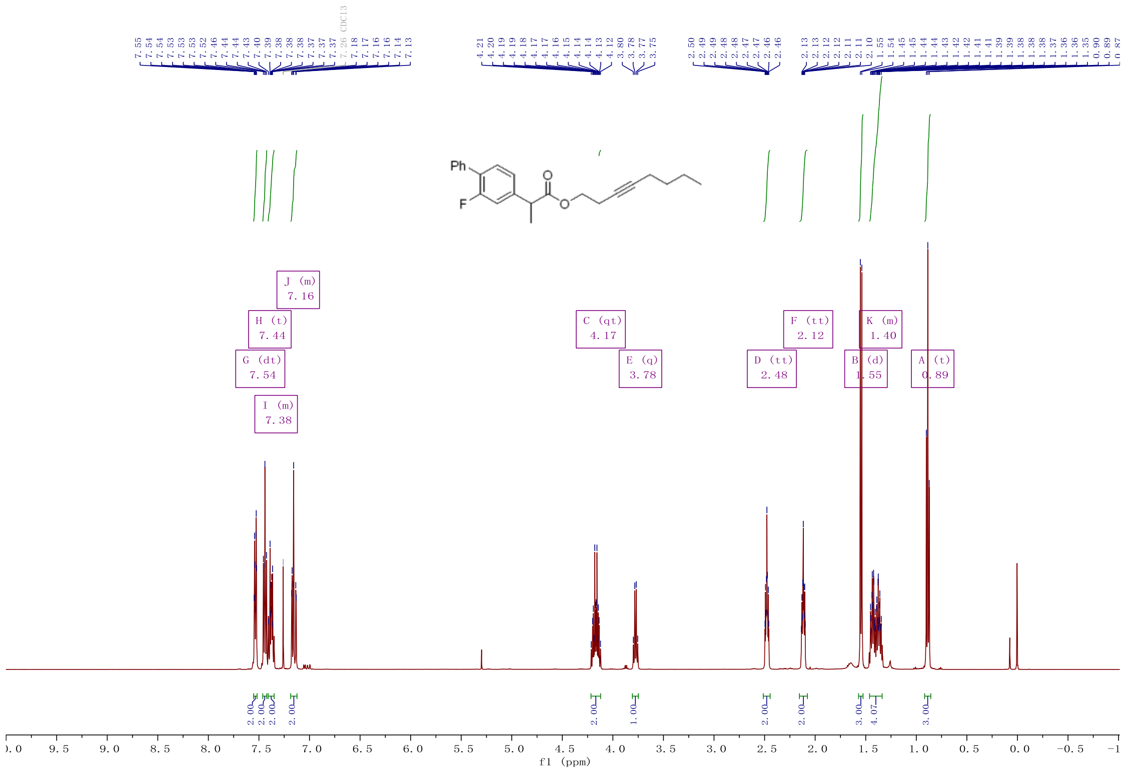

^{1}H NMR (500 MHz, 298 K, Chloroform-*d*) spectra for **S9**

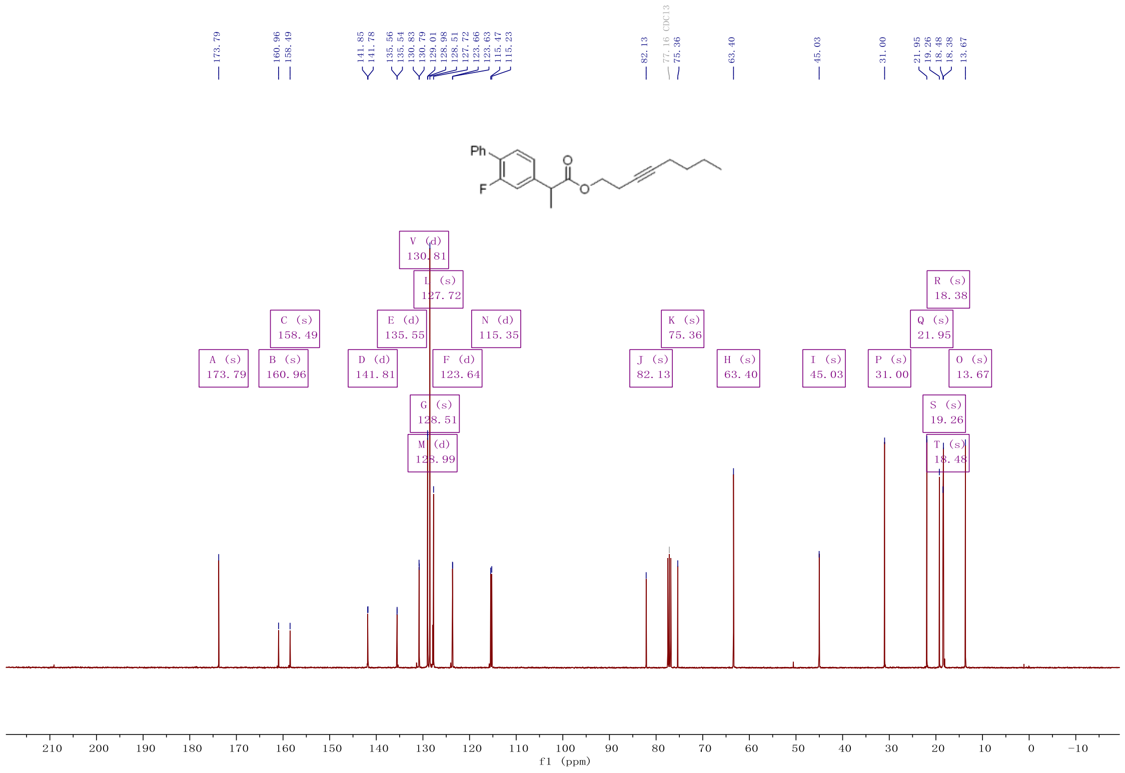

^{13}C NMR (101 MHz, 298 K, Chloroform-*d*) spectra for **S9**

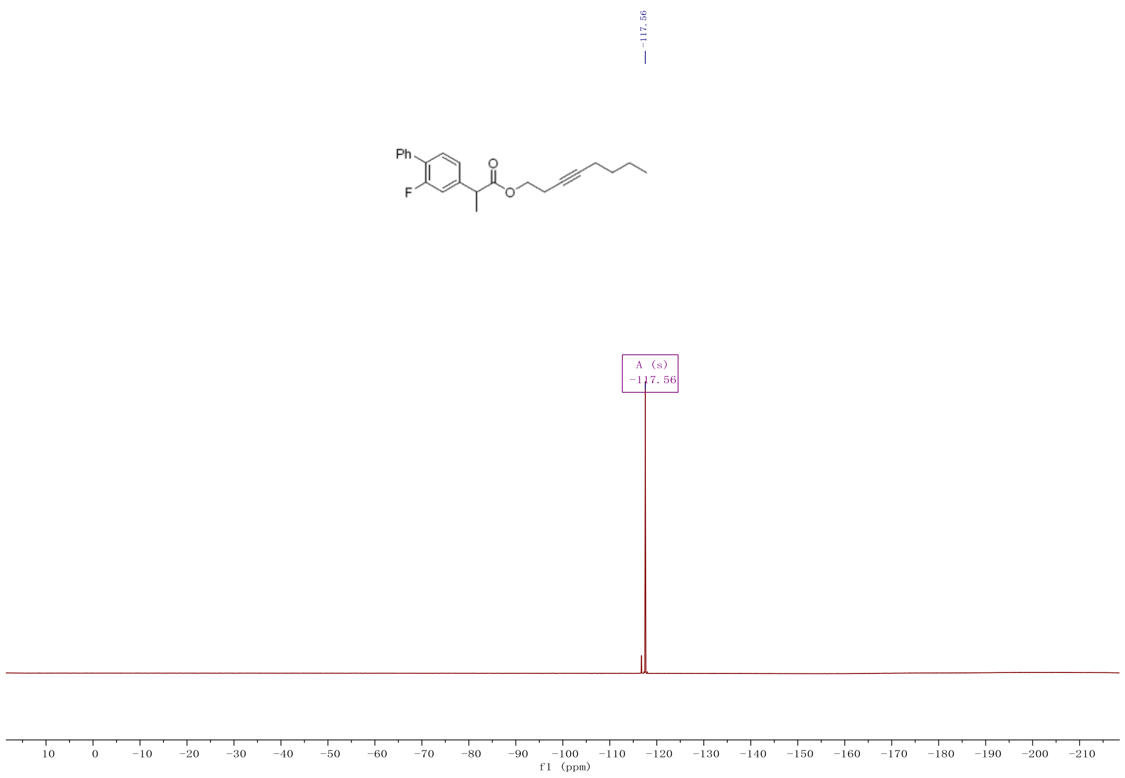

^{19}F NMR (376 MHz, 298 K, Chloroform-*d*) spectra for **S9**

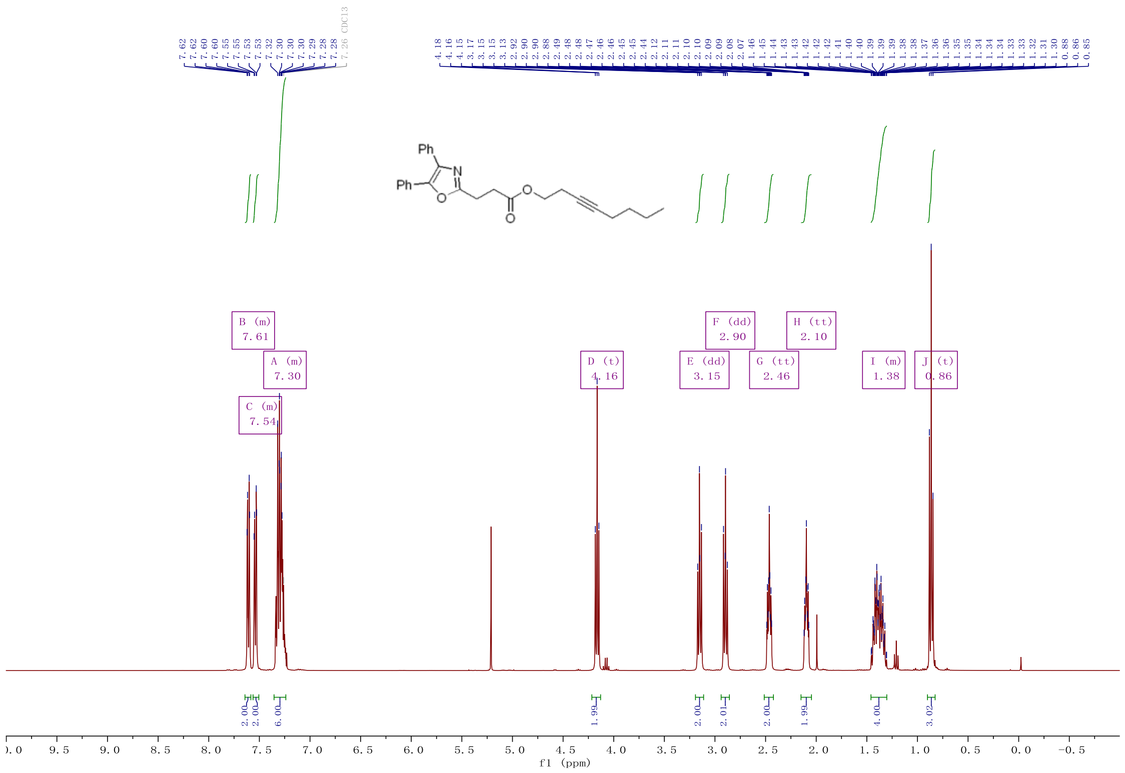

^{1}H NMR (400 MHz, 298 K, Chloroform-*d*) spectra for **S10**

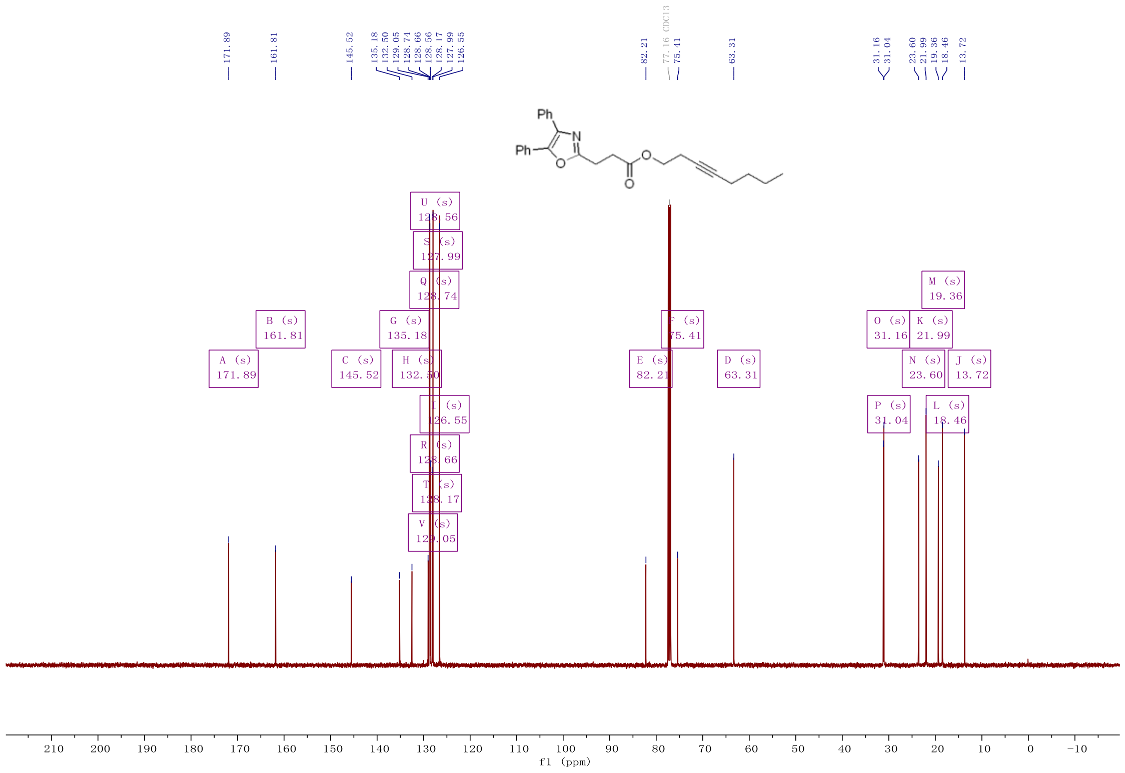

^{13}C NMR (126 MHz, 298 K, Chloroform-*d*) spectra for **S10**

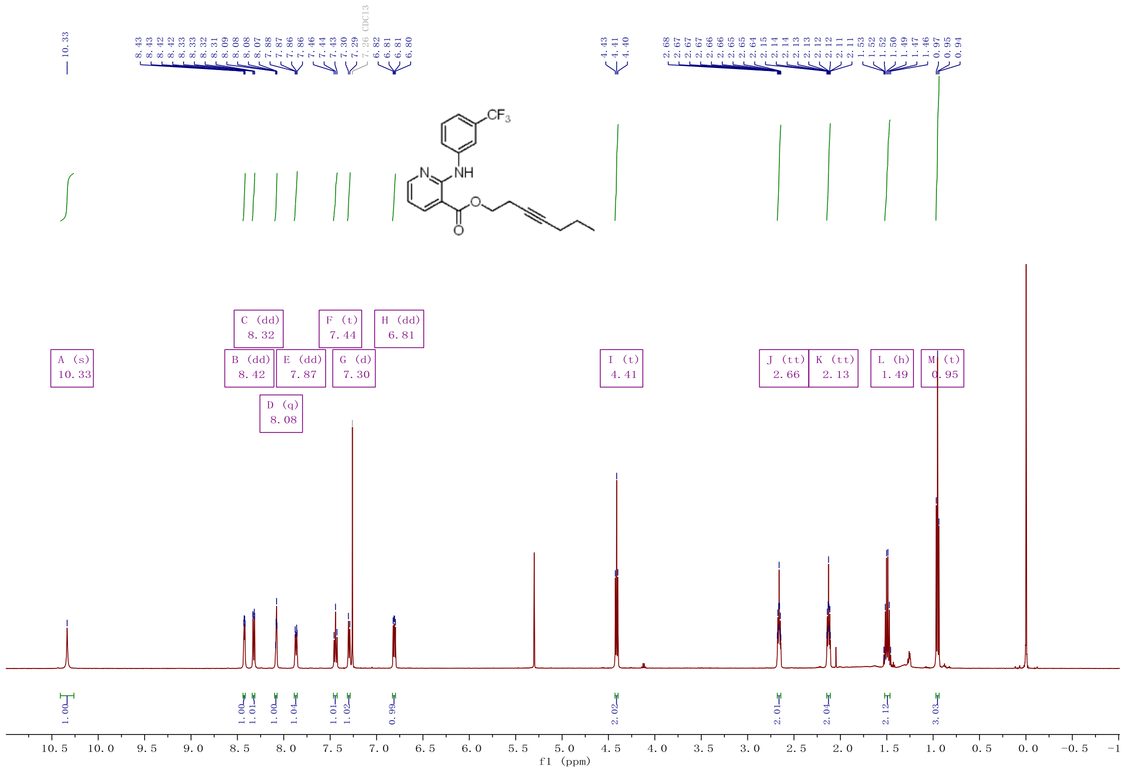

^{1}H NMR (500 MHz, 298 K, Chloroform-*d*) spectra for **S11**

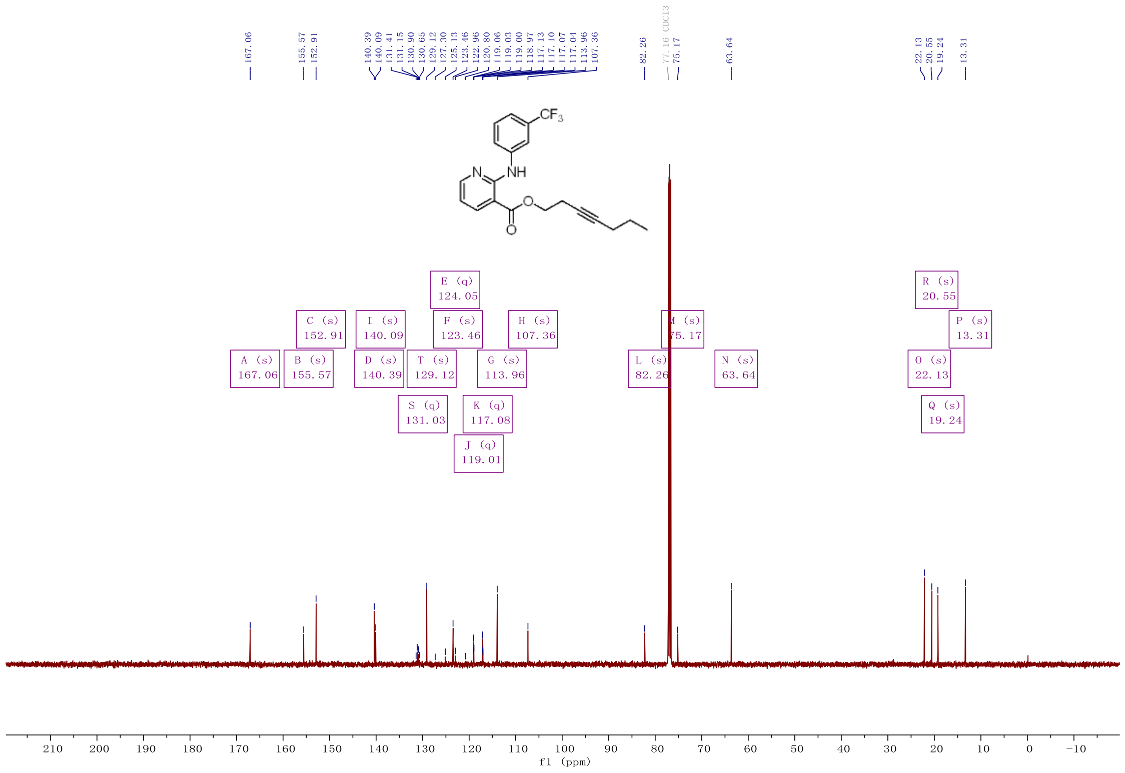

^{13}C NMR (126 MHz, 298 K, Chloroform-*d*) spectra for **S11**

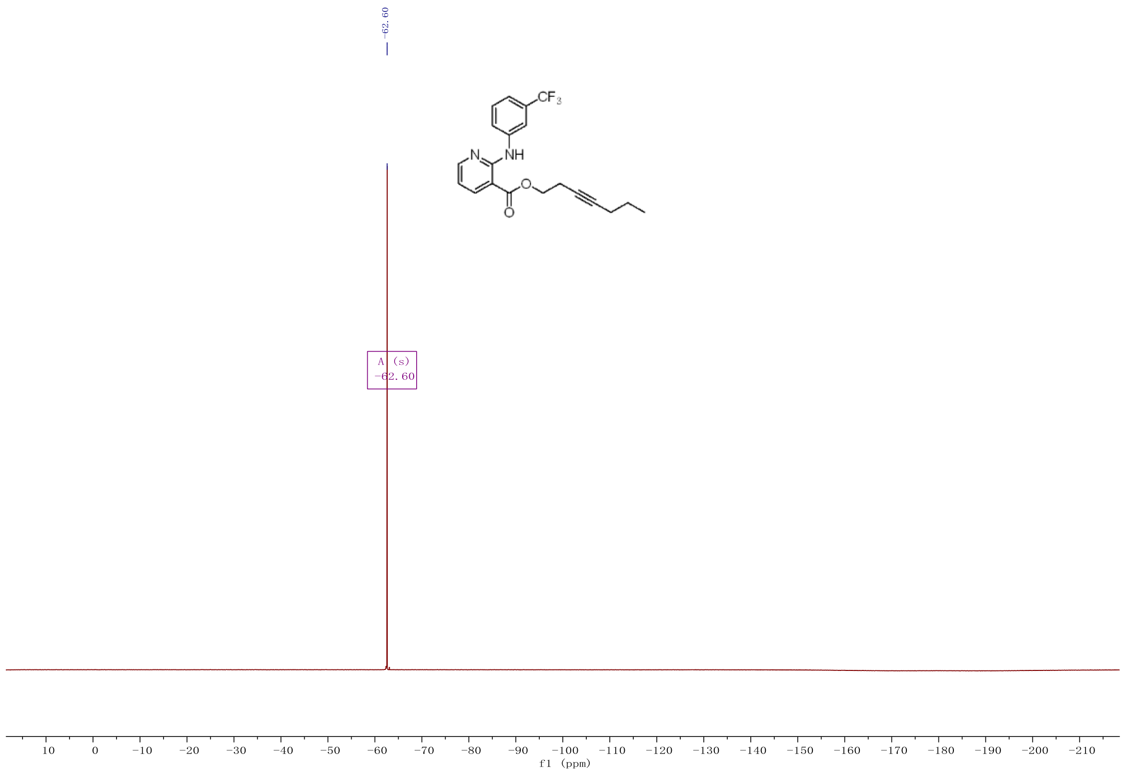

^{19}F NMR (376 MHz, 298 K, Chloroform-*d*) spectra for **S11**

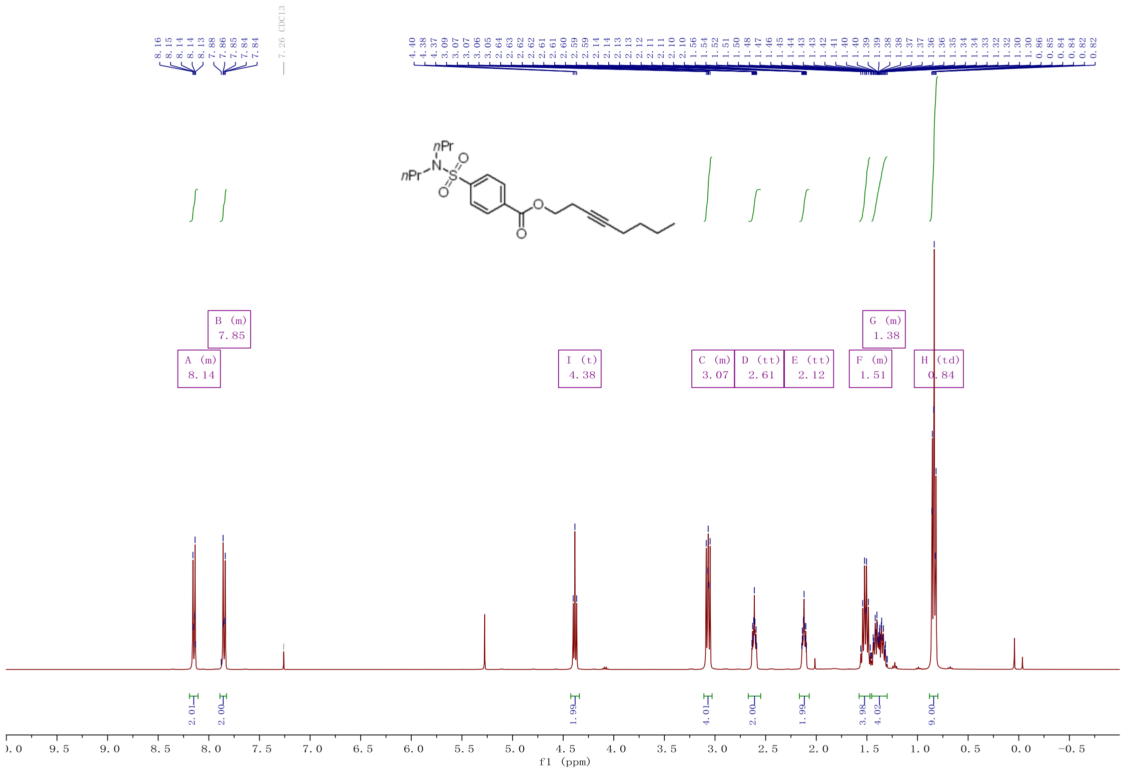

^{1}H NMR (400 MHz, 298 K, Chloroform-*d*) spectra for **S12**

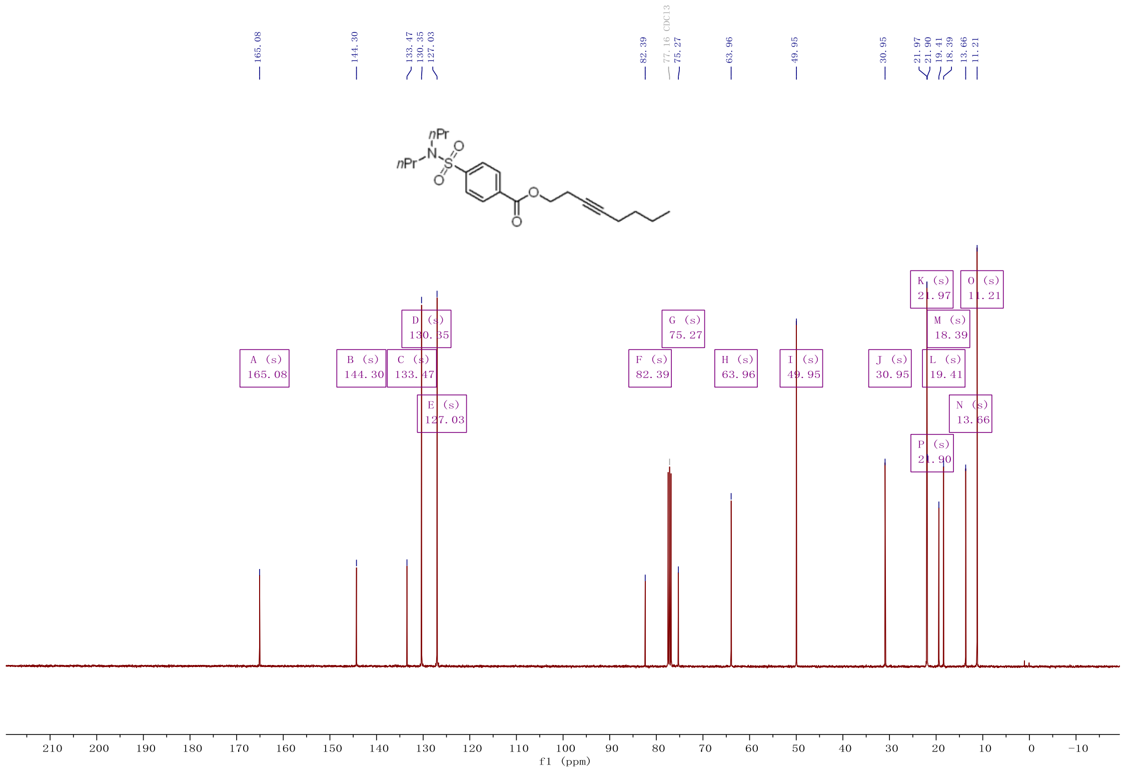

^{13}C NMR (101 MHz, 298 K, Chloroform-*d*) spectra for **S12**

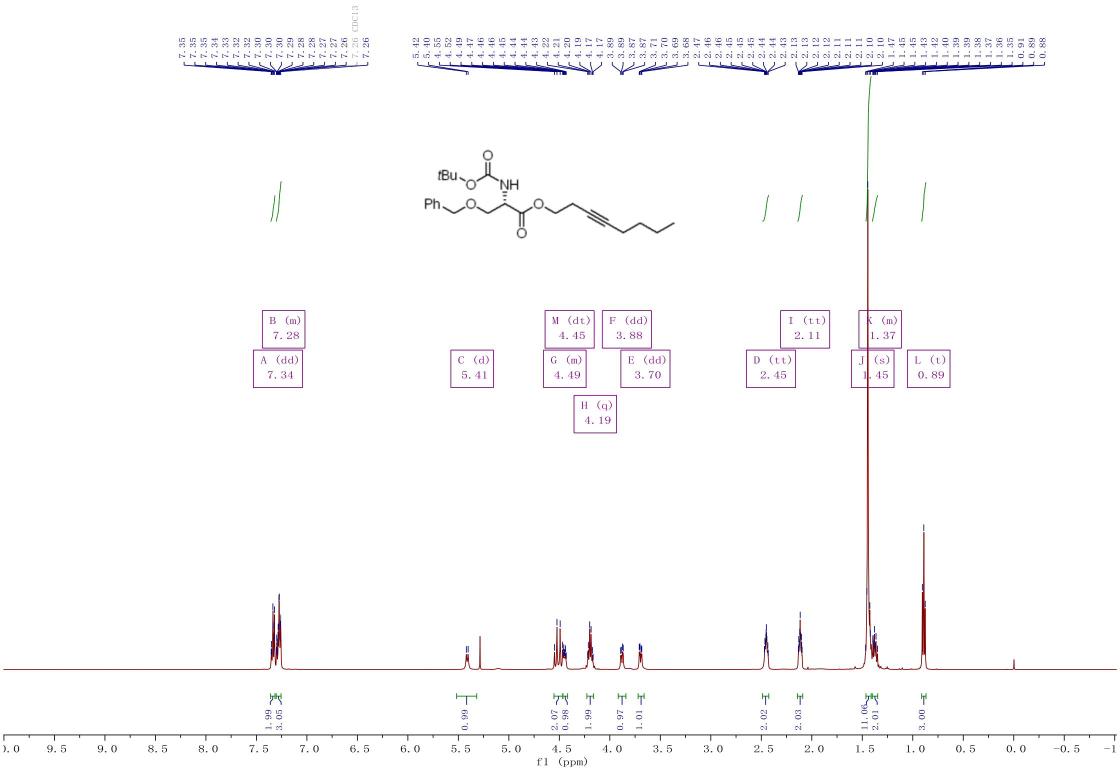

^{1}H NMR (500 MHz, Chloroform-*d*) spectra for **S13**

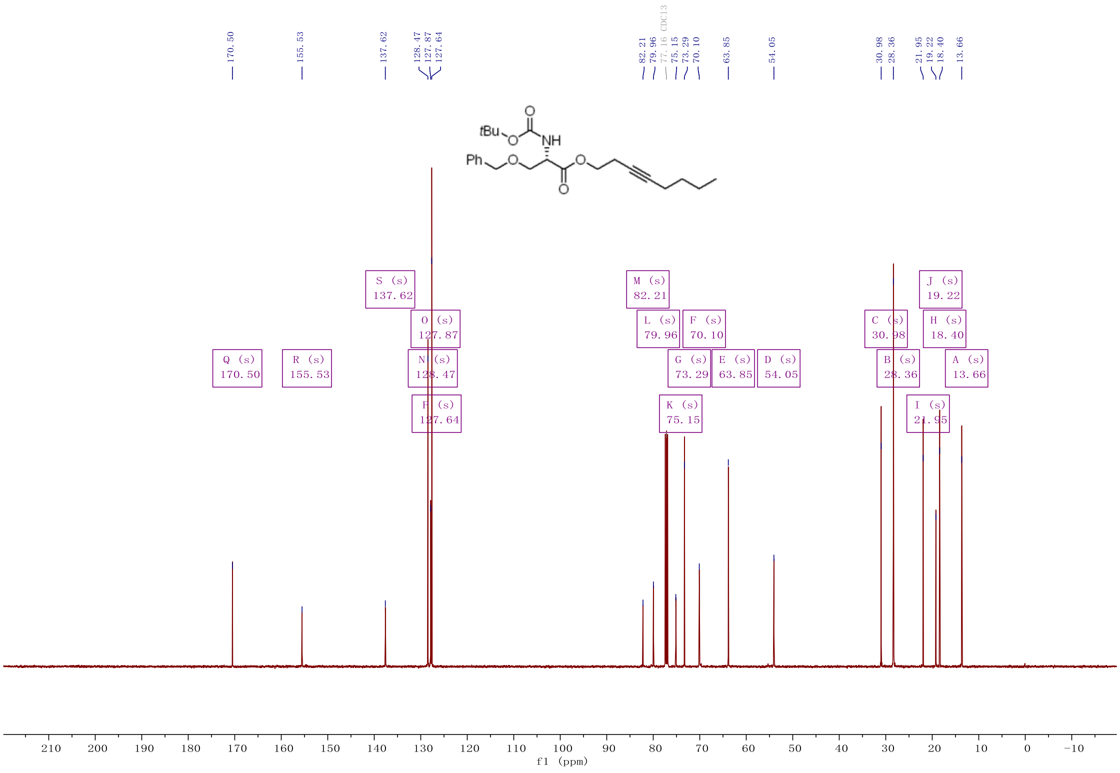

^{13}C NMR (126 MHz, 298 K, Chloroform-*d*) spectra for **S13**

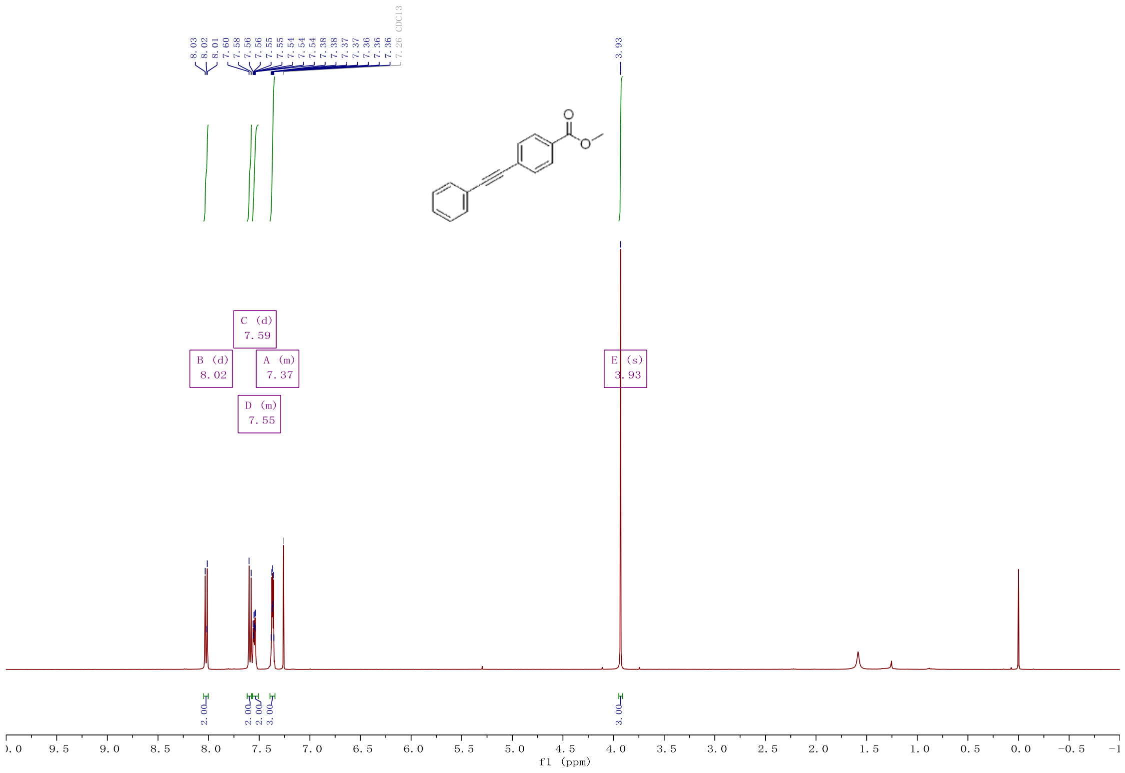

^{1}H NMR (400 MHz, 298 K, Chloroform-*d*) spectra for **S19**

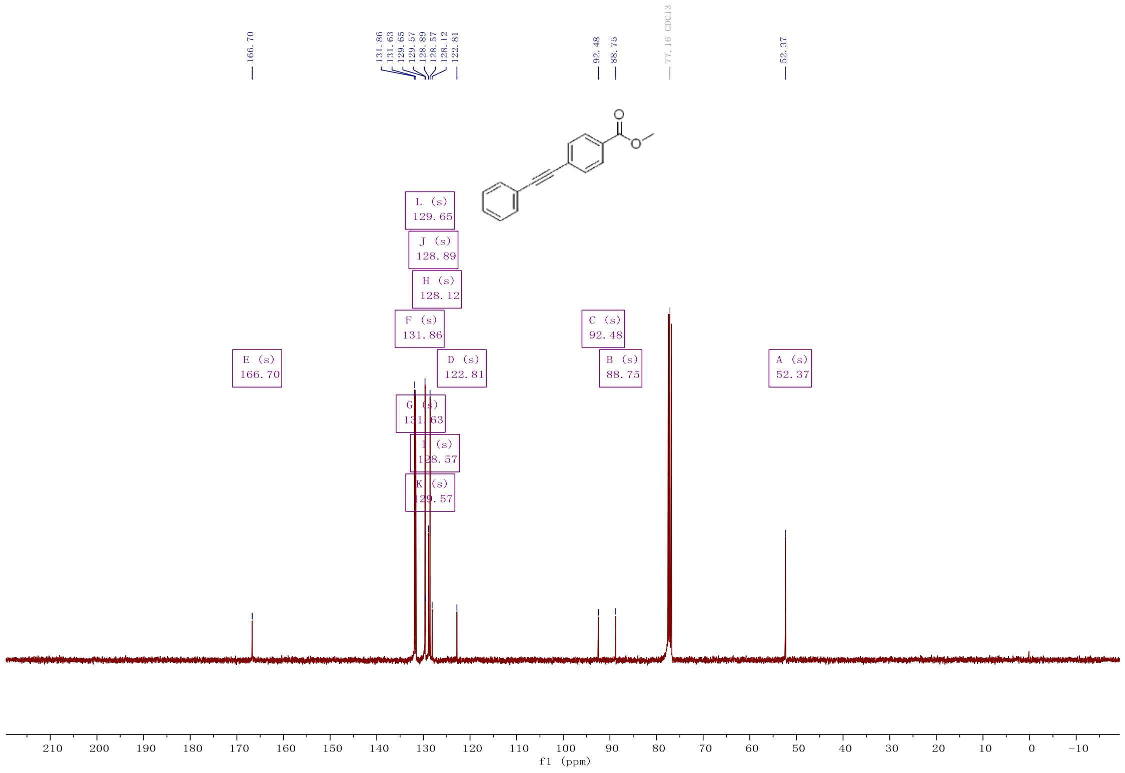

^{13}C NMR (101 MHz, 298 K, Chloroform-*d*) spectra for **S19**

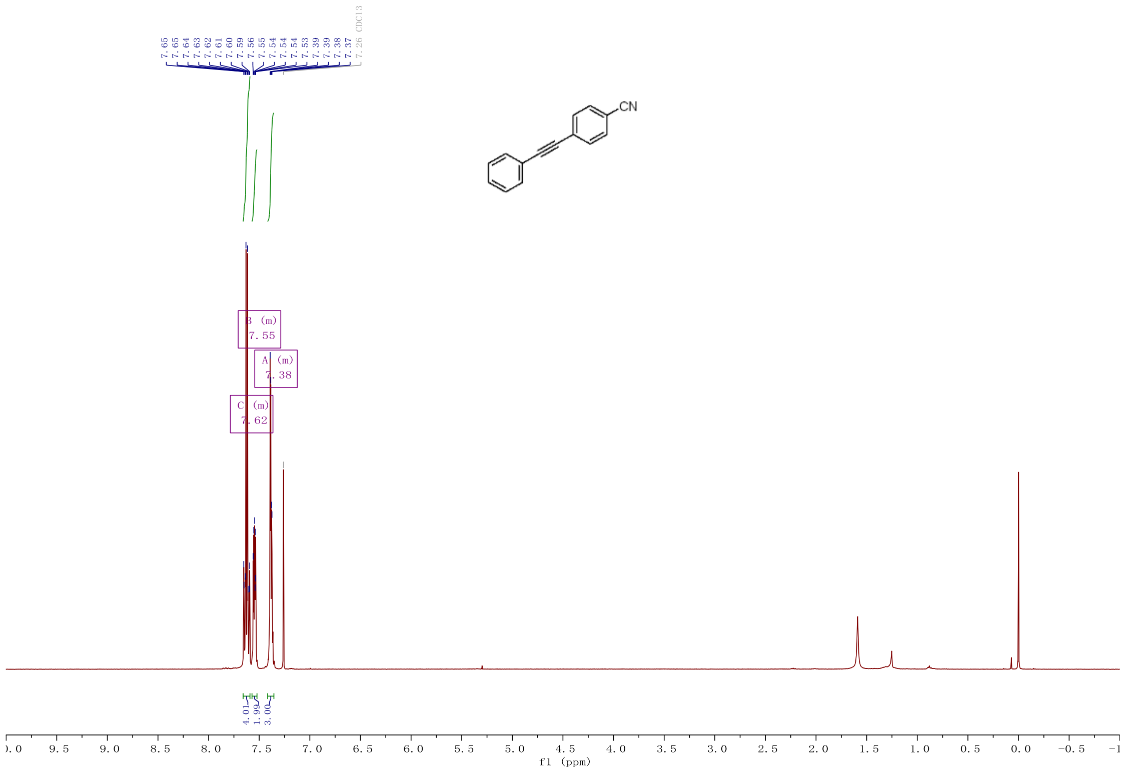

^{1}H NMR (400 MHz, 298 K, Chloroform-*d*) spectra for **S20**

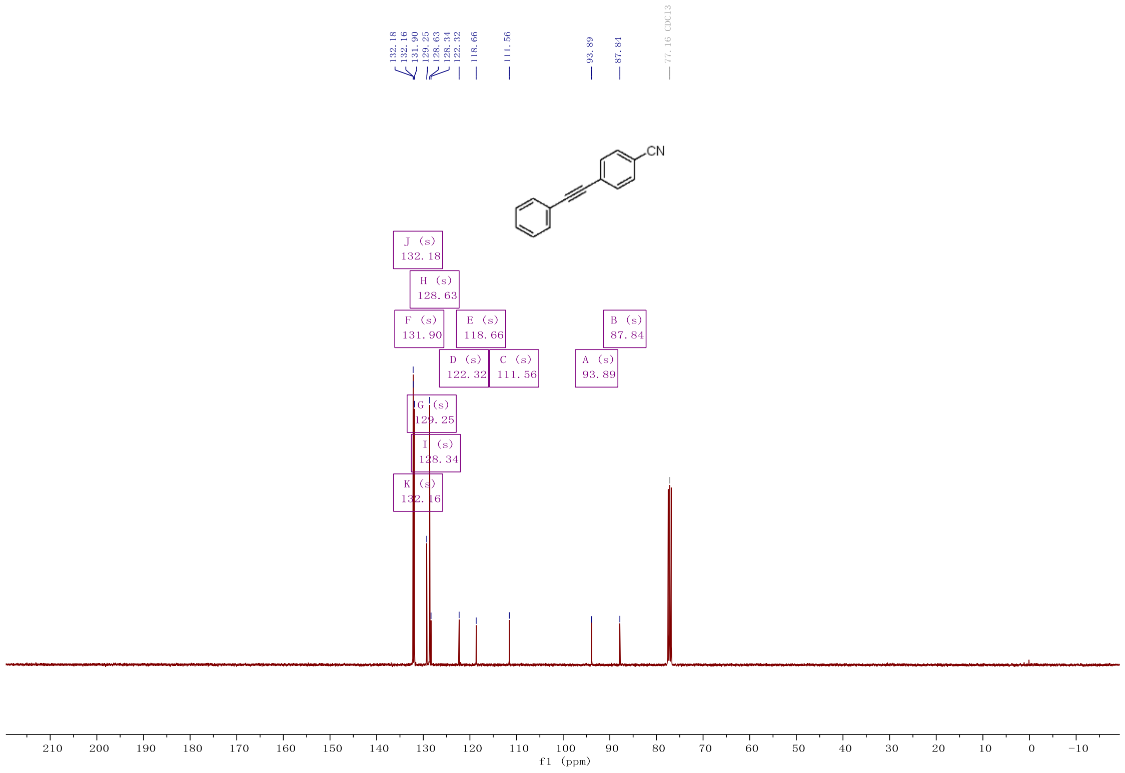

^{13}C NMR (101 MHz, 298 K, Chloroform-*d*) spectra for **S20**

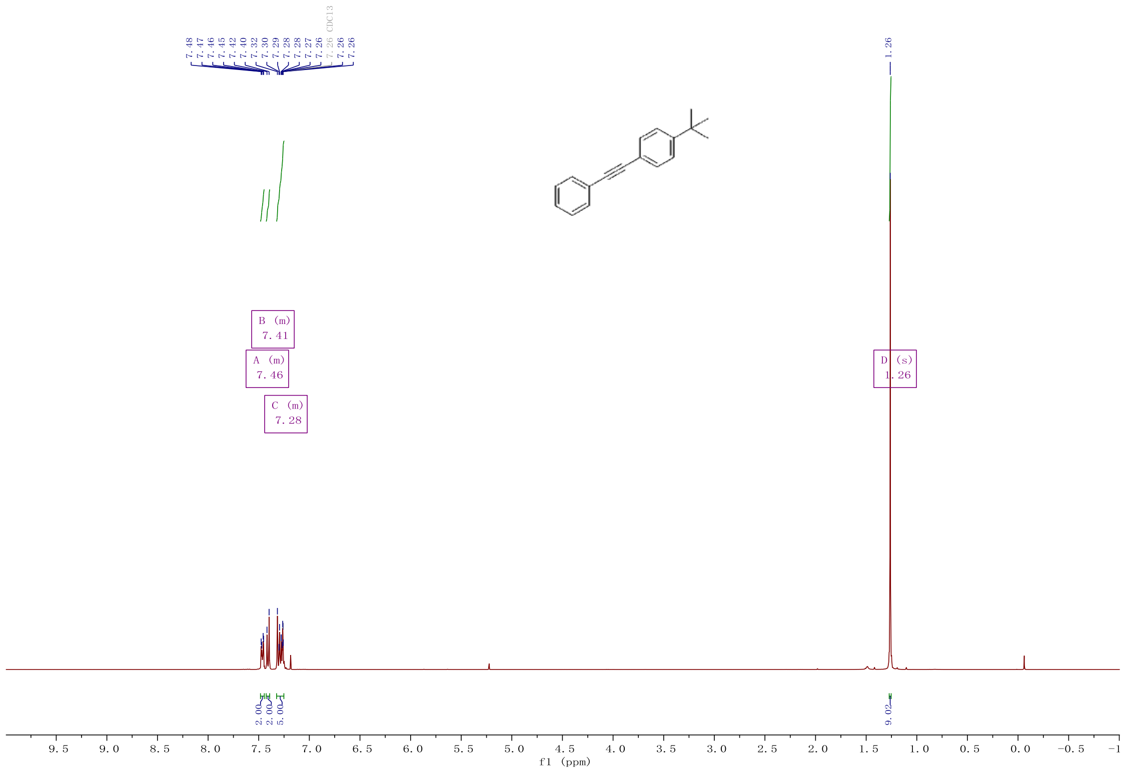

^{1}H NMR (400 MHz, 298 K, Chloroform-*d*) spectra for **S21**

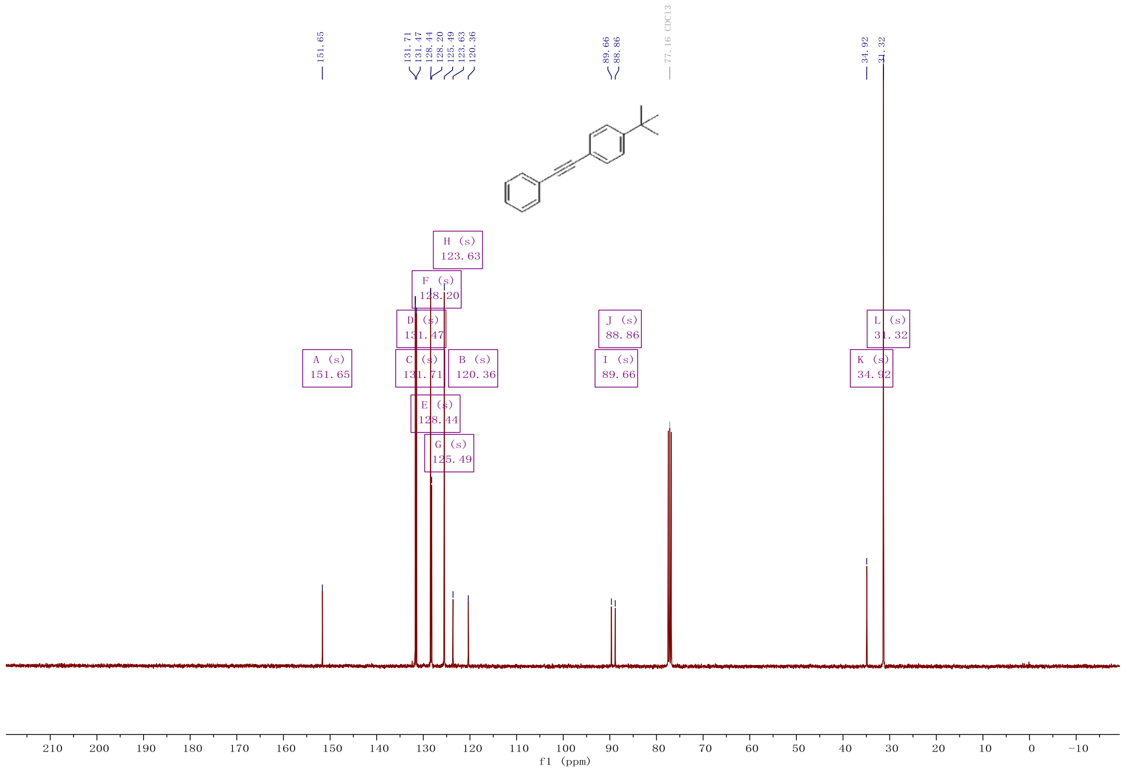

^{13}C NMR (101 MHz, 298 K, Chloroform-*d*) spectra for **S21**

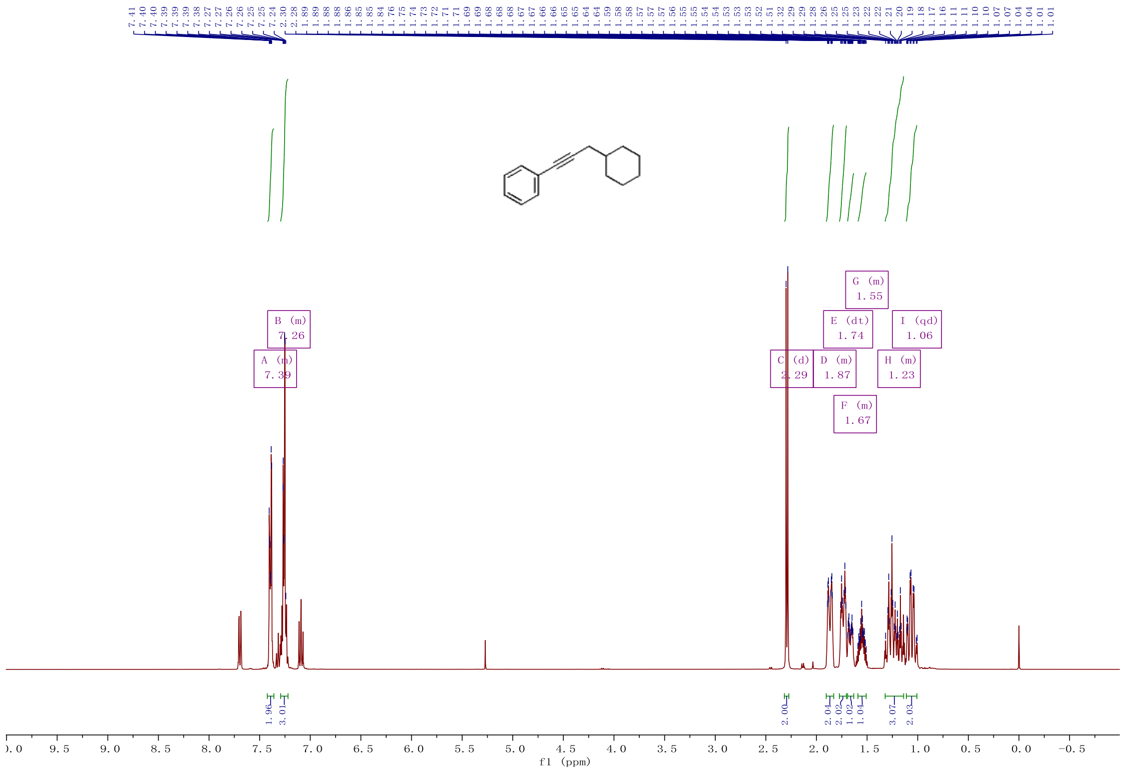

^{1}H NMR (400 MHz, 298 K, Chloroform-*d*) spectra for **S23**

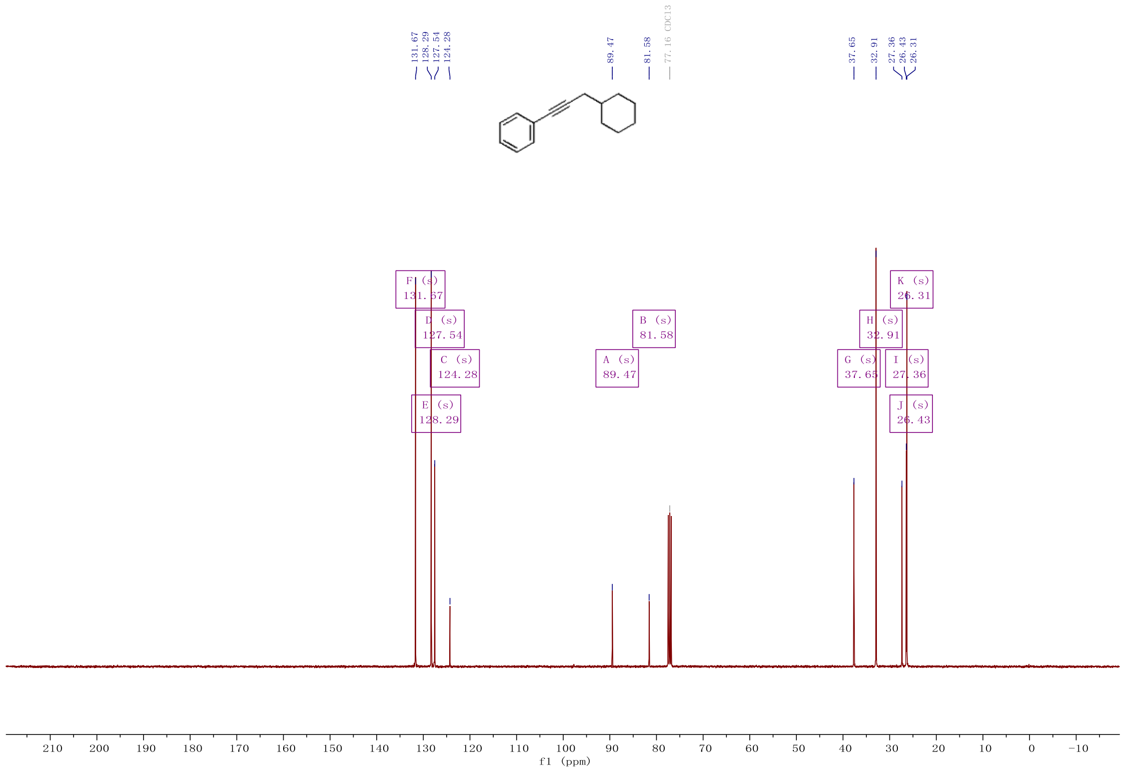

^{13}C NMR (101 MHz, 298 K, Chloroform-*d*) spectra for **S23**

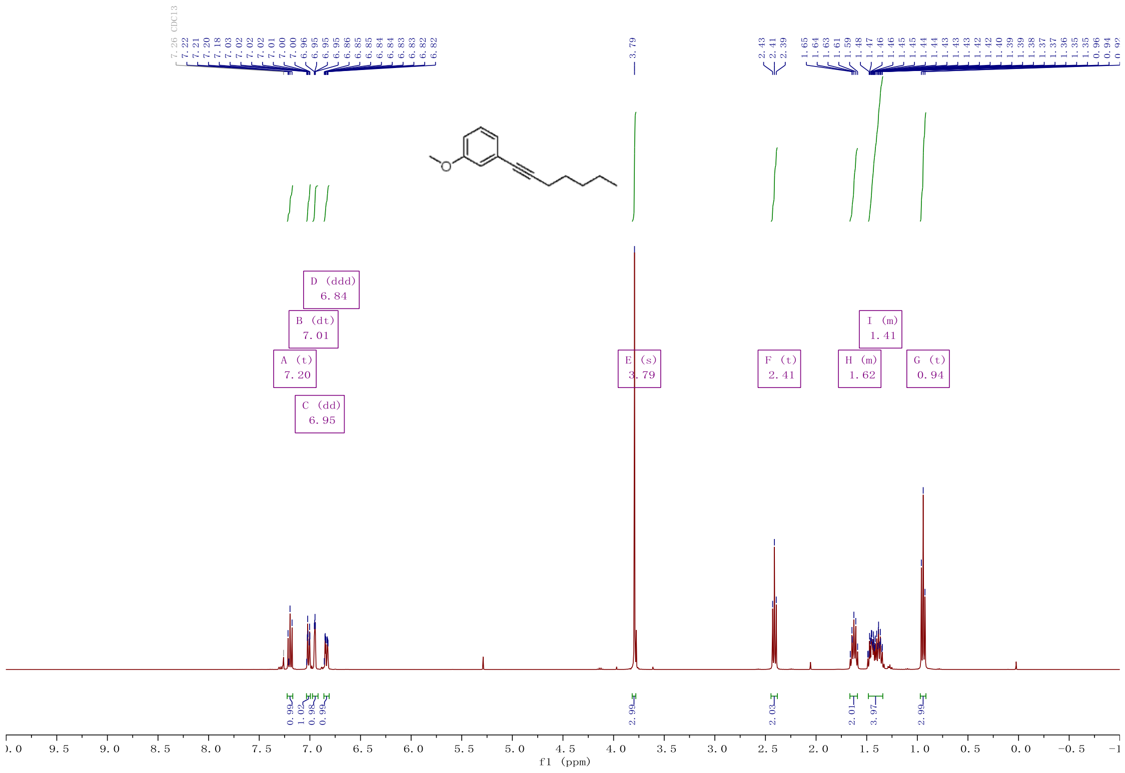

^{1}H NMR (400 MHz, 298 K, Chloroform-*d*) spectra for **S24**

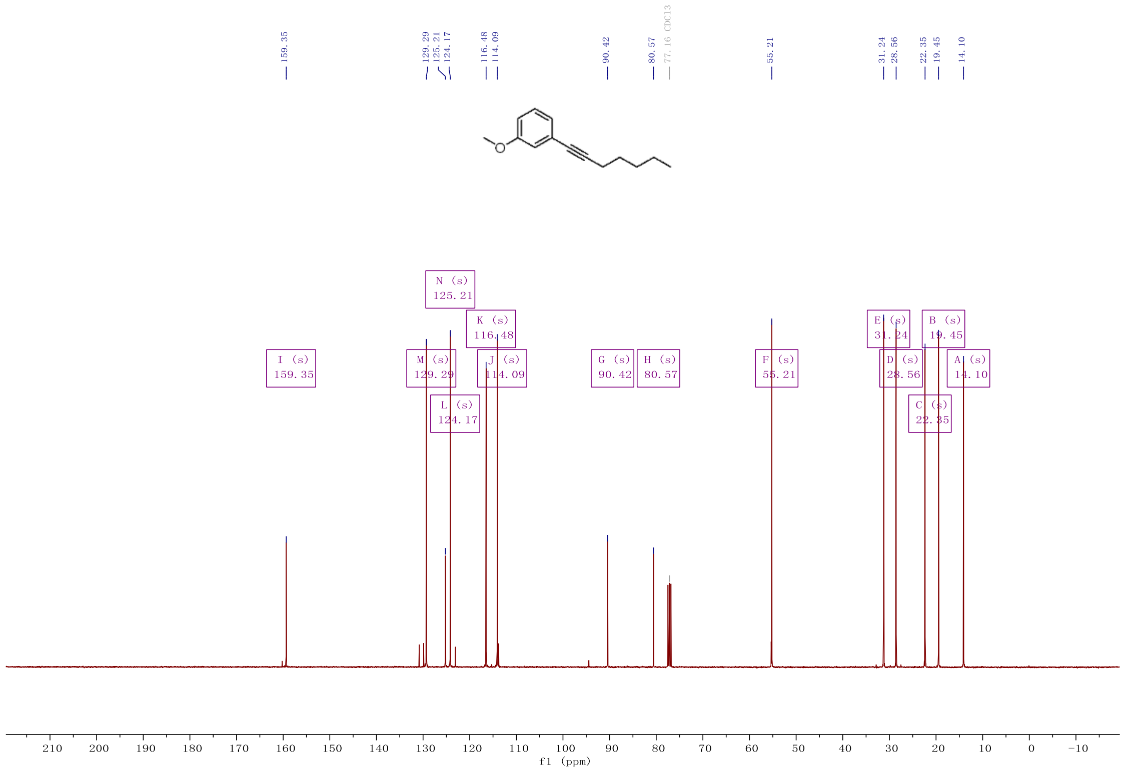

^{13}C NMR (101 MHz, 298 K, Chloroform-*d*) spectra for **S24**

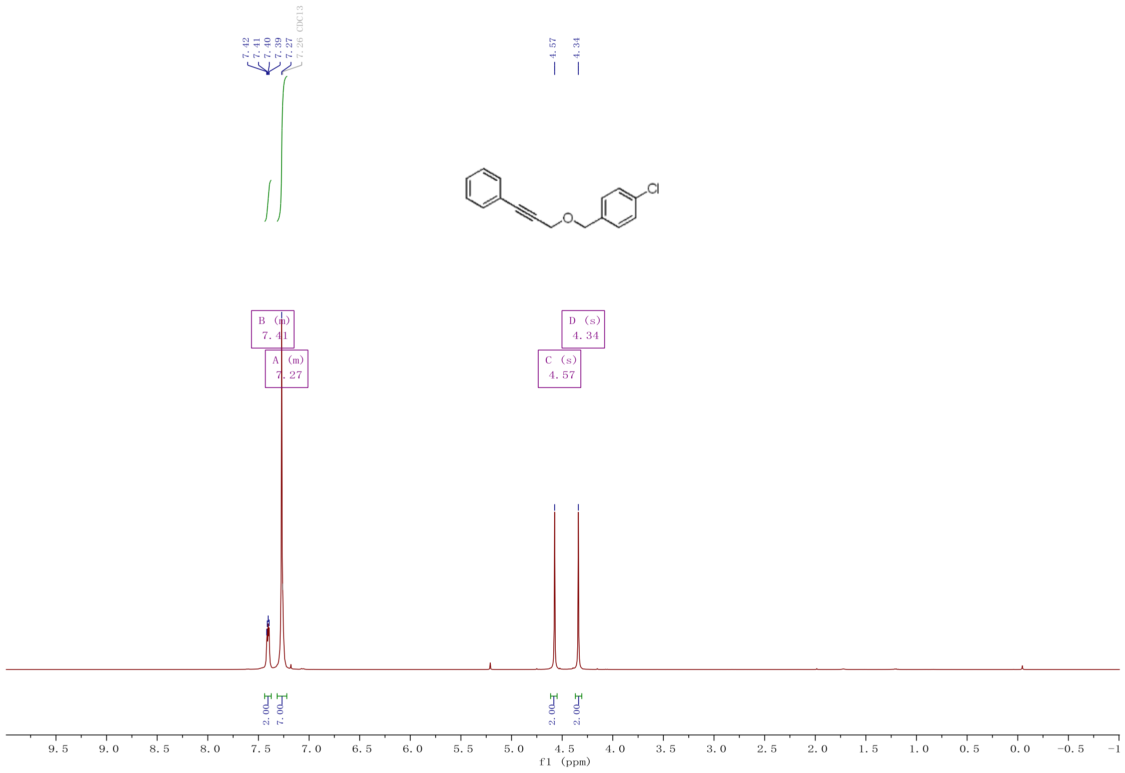

^{1}H NMR (400 MHz, 298 K, Chloroform-*d*) spectra for **S25**

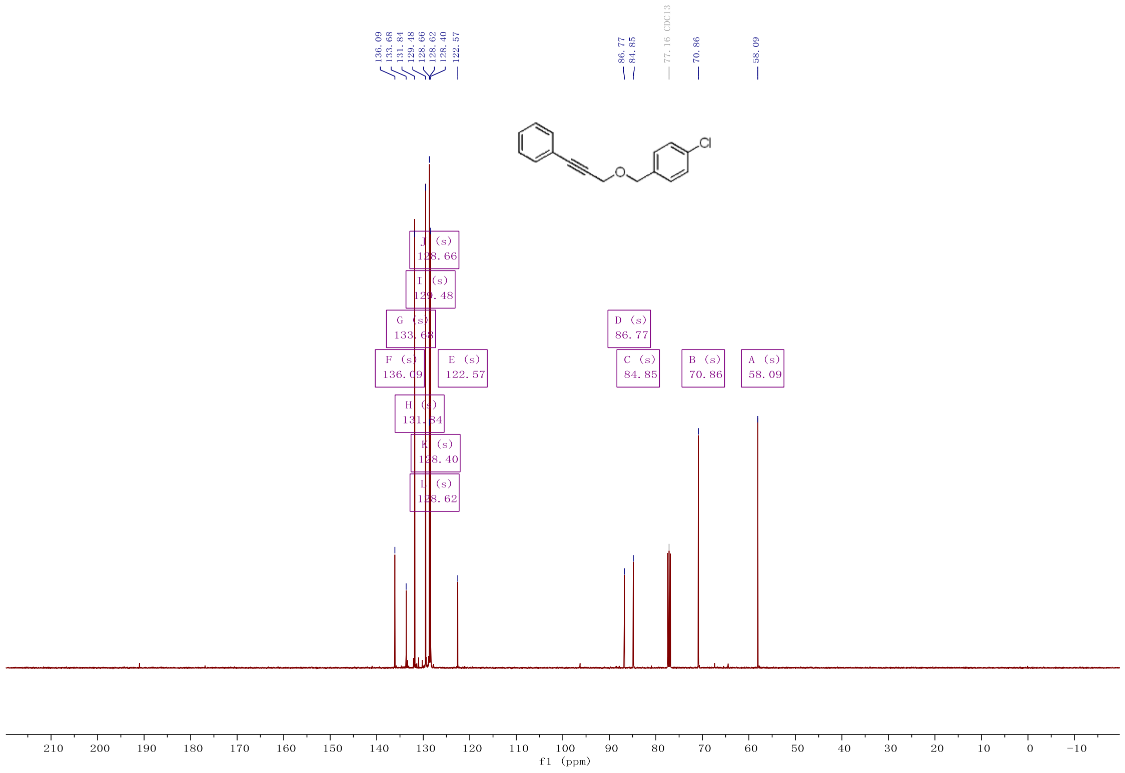

^{13}C NMR (126 MHz, 298 K, Chloroform-*d*) spectra for **S25**

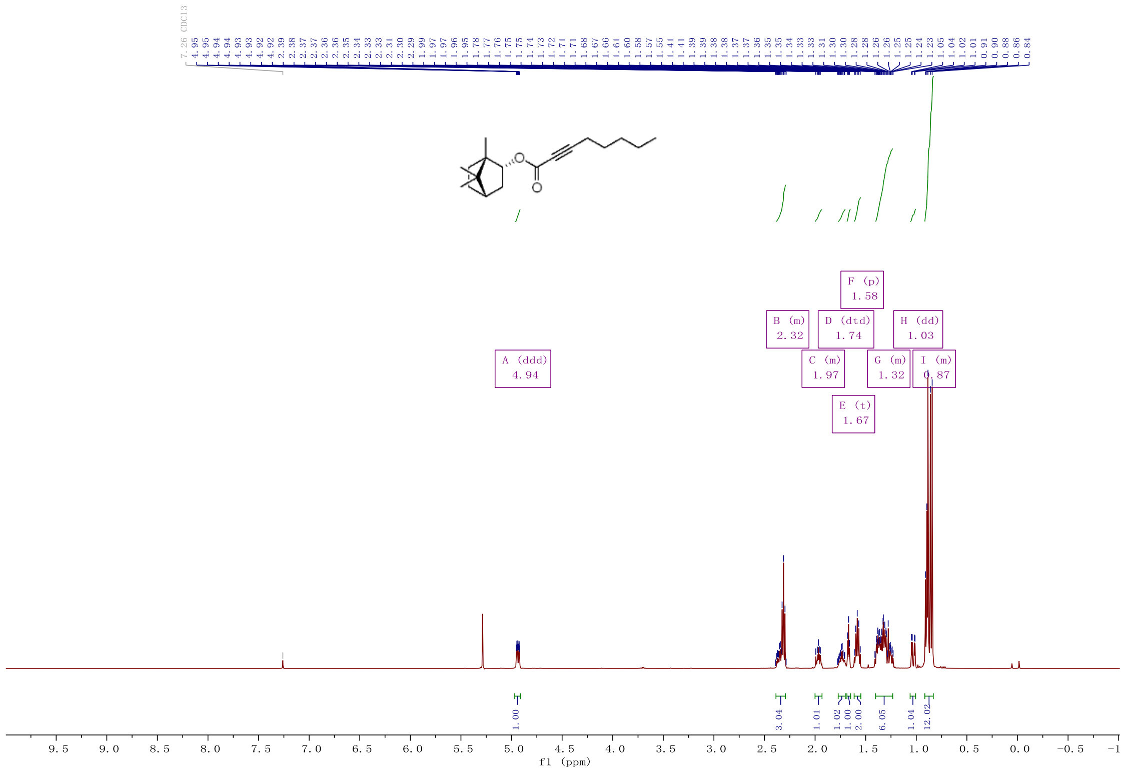

^{1}H NMR (500 MHz, 298 K, Chloroform-*d*) spectra for **S27**

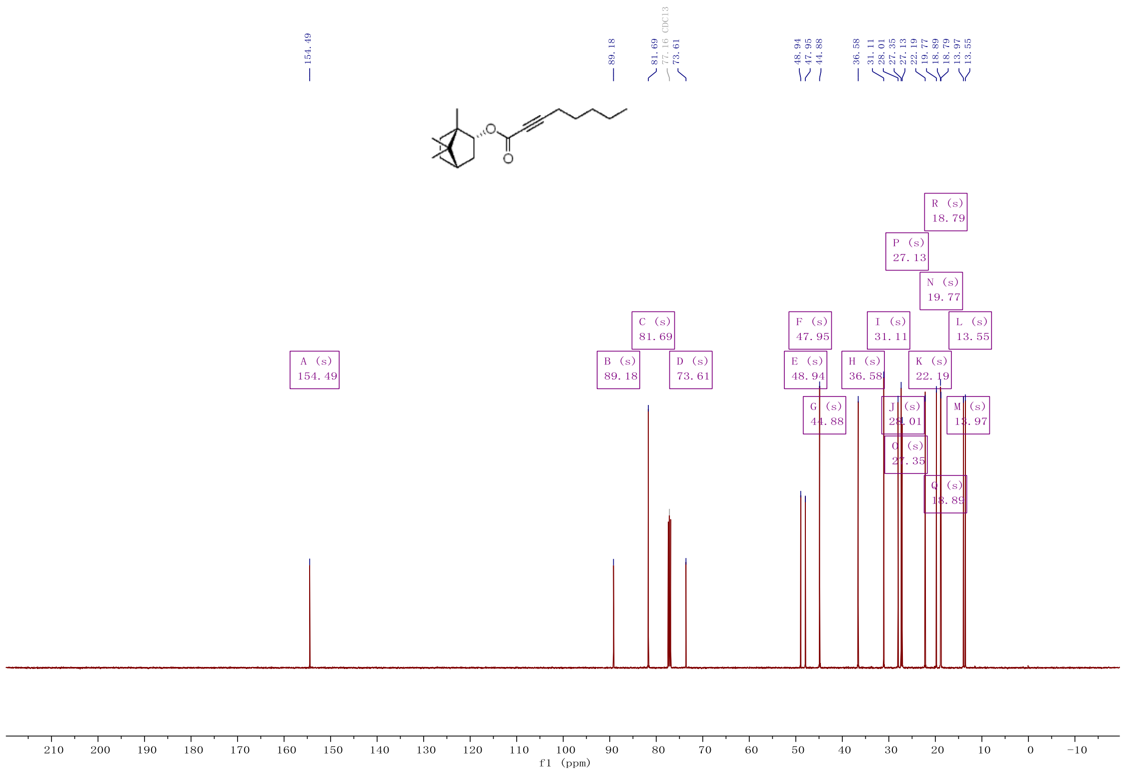

^{13}C NMR (126 MHz, 298 K, Chloroform-*d*) spectra for **S27**

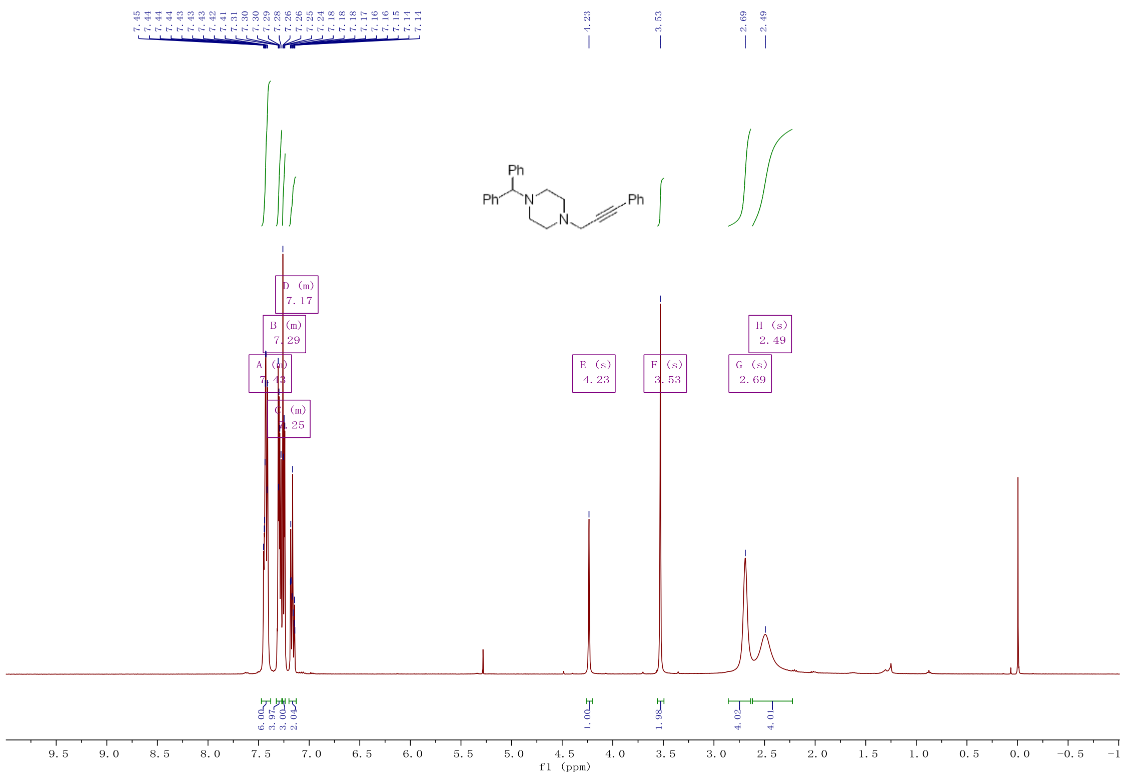

^{1}H NMR (400 MHz, 298 K, Chloroform-*d*) spectra for **31**

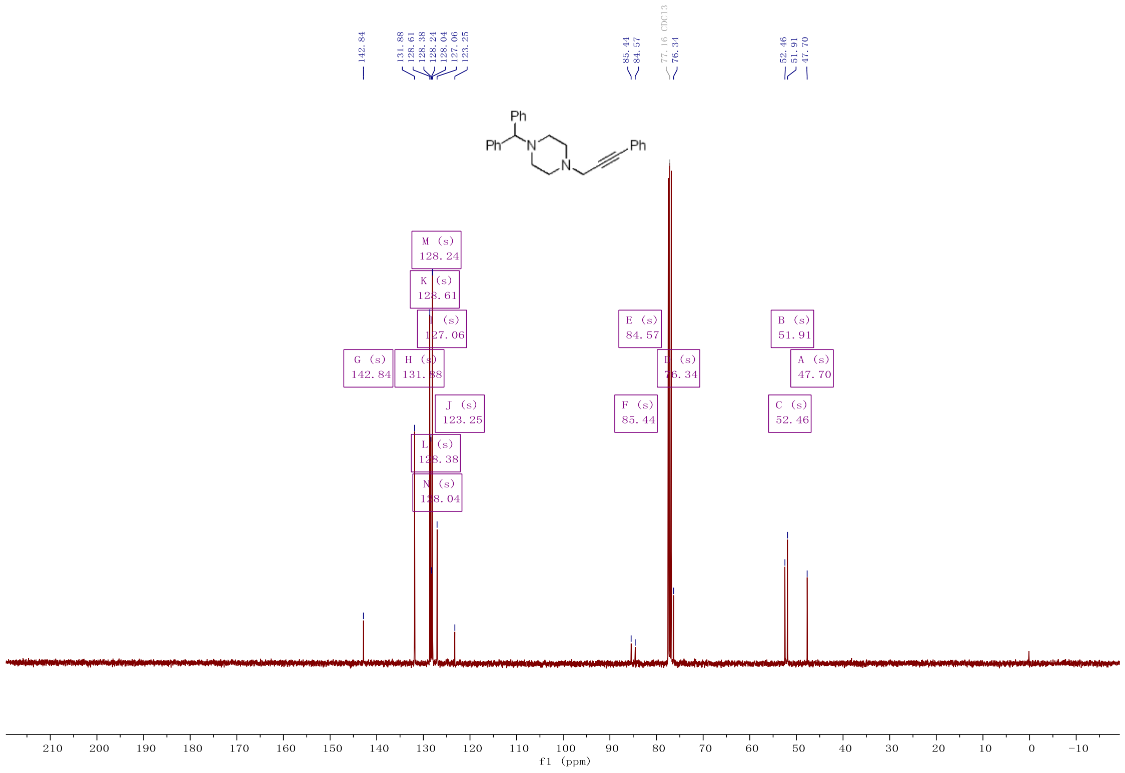

^{13}C NMR (101 MHz, 298 K, Chloroform-*d*) spectra for **31**

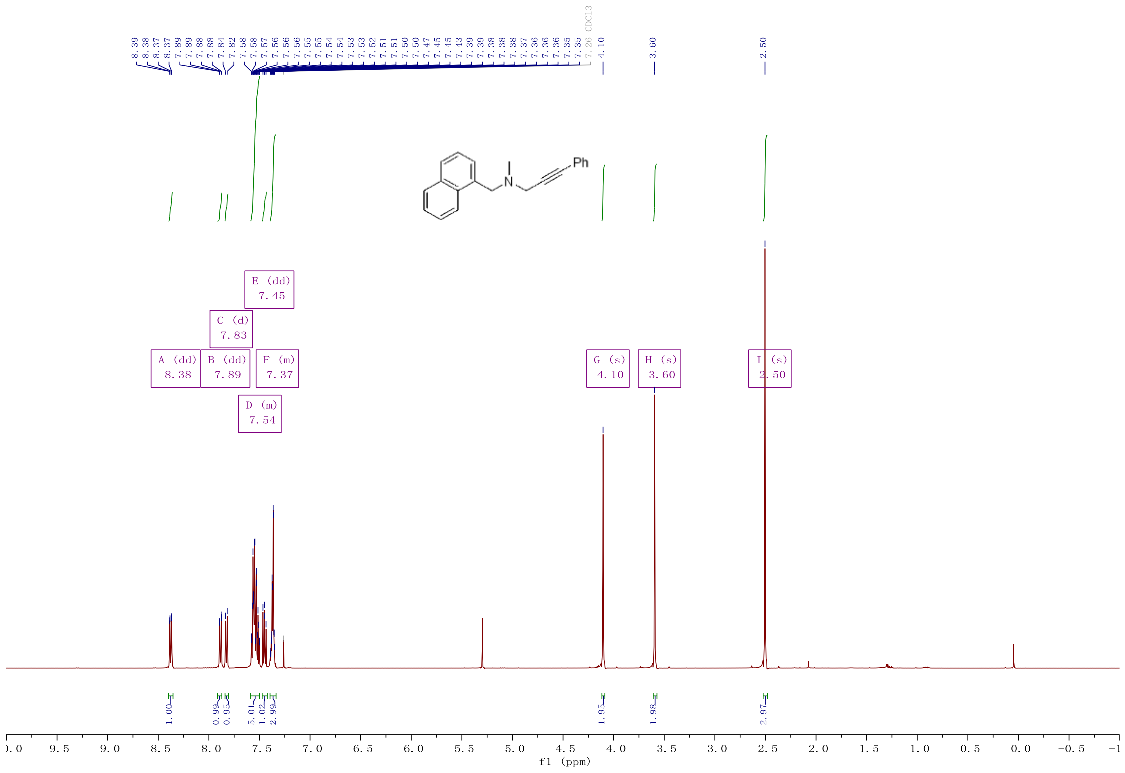

^{1}H NMR (500 MHz, 298 K, Chloroform-*d*) spectra for **33**

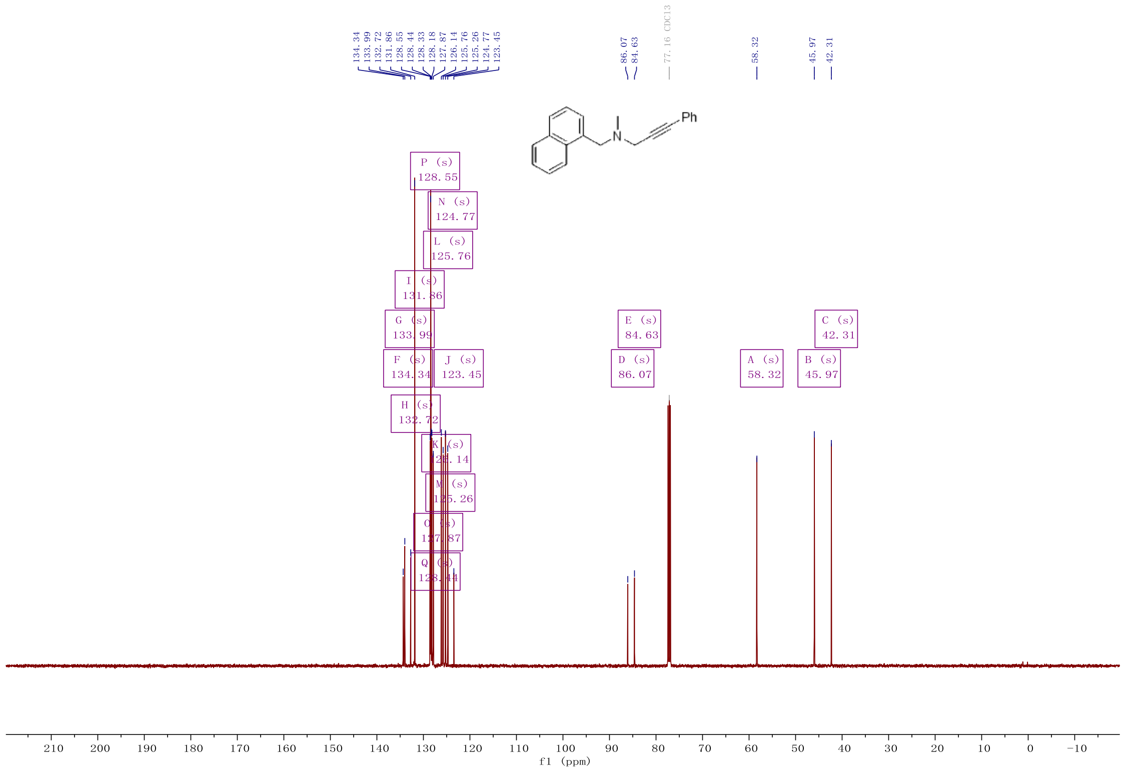

^{13}C NMR (126 MHz, 298 K, Chloroform-*d*) spectra for **33**

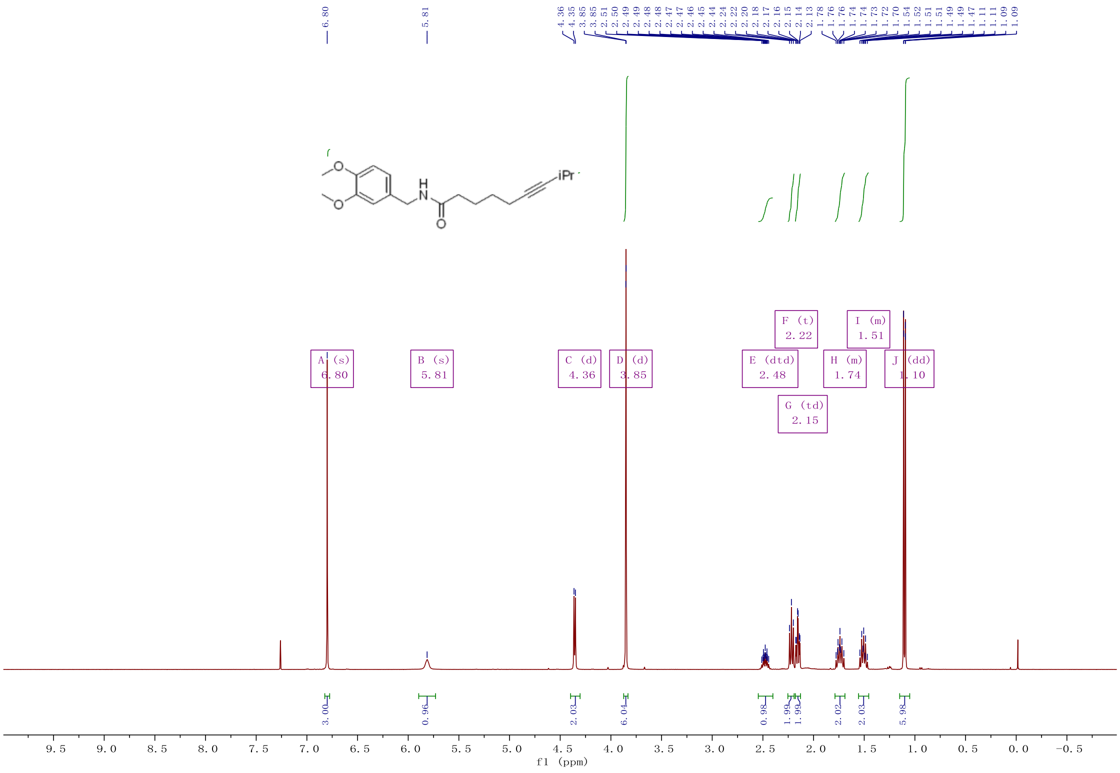

^{1}H NMR (400 MHz, 298 K, Chloroform-*d*) spectra for **35**

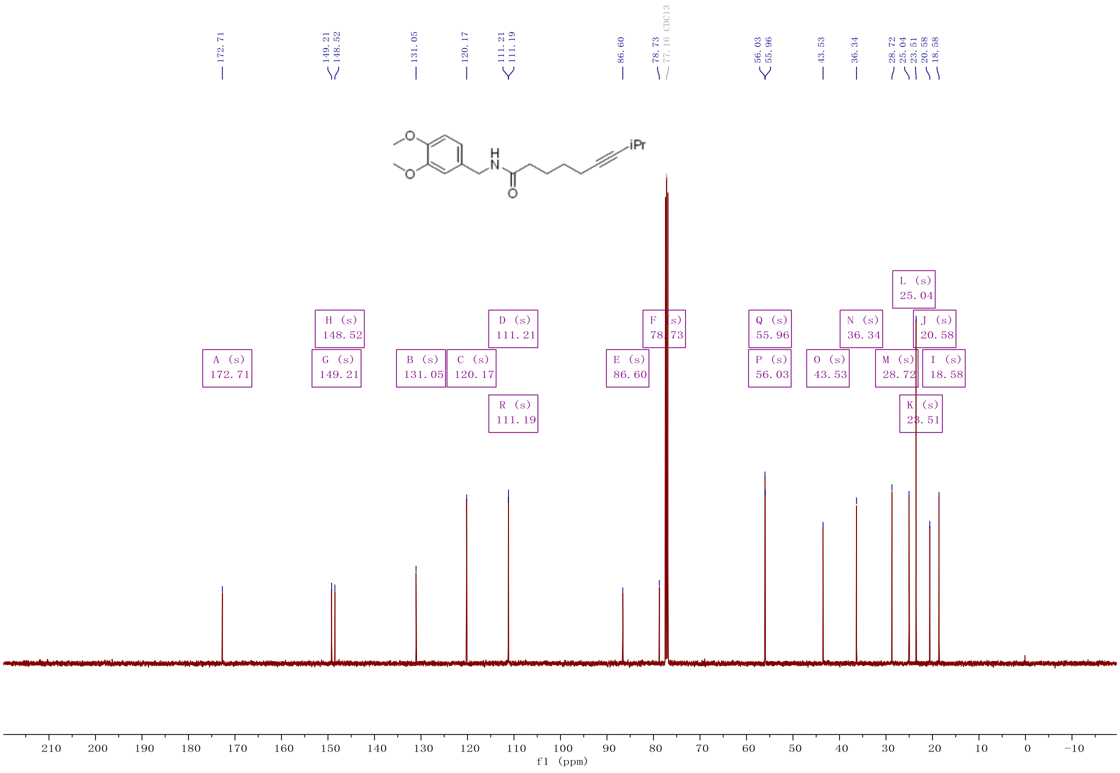

^{13}C NMR (126 MHz, 298 K, Chloroform-*d*) spectra for **35**

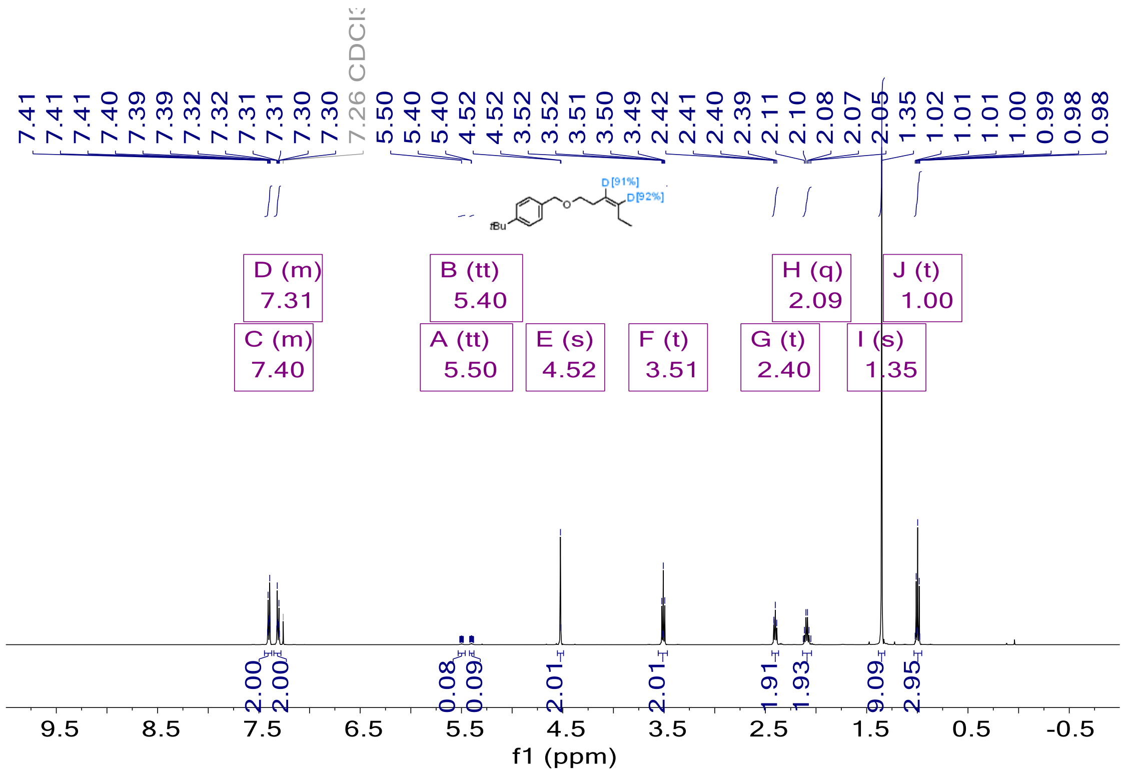

^{1}H NMR (500 MHz, 298 K, Chloroform-*d*) spectra for **2**

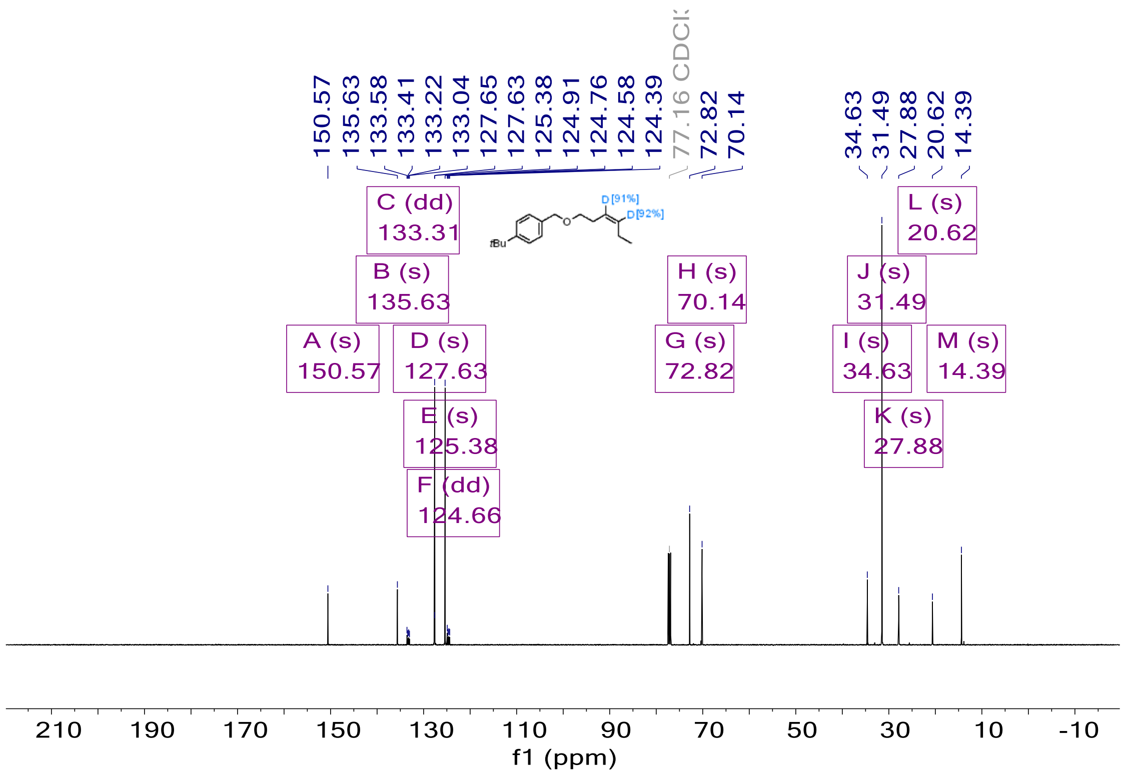

^{13}C NMR (126 MHz, 298 K, Chloroform-*d*) spectra for **2**

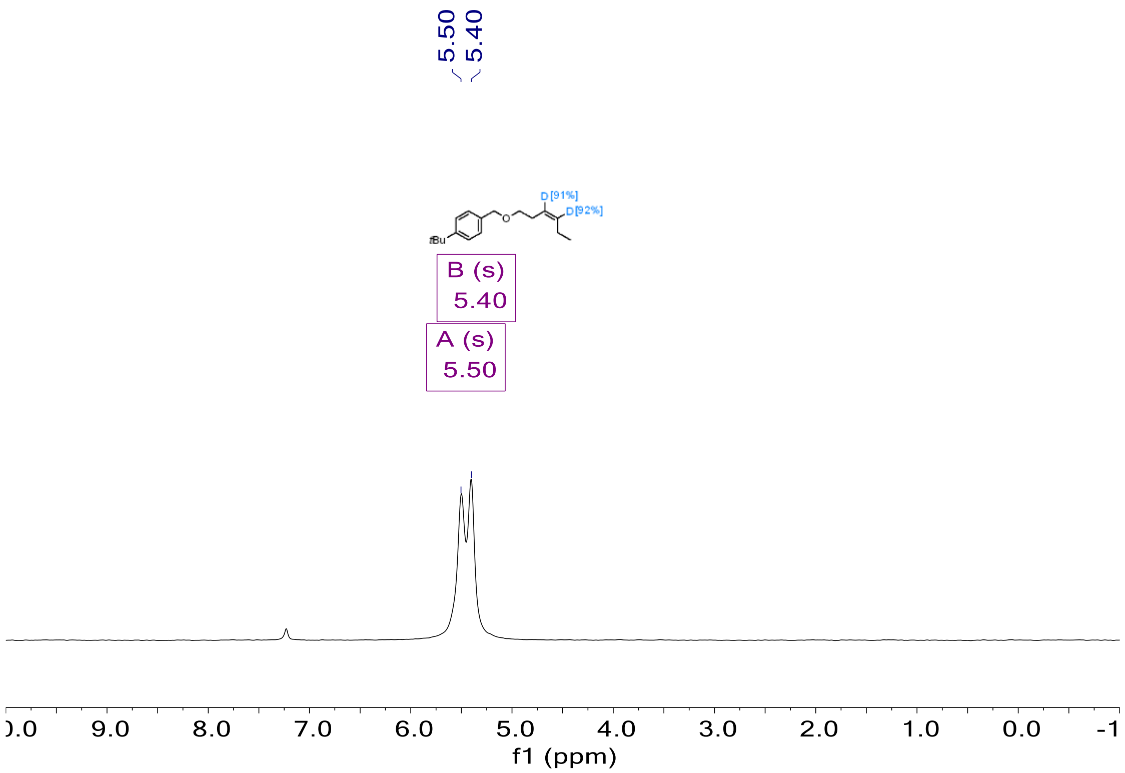

^{2}H NMR (61 MHz, 298 K, Chloroform) spectra for **2**

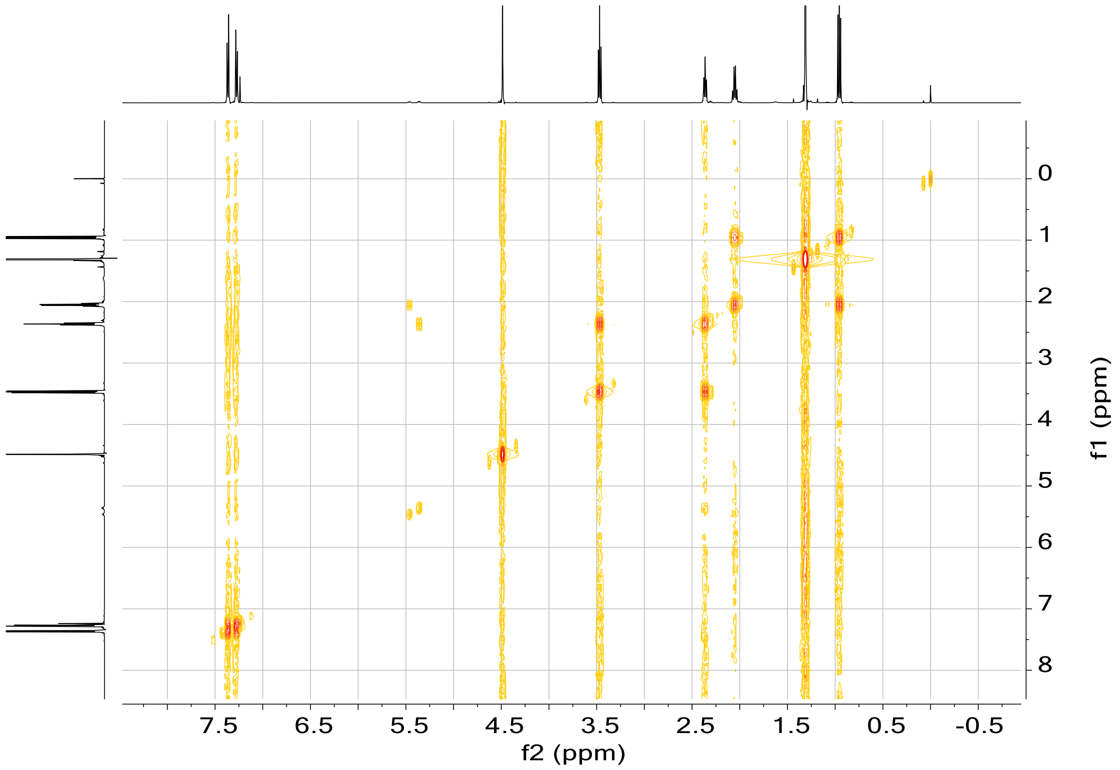

H,H-COSY 90° (500 MHz, 298 K, Chloroform-*d*) spectra for **2**

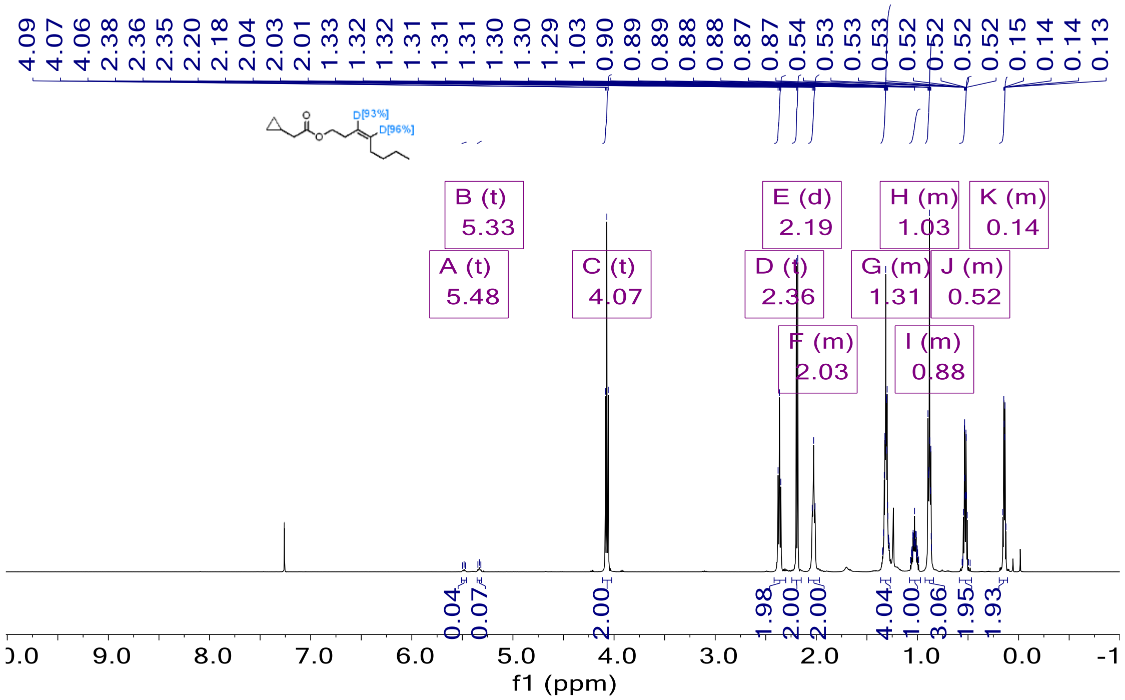

^{1}H NMR (500 MHz, 298 K, Chloroform-*d*) spectra for **3**

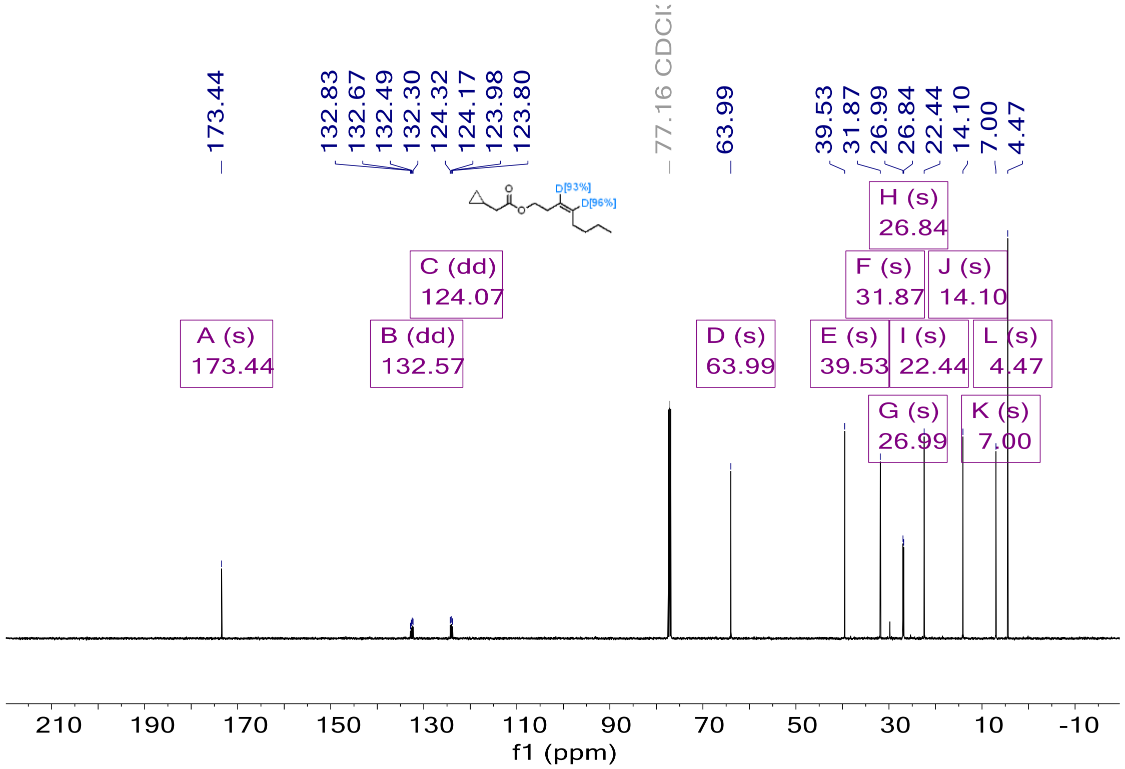

^{13}C NMR (126 MHz, 298 K, Chloroform-*d*) spectra for **3**

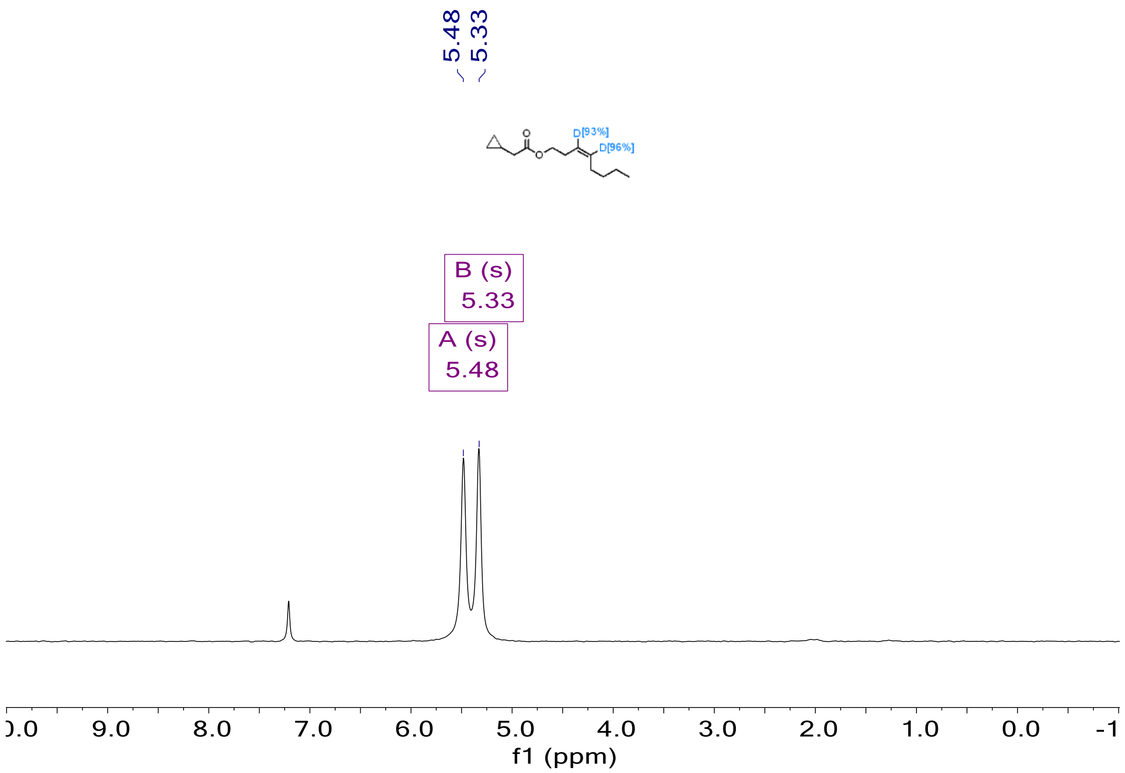

^{2}H NMR (92 MHz, 298 K, Chloroform) spectra for **3**

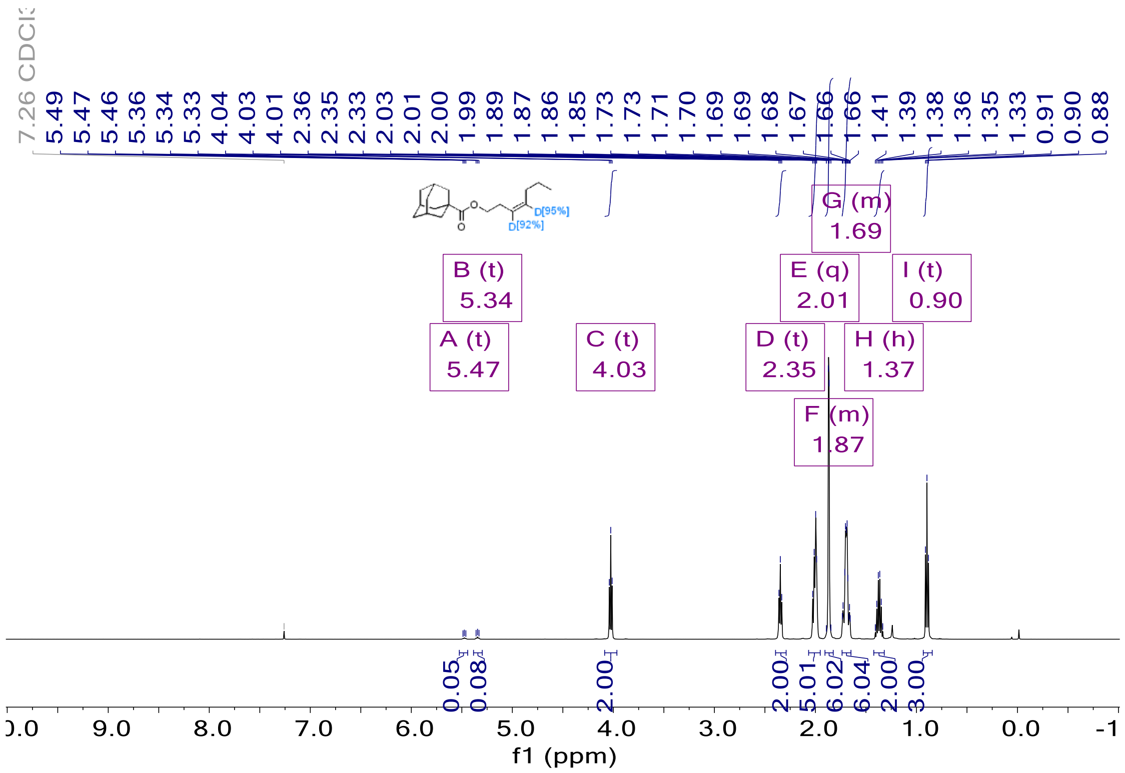

^{1}H NMR (500 MHz, 298 K, Chloroform-*d*) spectra for **4**

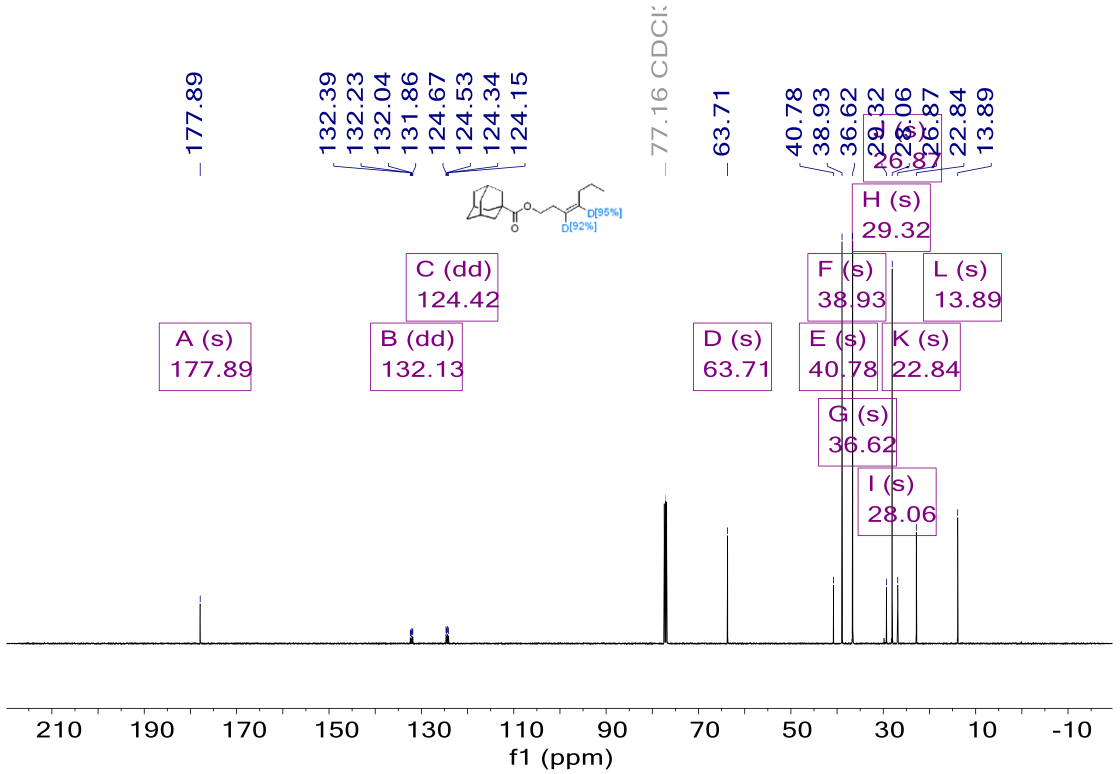

^{13}C NMR (126 MHz, 298 K, Chloroform-*d*) spectra for **4**

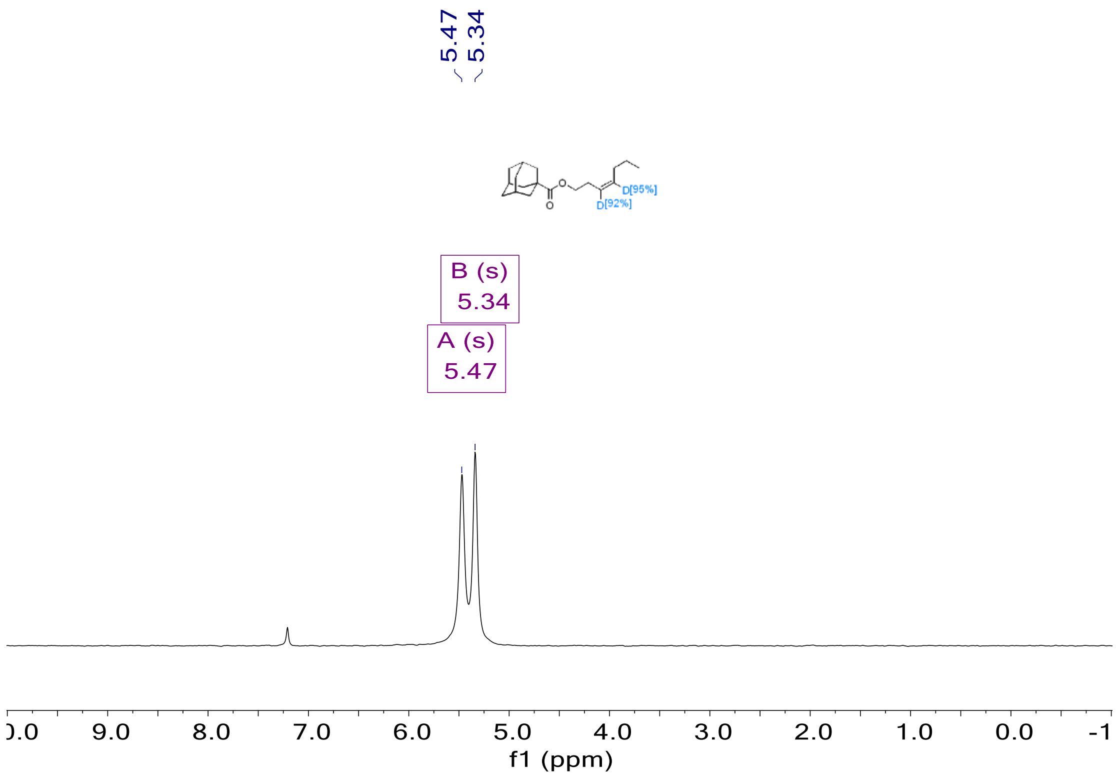

^{2}H NMR (92 MHz, 298 K, Chloroform) spectra for **4**

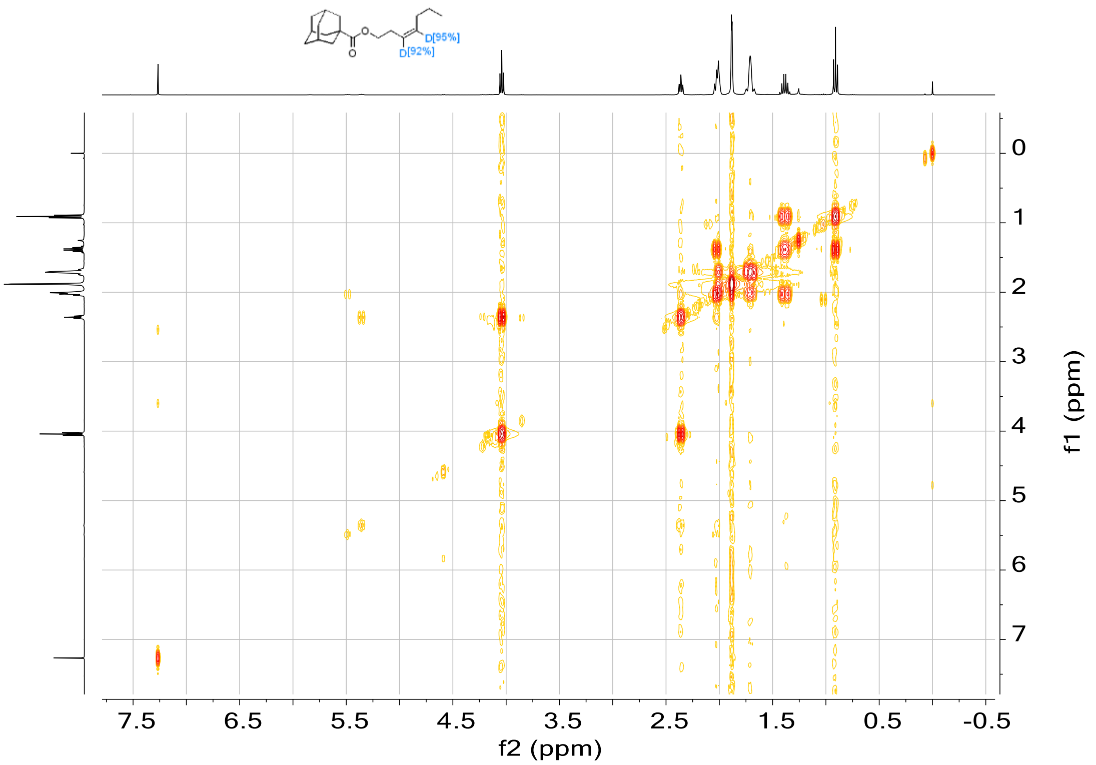

H,H-COSY 90° (500 MHz, 298 K, Chloroform-*d*) spectra for **4**

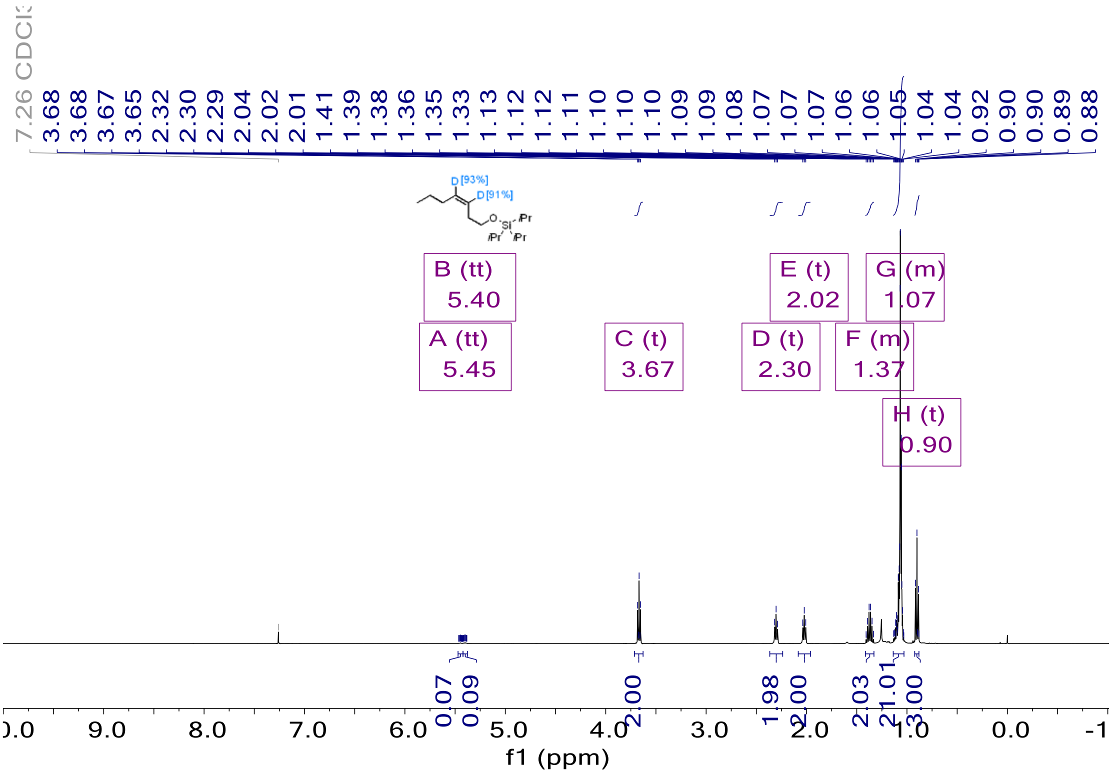

^{1}H NMR (500 MHz, 298 K, Chloroform-*d*) spectra for **5**

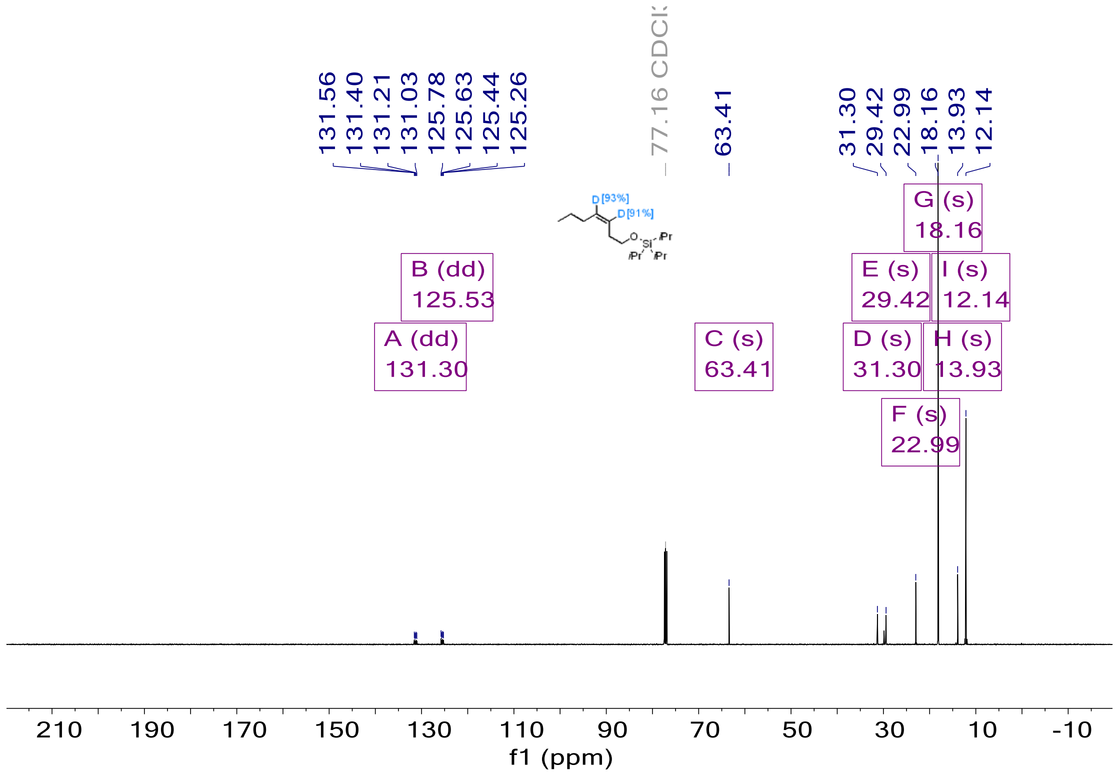

^{13}C NMR (126 MHz, 298 K, Chloroform-*d*) spectra for **5**

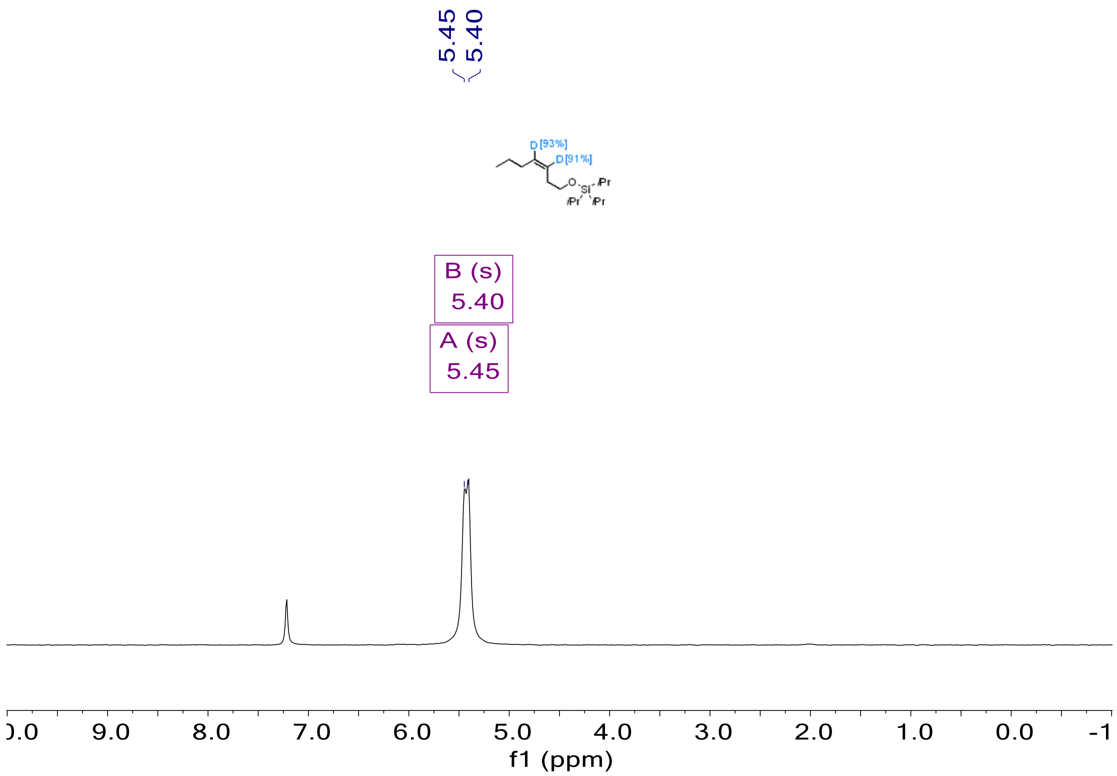

^{2}H NMR (92 MHz, 298 K, Chloroform) spectra for **5**

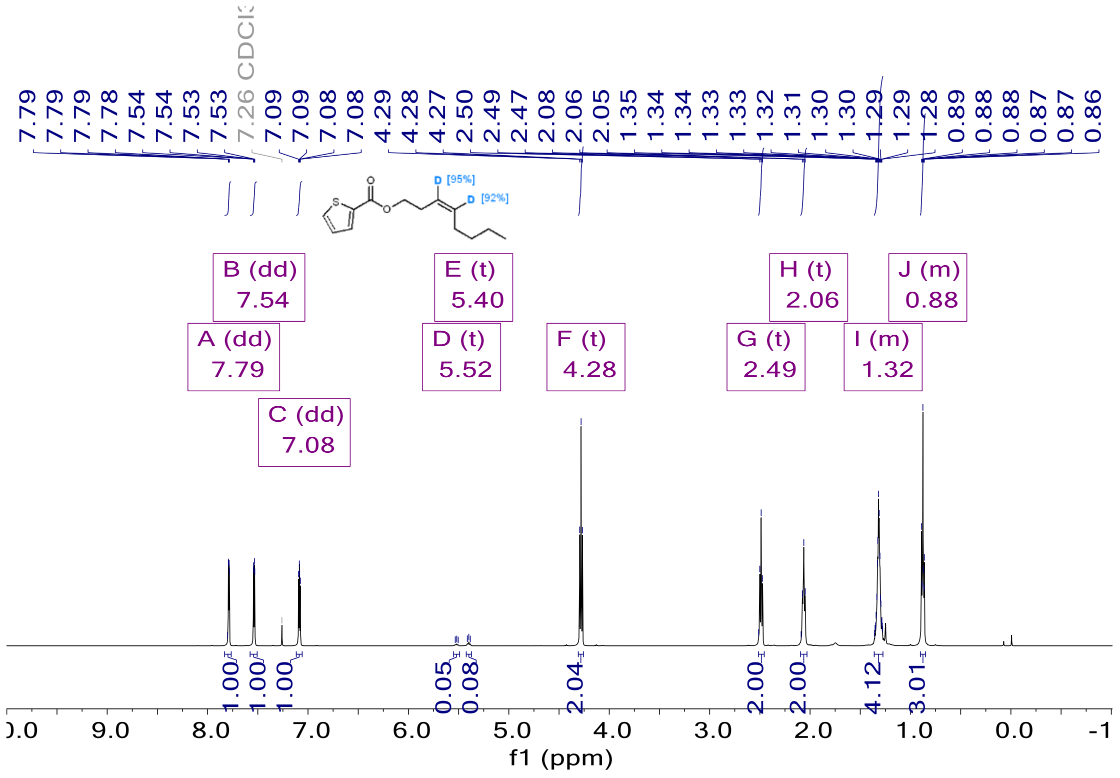

^{1}H NMR (500 MHz, 298 K, Chloroform-*d*) spectra for **6**

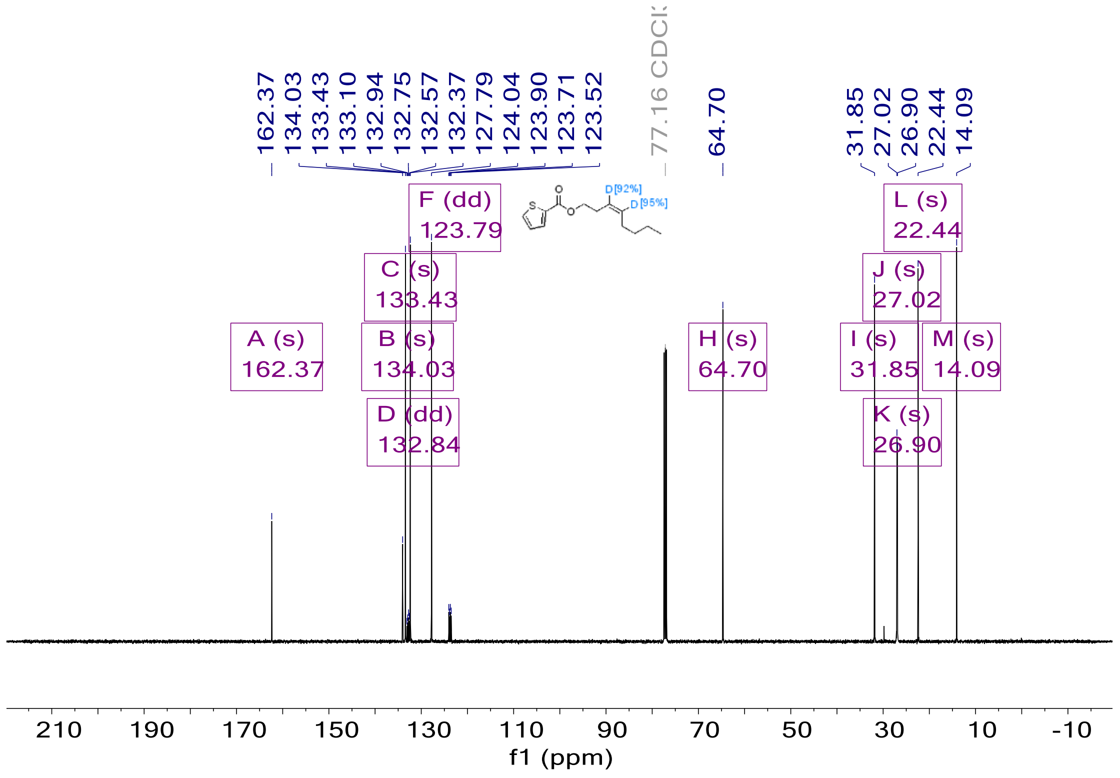

^{13}C NMR (126 MHz, 298 K, Chloroform-*d*) spectra for **6**

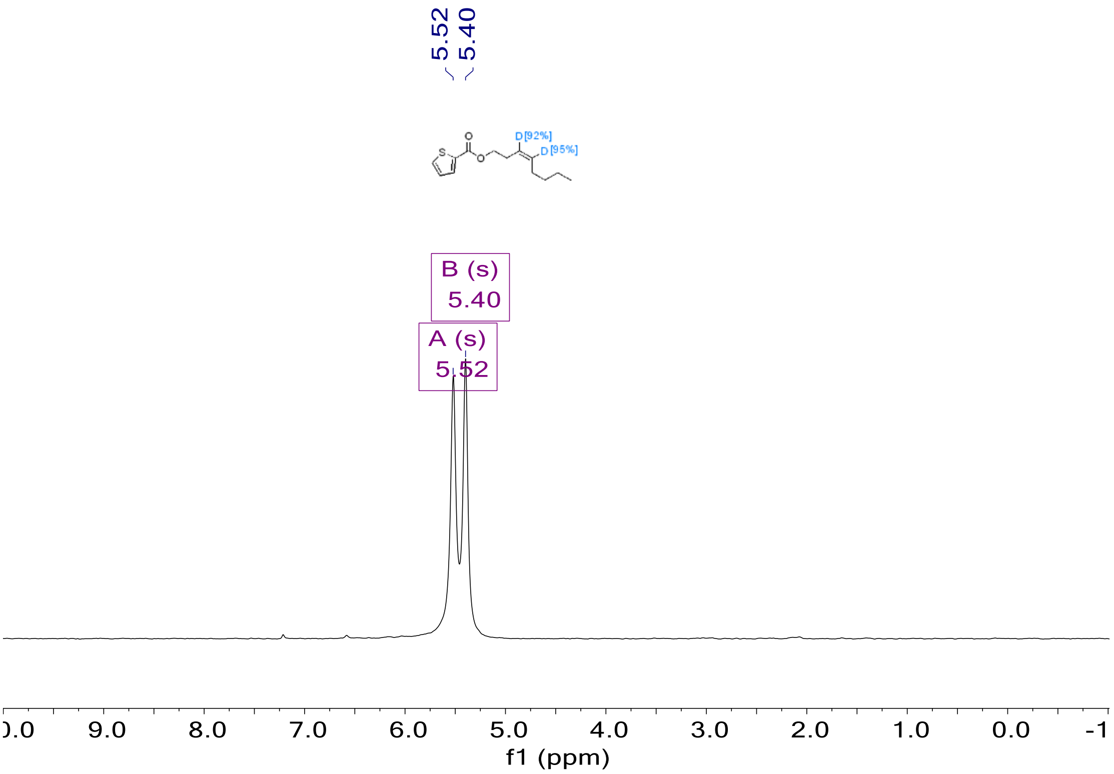

^{2}H NMR (92 MHz, 298 K, Chloroform) spectra for **6**

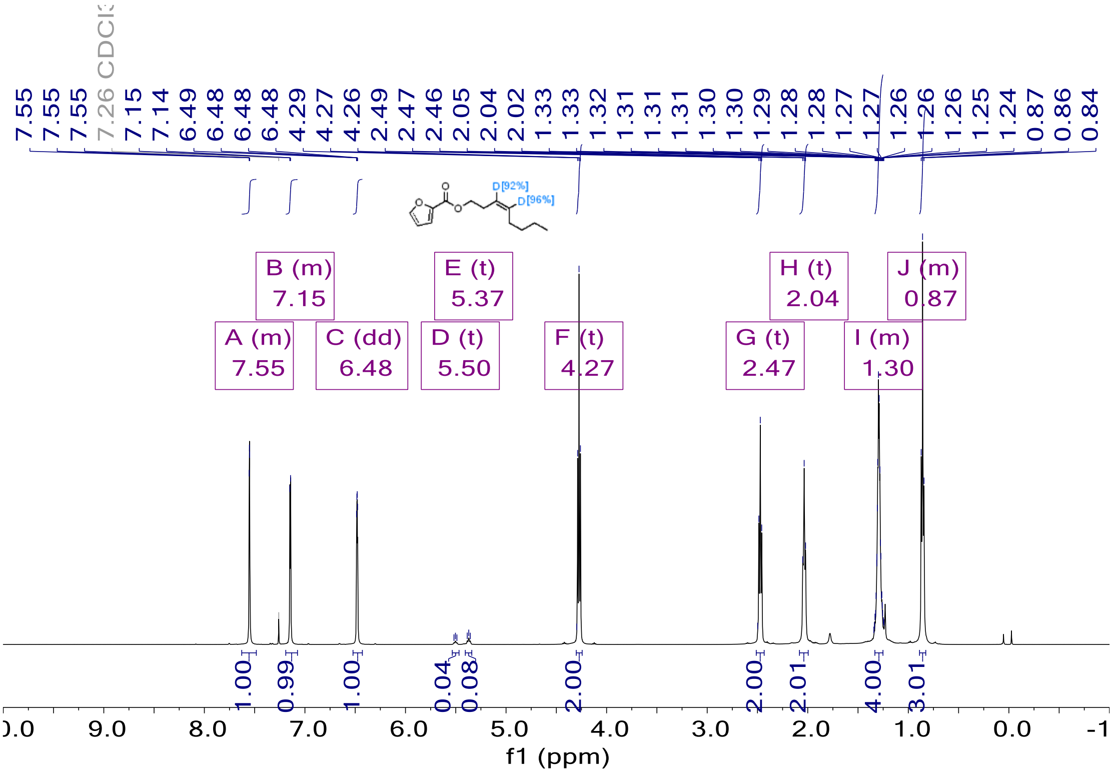

^{1}H NMR (500 MHz, 298 K, Chloroform-*d*) spectra for **7**

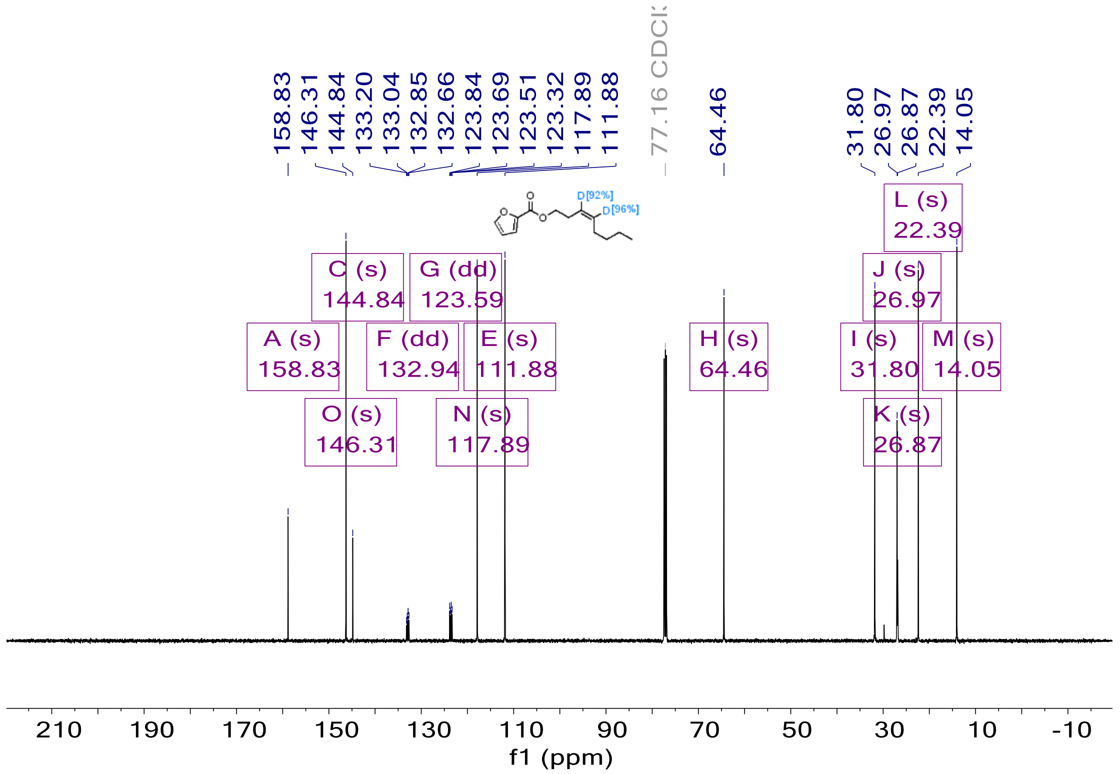

^{13}C NMR (126 MHz, 298 K, Chloroform-*d*) spectra for **7**

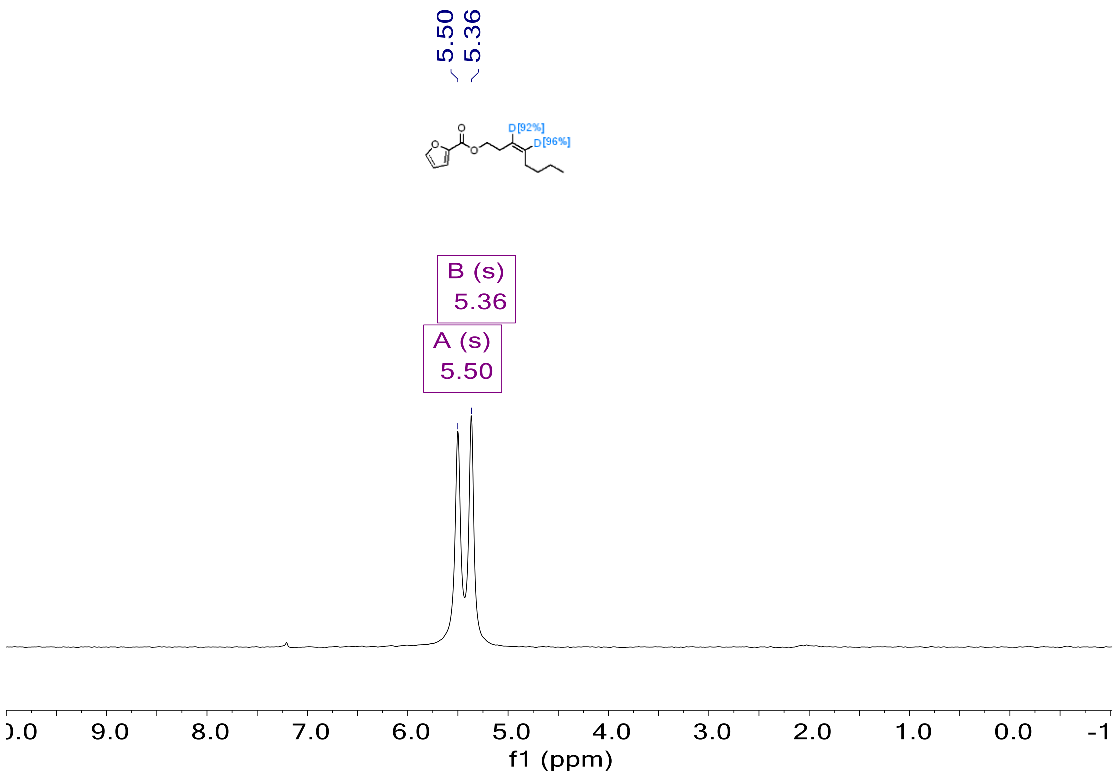

^{2}H NMR (92 MHz, 298 K, Chloroform) spectra for **7**

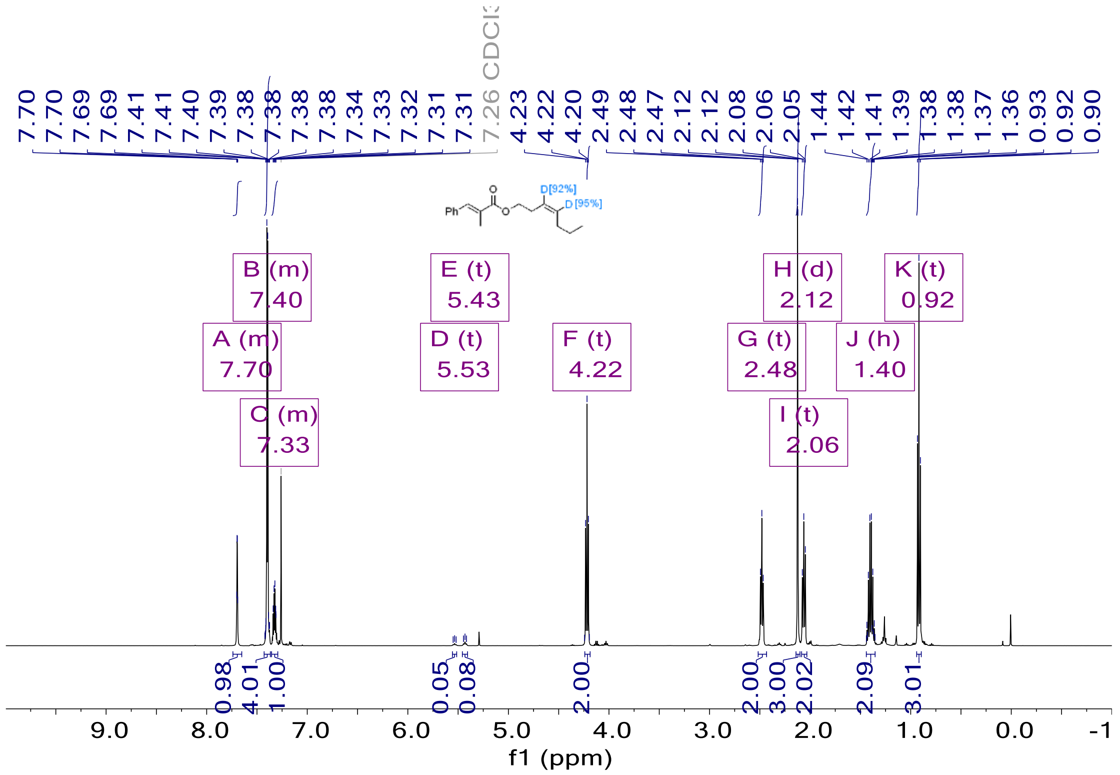

^{1}H NMR (500 MHz, 298 K, Chloroform-*d*) spectra for **8**

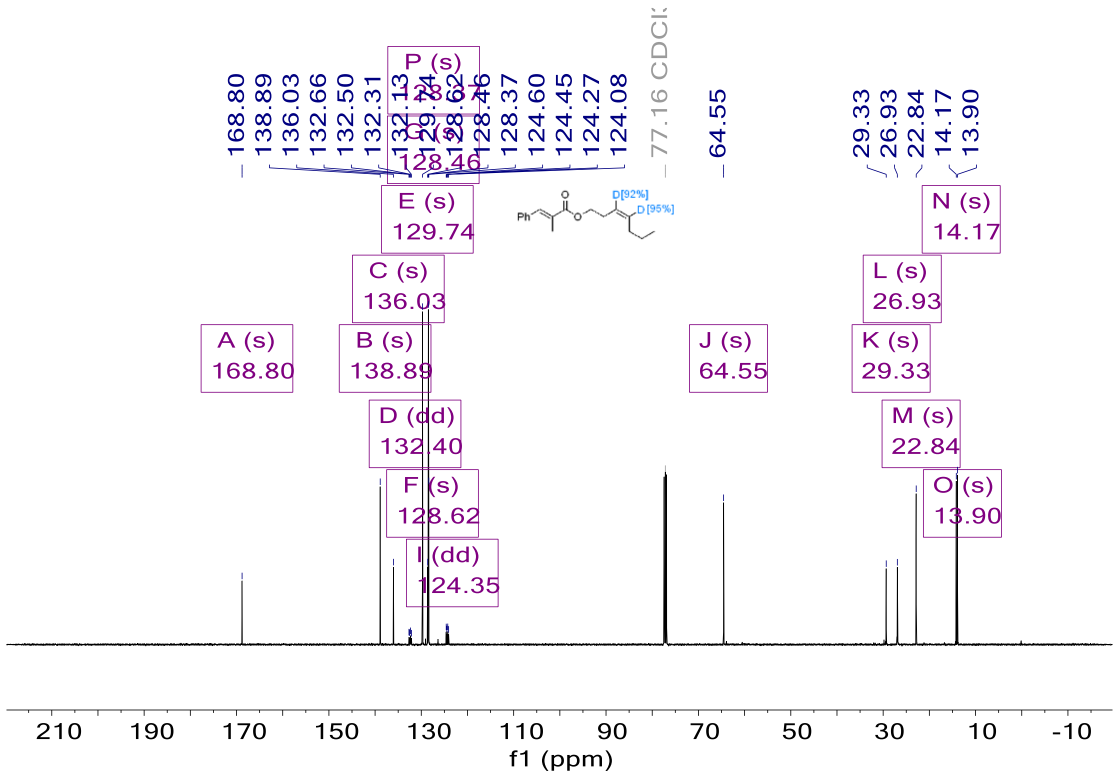

^{13}C NMR (126 MHz, 298 K, Chloroform-*d*) spectra for **8**

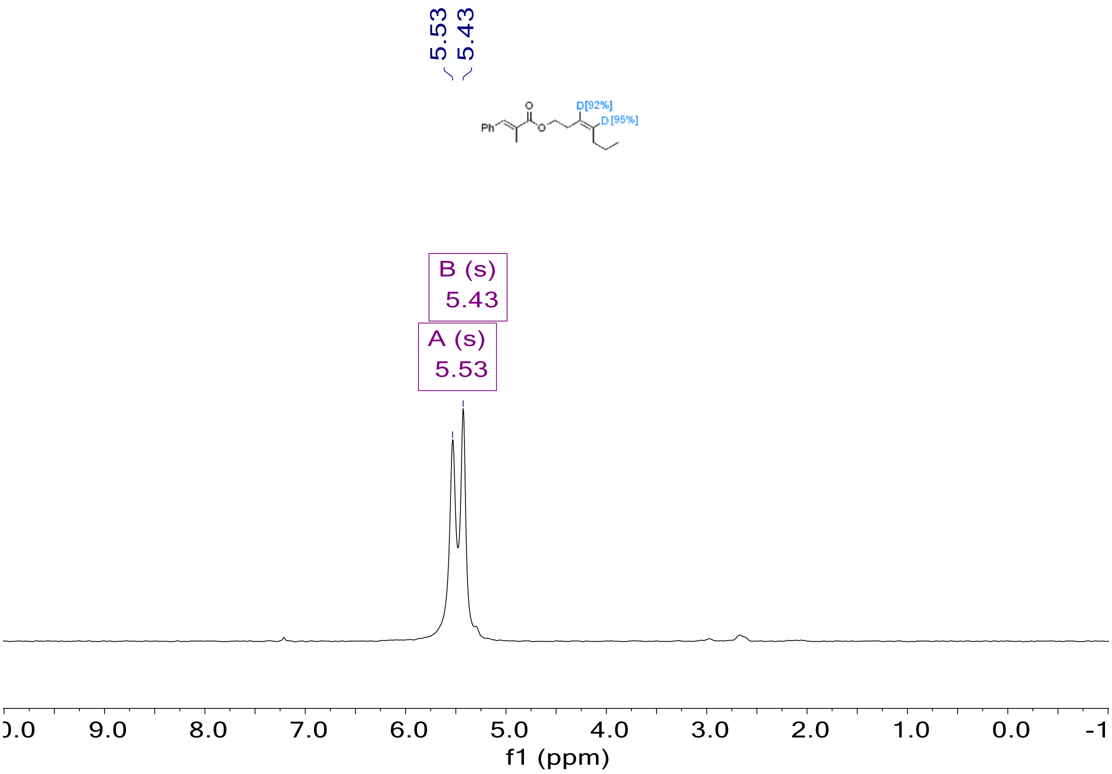

^{2}H NMR (92 MHz, 298 K, Chloroform) spectra for **8**

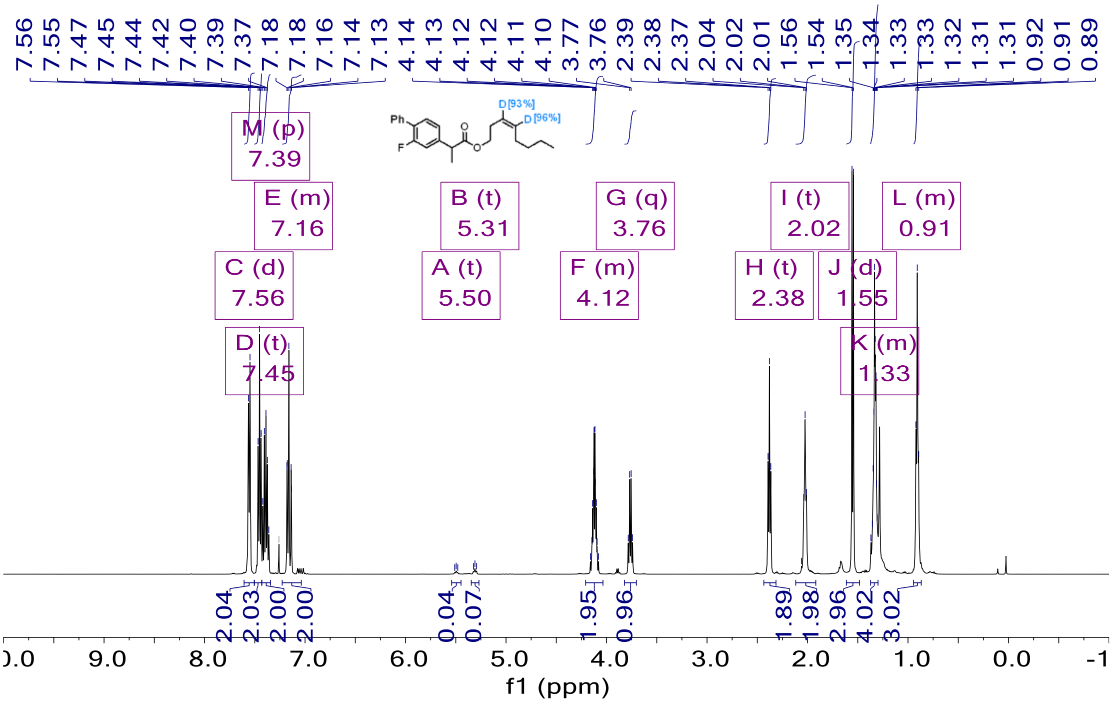

^{1}H NMR (500 MHz, 298 K, Chloroform-*d*) spectra for **9**

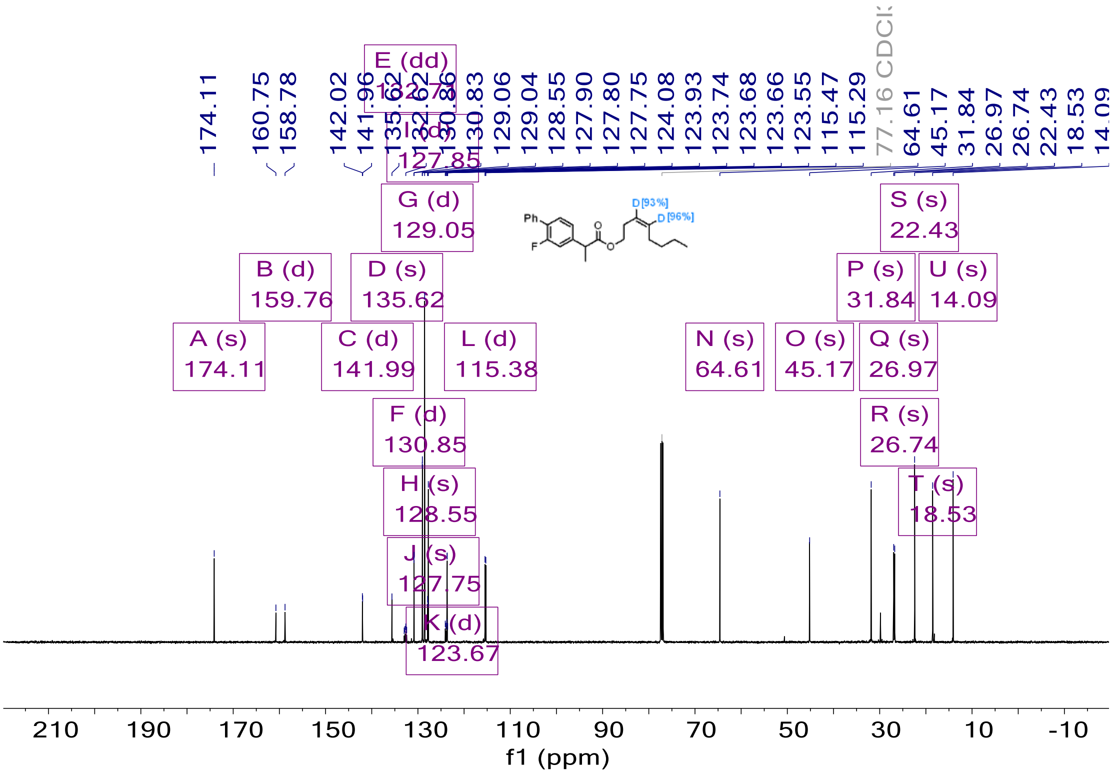

^{13}C NMR (126 MHz, 298 K, Chloroform-*d*) spectra for **9**

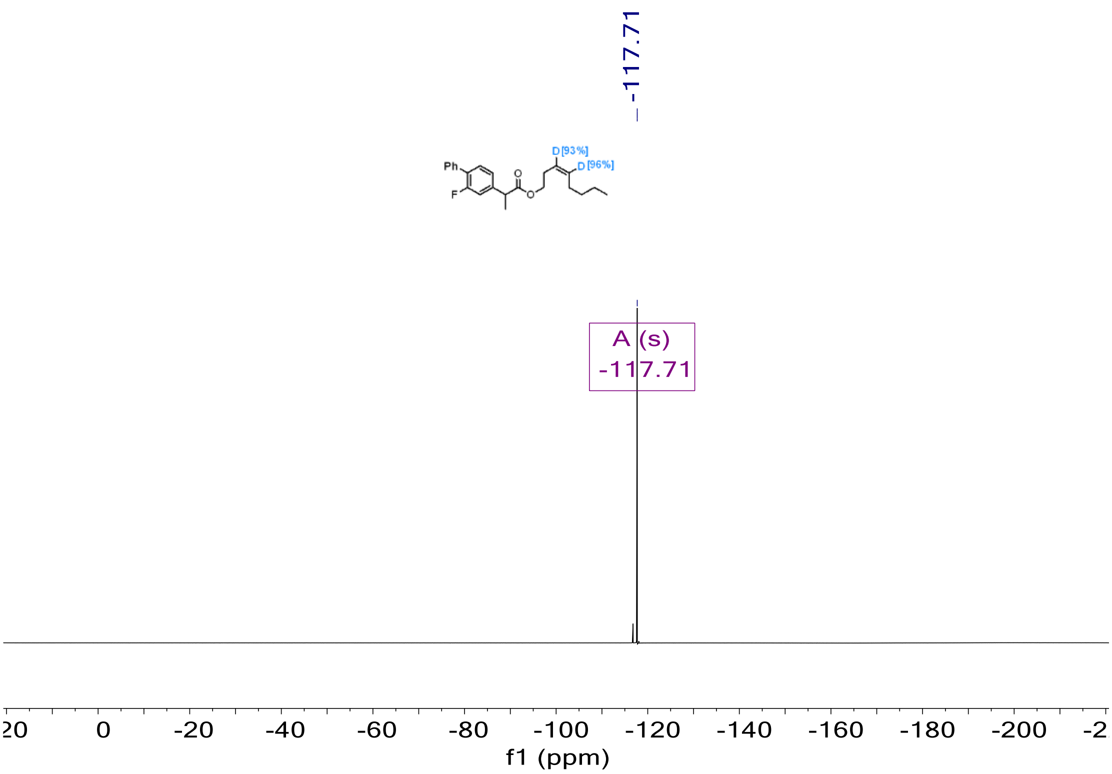

^{19}F NMR (471 MHz, 298 K, Chloroform-*d*) spectra for **9**

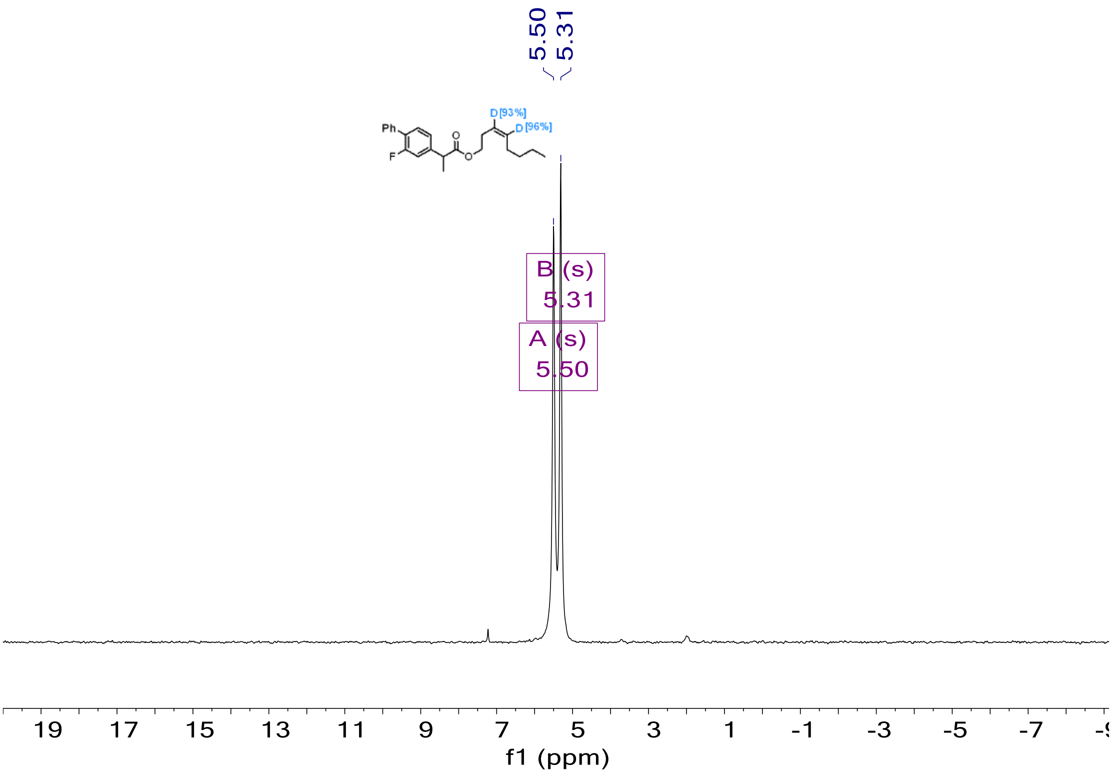

^{2}H NMR (92 MHz, 298 K, Chloroform) spectra for **9**

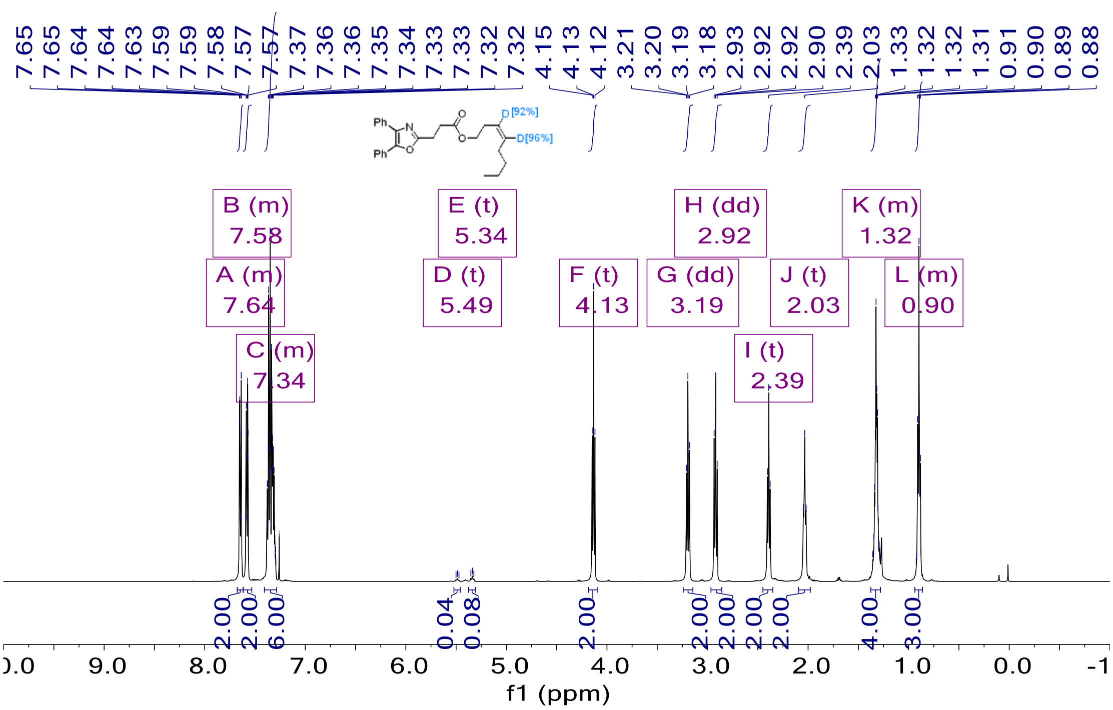

^{1}H NMR (500 MHz, 298 K, Chloroform-*d*) spectra for **10**

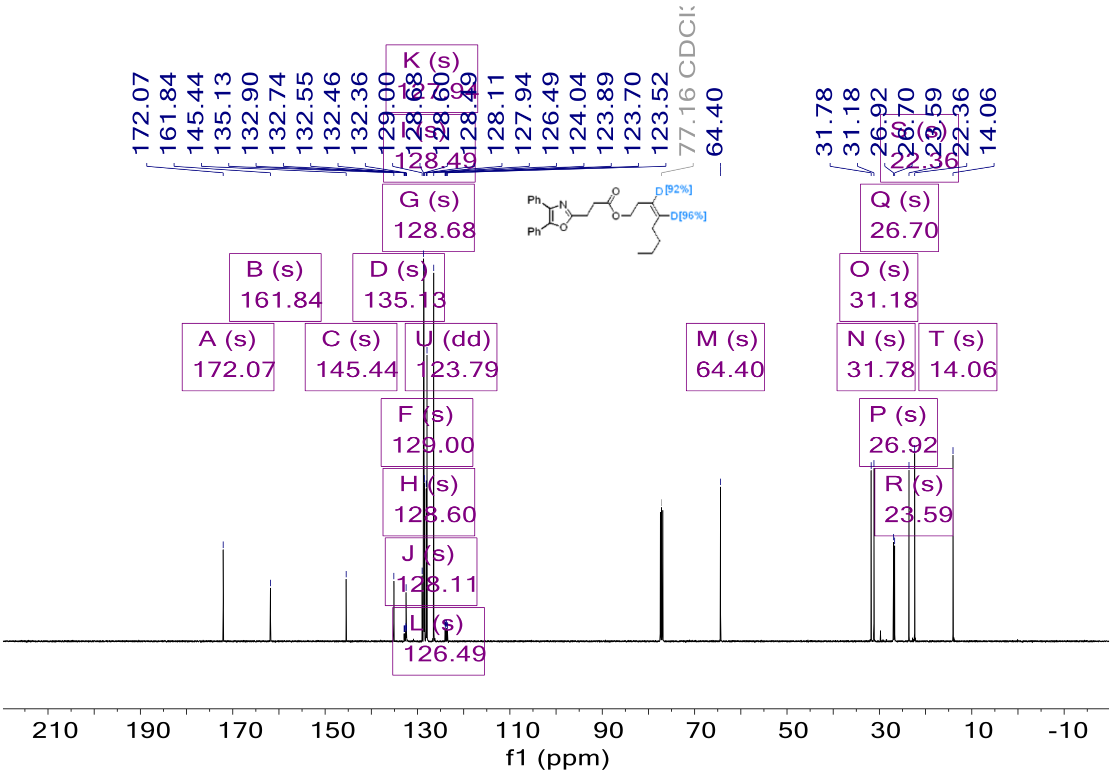

^{13}C NMR (126 MHz, 298 K, Chloroform-*d*) spectra for **10**

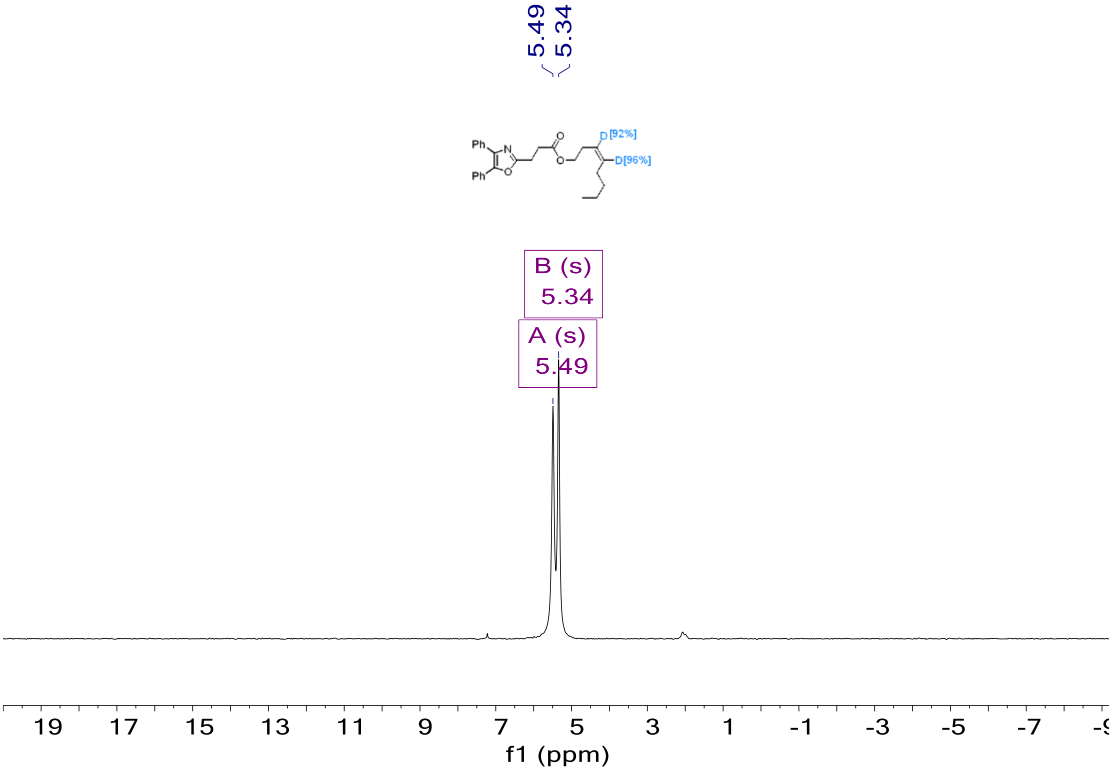

^{2}H NMR (92 MHz, 298 K, Chloroform) spectra for **10**

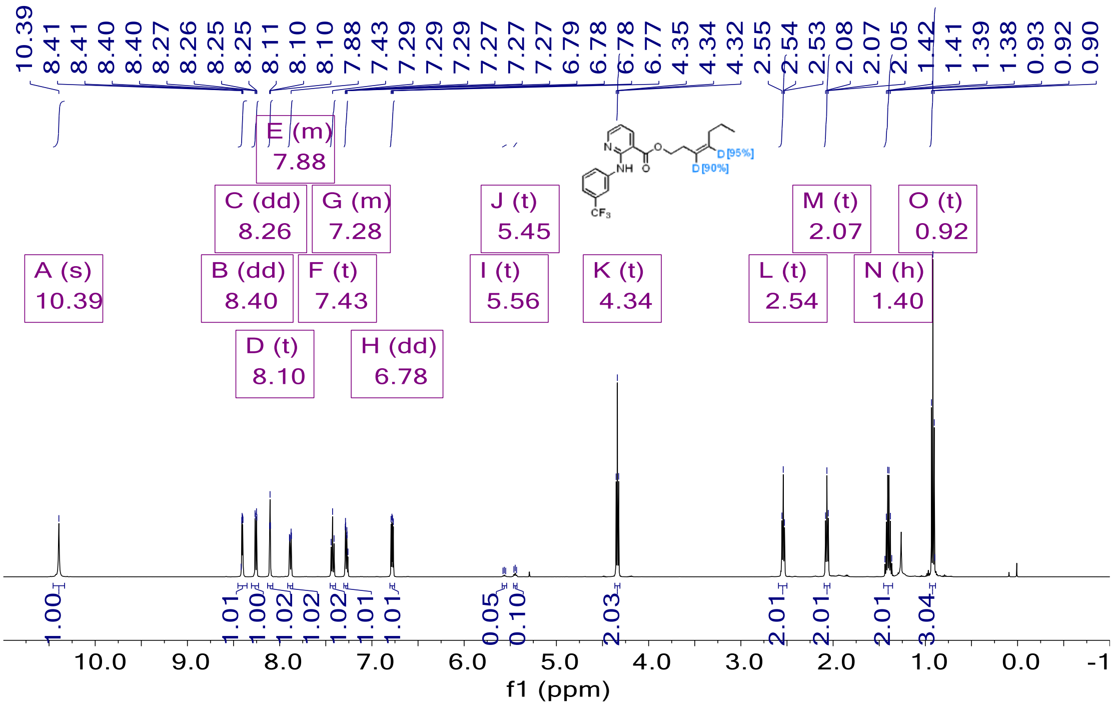

^{1}H NMR (500 MHz, 298 K, Chloroform-*d*) spectra for **11**

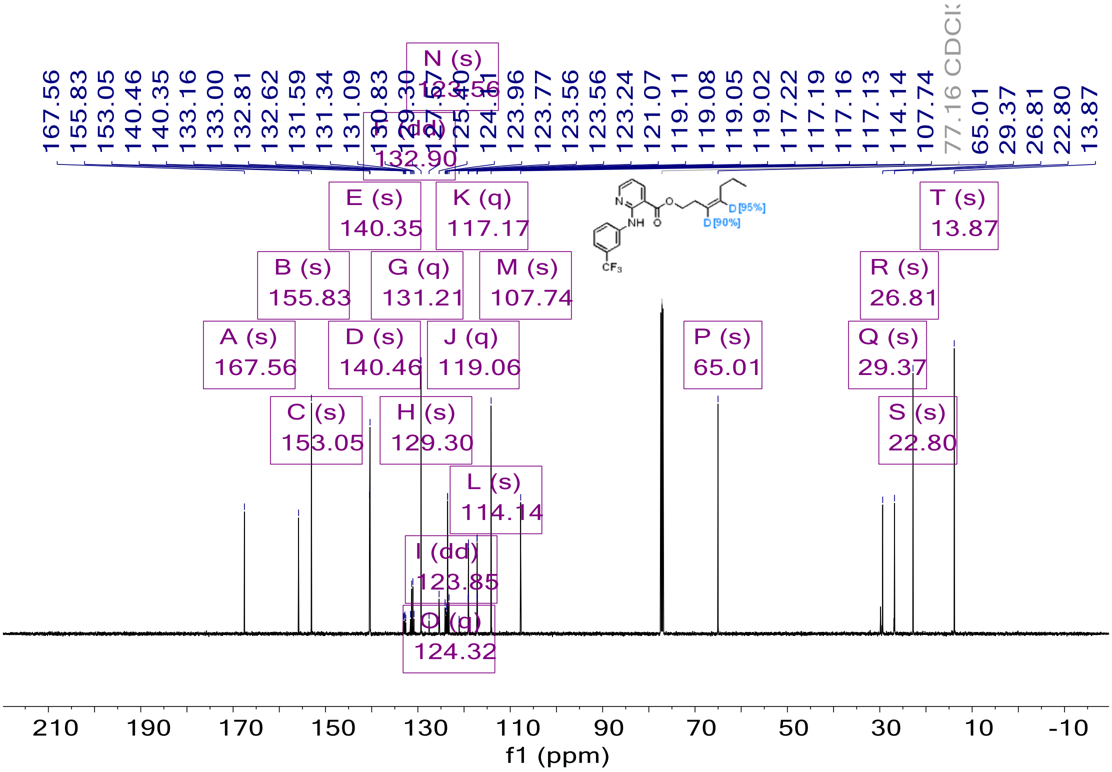

^{13}C NMR (126 MHz, 298 K, Chloroform-*d*) spectra for **11**

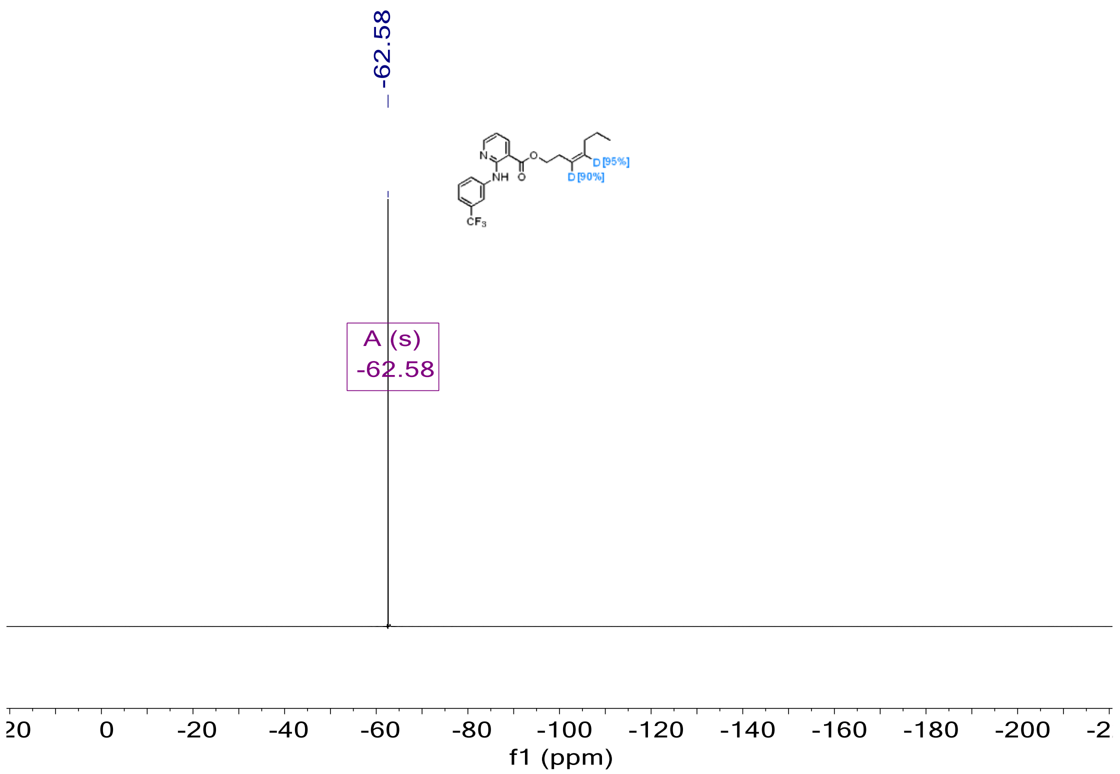

^{19}F NMR (471 MHz, 298 K, Chloroform-*d*) spectra for **11**

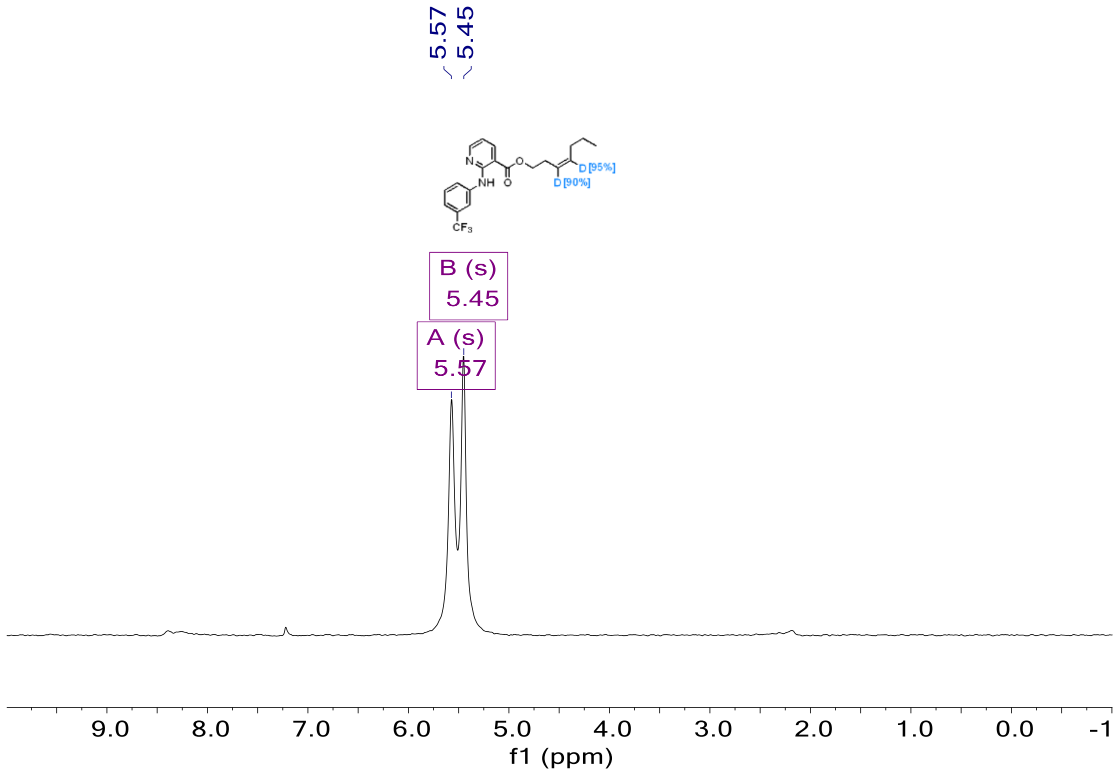

^{2}H NMR (92 MHz, 298 K, Chloroform) spectra for **11**

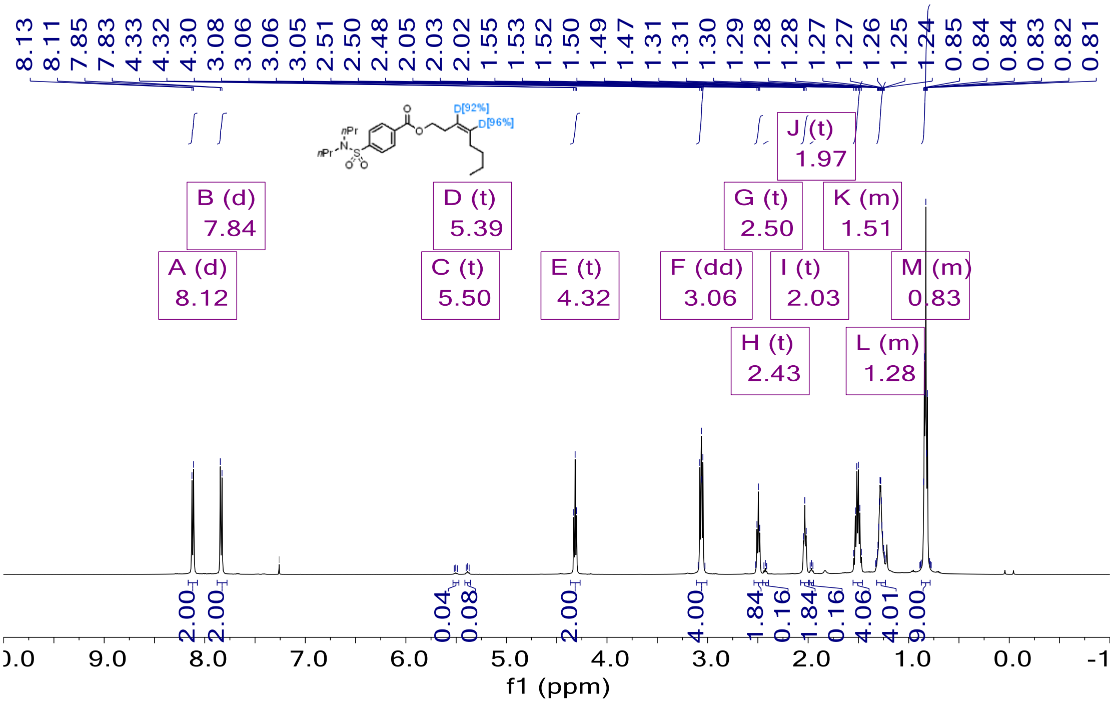

^{1}H NMR (500 MHz, 298 K, Chloroform-*d*) spectra for **12**

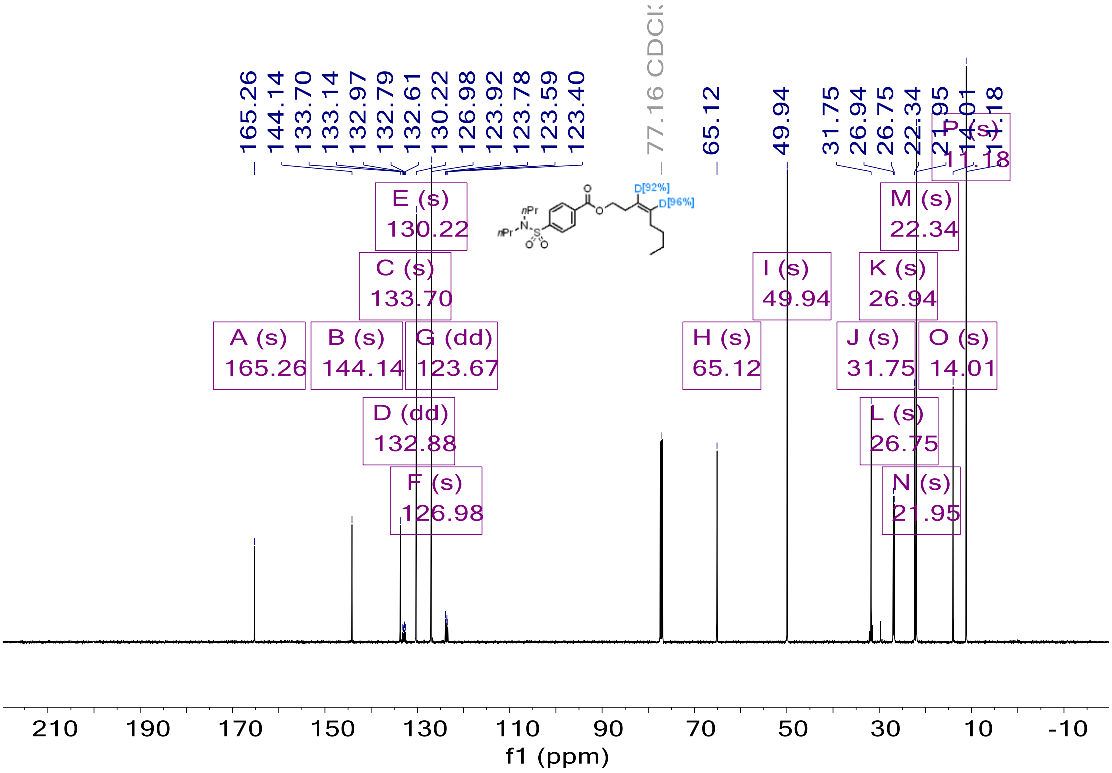

^{13}C NMR (126 MHz, 298 K, Chloroform-*d*) spectra for **12**

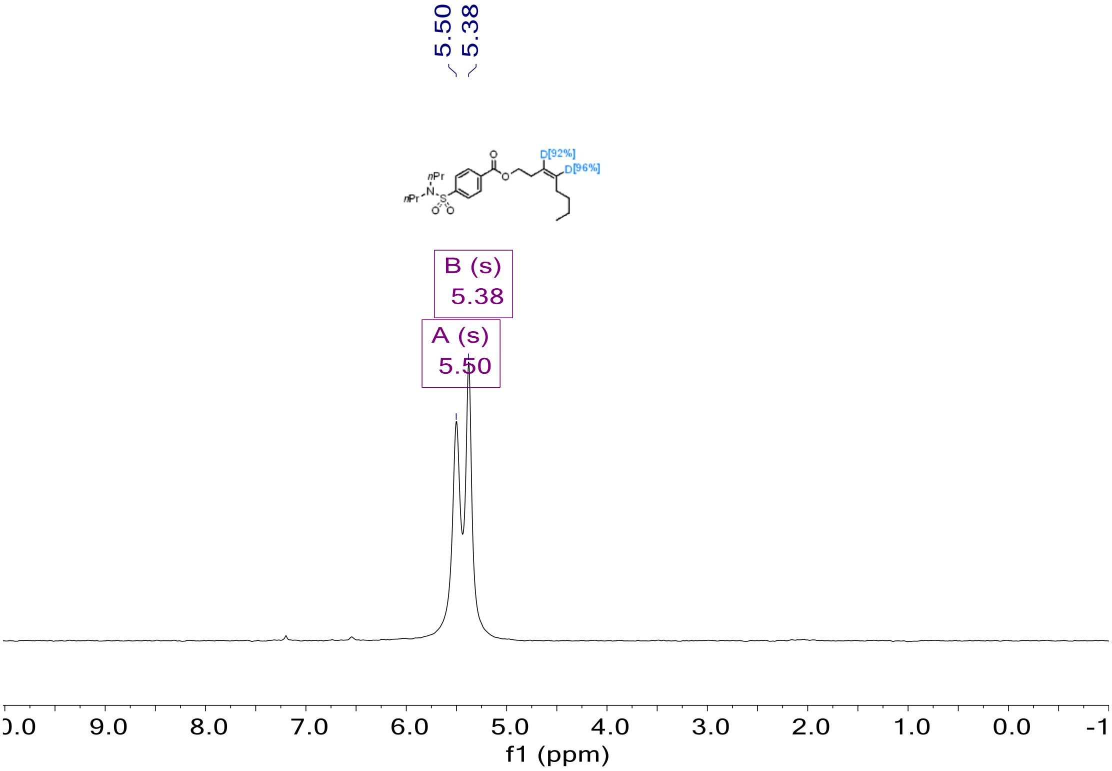

^{2}H NMR (92 MHz, 298 K, Chloroform) spectra for **12**

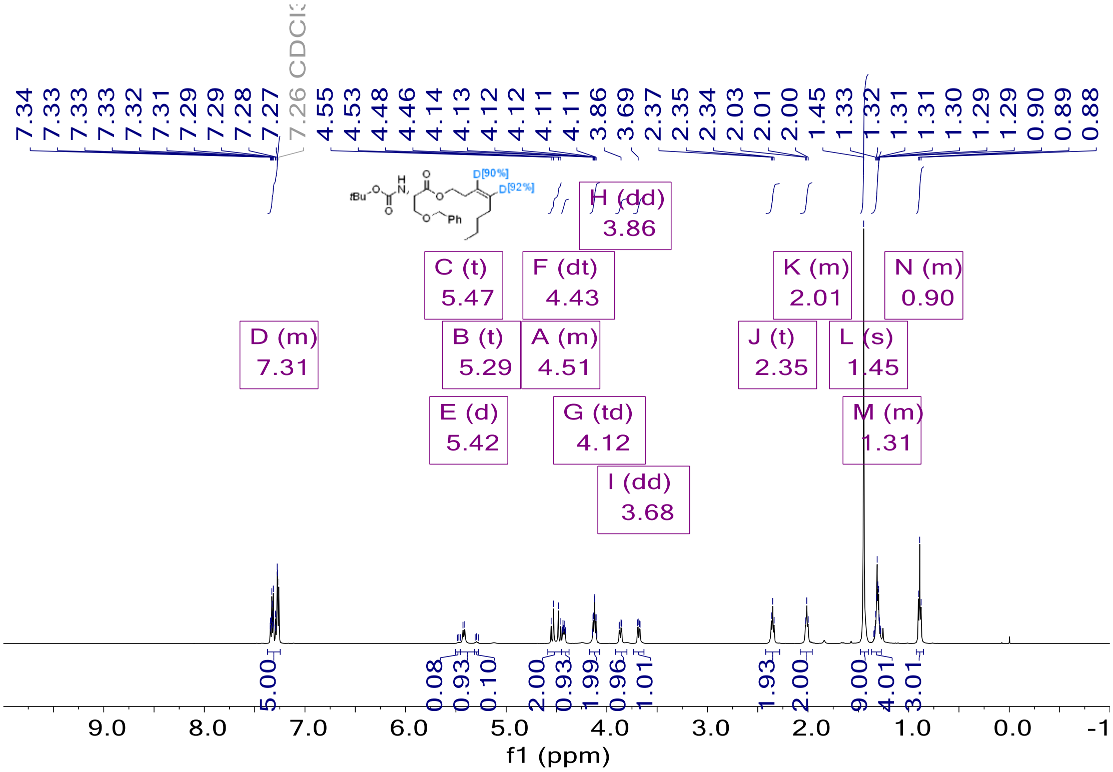

^{1}H NMR (500 MHz, 298 K, Chloroform-*d*) spectra for **13**

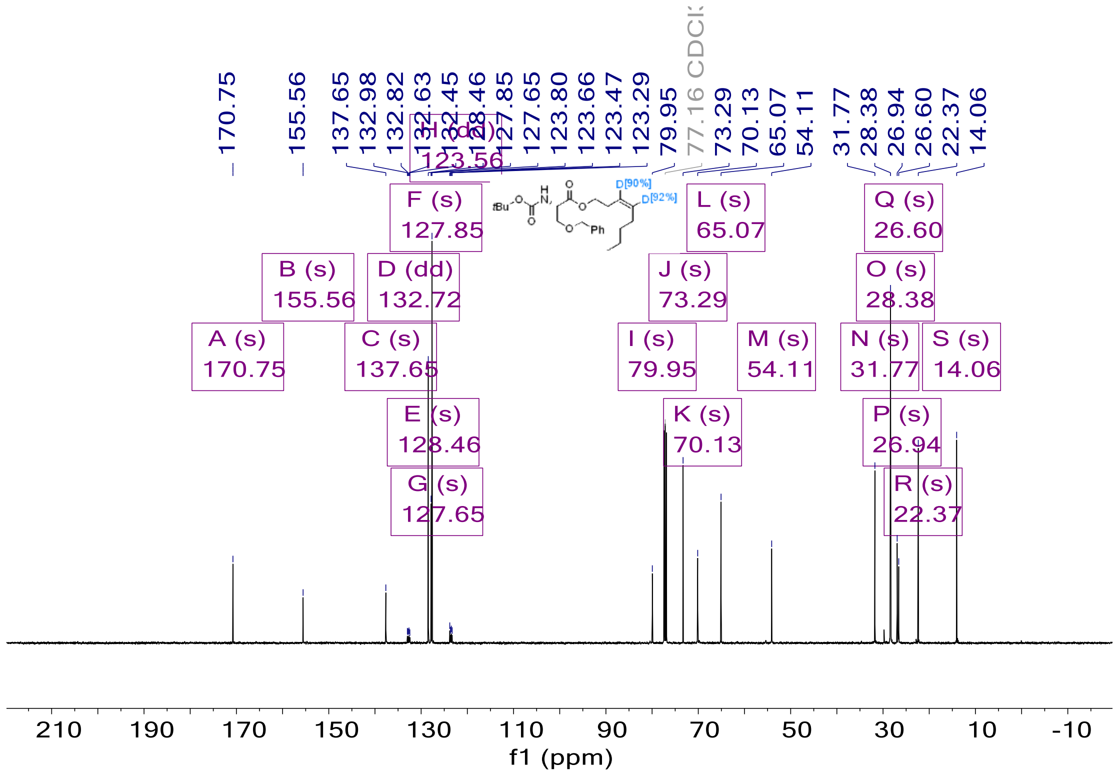

^{13}C NMR (126 MHz, 298 K, Chloroform-*d*) spectra for **13**

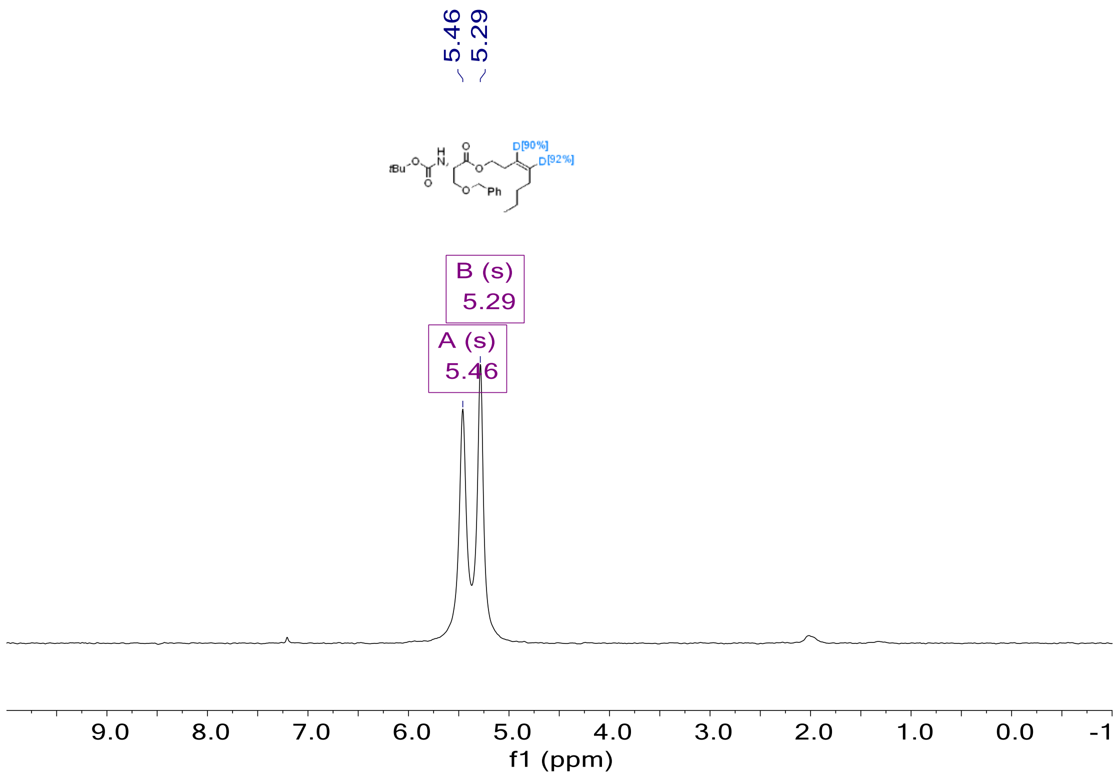

^{2}H NMR (92 MHz, 298 K, Chloroform) spectra for **13**

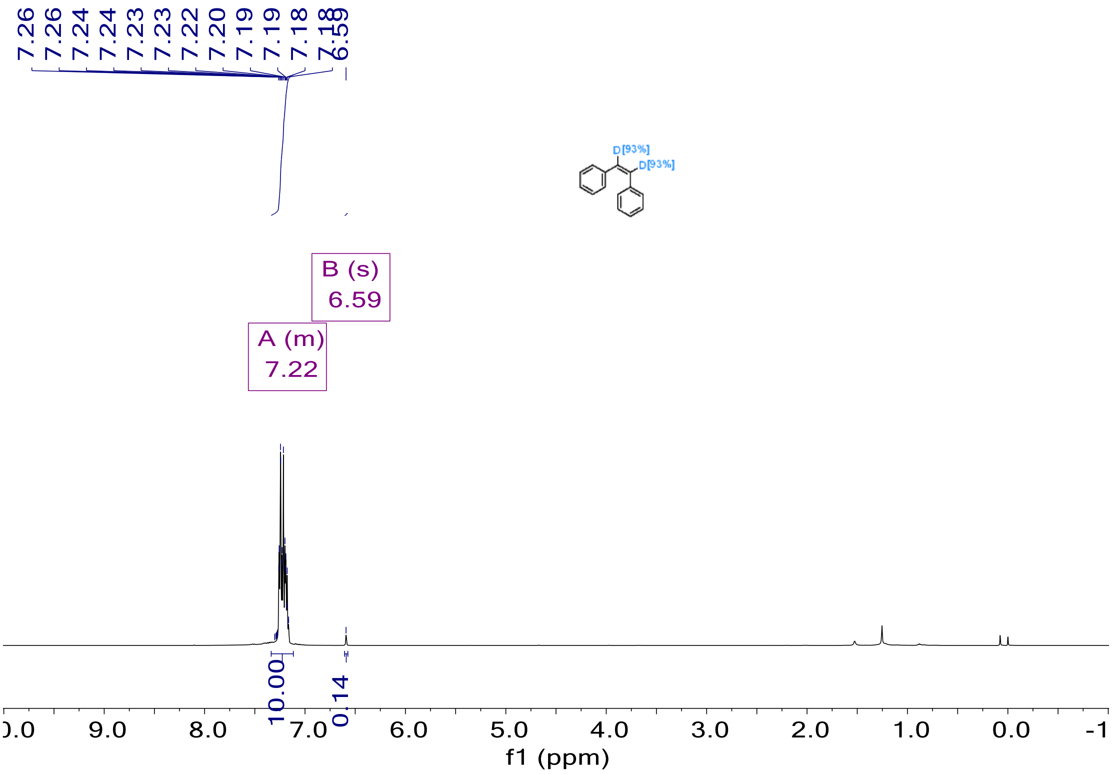

^{1}H NMR (500 MHz, 298 K, Chloroform-*d*) spectra for **14**

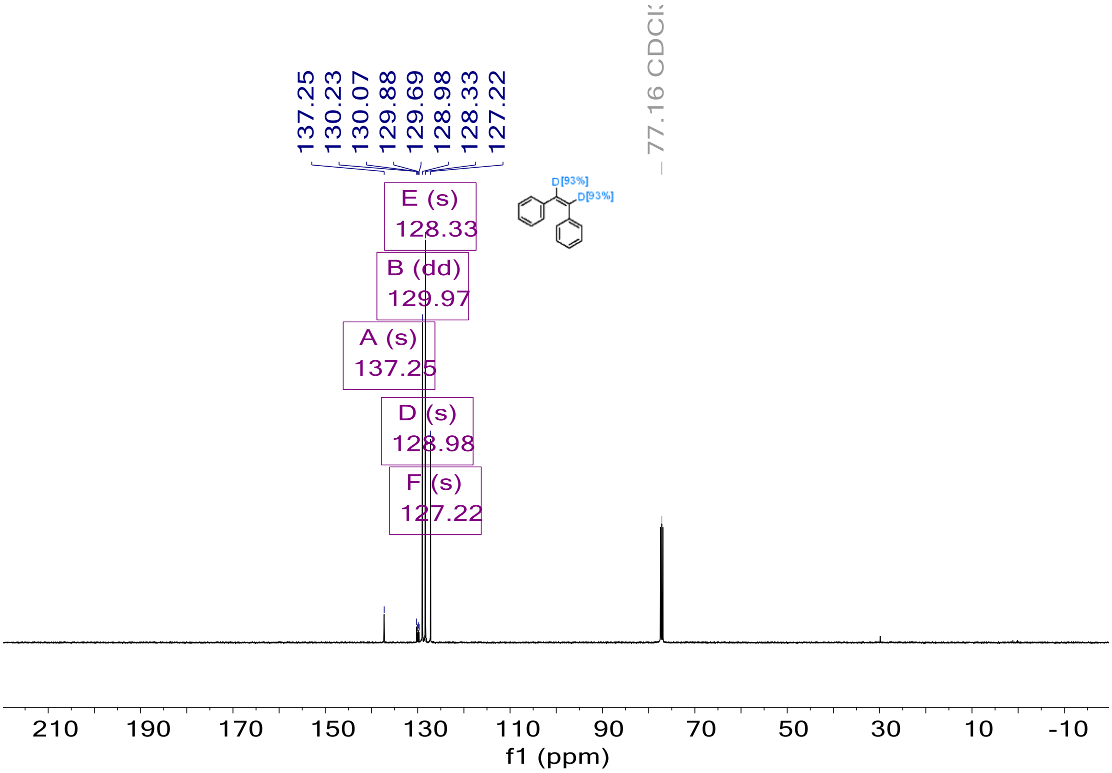

^{13}C NMR (126 MHz, 298 K, Chloroform-*d*) spectra for **14**

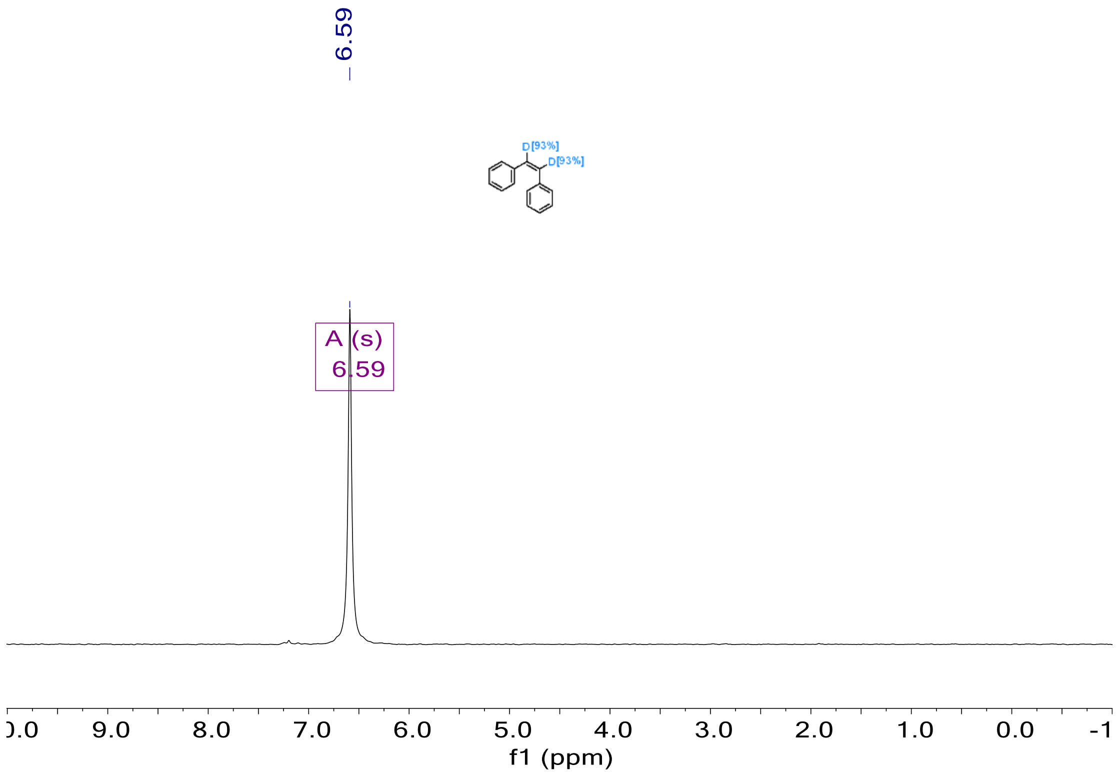

^{2}H NMR (92 MHz, 298 K, Chloroform) spectra for **14**

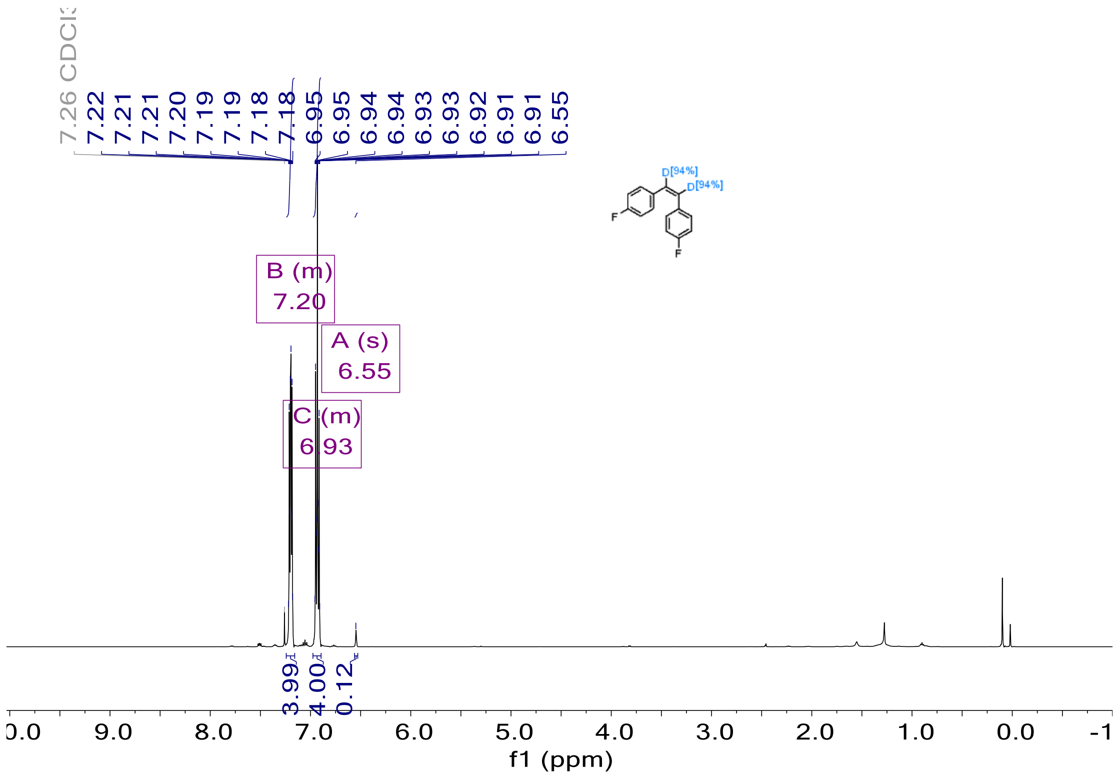

^{1}H NMR (500 MHz, 298 K, Chloroform-*d*) spectra for **15**

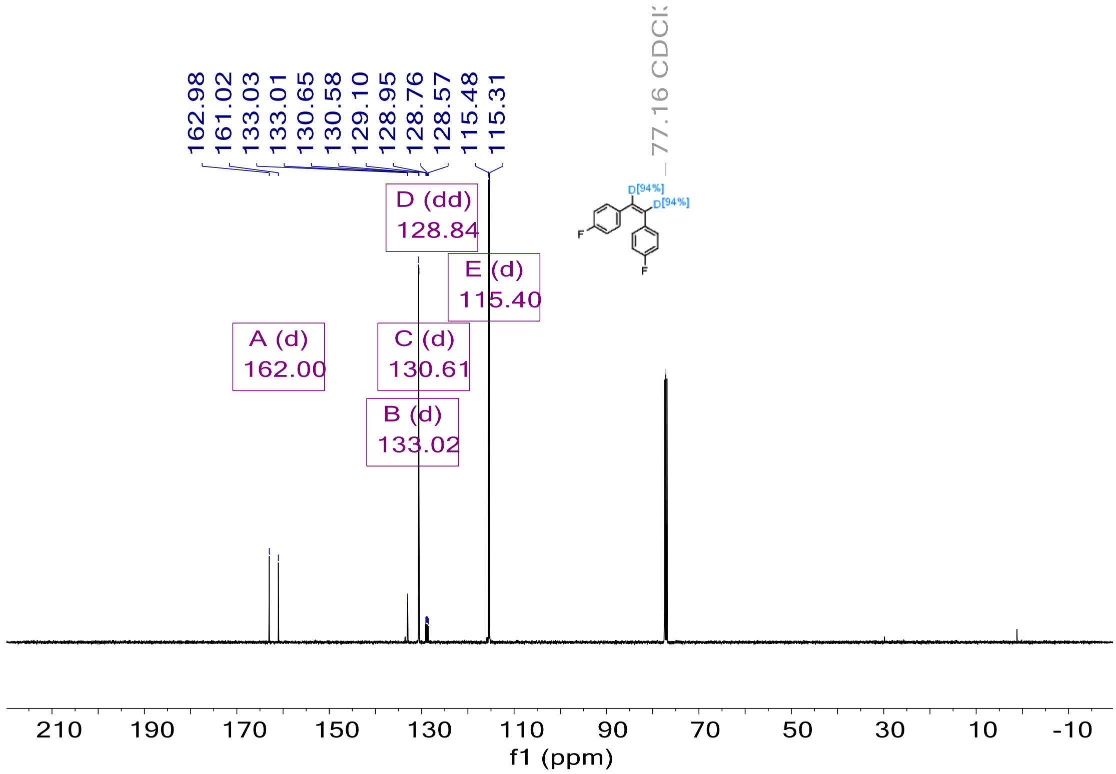

^{13}C NMR (126 MHz, 298 K, Chloroform-*d*) spectra for **15**

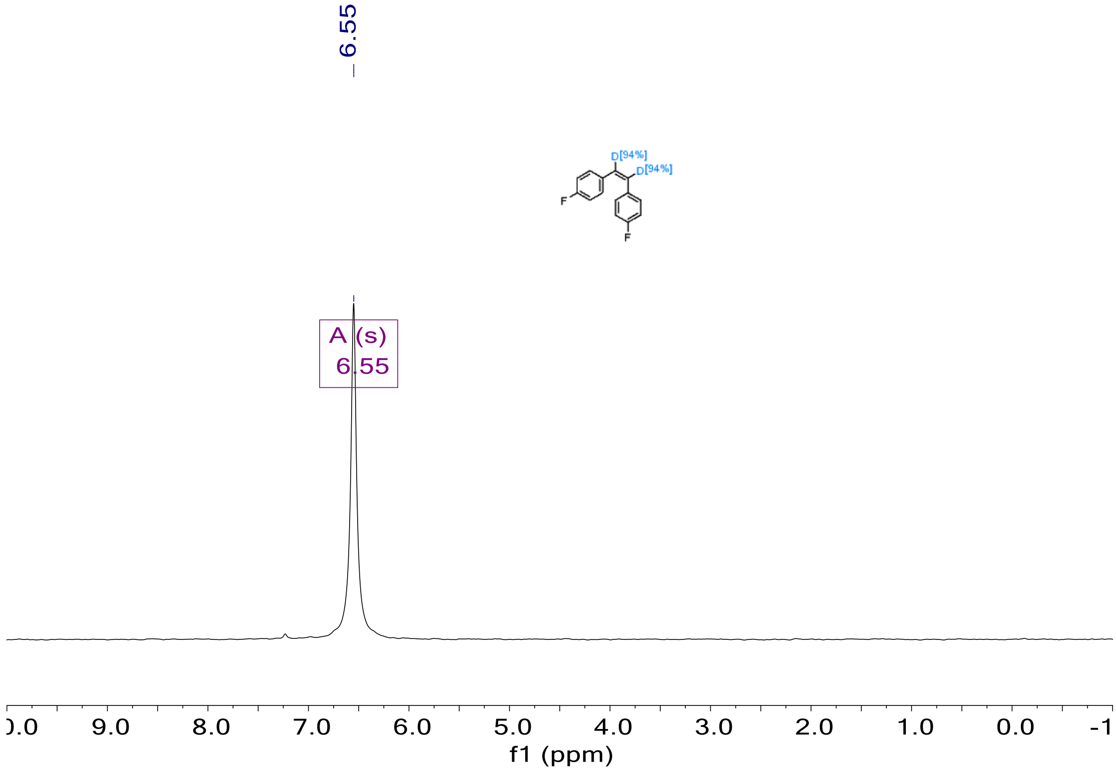

^{2}H NMR (61 MHz, 298 K, Chloroform) spectra for **15**

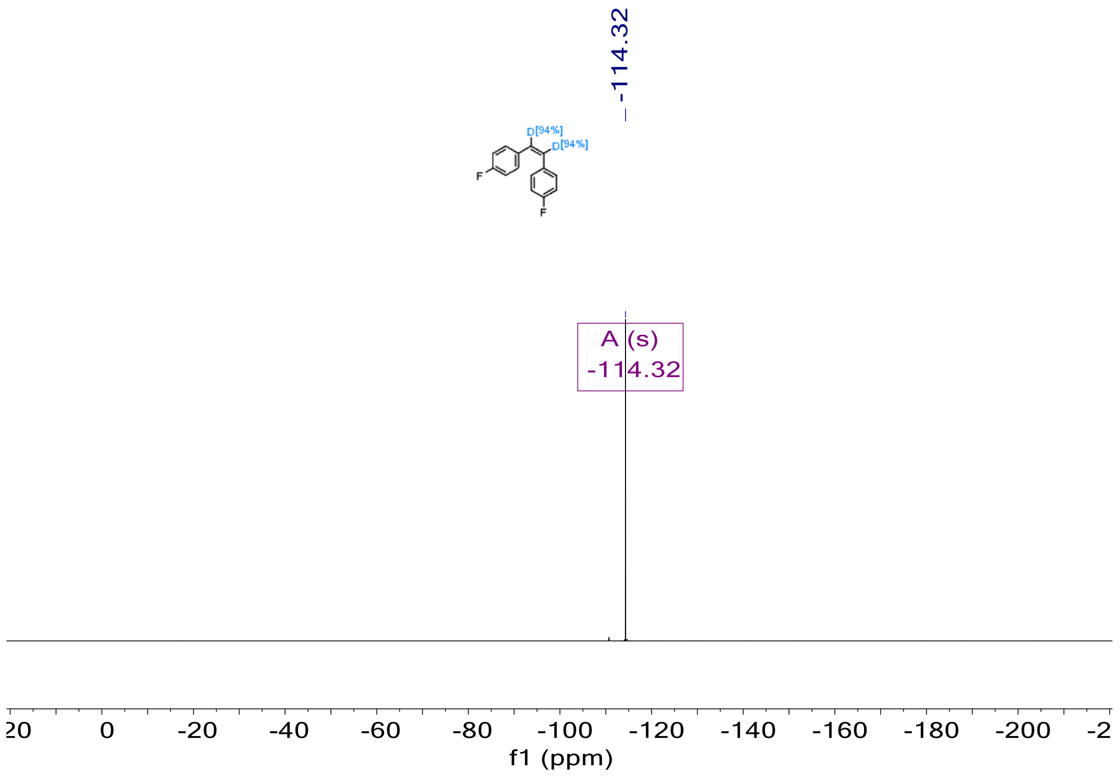

^{19}F NMR (471 MHz, 298 K, Chloroform-*d*) spectra for **15**

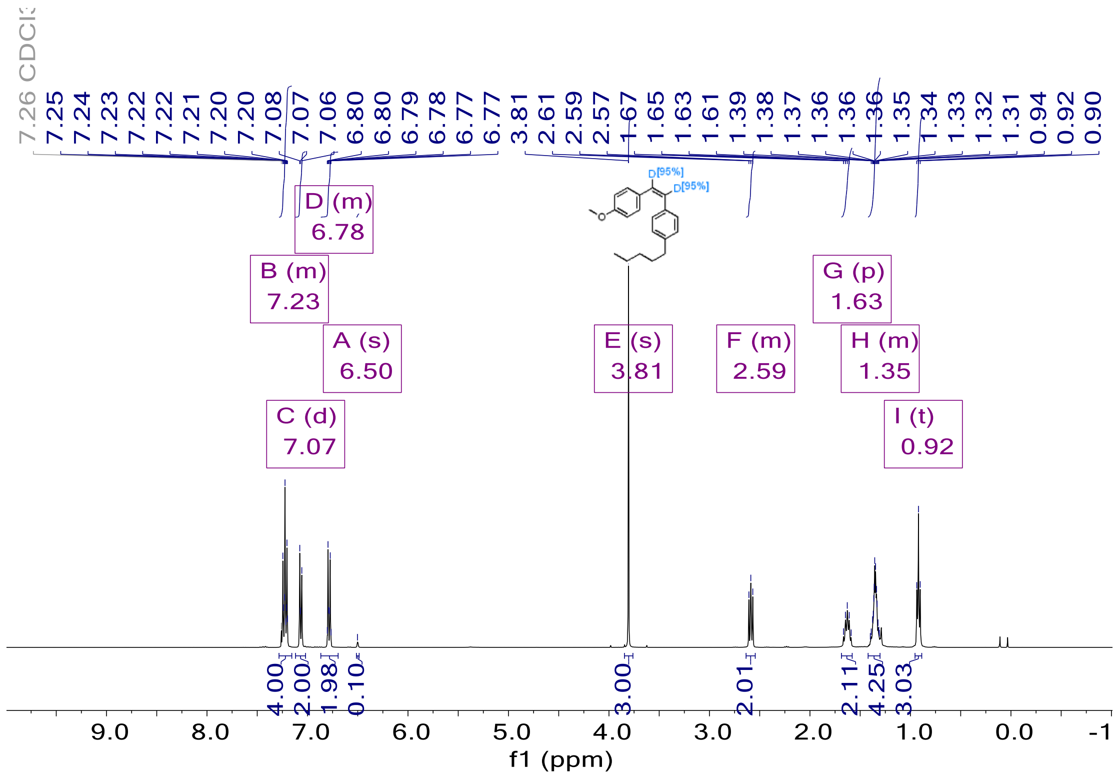

^{1}H NMR (400 MHz, 298 K, Chloroform-*d*) spectra for **16**

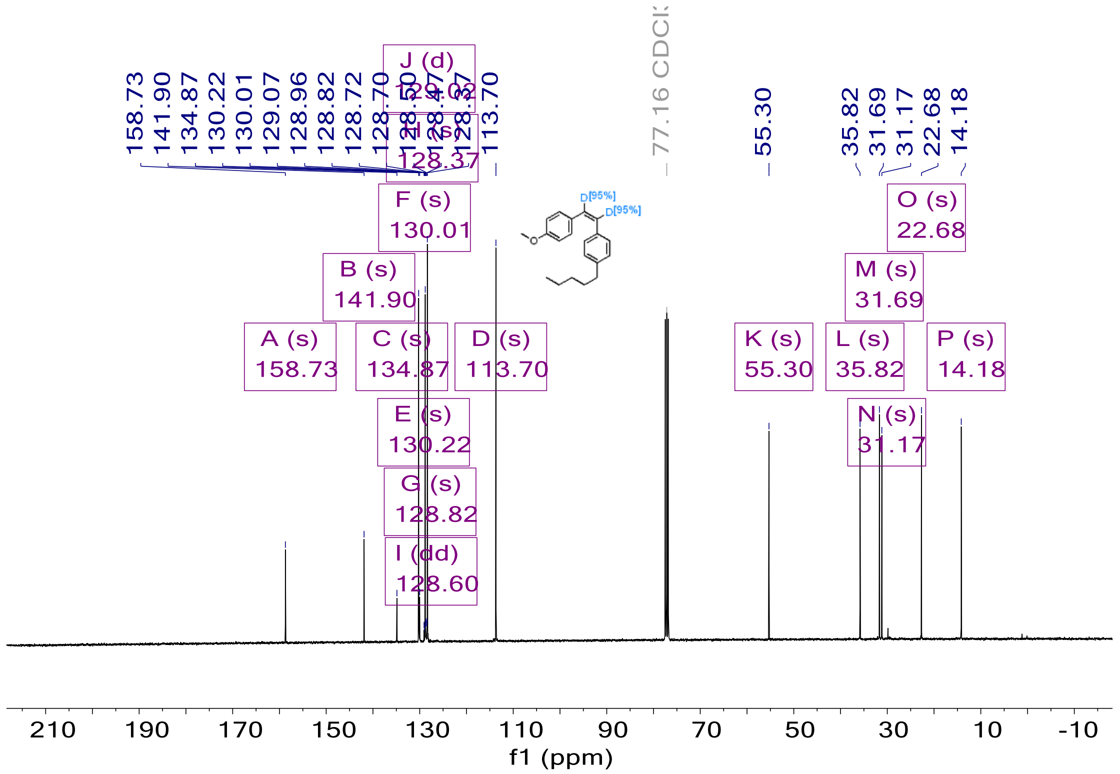

^{13}C NMR (101 MHz, 298 K, Chloroform-*d*) spectra for **16**

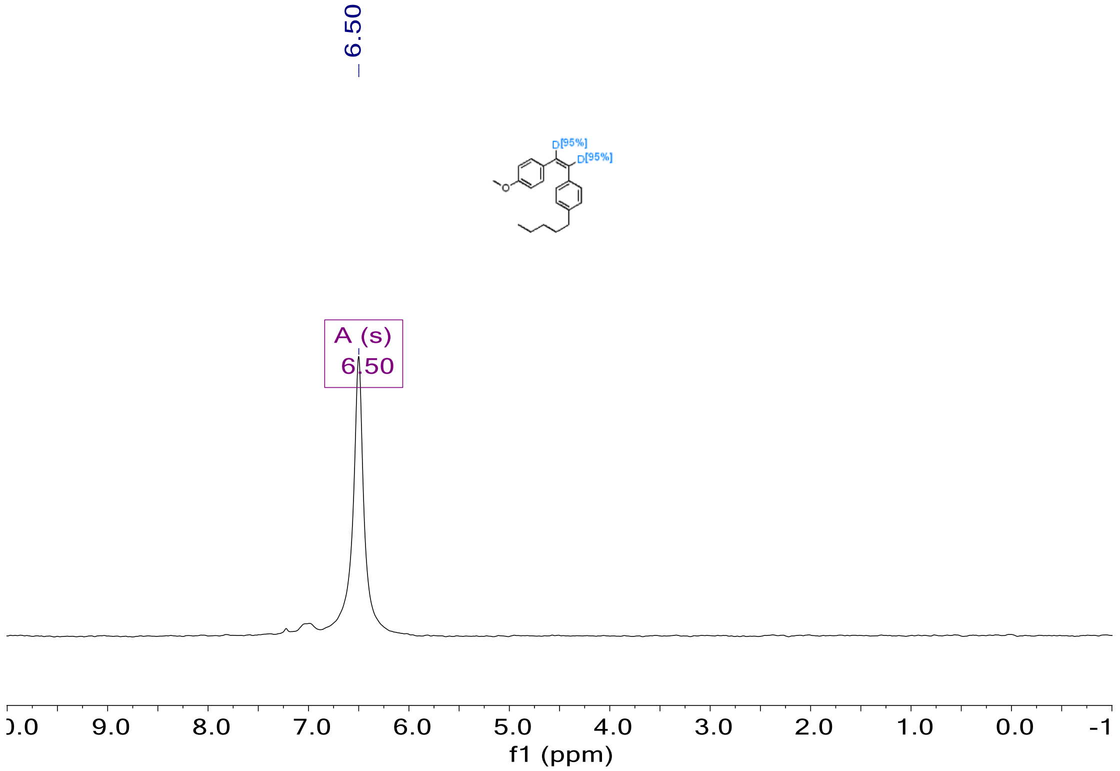

^{2}H NMR (61 MHz, 298 K, Chloroform) spectra for **16**

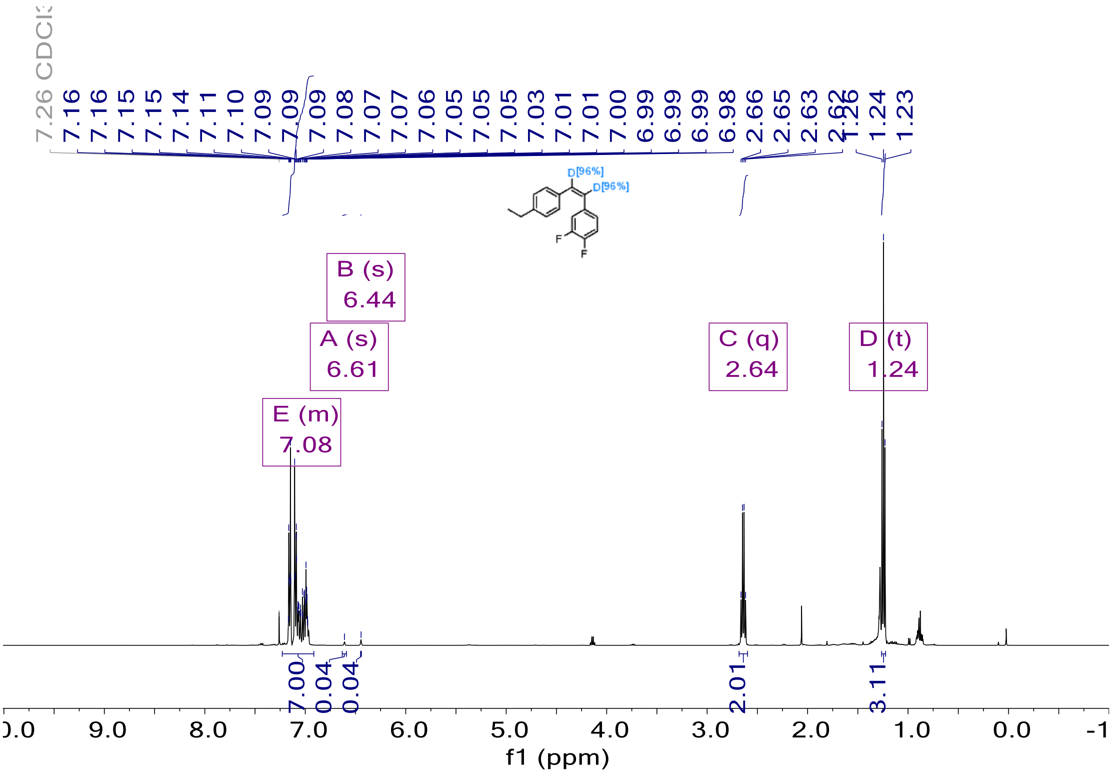

^{1}H NMR (500 MHz, 298 K, Chloroform-*d*) spectra for **17**

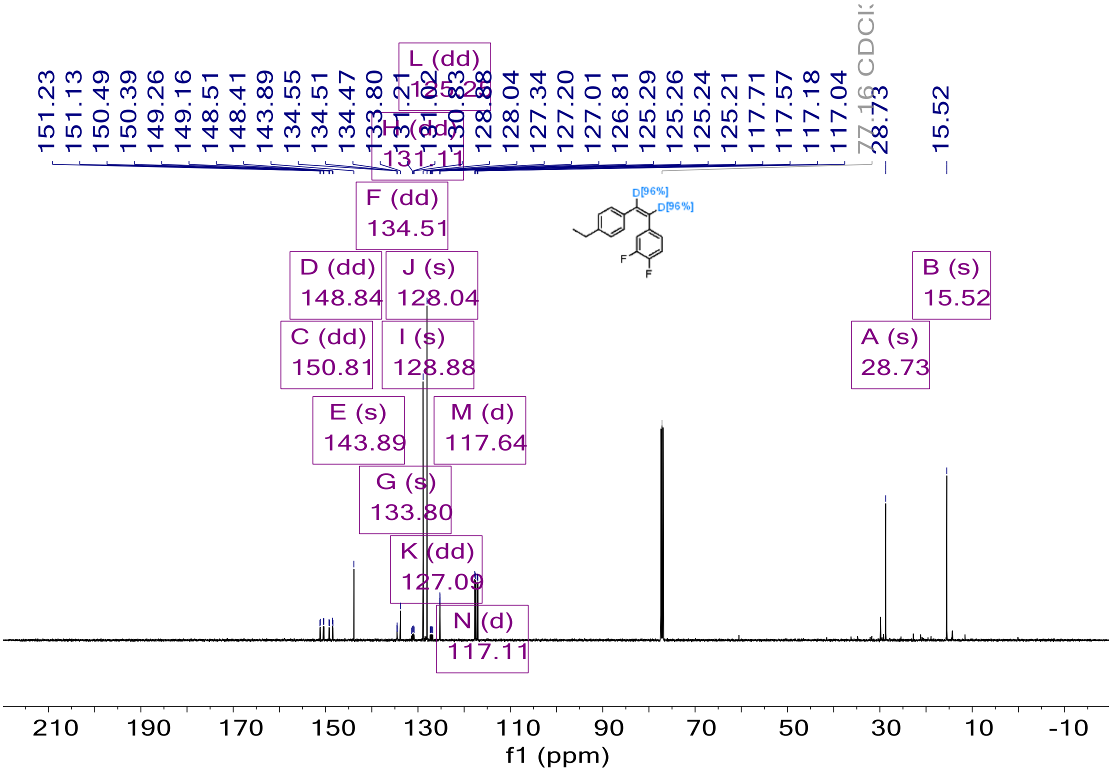

^{13}C NMR (126 MHz, 298 K, Chloroform-*d*) spectra for **17**

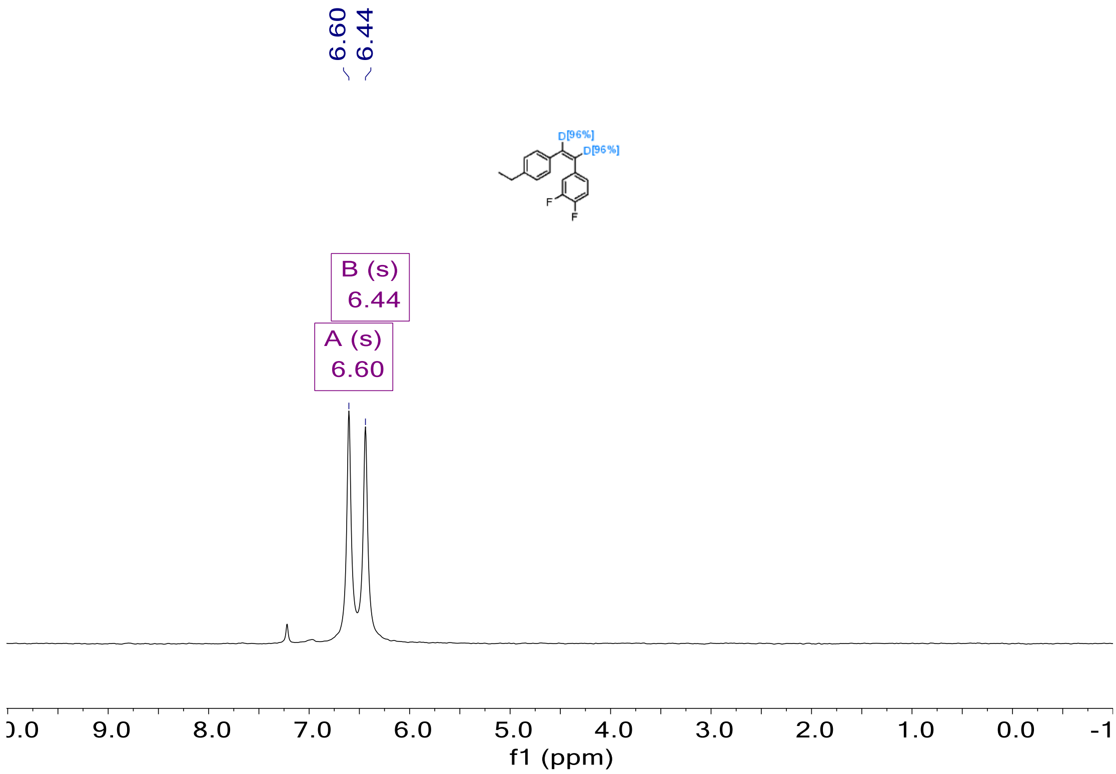

^{2}H NMR (92 MHz, 298 K, Chloroform) spectra for **17**

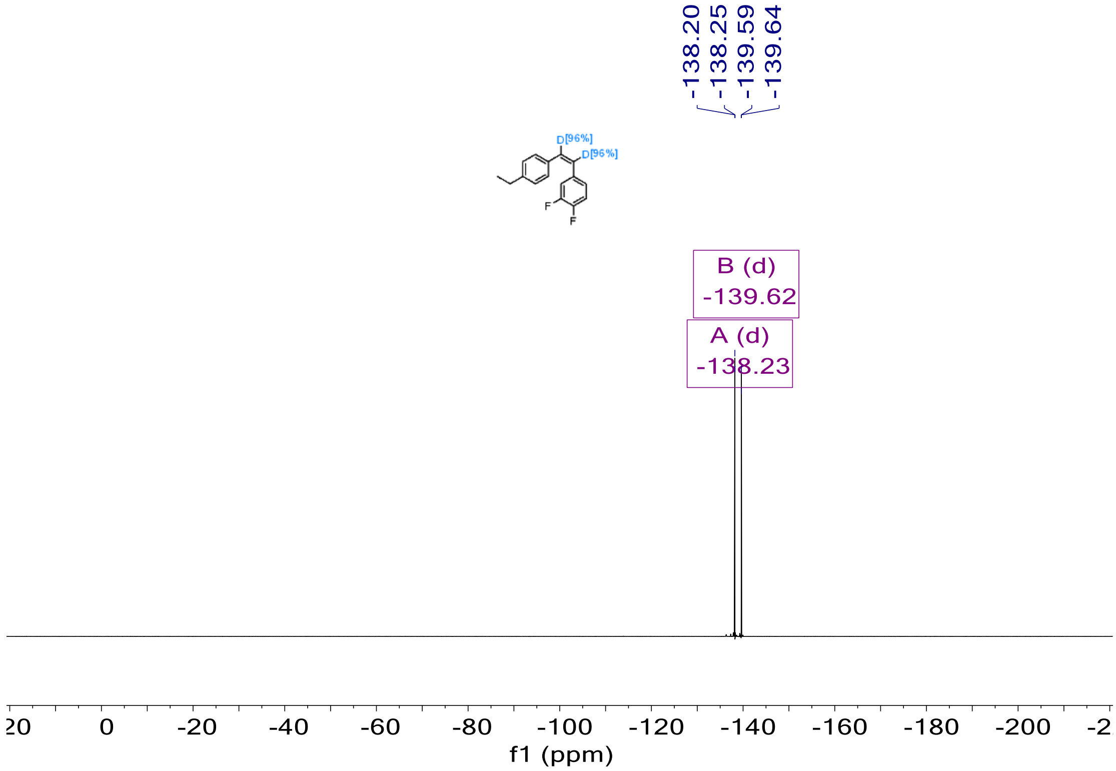

^{19}F NMR (471 MHz, 298 K, Chloroform-*d*) spectra for **17**

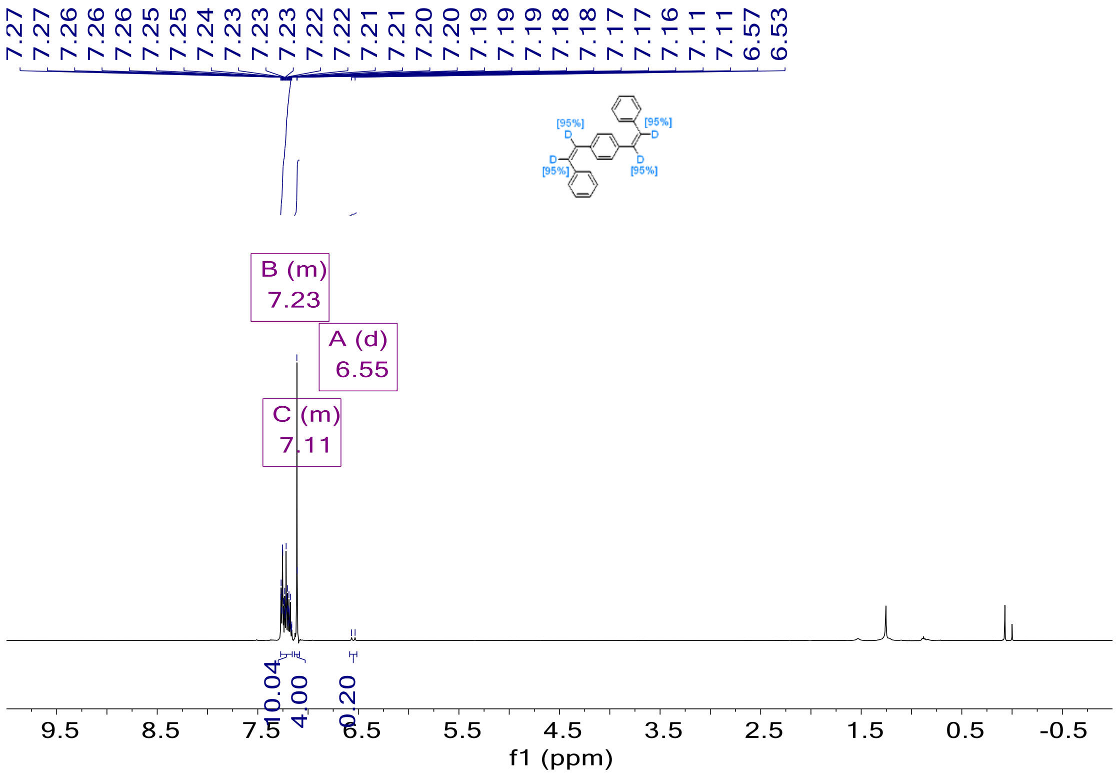

^{1}H NMR (500 MHz, 298 K, Chloroform-*d*) spectra for **18**

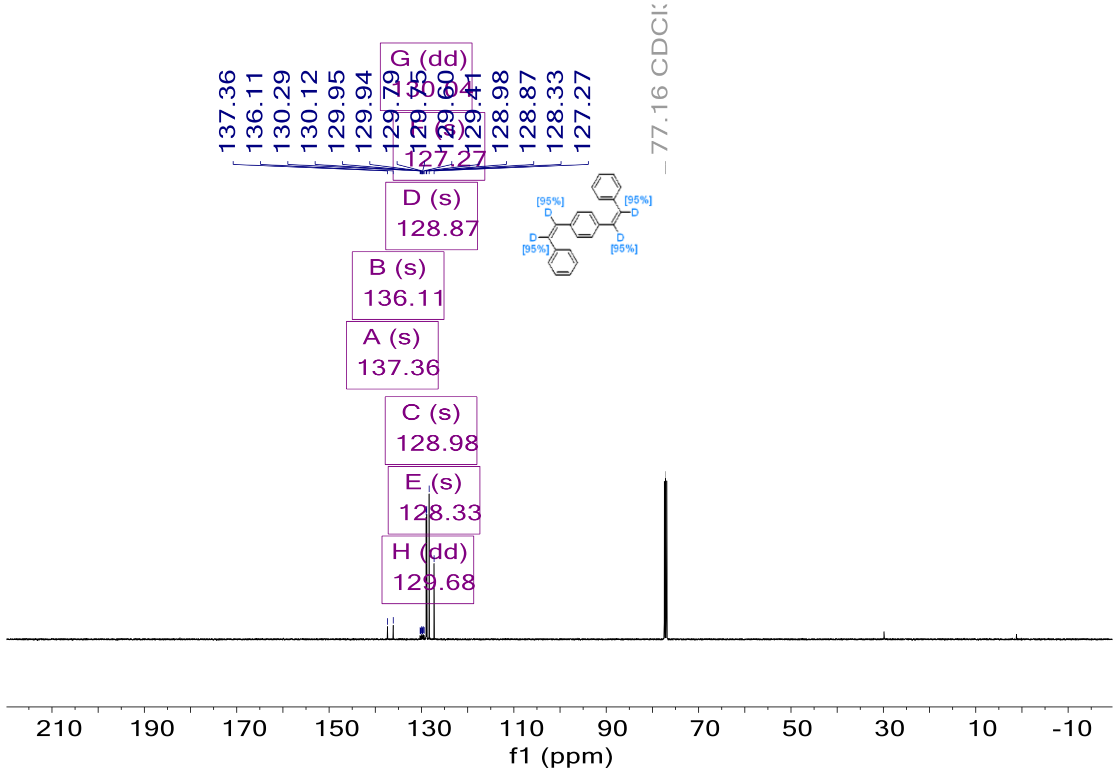

^{13}C NMR (126 MHz, 298 K, Chloroform-*d*) spectra for **18**

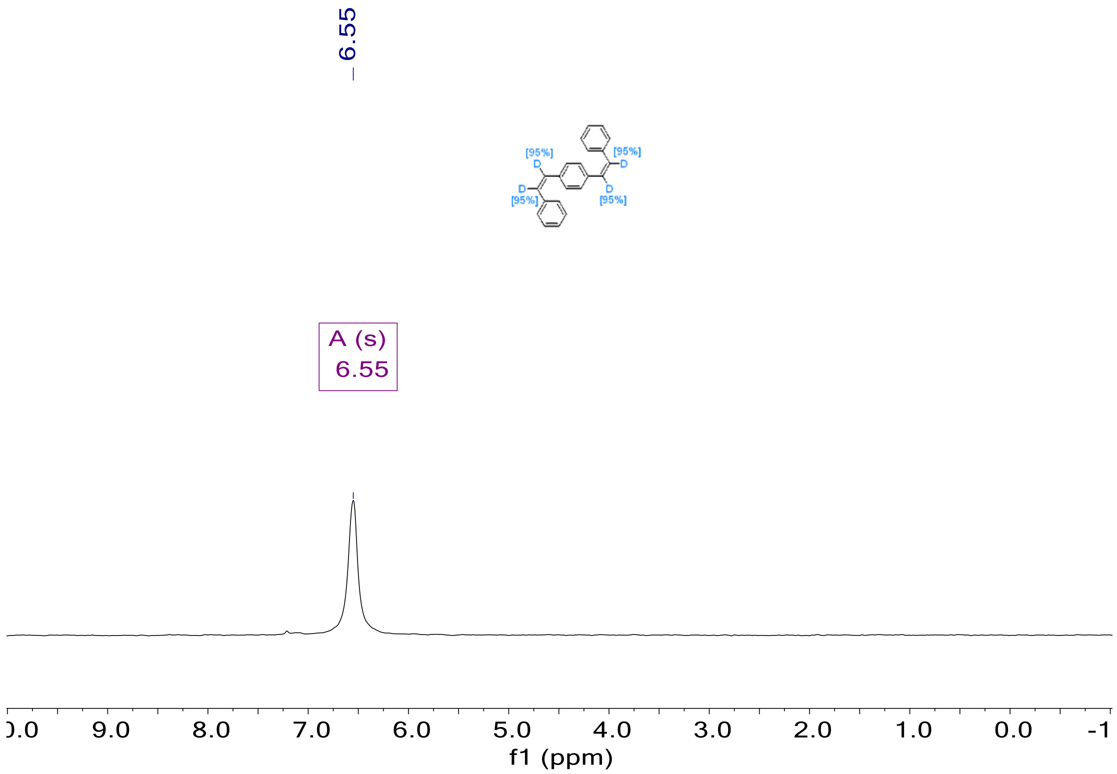

^{2}H NMR (61 MHz, 298 K, Chloroform) spectra for **18**

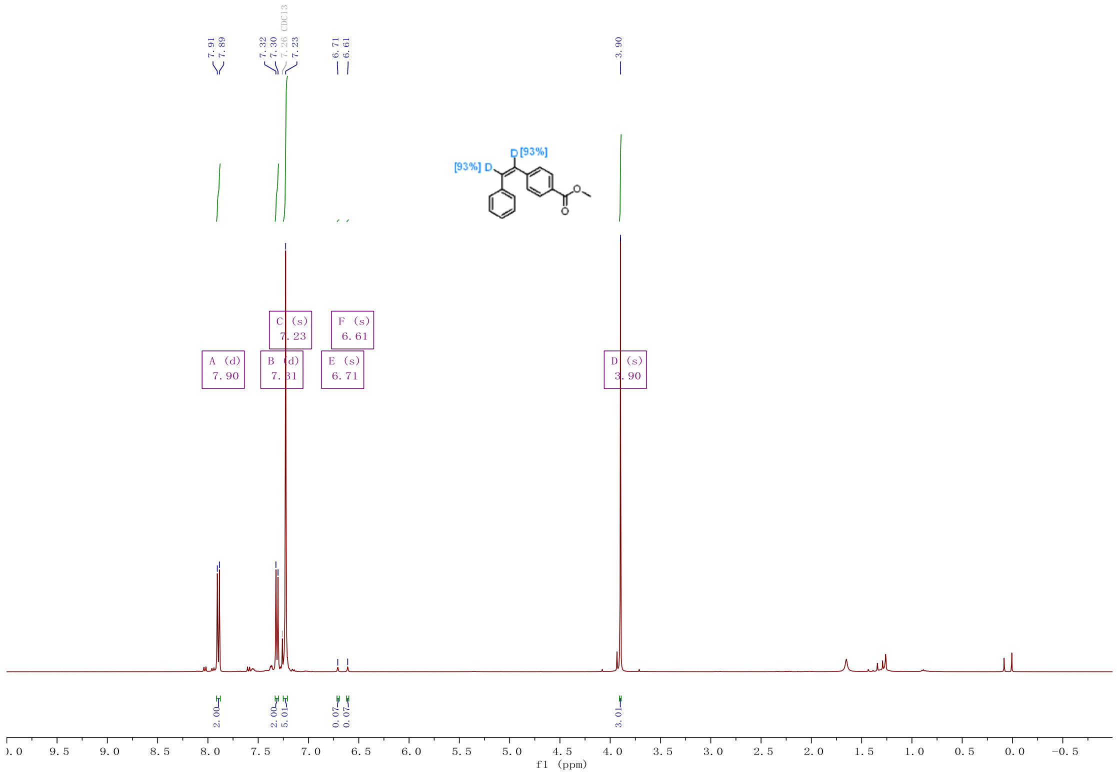

^{1}H NMR (400 MHz, 298 K, Chloroform-*d*) spectra for **19**

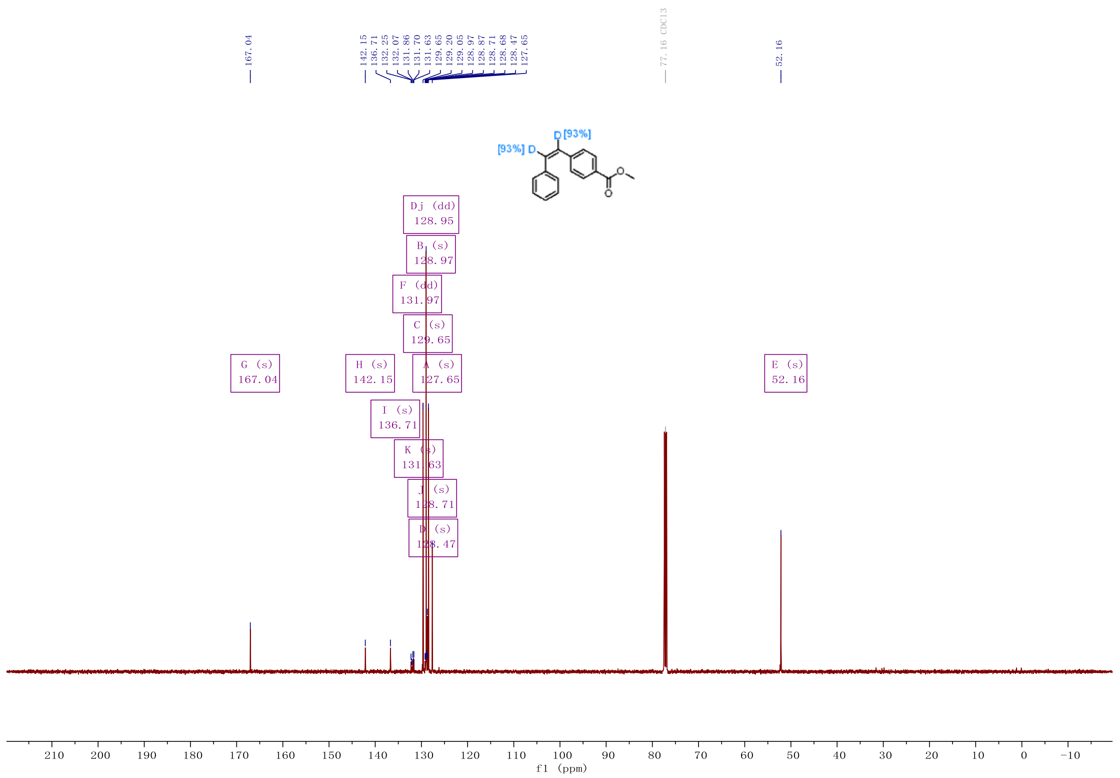

^{13}C NMR (126 MHz, 298 K, Chloroform-*d*) spectra for **19**

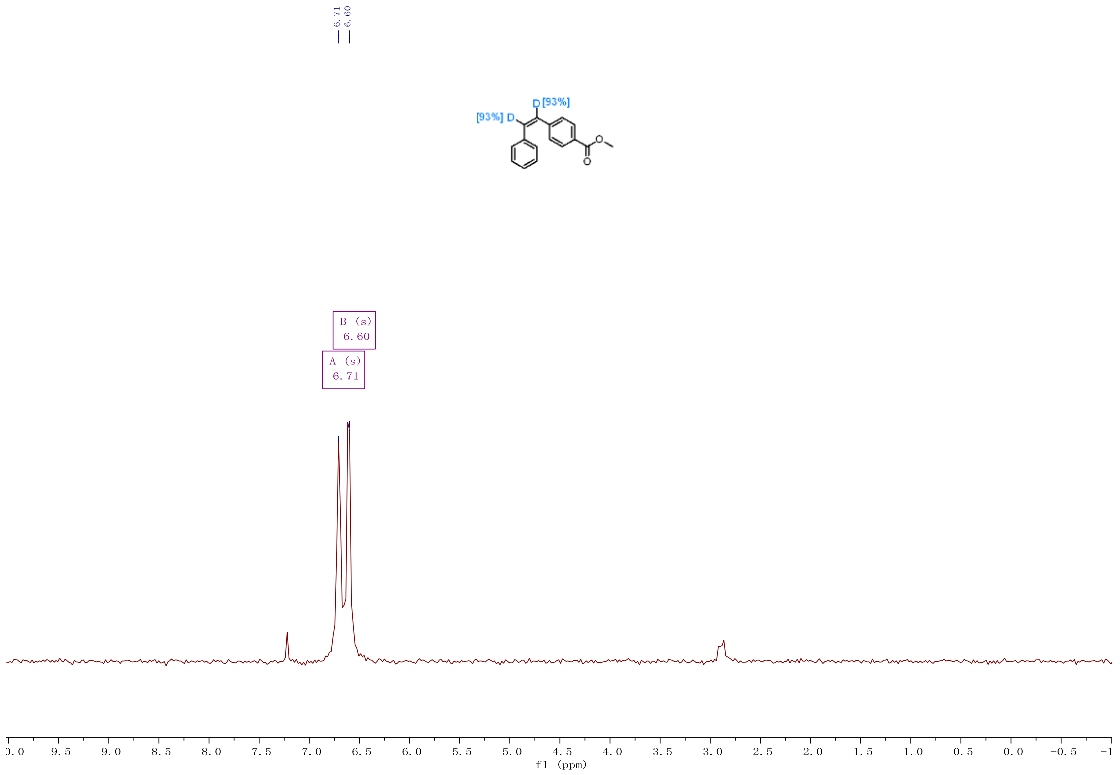

^{2}H NMR (92 MHz, 298 K, Chloroform) spectra for **19**

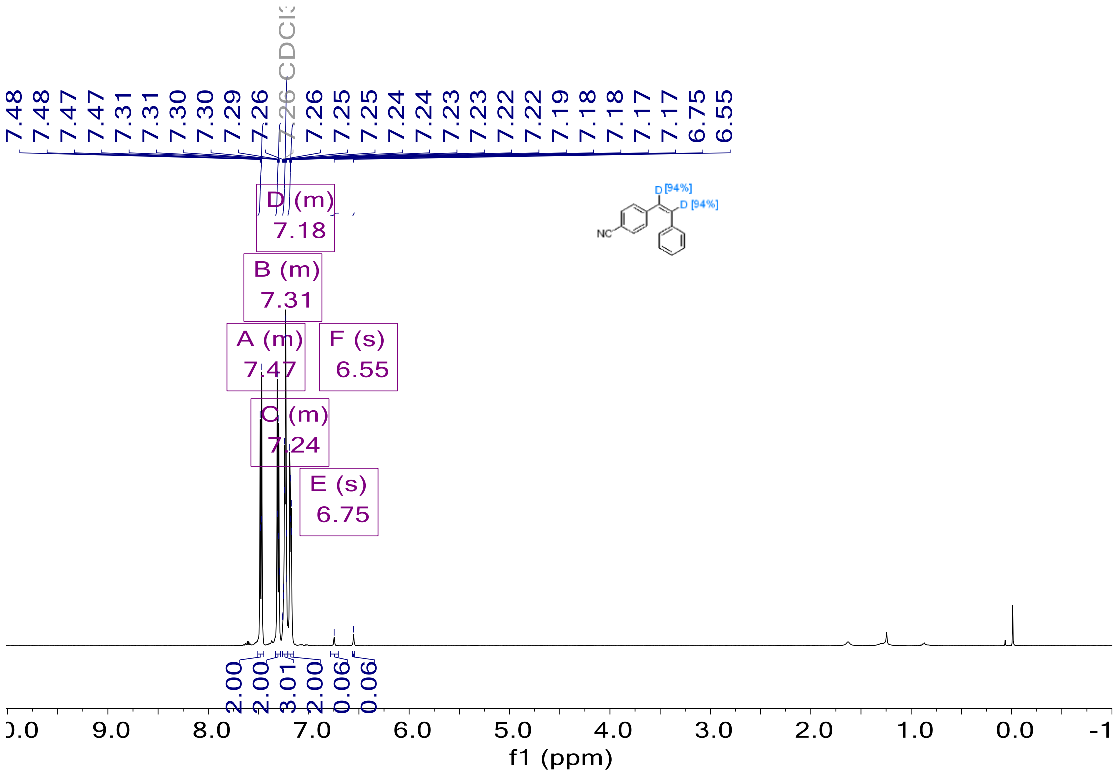

^{1}H NMR (500 MHz, 298 K, Chloroform-*d*) spectra for **20**

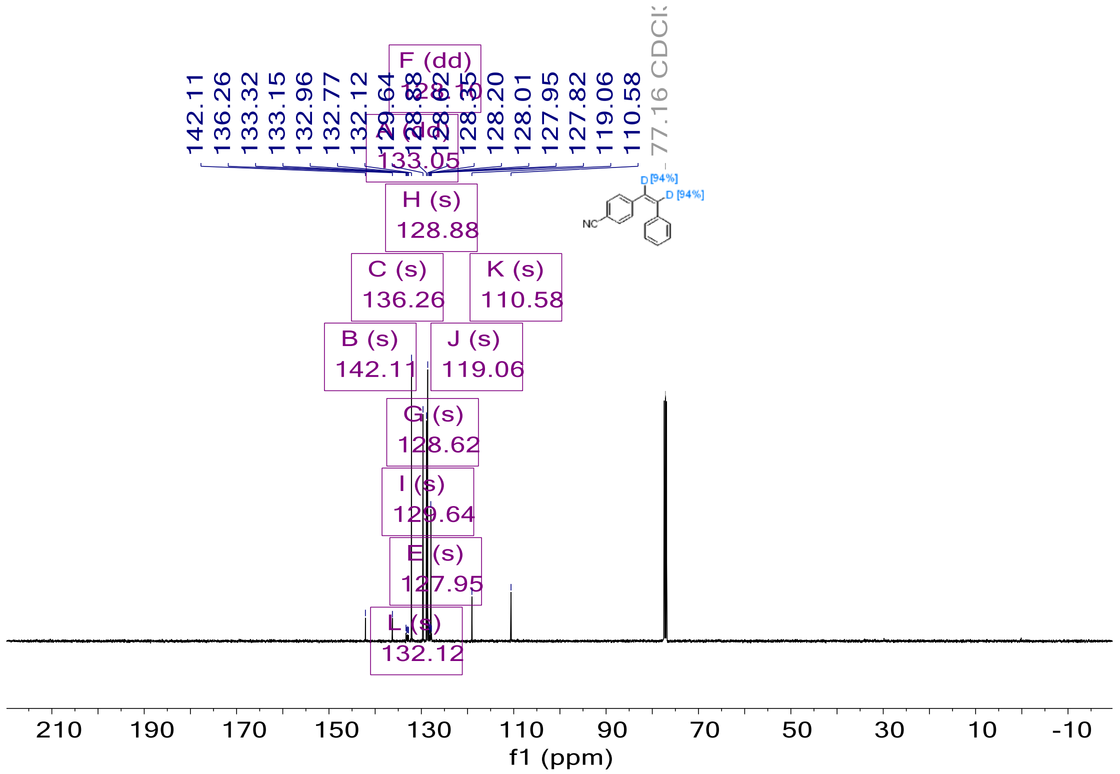

^{13}C NMR (126 MHz, 298 K, Chloroform-*d*) spectra for **20**

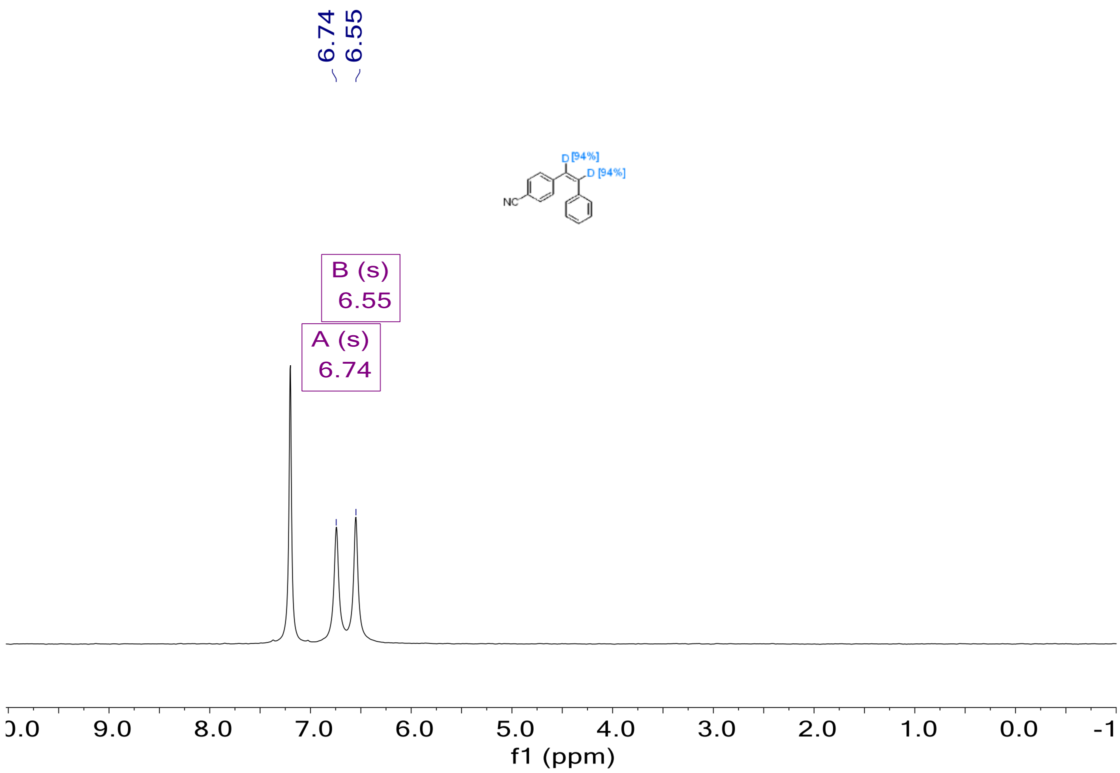

^{2}H NMR (92 MHz, 298 K, Chloroform) spectra for **20**

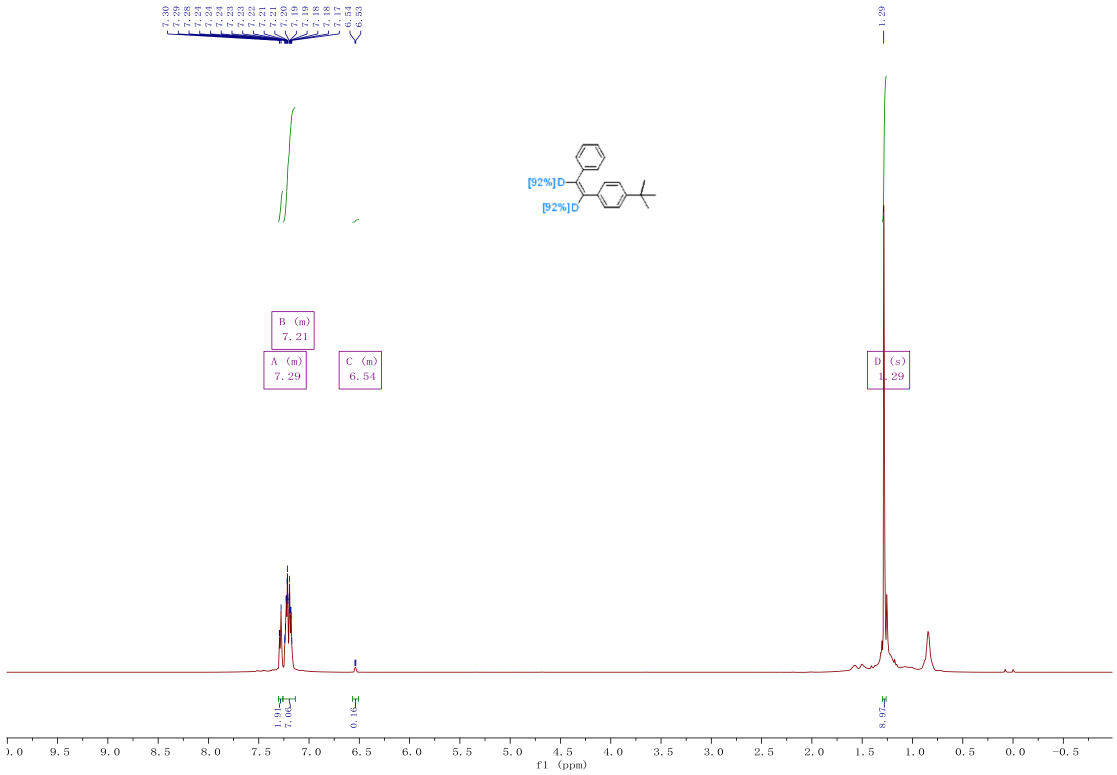

^{1}H NMR (500 MHz, 298 K, Chloroform-*d*) spectra for **21**

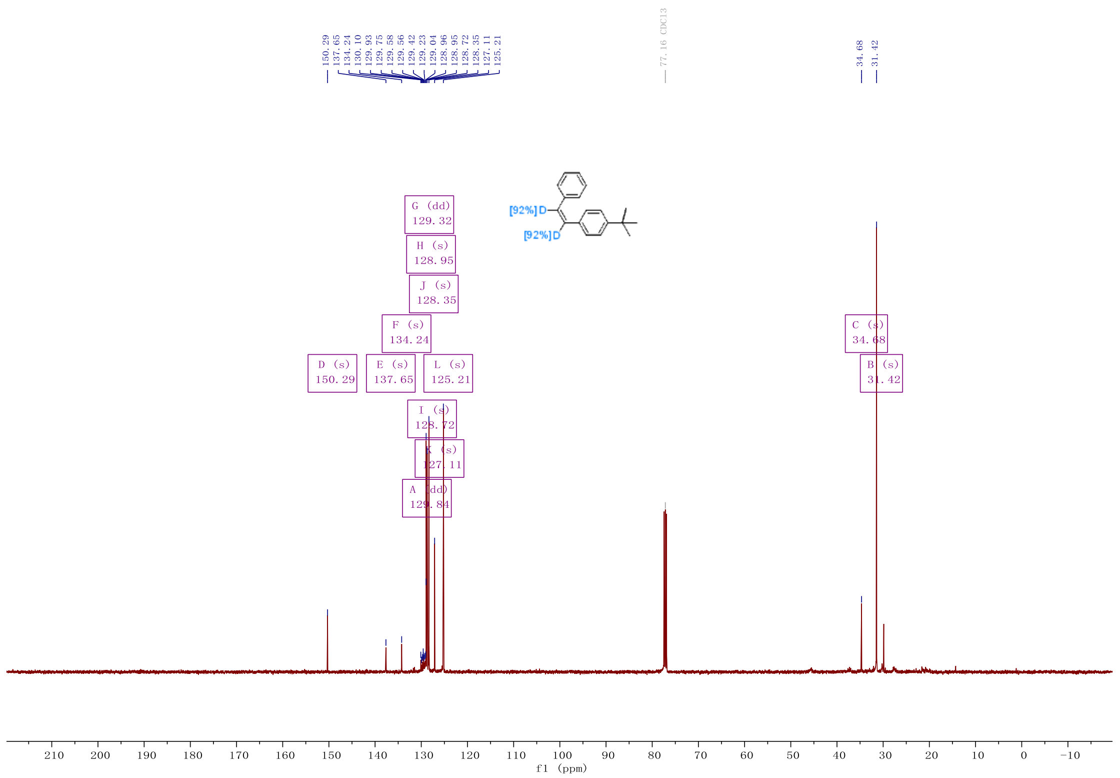

^{13}C NMR (126 MHz, 298 K, Chloroform-*d*) spectra for **21**

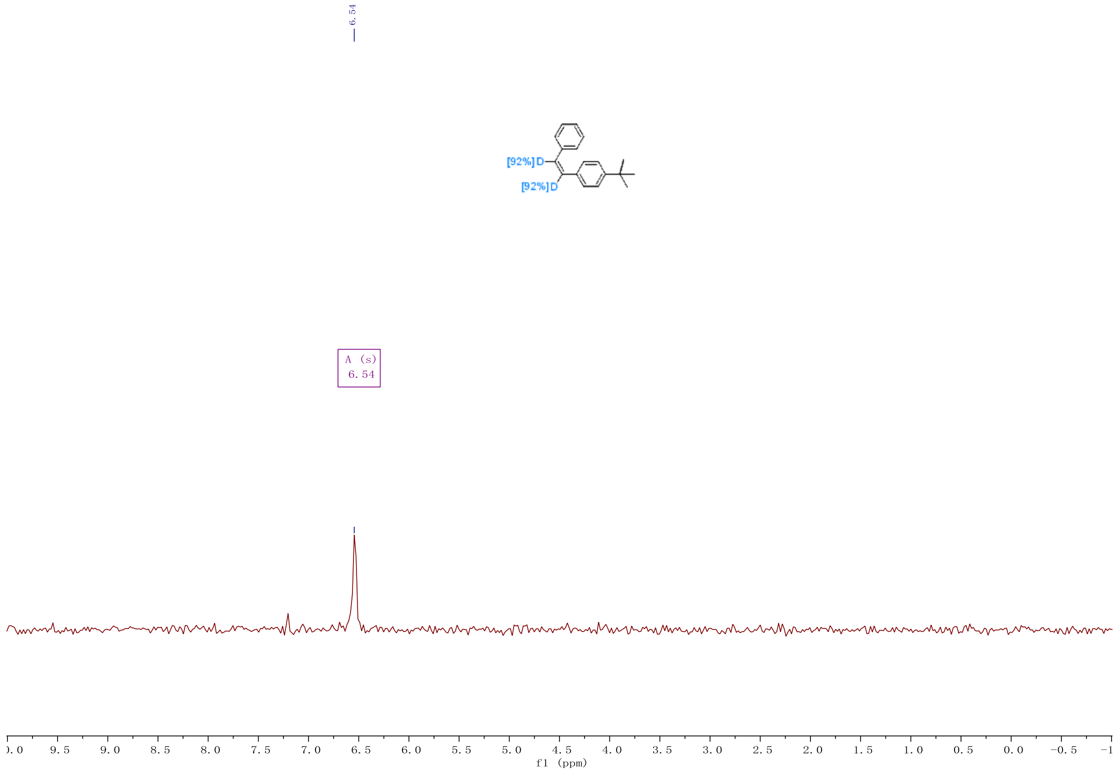

^{2}H NMR (92 MHz, 298 K, Chloroform) spectra for **21**

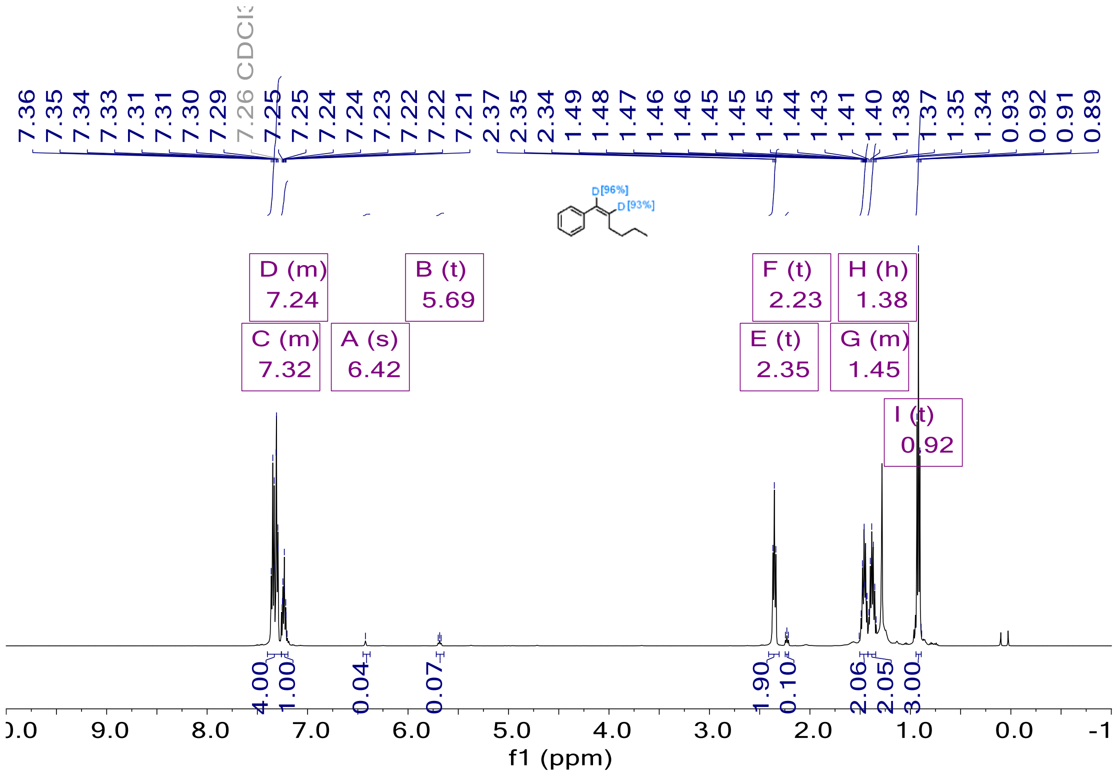

^{1}H NMR (500 MHz, 298 K, Chloroform-*d*) spectra for **22**

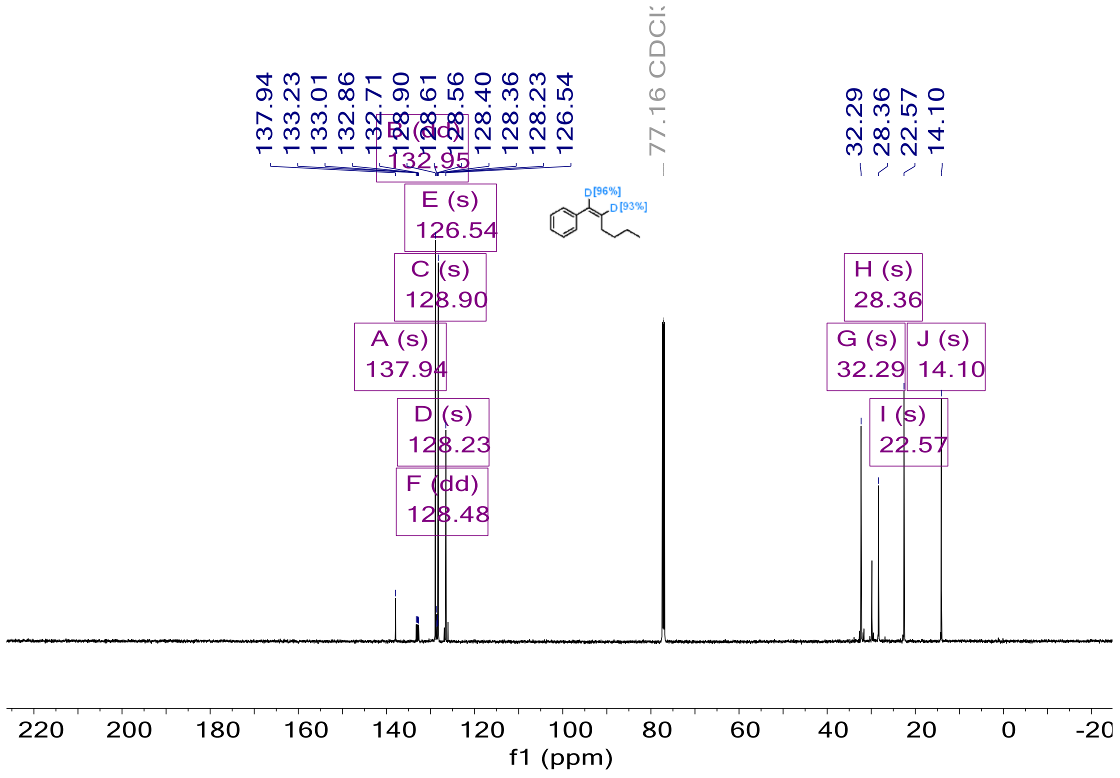

^{13}C NMR (151 MHz, 298 K, Chloroform-*d*) spectra for **22**

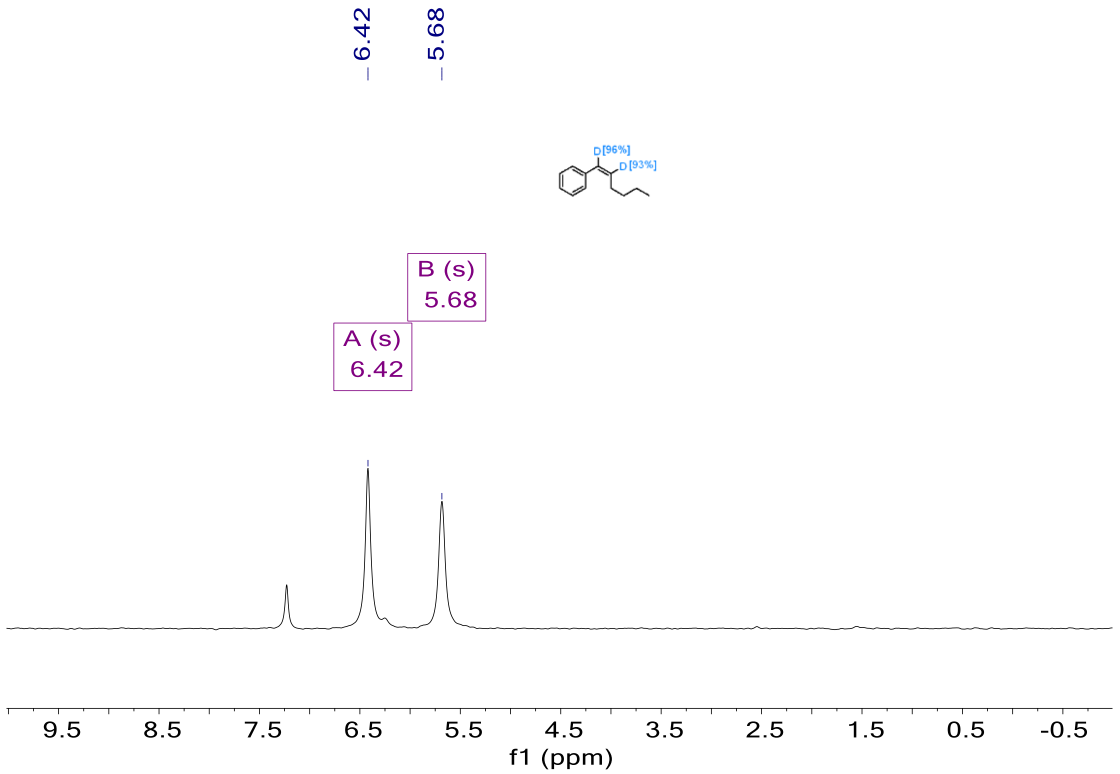

^{2}H NMR (61 MHz, 298 K, Chloroform) spectra for **22**

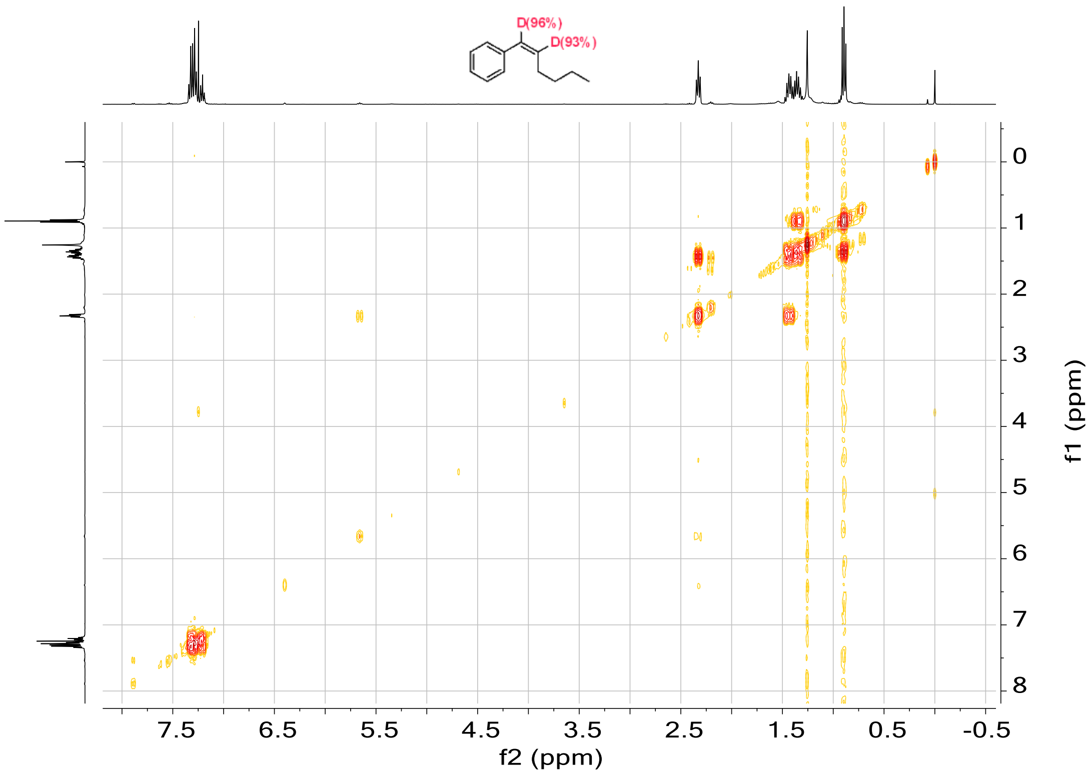

H,H-COSY 90° (500 MHz, 298 K, Chloroform-*d*) spectra for **22**

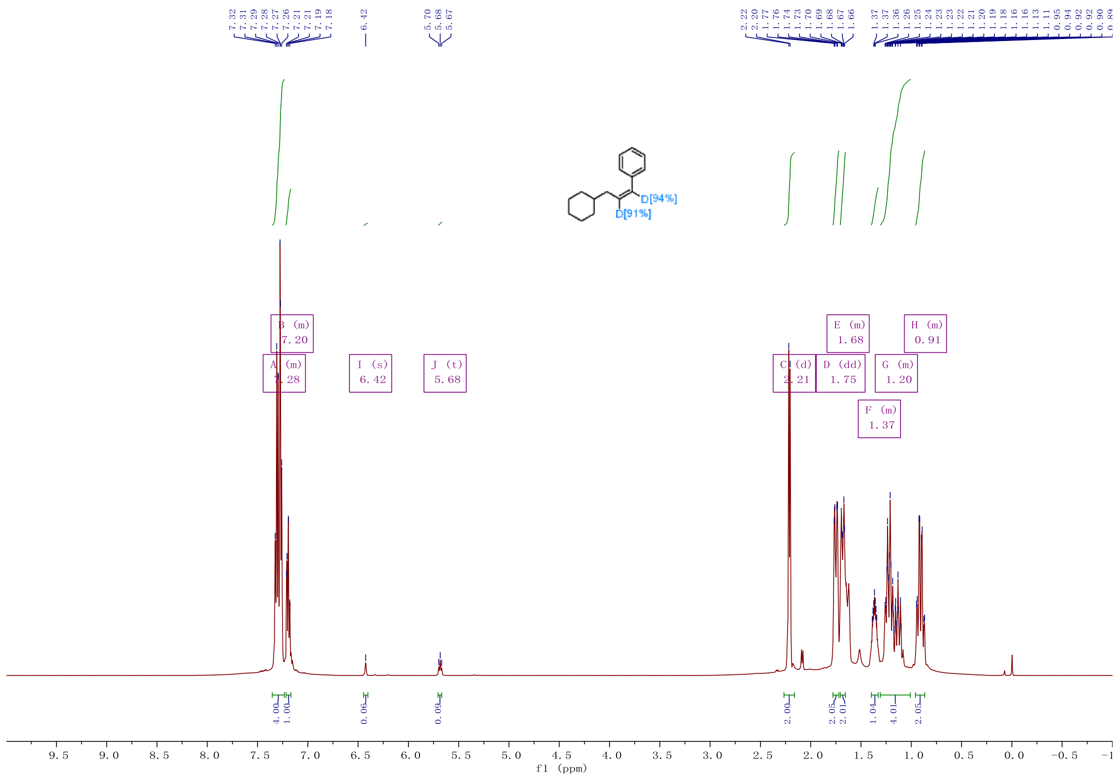

^{1}H NMR (500 MHz, 298 K, Chloroform-*d*) spectra for **23**

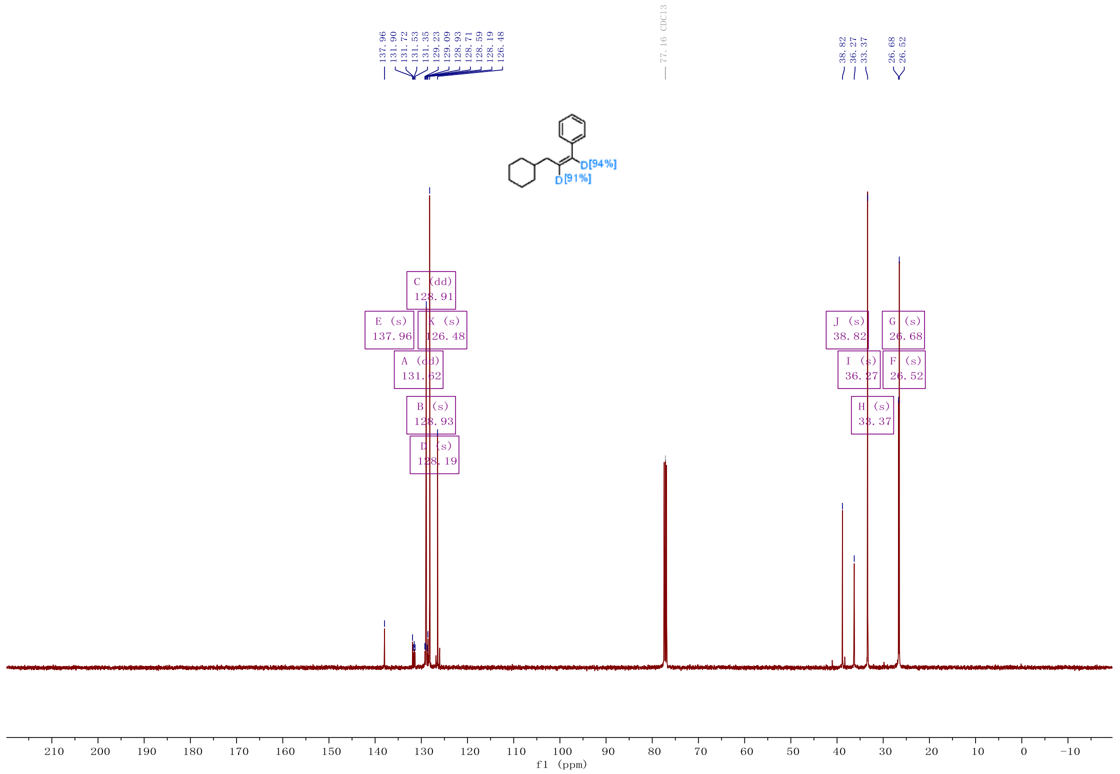

^{13}C NMR (126 MHz, 298 K, Chloroform-*d*) spectra for **23**

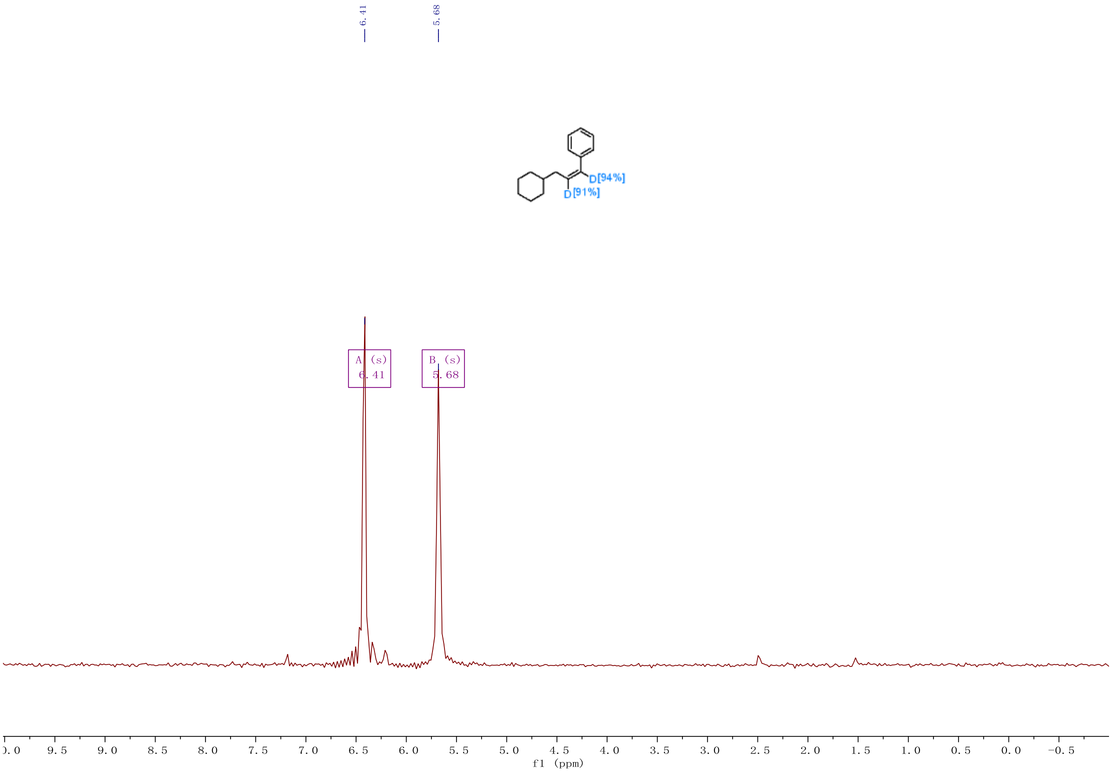

^{2}H NMR (92 MHz, 298 K, Chloroform) spectra for **23**

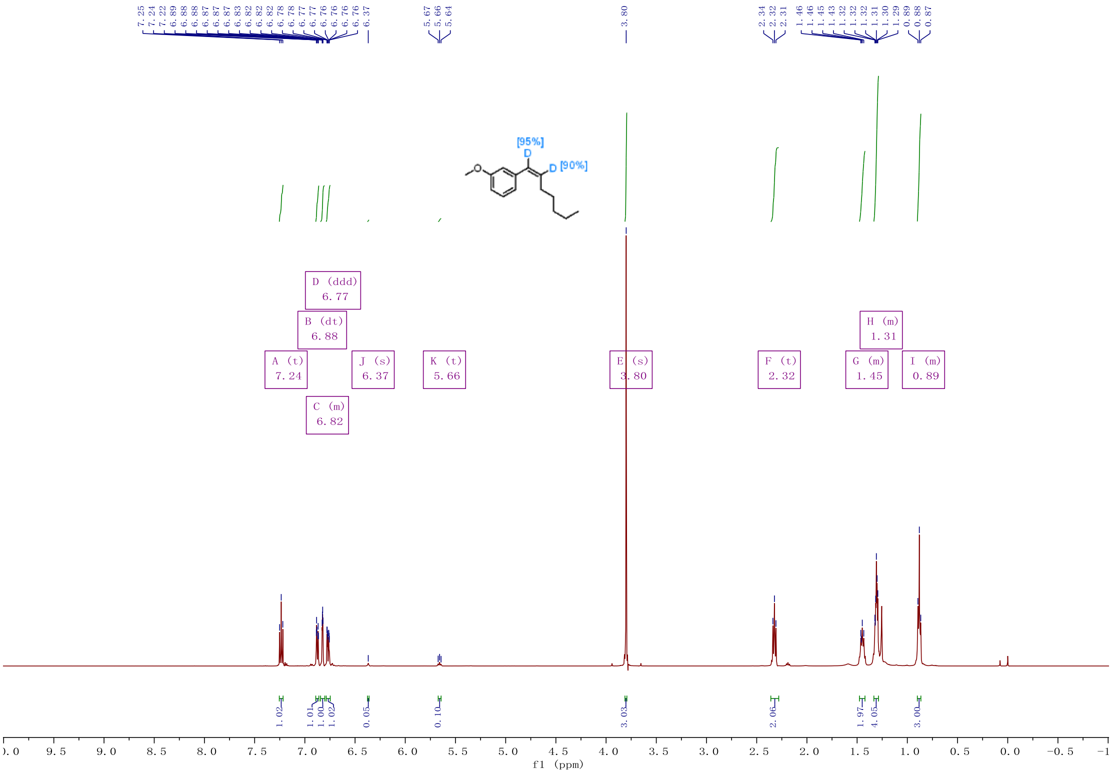

^{1}H NMR (500 MHz, 298 K, Chloroform-*d*) spectra for **24**

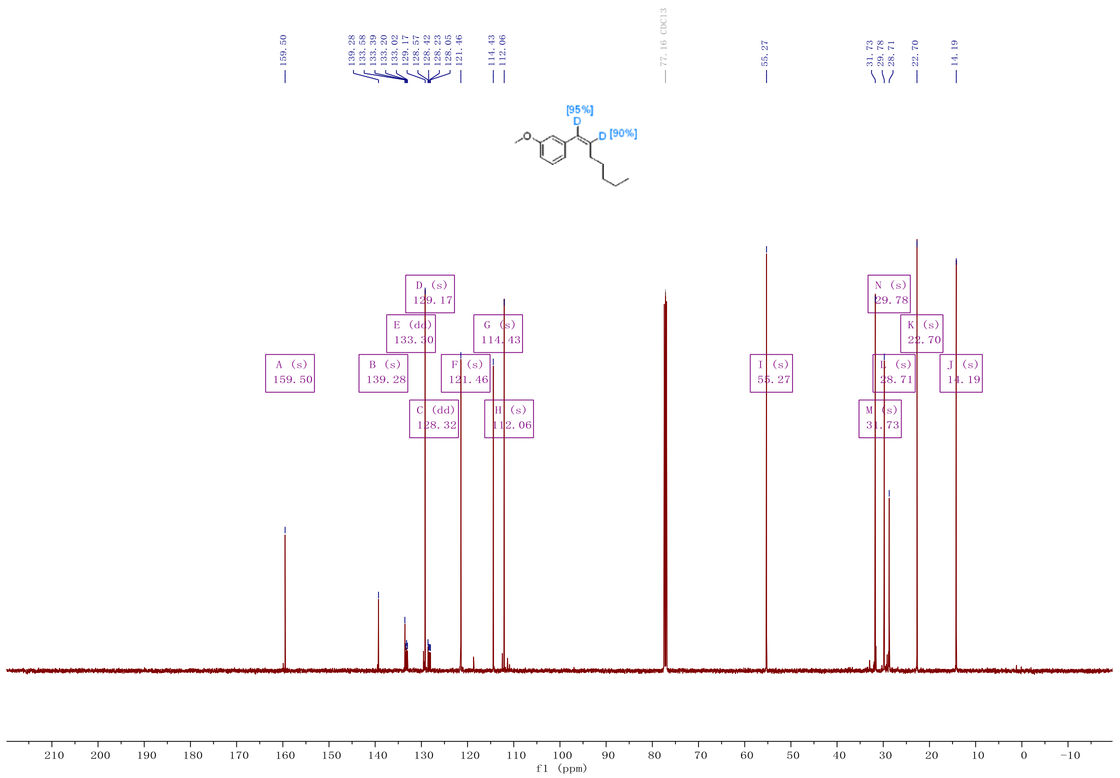

^{13}C NMR (126 MHz, 298 K, Chloroform-*d*) spectra for **24**

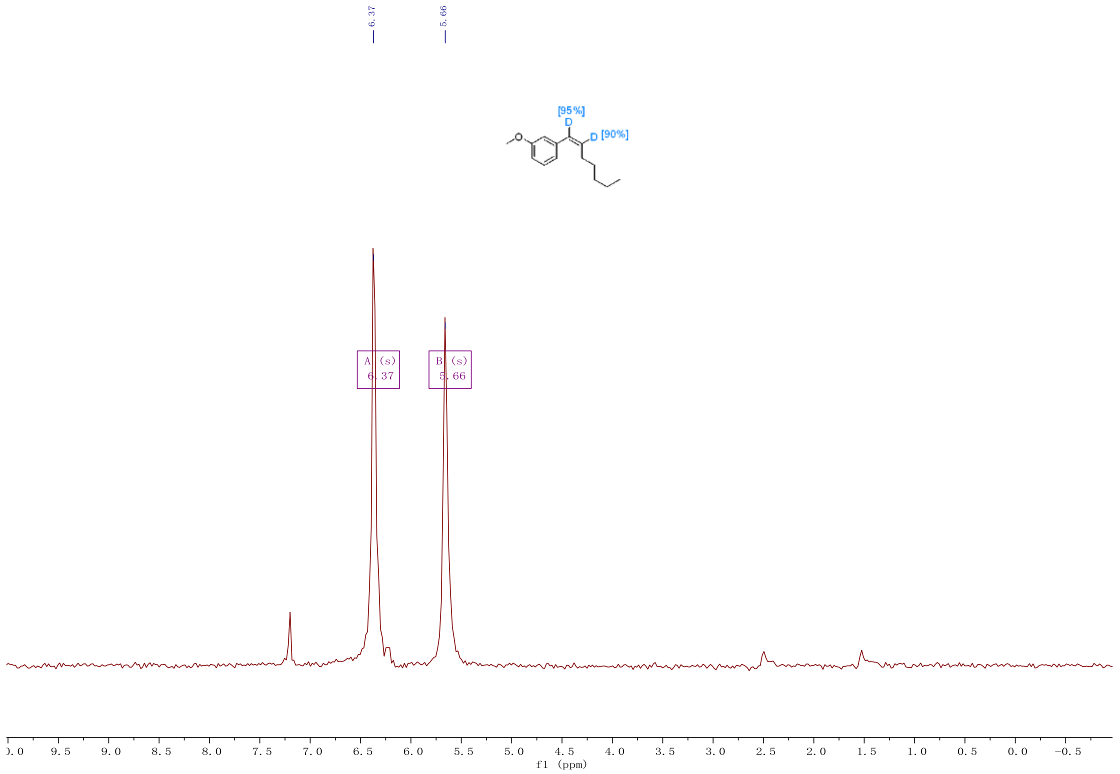

^{2}H NMR (92 MHz, 298 K, Chloroform) spectra for **24**

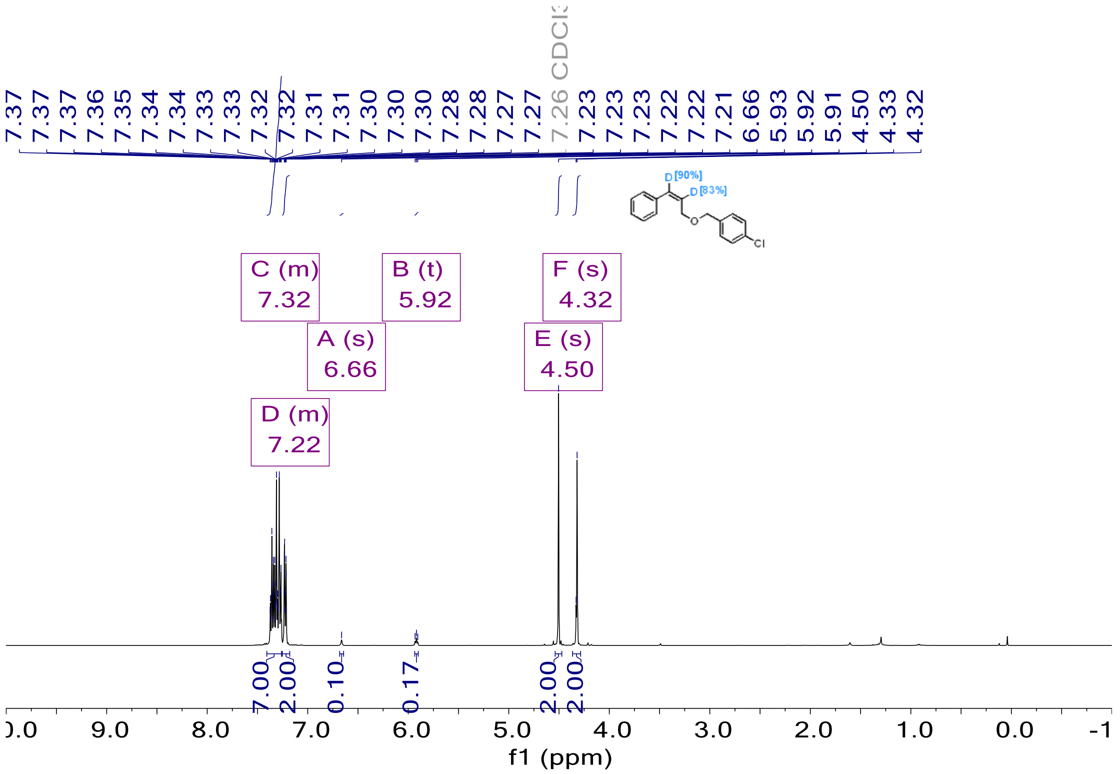

^{1}H NMR (500 MHz, 298 K, Chloroform-*d*) spectra for **25**

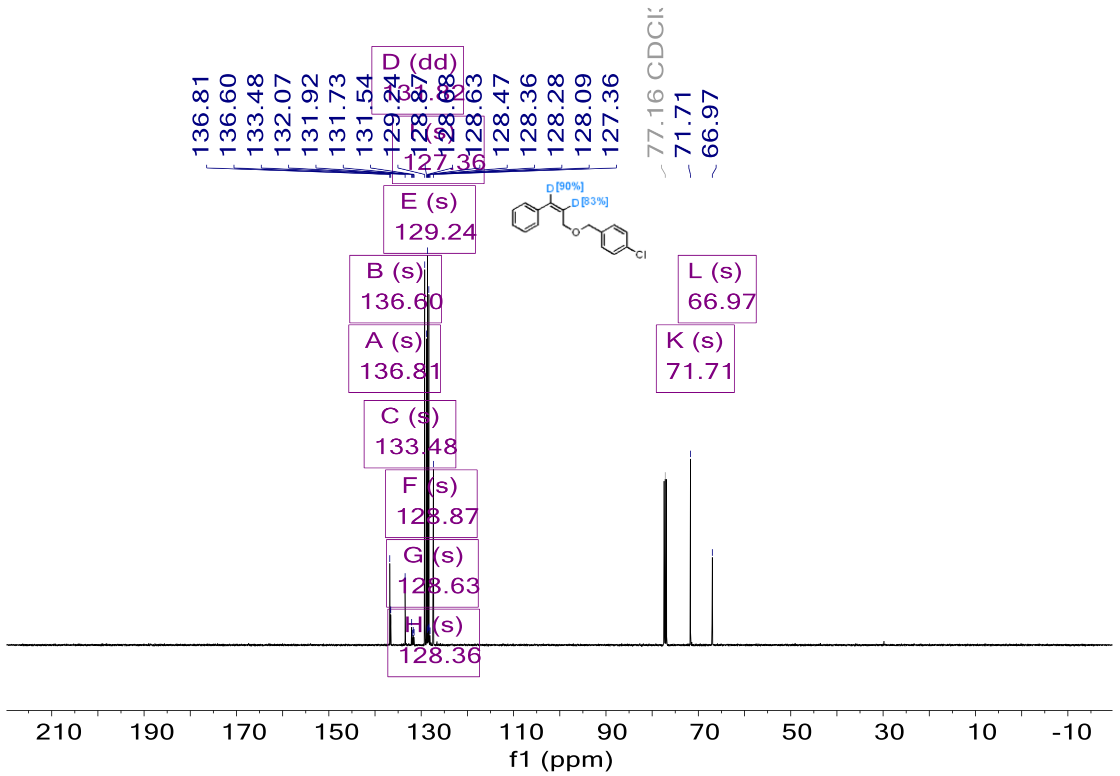

^{13}C NMR (126 MHz, 298 K, Chloroform-*d*) spectra for **25**

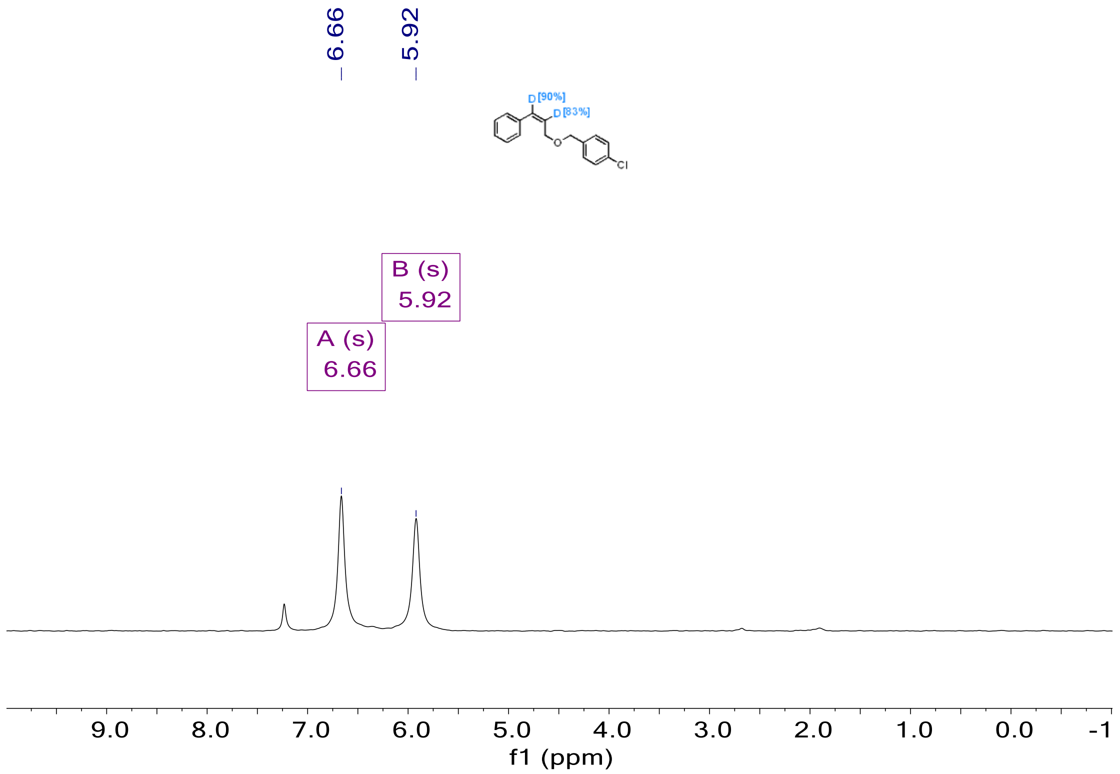

^{2}H NMR (61 MHz, 298 K, Chloroform) spectra for **25**

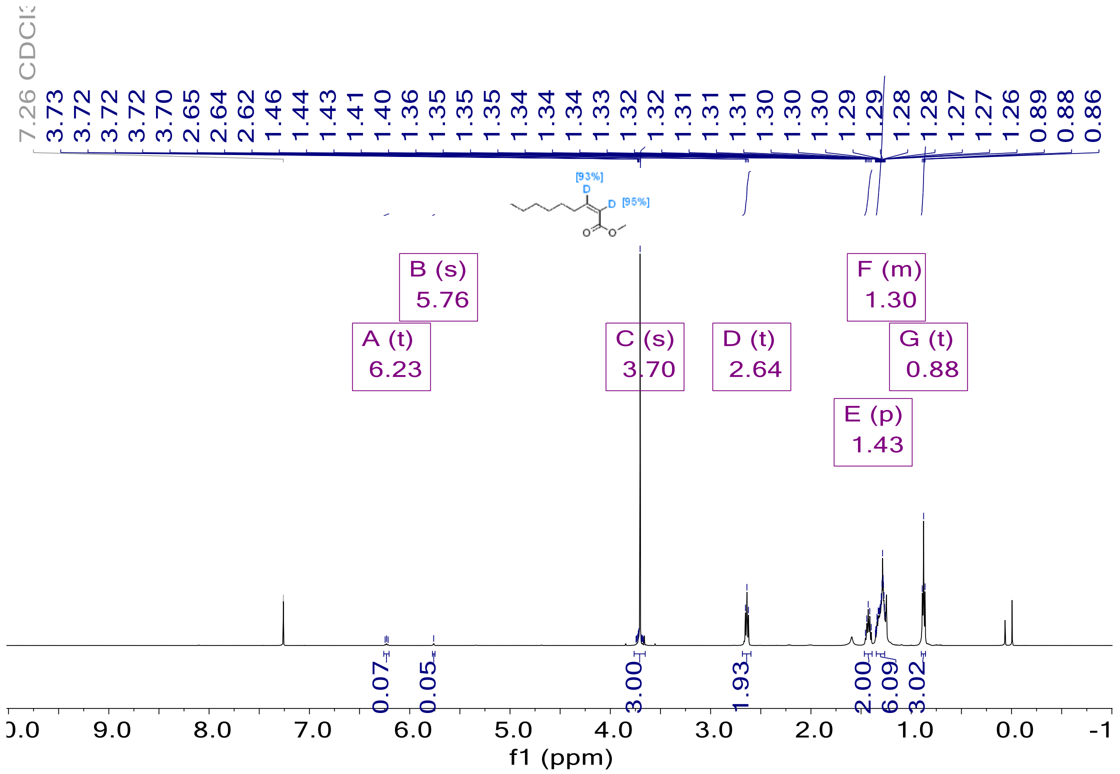

^{1}H NMR (500 MHz, 298 K, Chloroform-*d*) spectra for **26**

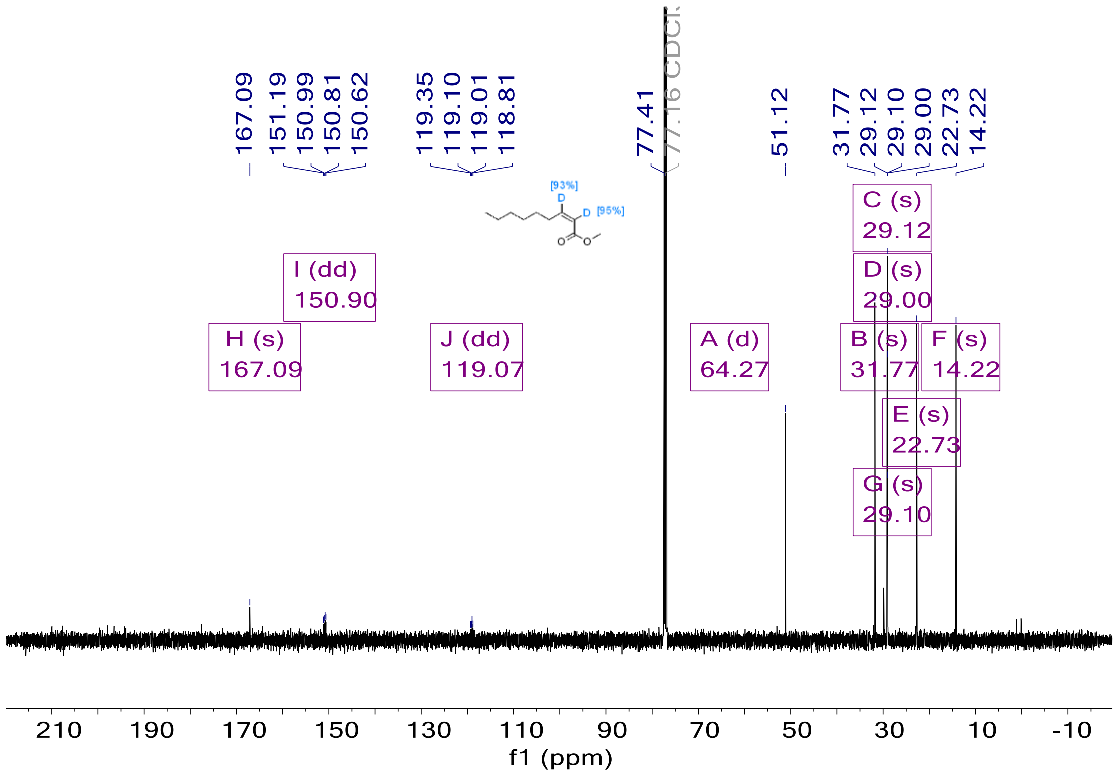

^{13}C NMR (126 MHz, 298 K, Chloroform-*d*) spectra for **26**

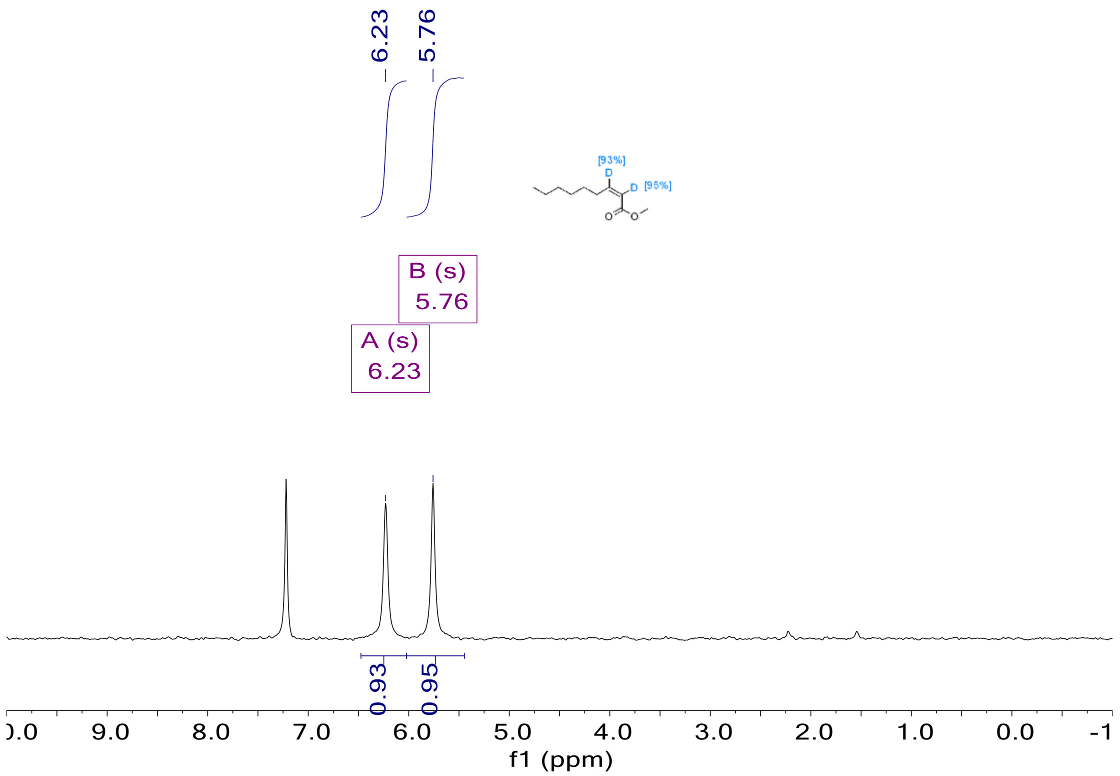

^{2}H NMR (92 MHz, 298 K, Chloroform) spectra for **26**

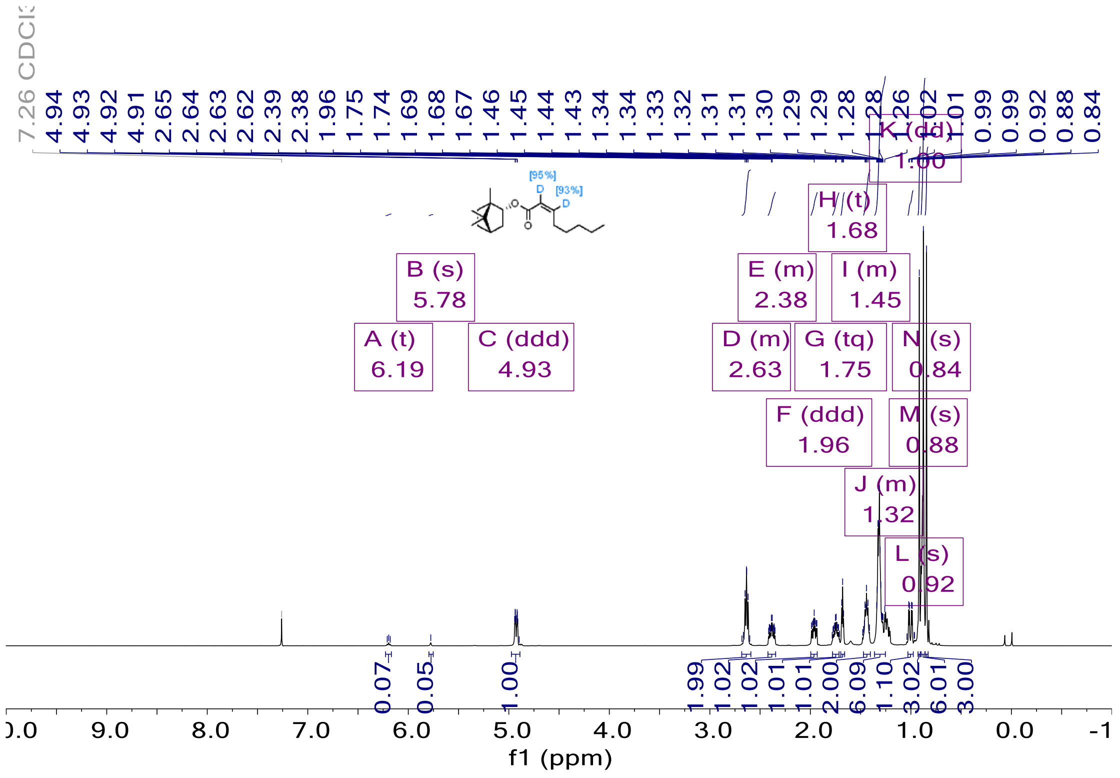

^{1}H NMR (500 MHz, 298 K, Chloroform-*d*) spectra for **27**

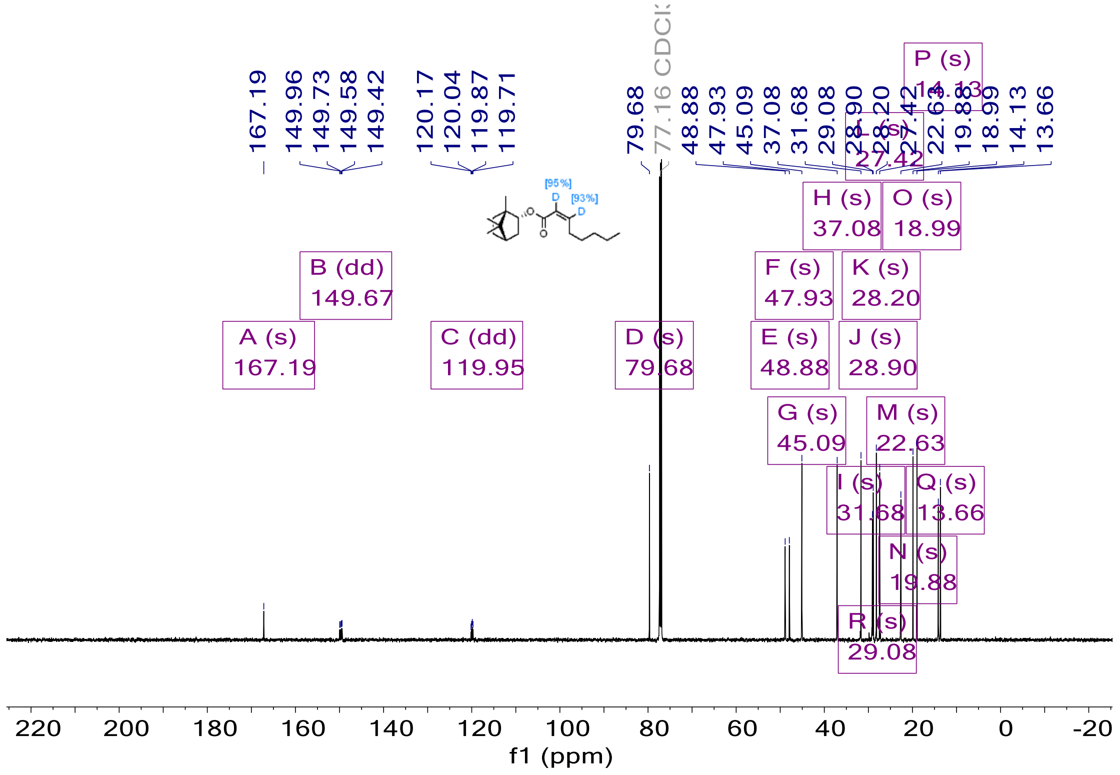

^{13}C NMR (151 MHz, 298 K, Chloroform-*d*) spectra for **27**

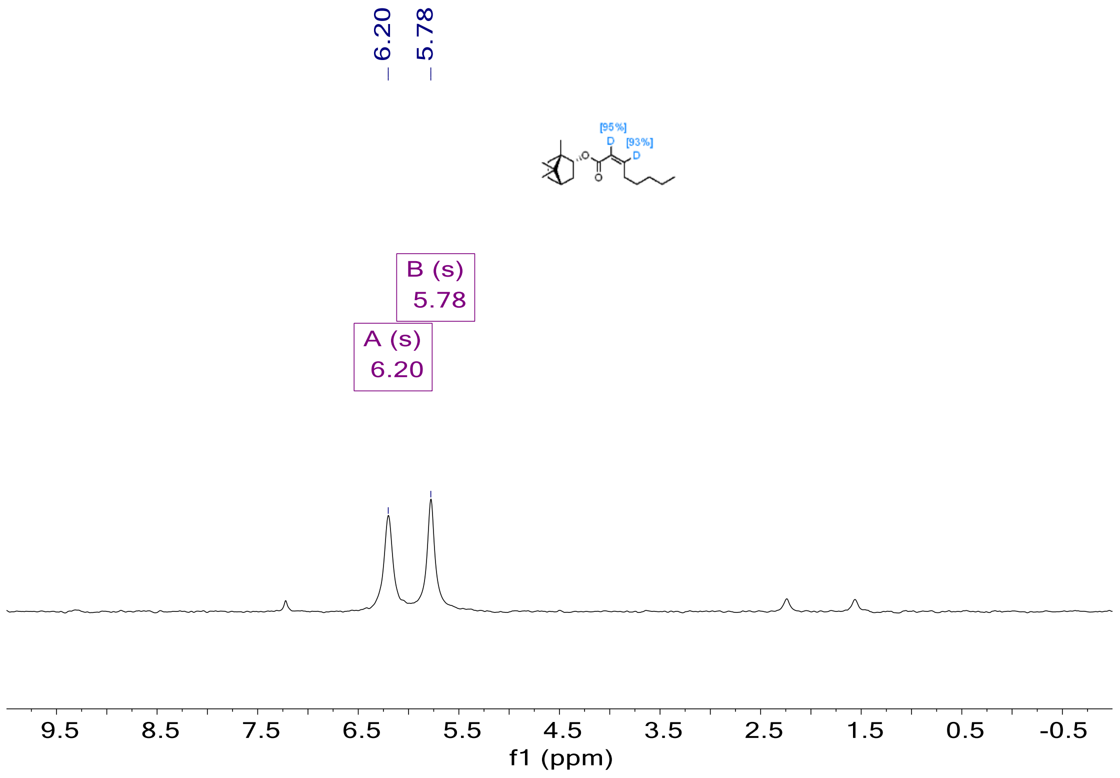

^{2}H NMR (61 MHz, 298 K, Chloroform) spectra for **27**

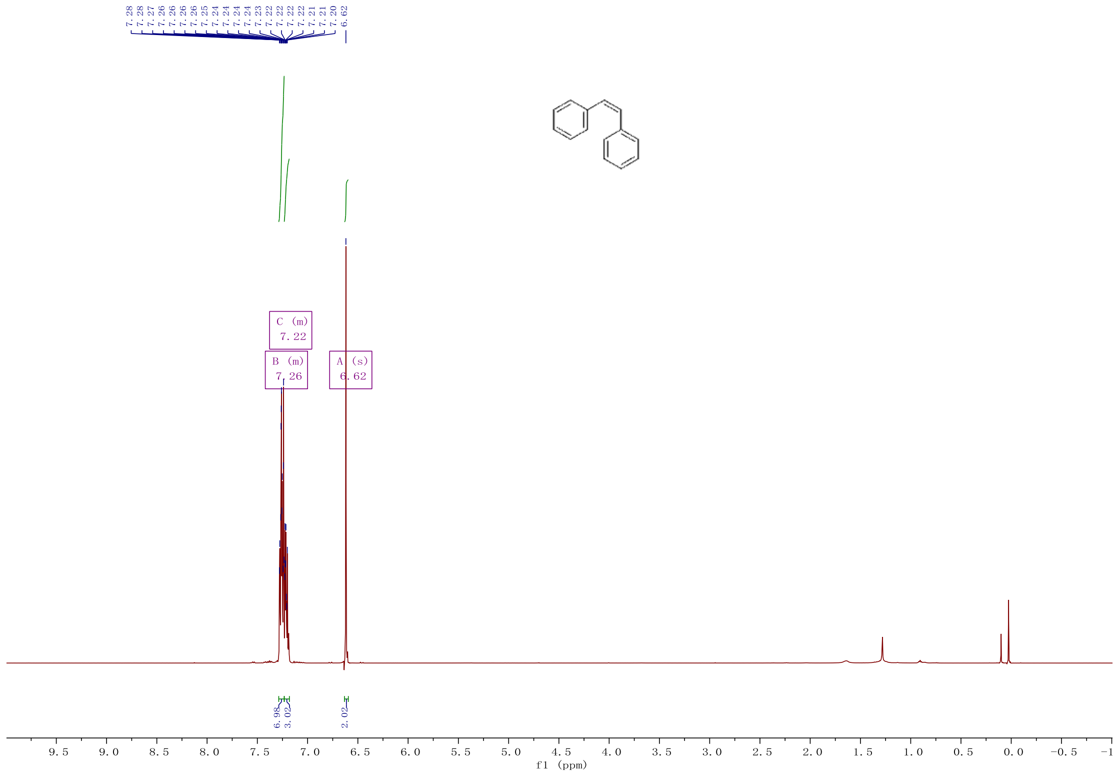

^{1}H NMR (500 MHz, 298 K, Chloroform-*d*) spectra for **28**

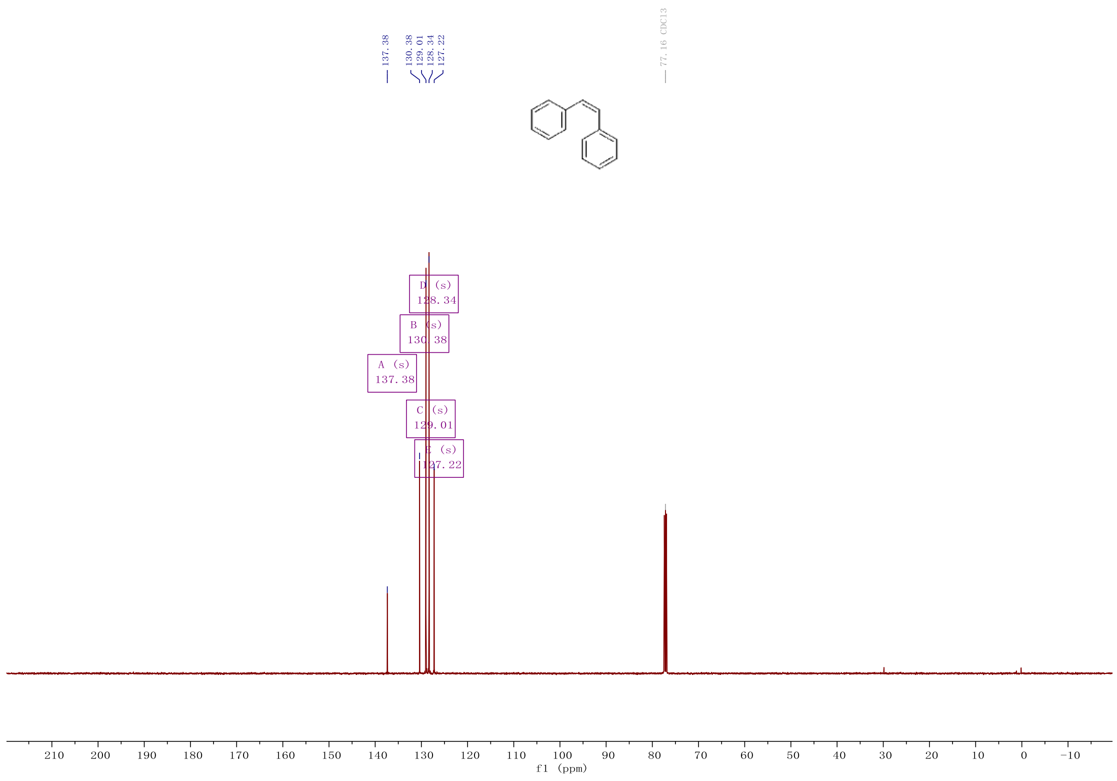

^{13}C NMR (126 MHz, 298 K, Chloroform-*d*) spectra for **28**

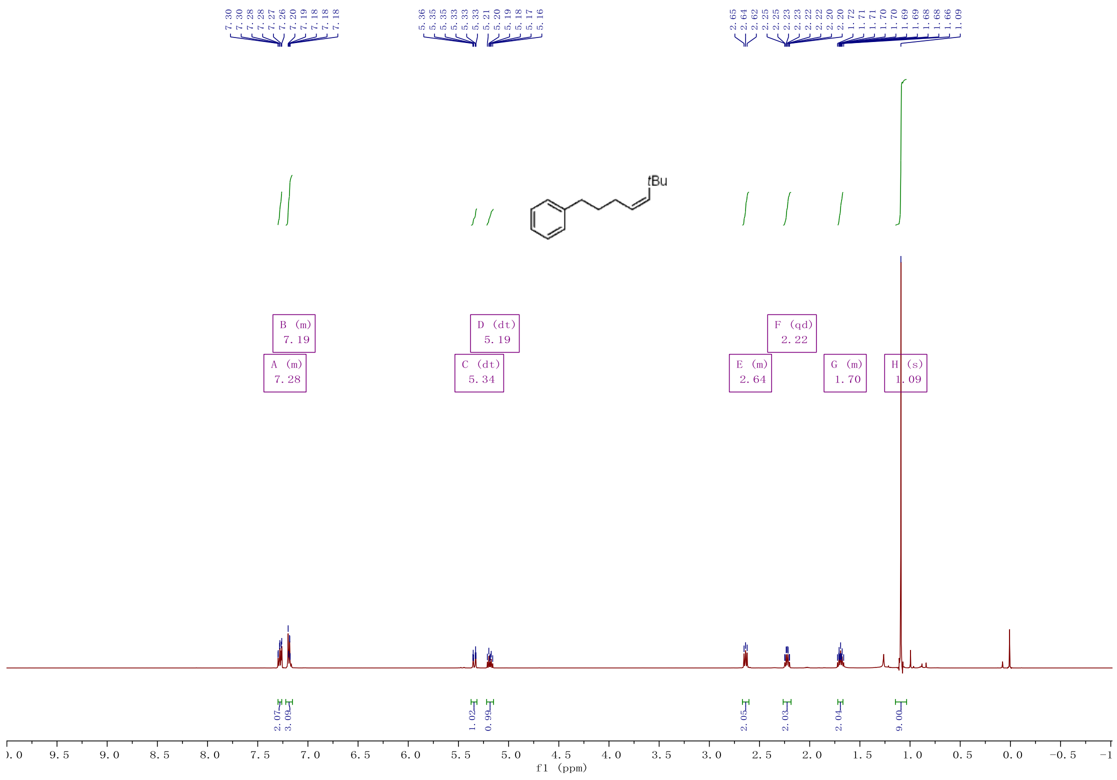

^{1}H NMR (500 MHz, 298 K, Chloroform-*d*) spectra for **29**

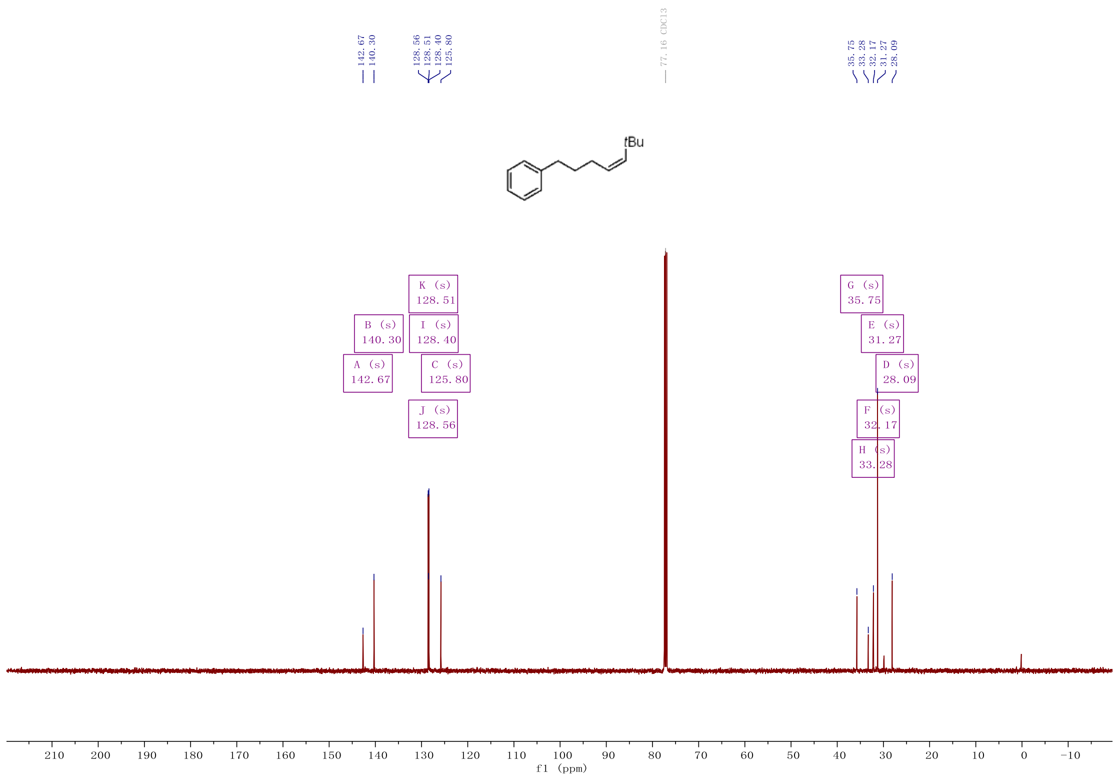

^{13}C NMR (126 MHz, 298 K, Chloroform-*d*) spectra for **29**

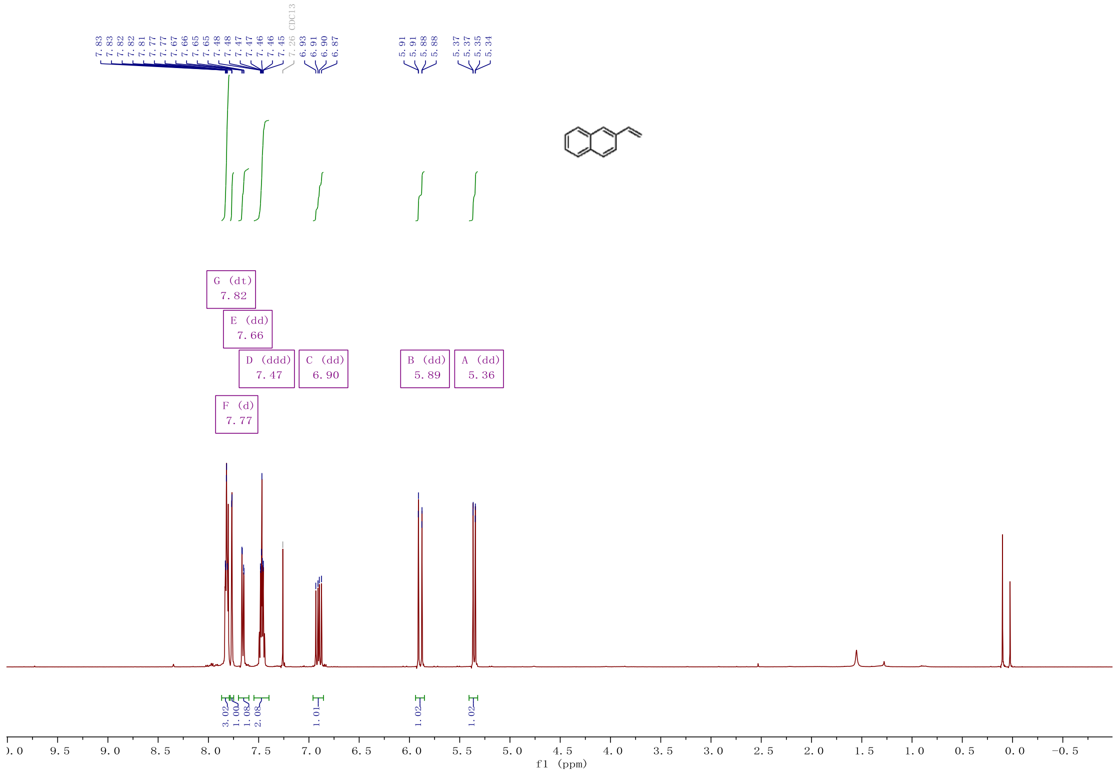

^{1}H NMR (500 MHz, 298 K, Chloroform-*d*) spectra for **30**

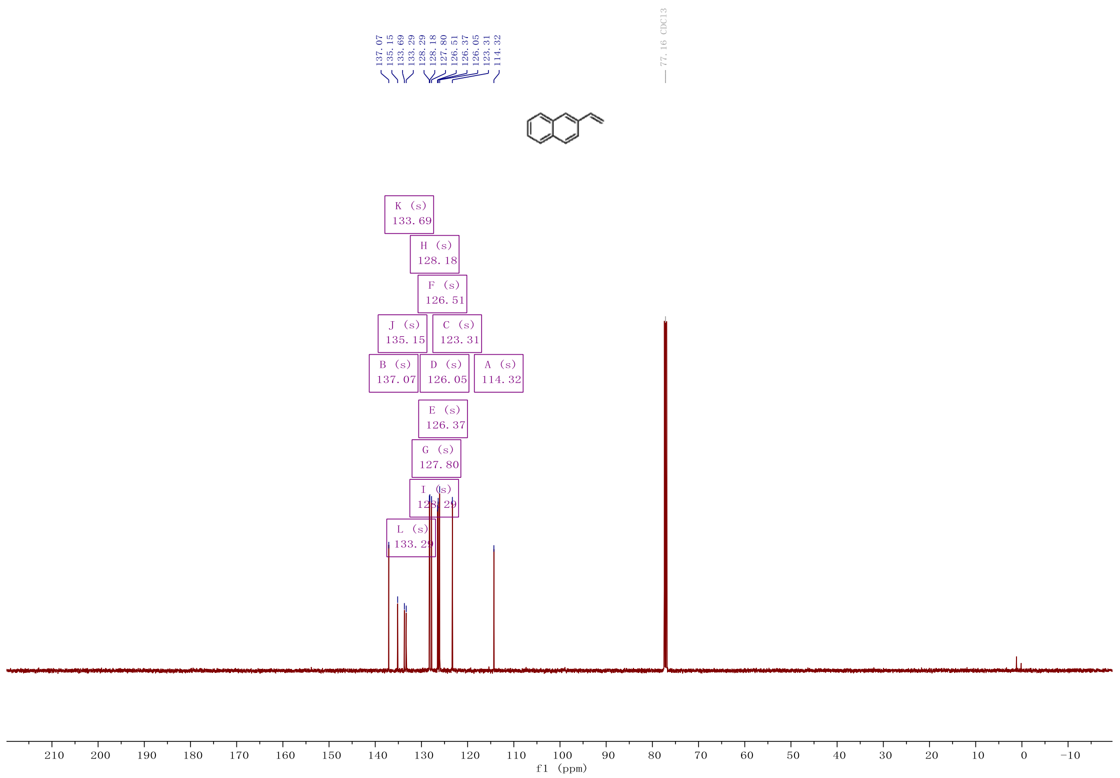

^{13}C NMR (126 MHz, 298 K, Chloroform-*d*) spectra for **30**

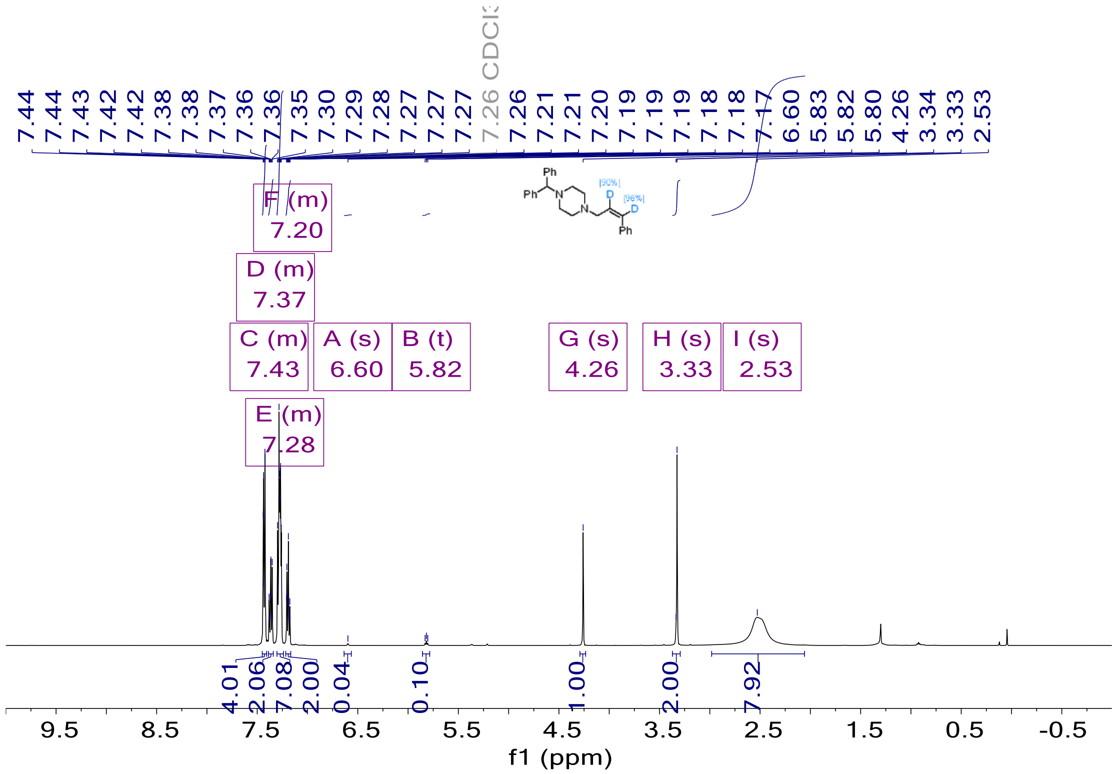

^{1}H NMR (500 MHz, 298 K, Chloroform-*d*) spectra for **32**

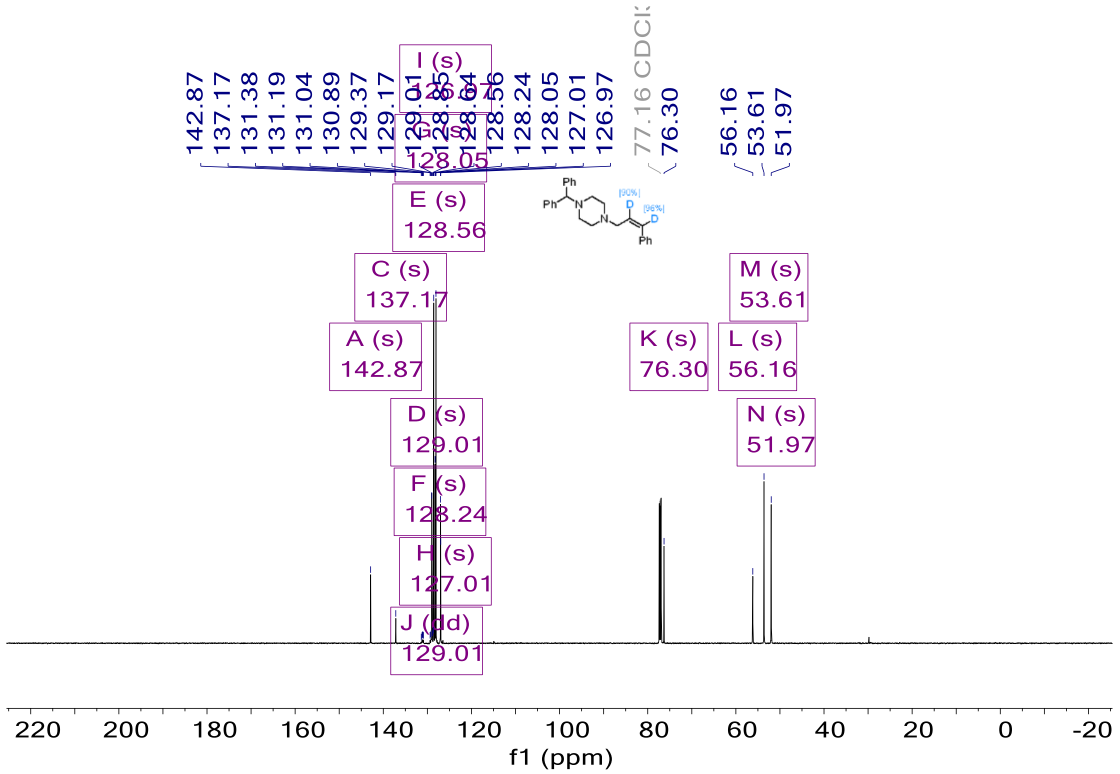

^{13}C NMR (151 MHz, 298 K, Chloroform-*d*) spectra for **32**

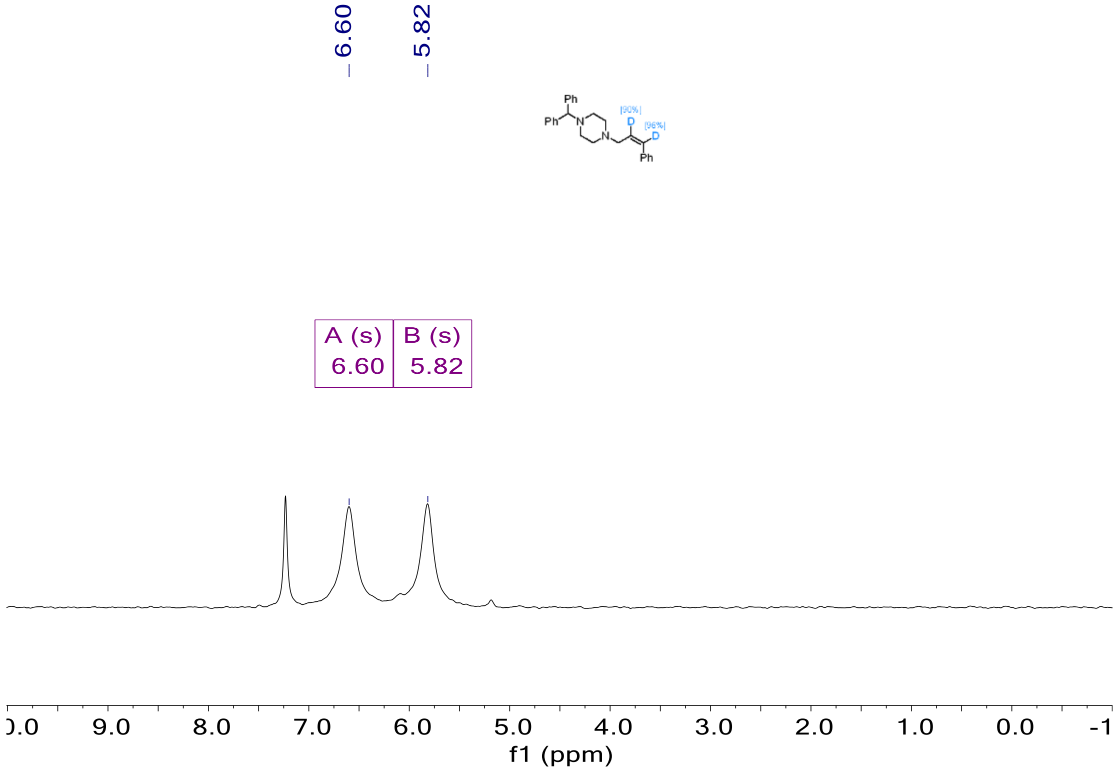

^{2}H NMR (61 MHz, 298 K, Chloroform) spectra for **32**

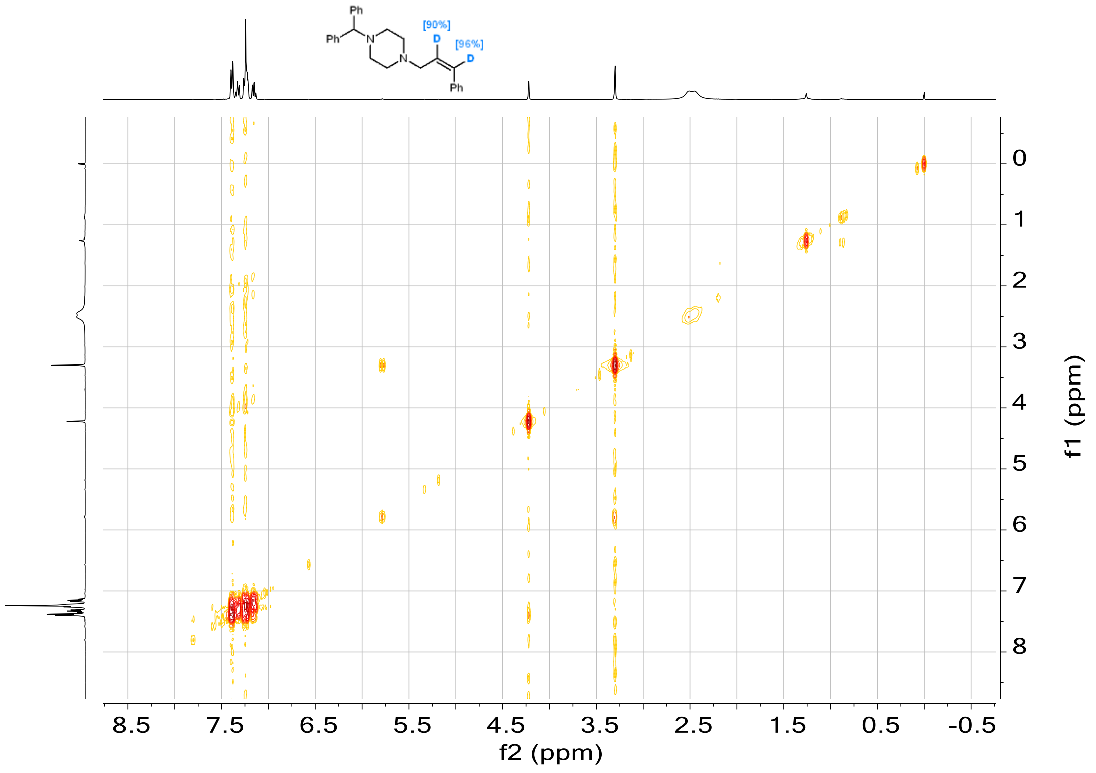

H,H COSY 90° (500 MHz, 298 K, Chloroform-*d*) spectra for **32**

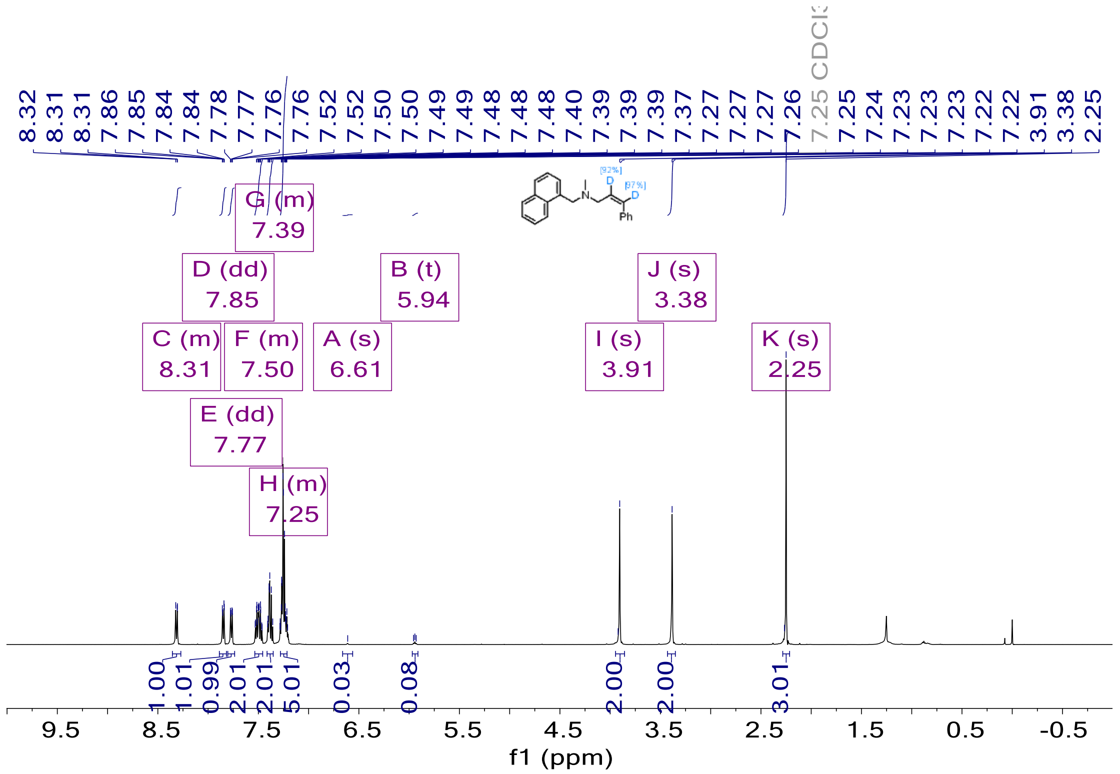

^{1}H NMR (500 MHz, 298 K, Chloroform-*d*) spectra for **34**

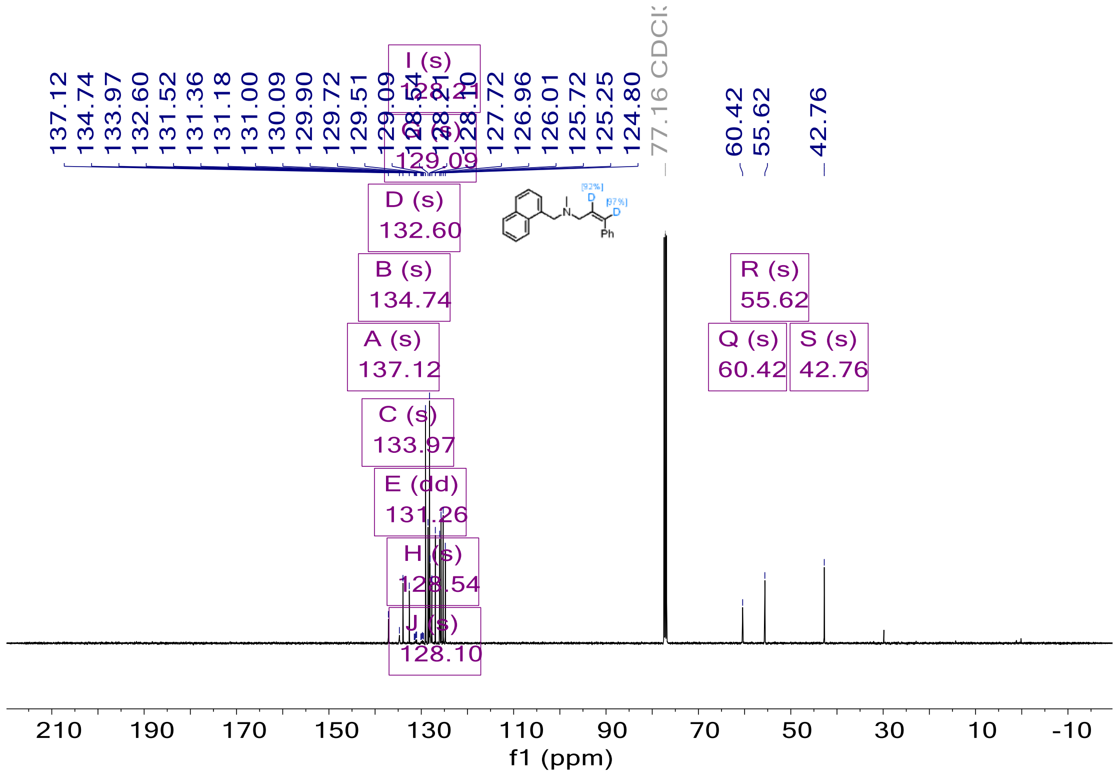

^{13}C NMR (126 MHz, 298 K, Chloroform-*d*) spectra for **34**

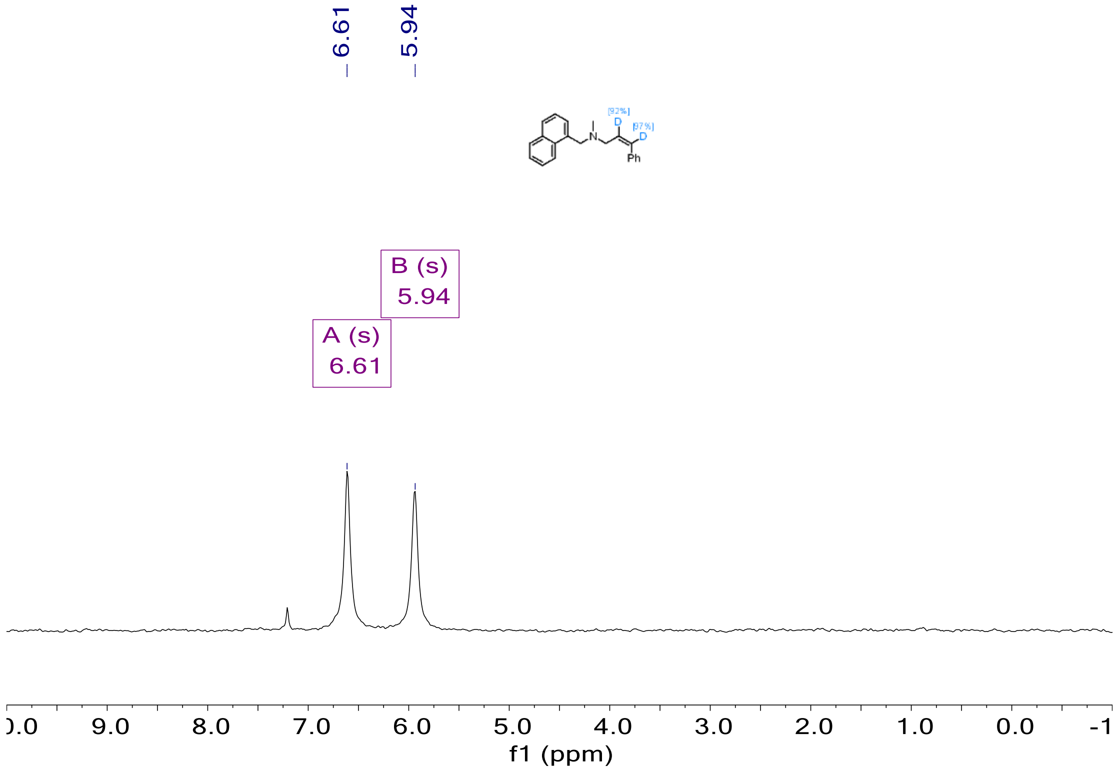

^{2}H NMR (92 MHz, 298 K, Chloroform) spectra for **34**

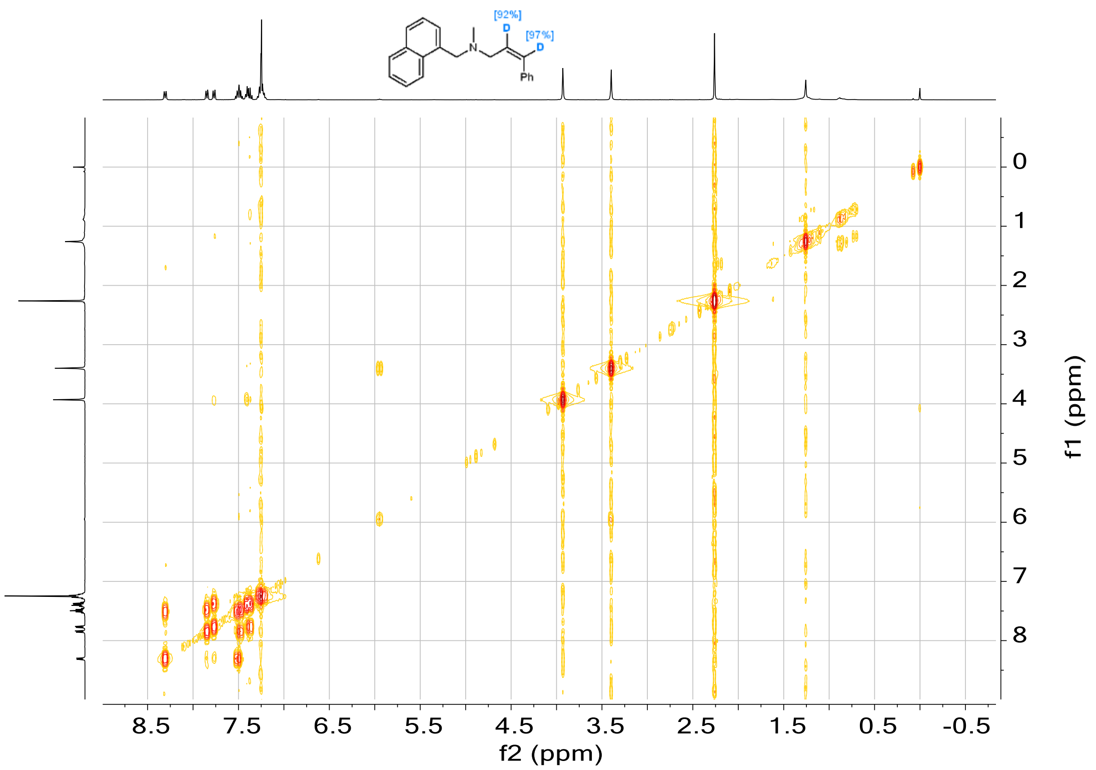

H,H COSY 90° (500 MHz, 298 K, Chloroform-*d*) spectra for **34**

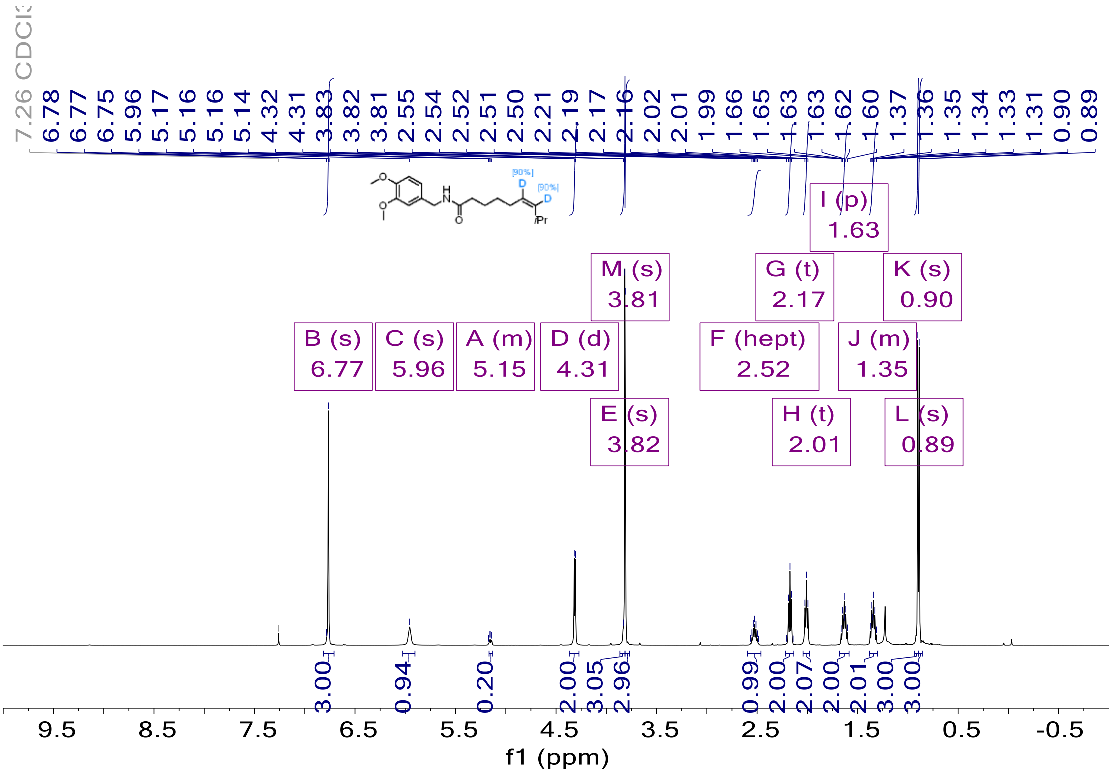

^{1}H NMR (500 MHz, 298 K, Chloroform-*d*) spectra for **36**

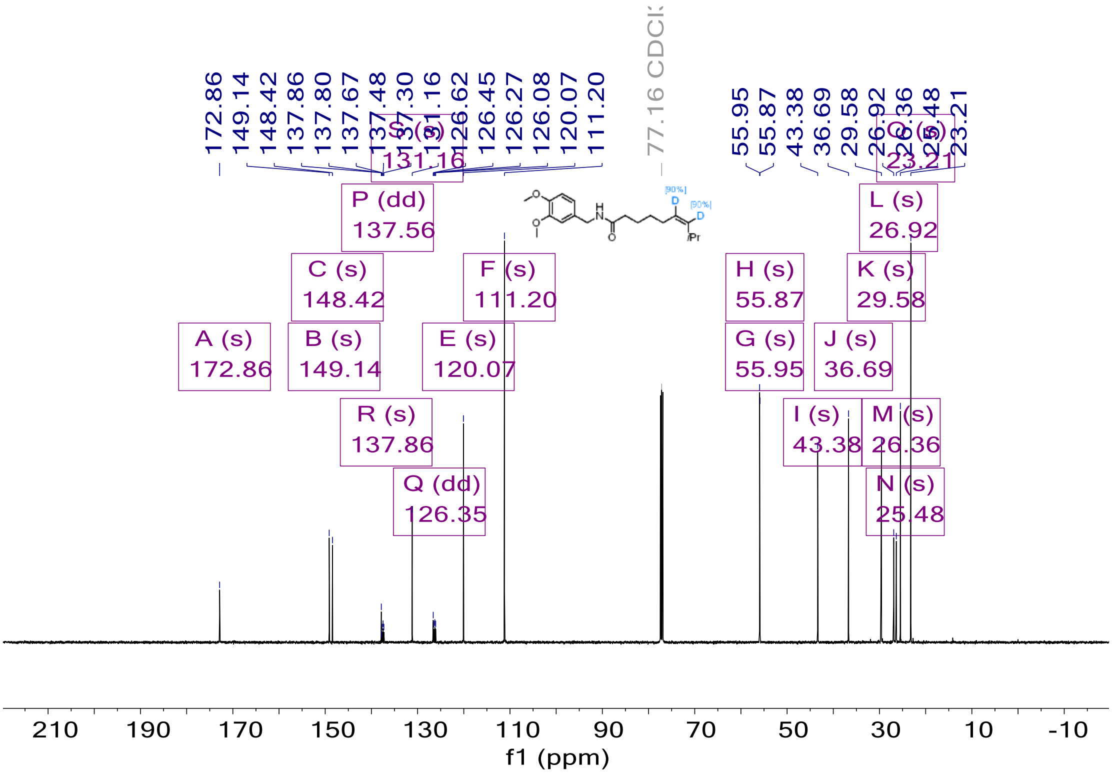

^{13}C NMR (126 MHz, 298 K, Chloroform-*d*) spectra for **36**

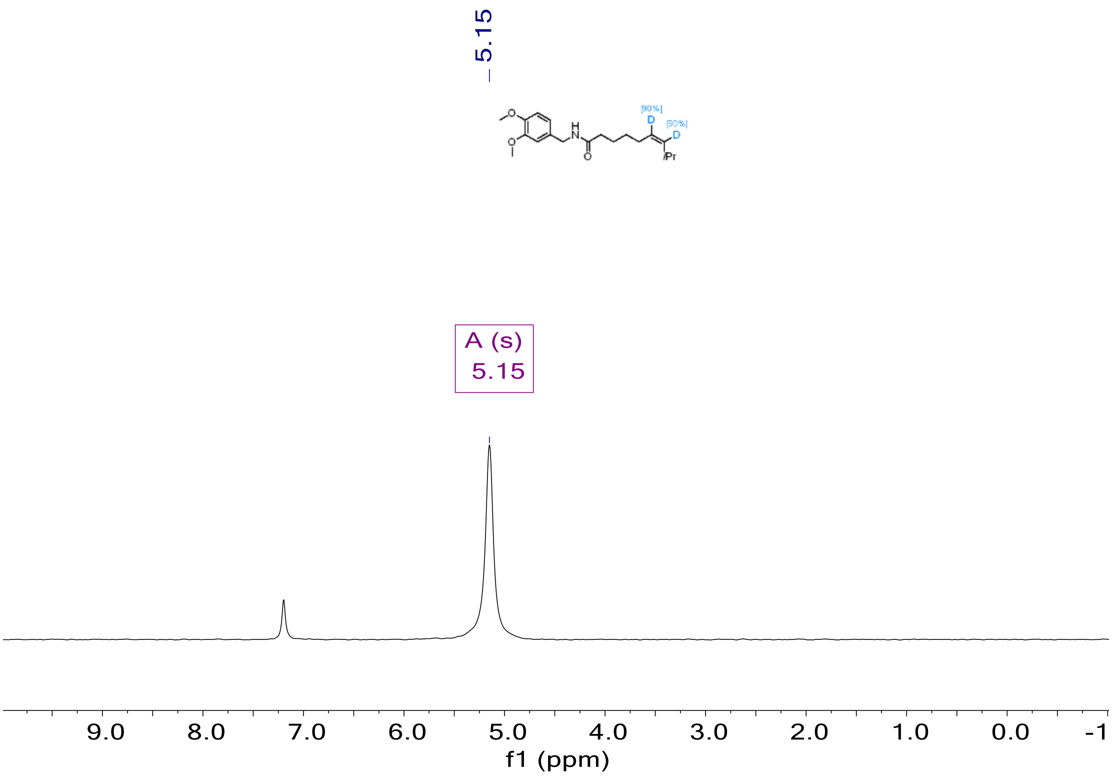

^{2}H NMR (61 MHz, 298 K, Chloroform) spectra for **36**

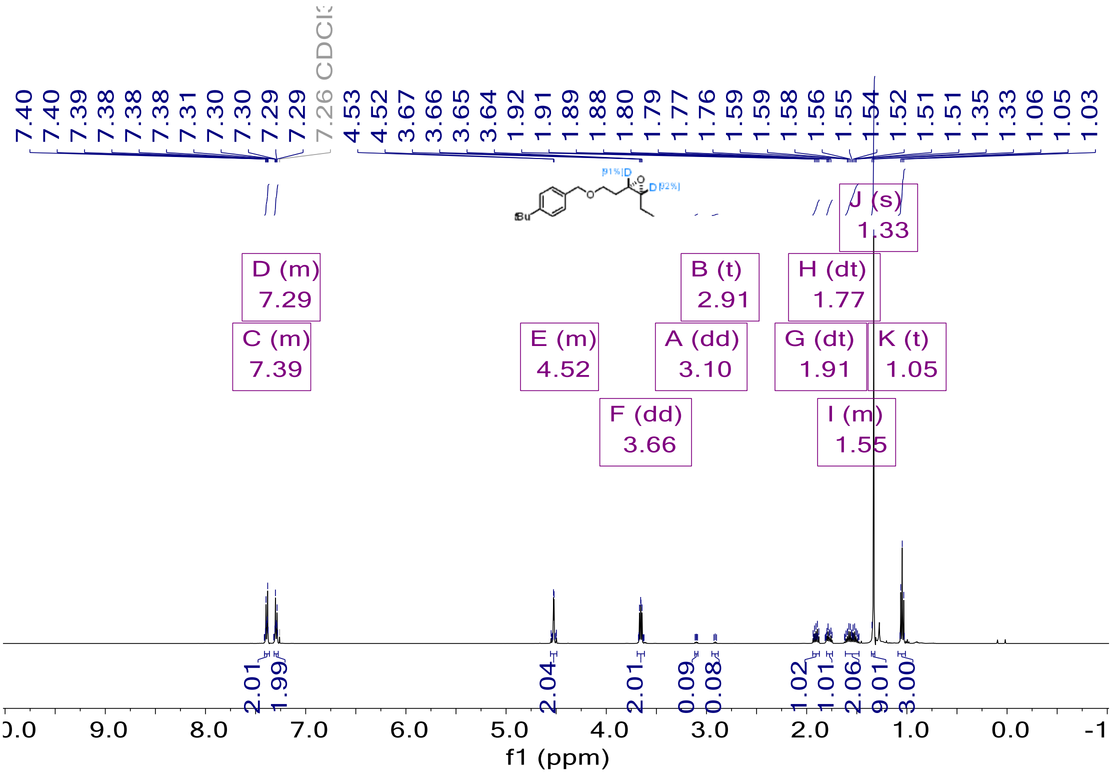

^{1}H NMR (500 MHz, 298 K, Chloroform-*d*) spectra for **37**

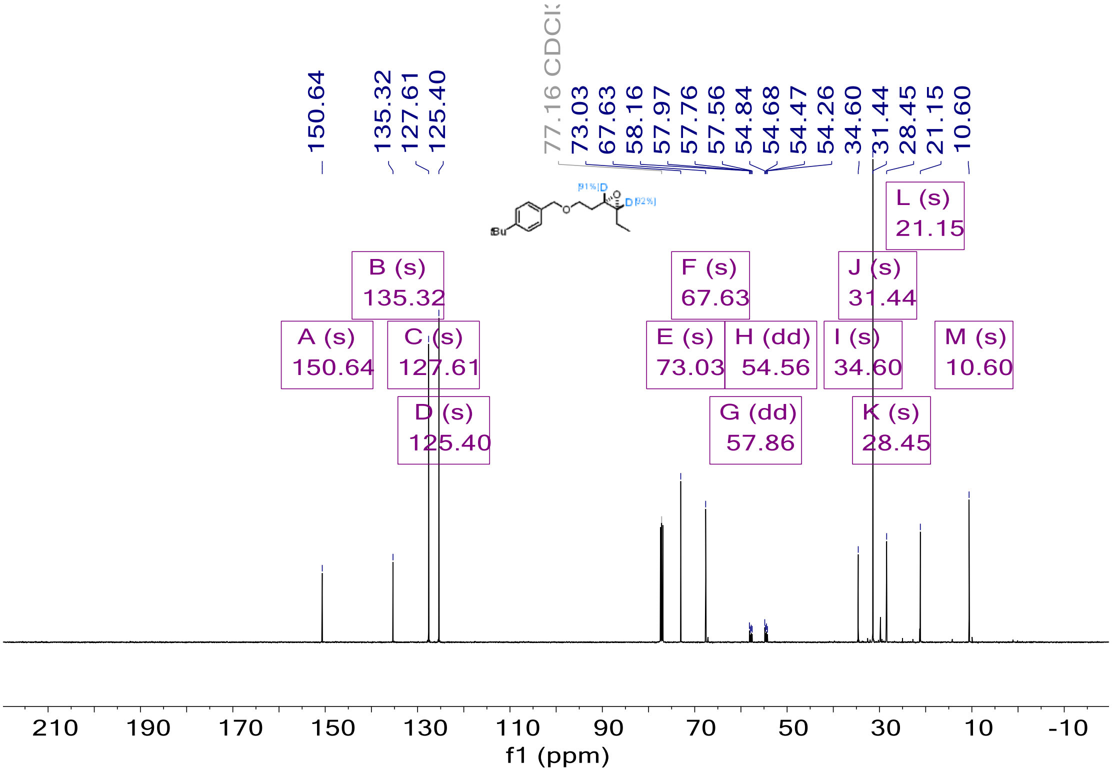

^{13}C NMR (126 MHz, 298 K, Chloroform-*d*) spectra for **37**

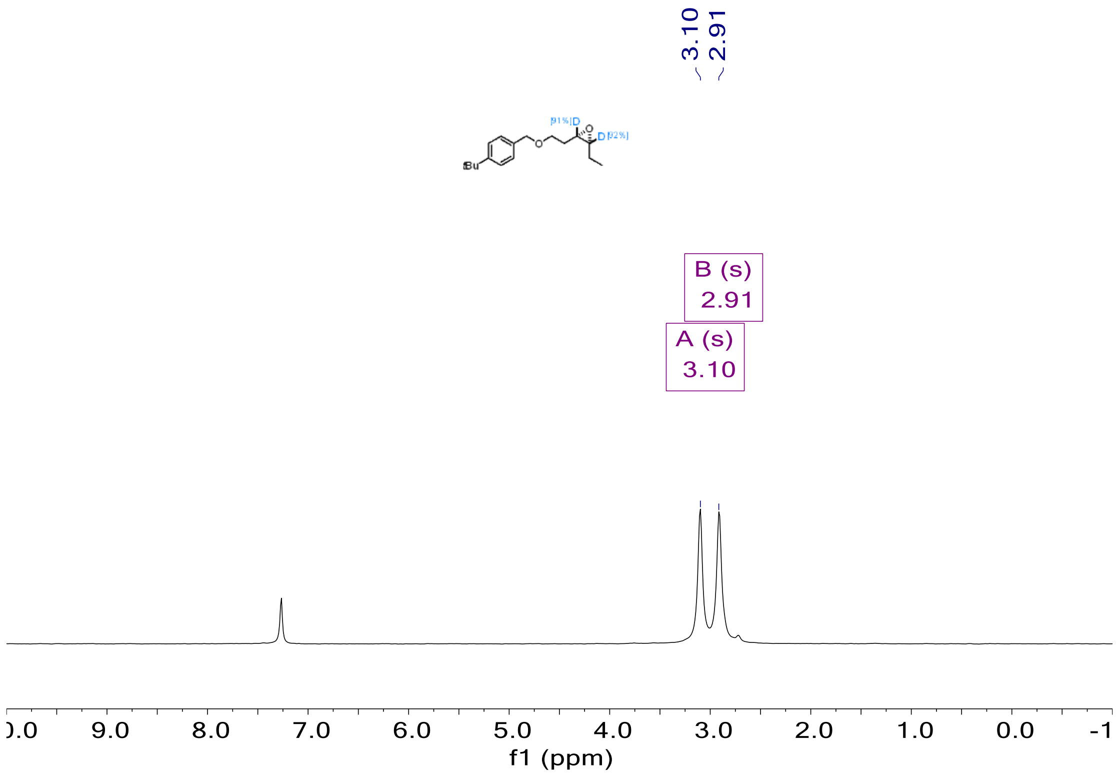

^{2}H NMR (92 MHz, 298 K, Chloroform) spectra for **37**

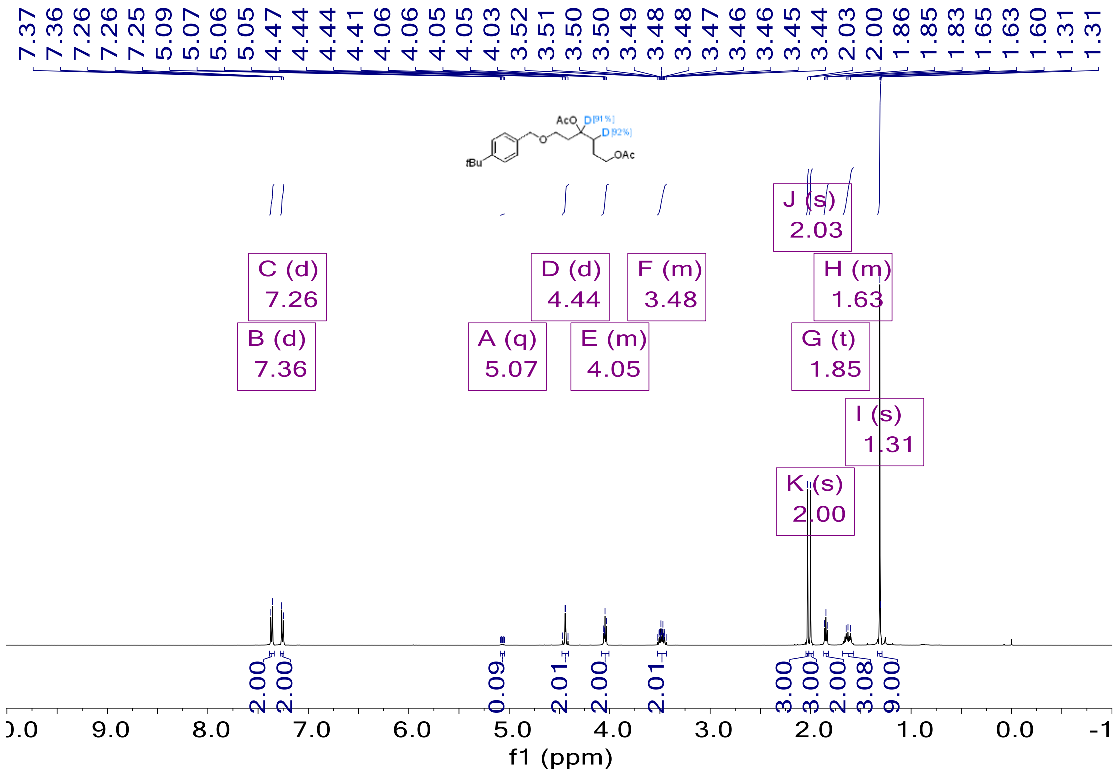

^{1}H NMR (500 MHz, 298 K, Chloroform-*d*) spectra for **38**

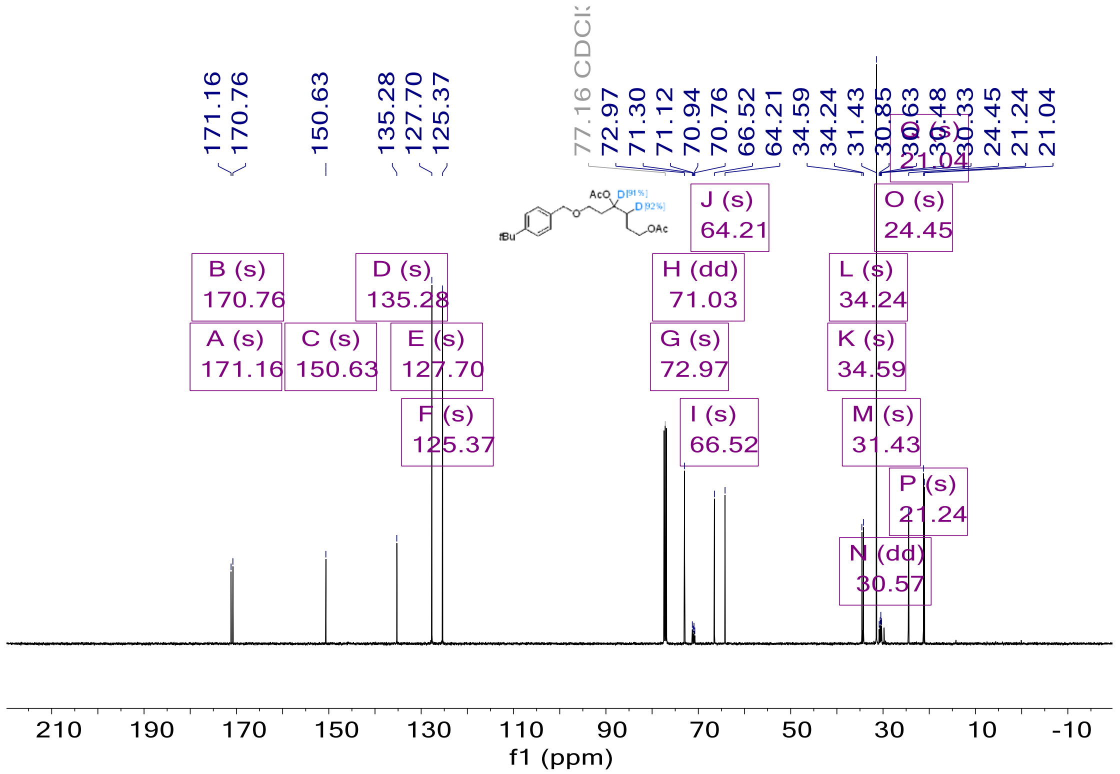

^{13}C NMR (126 MHz, 298 K, Chloroform-*d*) spectra for **38**

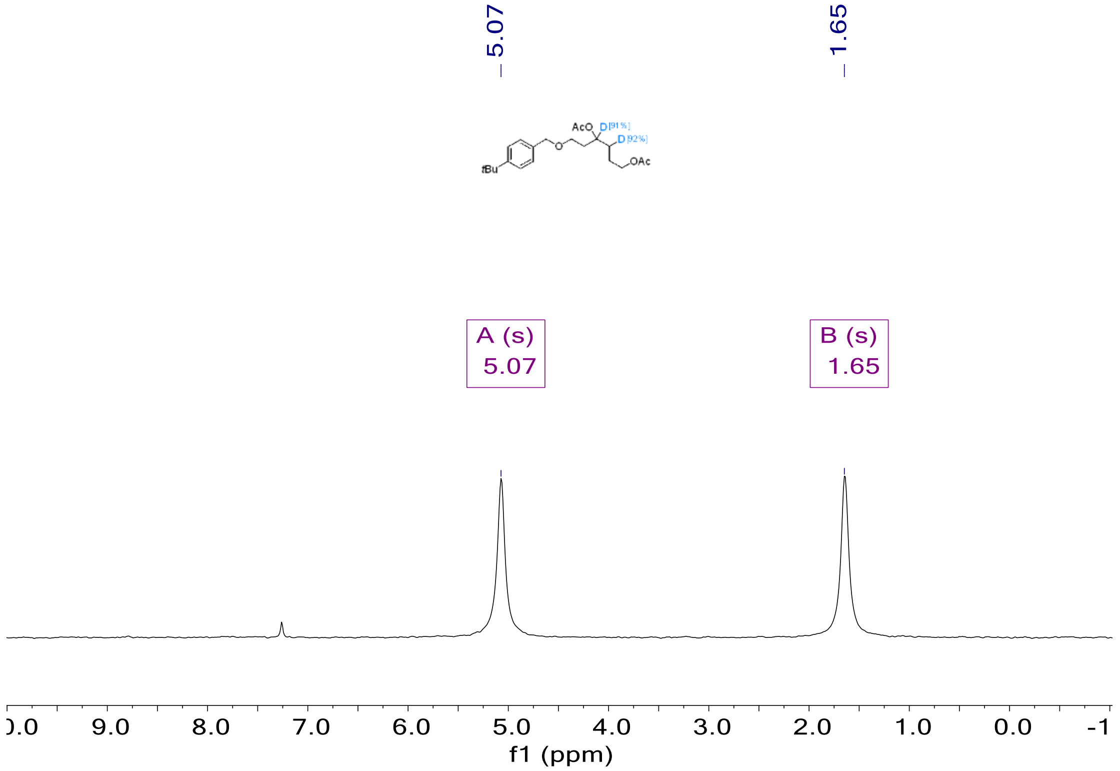

^{2}H NMR (92 MHz, 298 K, Chloroform) spectra for **38**

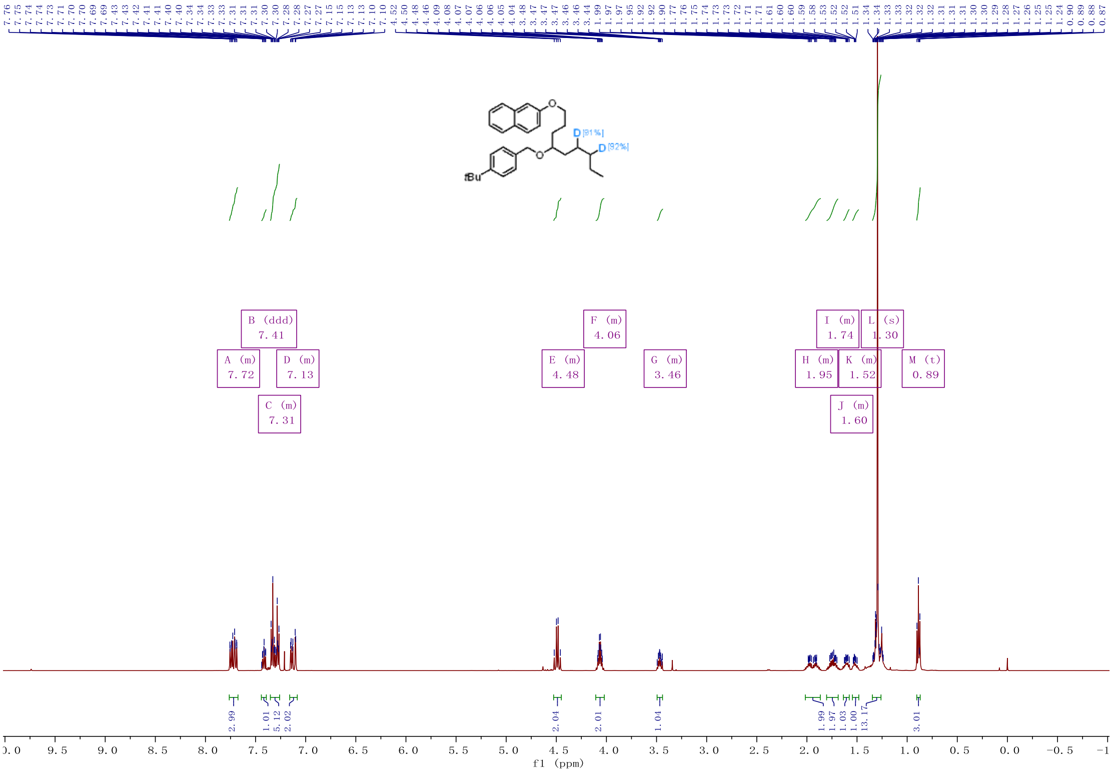

^{1}H NMR (500 MHz, 298 K, Chloroform-*d*) spectra for **39**

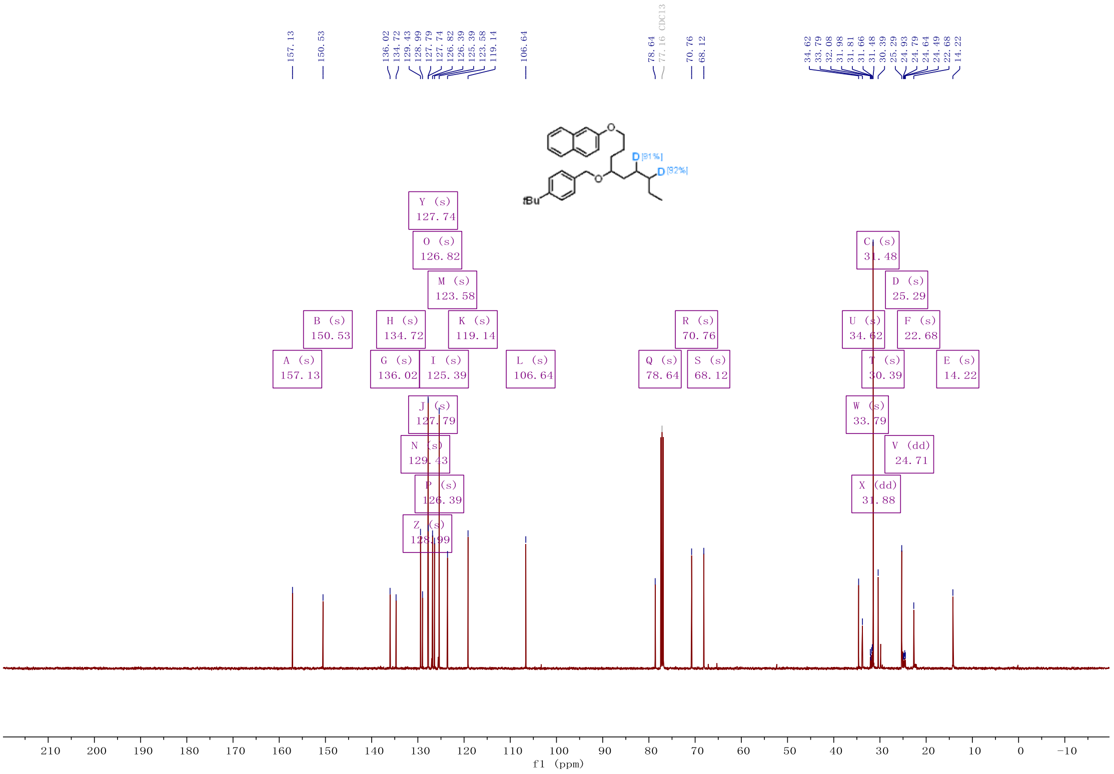

^{13}C NMR (126 MHz, 298 K, Chloroform-*d*) spectra for **39**

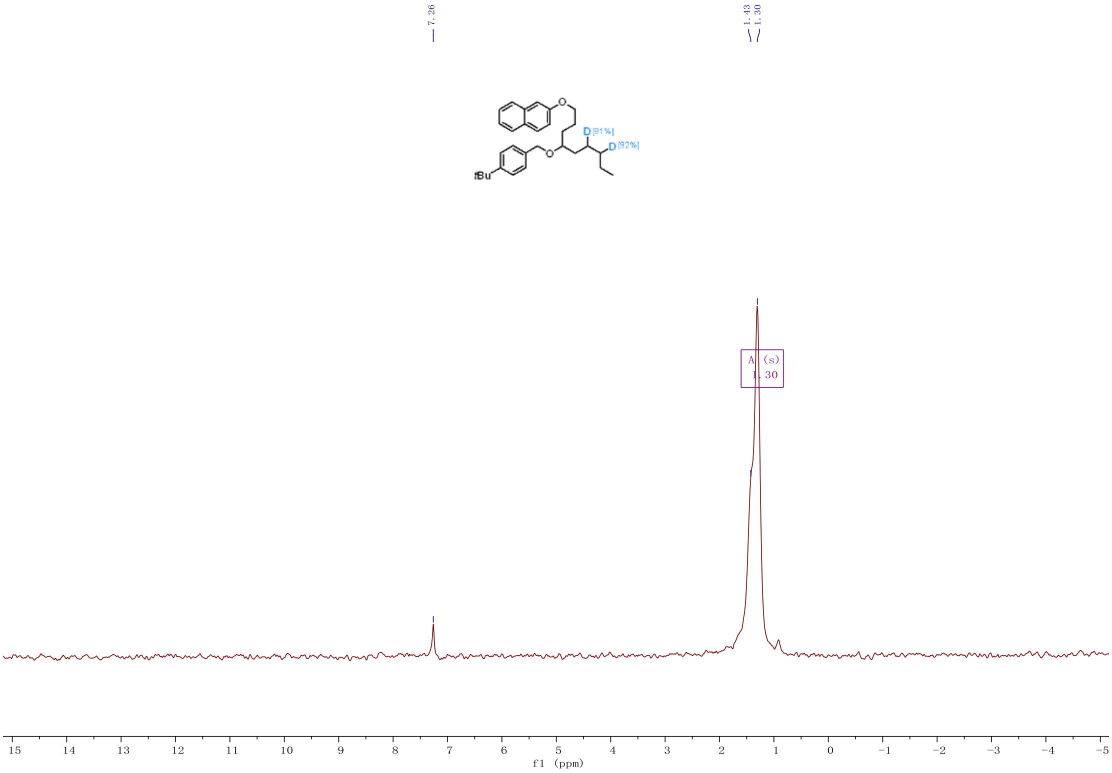

^2H NMR (61 MHz, 298 K, Chloroform) spectra for **39**

Supplementary References

1. Gnaim, S., *et al.* Cobalt-electrocatalytic HAT for functionalization of unsaturated C-C bonds. *Nature* **605**, 687-695 (2022). https://doi.org/10.1038/s41586-022-04595-3
2. Akai, Y., *et al.* Asymmetric Suzuki-Miyaura cross-coupling of 1-bromo-2-naphthoates using the helically chiral polymer ligand PQXphos. *Chem. Commun.* **51**, 7211-7214 (2015). https://doi.org/10.1039/c5cc01074h
3. Li, W., *et al.* Synthesis of axially chiral alkenylboronates through combined copper- and palladium-catalysed atroposelective arylboration of alkynes. *Nat. Synth.* **2**, 140-151 (2023). https://doi.org/10.1038/s44160-022-00201-6
4. Liu, X., *et al.* Migratory Hydrogenation of Terminal Alkynes by Base/Cobalt Relay Catalysis. *Angew. Chem. Int. Ed.* **59**, 6750-6755 (2020). https://doi.org/10.1002/anie.201916014
5. Teplý, F., *et al.* A Convenient Route to 2-Hydroxy- and 2,15-Dihydroxyhexahelicene. *Eur. J. Org. Chem.* **2007**, 4244-4250 (2007). https://doi.org/10.1002/ejoc.200700381
6. Fu, S. M., *et al.* Ligand-Controlled Cobalt-Catalyzed Transfer Hydrogenation of Alkynes: Stereodivergent Synthesis of Z- and E-Alkenes. *J. Am. Chem. Soc.* **138**, 8588-8594 (2016). https://doi.org/10.1021/jacs.6b04271
7. Melzig, L., *et al.* Pd- and Ni-catalyzed cross-coupling reactions of functionalized organozinc reagents with unsaturated thioethers. *Chem. Eur. J.* **17**, 2948-2956 (2011). https://doi.org/10.1002/chem.201002850
8. Nasrollahzadeh, M., *et al.* Facile synthesis of Pd nanoparticles supported on a novel Schiff base modified chitosan-kaolin: Antibacterial and catalytic activities in Sonogashira coupling reaction. *J. Organom. Chem.* **945**, 121849 (2021). https://doi.org/10.1016/j.jorganchem.2021.121849
9. Ruengsangtongkul, S., *et al.* Rate Enhancement in CAN-Promoted $Pd(PPh_3)_2Cl_2$-Catalyzed Oxidative Cyclization: Synthesis of 2-Ketofuran-4-carboxylate Esters. *Org. Lett.* **21**, 2514-2517 (2019). https://doi.org/10.1021/acs.orglett.9b00053
10. Sil, S., *et al.* Cross-Coupling Between Aryl Halides and Aryl Alkynes Catalyzed by an Odd Alternant Hydrocarbon. *Chem. Eur. J.* **30**, e202400895 (2024). https://doi.org/10.1002/chem.202400895
11. Xing, Y. K., *et al.* Divergent rhodium-catalyzed electrochemical vinylic C-H

annulation of acrylamides with alkynes. *Nat. Commun.* **12**, 930 (2021). https://doi.org/10.1038/s41467-021-21190-8

12. Ye, M.*, et al.* Arylation of Terminal Alkynes: Transition-Metal-Free Sonogashira-Type Coupling for the Construction of C(sp)-C(sp^2) Bonds. *Org. Lett.* **25**, 1787-1792 (2023). https://doi.org/10.1021/acs.orglett.3c00586
13. Zhang, W. W.*, et al.* Palladium-catalyzed decarboxylative coupling of alkynyl carboxylic acids with benzyl halides or aryl halides. *J. Org. Chem.* **75**, 5259-5264 (2010). https://doi.org/10.1021/jo1010284
14. Zhao, C. Q.*, et al.* Water as a Hydrogenating Agent: Stereodivergent Pd-Catalyzed Semihydrogenation of Alkynes. *Org. Lett.* **21**, 1412-1416 (2019). https://doi.org/10.1021/acs.orglett.9b00148
15. Pflueger, J. J.*, et al.* Magnesiate Addition/Ring-Expansion Strategy To Access the 6-7-6 Tricyclic Core of Hetisine-Type C(20)-Diterpenoid Alkaloids. *Org. Lett.* **19**, 4632-4635 (2017). https://doi.org/10.1021/acs.orglett.7b02260
16. Vallribera, A.*, et al.* Sonogashira Cross-Coupling Using Carbon Aerogel Doped with Palladium Nanoparticles; A Recoverable and Reusable Catalyst. *Synthesis* **2007**, 3068-3072 (2007). https://doi.org/10.1055/s-2007-983888
17. Park, J. Y.*, et al.* Synthesis of Benzoisoxazole Derivatives and Evaluation of Inhibitory Potency against Cholinesterase for Alzheimer's Disease Therapeutics. *Bull. Korean Chem. Soc.* **37**, 1464-1471 (2016). https://doi.org/10.1002/bkcs.10891
18. Wang, Z. C.*, et al.* Capsaicin derivatives with nitrothiophene substituents: Design, synthesis and antibacterial activity against multidrug-resistant S. aureus. *Eur. J. Med. Chem.* **198**, 112352 (2020). https://doi.org/10.1016/j.ejmech.2020.112352
19. Han, X.*, et al.* Copper-catalysed, diboron-mediated cis-dideuterated semihydrogenation of alkynes with heavy water. *Chem. Commun.* **55**, 6922-6925 (2019). https://doi.org/10.1039/c9cc03213d
20. Masing, F.*, et al.* Light Mediated Preparation of Palladium Nanoparticles as Catalysts for Alkyne cis-Semihydrogenation. *Org. Lett.* **19**, 2658-2661 (2017). https://doi.org/10.1021/acs.orglett.7b00999
21. Murugappan, K.*, et al.* Supported molybdenum oxides as effective catalysts for the catalytic fast pyrolysis of lignocellulosic biomass. *Green Chem.* **18**, 5548-5557 (2016). https://doi.org/10.1039/c6gc01189f
22. Liu, W.*, et al.* A General Regioselective Synthesis of Alcohols by Cobalt-

Catalyzed Hydrogenation of Epoxides. *Angew. Chem. Int. Ed.* **59**, 11321-11324 (2020). https://doi.org/10.1002/anie.202002844

23. Li, X., *et al.* Regio- and enantioselective remote dioxygenation of internal alkenes. *Nat. Chem.* **15**, 862-871 (2023). https://doi.org/10.1038/s41557-023-01192-3

24. Wang, J. W., *et al.* Nickel-Catalyzed Remote Asymmetric Hydroalkylation of Alkenyl Ethers to Access Ethers of Chiral Dialkyl Carbinols. *J. Am. Chem. Soc.* **145**, 10411-10421 (2023). https://doi.org/10.1021/jacs.3c02950
